# Photocatalytic Boryl Radicals Triggered Sequential B─N/C─N Bond Formation to Assemble Boron‐Handled Pyrazoles

**DOI:** 10.1002/advs.202306728

**Published:** 2023-11-29

**Authors:** Yang Xie, Ruilong Zhang, Ze‐Le Chen, Mengtao Rong, Hui He, Shaofei Ni, Xiang‐Kui He, Wen‐Jing Xiao, Jun Xuan

**Affiliations:** ^1^ Anhui Province Key Laboratory of Chemistry for Inorganic/Organic Hybrid Functionalized Materials and Key Laboratory of Functional Inorganic Materials of Anhui Province College of Chemistry & Chemical Engineering Anhui University Hefei Anhui 230601 P. R. China; ^2^ Department of Chemistry and Key Laboratory for Preparation and Application of Ordered Structural Materials of Guangdong Province Shantou University Shantou Guangdong 515063 P. R. China; ^3^ Key Laboratory of Pesticide and Chemical Biology Ministry of Education College of Chemistry Central China Normal University Wuhan Hubei 430079 P. R. China

**Keywords:** cascade radical cyclization, heterocycle synthesis, photochemistry, reaction mechanisms, vinyldiazo reagents

## Abstract

Vinyldiazo compounds are one of the most important synthons in the construction of a cyclic ring. Most photochemical transformations of vinyldiazo compounds are mainly focusing on utilization of their C═C bond site, while reactions taking place at terminal nitrogen atom are largely unexplored. Herein, a photocatalytic cascade radical cyclization of LBRs with vinyldiazo reagents through sequential B─N/C─N bond formation is described. The reaction starts with the addition of LBRs (Lewis base–boryl radicals) at diazo site, followed by intramolecular radical cyclization to access a wide range of important boron‐handled pyrazoles in good to excellent yields. Control experiments, together with detailed mechanism studies well explain the observed reactivity. Further studies demonstrate the utility of this approach for applications in pharmaceutical and agrochemical research.

## Graphical abstract

A mild strategy for the synthesis of boron‐handled pyrazoles through photocatalytic cascade radical cyclization of LBRs (Lewis base‐boryl radicals) with vinyldiazo reagents is described here. The reaction starts with the addition of LBRs at diazo site, followed by intramolecular radical cyclization to access a wide range of important boron‐handled pyrazoles in good to excellent yields. Control experiments, together with detailed mechanism studies well explain the observed reactivity.

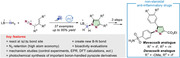

## 1 Introduction

**Scheme 1:**
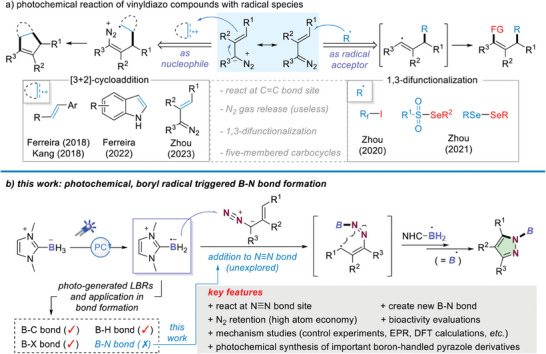
Background and evolution of a strategy for radical B─N bond formation.

Radical cascade cyclization reactions are highly valued in organic synthesis as robust strategies to the de novo construction of ring systems omnipresent in various biologically important structures.^[^ ^1^ ^]^ Bearing with conjugated functionalities of both alkene and diazo groups, vinyldiazo compounds have proven to be one of the most versatile building blocks in contemporary chemical synthesis.^[^ ^2^ ^]^ Typically, decomposition of vinyldiazo reagents in presence of transition metals (Rh, Au, Cu, Ag, etc.) forms electrophilic metallo–vinylcarbenes as dipolar adducts which have been well‐applied in many cyclization processes to access diverse carbo‐ and heterocycles.^[^ ^3^ ^]^ On the contrary, compared with those widely explored in polar chemistry, reaction of vinyldiazo compounds with radicals and related active species remains largely unexplored. In 2018, Ferreira and co‐workers^[^ ^4a^ ^]^ found that vinyldiazo compounds could serve as nucleophiles to intercept the photogenerated radical cations, thus providing a powerful approach to access cyclopentenes (**Scheme** **1**a, left). Subsequently, Kang et al.^[^ ^4b^ ^]^ realized a similar light‐mediated radical cation [3+2]‐cycloaddition of electron‐rich alkenes and vinyl diazoesters with Fe(phen)_3_(PF_6_)_3_ as photoredox catalyst. Later, Ferreira group further expanded the strategy to dearomative photocatalyzed [3+2]‐cycloaddition between indoles and vinyldiazo reagents using a novel oxidizing [Cr(PMP_2_phen)_3_](BF_4_)_3_ photocatalyst.^[^ ^5^ ^]^ Very recently, Zhou, Koenigs et al.^[^ ^6^ ^]^ realized the photocatalytic self‐[3+2]‐cycloaddition of vinyldiazo reagents leading to a variety of cyclopentenyl α‐diazo compounds in good yields. Remarkably, Zhou group utilized vinyldiazo reagent as radical acceptor to realize its photochemical 1,3‐difunctionalization, including 1,3‐perfluoroalkyliodization,^[^ ^7a^ ^]^ 1,3‐selenosulfonylation and 1,3‐diselenation (Scheme 1a, right).^[^ ^7b^ ^]^ Despite these impressive advances provided very effective value‐added downstream transformations, the initial step of those reactions all occurred at C═C bond site of vinyldiazo reagents. Moreover, diazo moiety released through nitrogen gas extrusion, thus could not conserve the N_2_ function into the product structure. Further exploration of novel and synthetic useful radical transformation of vinyldiazo compound, especially with the utilization of its terminal nitrogen reaction site to facile synthesis biologically active nitrogen‐containing heterocycles, is still attractive, yet a challenge goal.

Organoboron compounds are versatile building blocks in chemical society, with applications that span natural isolates, pharmaceuticals, as well as functional materials.^[^ ^8^ ^]^ In the past years, tremendous progresses have been made on radical borylation reactions with the formation of boron centered radicals as the key intermediates, especially the relative stable Lewis base–boryl radicals (LBRs), e.g. NHC–boryl radicals (NHC─BH_2_^⚫^).^[^ ^9^, ^10^ ^]^ These active species are formed either through catalytic hydrogen atom abstraction or photoredox‐mediated single‐electron oxidation.^[^ ^10^ ^]^ To date, the photo‐generated nucleophilic LBRs^[^ ^11^ ^]^ have been widely applied in the formation of new B─C bonds through radical addition to C═Y bonds (Y═C, N).^[^ ^12^ ^]^ Remarkably, they can also be used to trigger hydrogen‐atom transfer (HAT)^[^ ^13^ ^]^ from electrophilic C─H bonds or halogen–atom transfer (XAT)^[^ ^14^ ^]^ from alkyl halides, enabling access to the corresponding alkyl radicals for further photochemical transformations. In the latter scenario, new B─H or B─X bonds are formed during the catalytic cycle. In sharp contrast, application of boryl radicals for the construction of B─N bonds are largely overlooked. Some sporadic reported examples have been so far invariably relied on thermal strategy.^[^ ^15^ ^]^ To the best of our knowledge, photochemical application of LBRs into the formation of new B─N bond is still unknown.

Against this background, we questioned whether a borylative radical cascade strategy, that is, LBRs‐trigged chemoselective addition/cyclization with vinyldiazo compounds, could serve as a handle to overcome present limitations. As shown in Scheme 1b, the designed reaction starts with chemo‐selective addition of LBRs to the terminal nitrogen atom of vinyldiazo reagents, followed by radical resonation and intramolecular cyclization, yielding a boron‐handled pyrazoles. Significantly, the formed boron‐handled pyrazoles would be of great interest in medicinal chemistry and material science, due to the fact that pyrazole is a core motif in numerous biologically relevant molecules.^[^ ^16^ ^]^ In addition, B─N bonds are also widely employed in various materials with intriguing electronic and optoelectronic properties.^[^ ^17^ ^]^ To achieve this goal, the following challenges should be considered: 1) identification of a suitable LBRs that can selectively add to unsaturated N─N bonds while being compatible with C═C bonds, and 2) exploitation of a photoredox catalytic system that maintains an efficient radical cascade cyclization over the vinyldiazo self‐1,5‐electrocyclization^[^ ^7^, ^18^ ^]^ or self‐[3+2] cycloaddition.^[^ ^6^ ^]^ Herein, we disclose the successful execution of this design plan.

## 2 Results and Discussion

### 2.1 Evaluation of Reaction Conditions

At the outset of our investigation, N‐heterocyclic carbene (NHC)‐borane **1a** and vinyldiazoacetate **2a** were used as model substrates in CH_3_CN under irradiation of 24 W blue LEDs at room temperature (**Table** **1**). Note that, all reactions were performed with a commercial light source to ensure reproducibility of the data. To our delight, the anticipated boron‐handled pyrazole **3** could be obtained in 71% isolated yield when using a combination of 2.0 mol% Ir(ppy)_2_(dtbbpy)PF_6_ as photocatalyst, and 1.0 equivalent DABCO (1,4‐diaza[2.2.2]bicyclooctane) as the base (entry 1). A slightly decrease of the yield was observed when the model reaction was carried out in 5 mmol scale. We further examined the influence of different reaction media, including DMC (dimethyl carbonate), EtOH and H_2_O, but no significant improvement of yield (22–60%) was achieved (entries 2–4). Replacement of Ir(ppy)_2_(dtbbpy)PF_6_ to other commonly used photoredox catalysts, such as Ru(bpy)_3_Cl_2_⚫6H_2_O and *fac*‐Ir(ppy)_3_ did not give better results (entries 5 and 6). The yield of **3** decreased to 15% when the reaction was performed in argon atmosphere (entry 7). It was found that the yield of **3** can still increase up to 54% without the addition of DABCO, which demonstrated the major function of DABCO should be as a base to accurate the deprotonation (Table 1, entry 8). Notably, control experiments confirmed that visible light irradiation and photoredox catalyst were all indispensable (entries 9 and 10).

**Table 1:** Optimization of reaction condition.

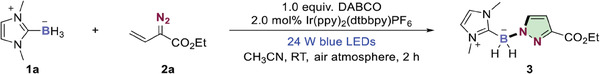		
Entry^a)^	Variation from the standard conditions	Yield (%)^b)^
1	none	71 (64)^c)^
2	DMC instead of CH_3_CN	54
3	EtOH instead of CH_3_CN	60
4	H_2_O instead of CH_3_CN	22
5	Ru(bpy)_3_Cl_2_⚫6H_2_O instead of Ir(ppy)_2_(dtbbpy)PF_6_	33
6	*fac*‐Ir(ppy)_3_ instead of Ir(ppy)_2_(dtbbpy)PF_6_	35
7	under an argon atmosphere	15
8	without DABCO	54
9	without Ir(ppy)_2_(dtbbpy)PF_6_	0
10	in dark	0

^a)^ Reaction conditions: **1a** (0.3 mmol), **2a** (0.9 mmol), DABCO (0.3 mmol), and Ir(ppy)_2_(dtbbpy)PF_6_ (2.0 mol%) in MeCN (1.0 mL), with 24 W Blue LEDs irradiation at rt for 2 h under air atmosphere;

^b)^ isolated yield;

^c)^ 5 mmol scale reaction.

### 2.2 Reaction Scope

**Scheme 2:**
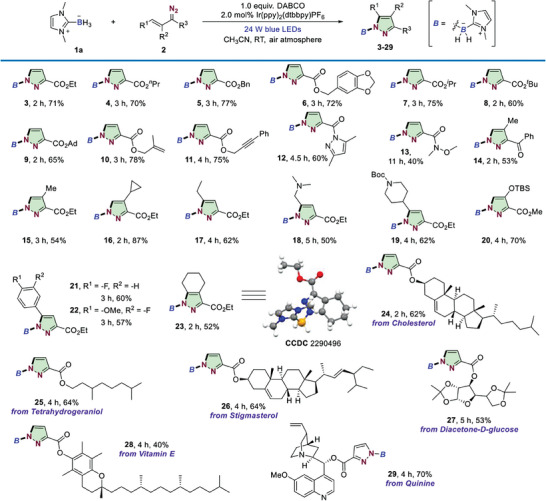
Scope of vinyldiazo compounds. ^a^ **1** (0.3 mmol), **2** (0.9 mmol), DABCO (0.3 mmol), and Ir(ppy)_2_(dtbbpy)PF_6_ (2.0 mol%) in MeCN (1.0 mL), with 24 W blue LEDs irradiation at rt under air atmosphere. ^b^ isolated yield.

Having finalized the optimal reaction conditions, the scope of this photocatalytic boron‐handled pyrazole formation protocol was explored (**Scheme** **2**). The reaction was quite general for a wide range of vinyldiazo compounds bearing various ester moieties, including primary alkyl (**3‐6**), secondary alkyl (**7**), tertiary alkyl (**8**, **9**) and unsaturated alkene and alkyne functional groups (**10**, **11**), thus giving the desired pyrazoles in 65–78% yields. To our delight, the reaction was not limited to diazoesters; the process was also effective using an acylpyrazole‐, amide or ketone‐based vinyldiazo species (**12–14**). Next, we investigated the reactivity of *β –* or *γ*‐substituted vinyldiazo esters. It was found that diazoacetate with *β*‐methyl‐ and cyclopropyl‐substitution could also participate in the cascade radical cyclization, affording the target heterocyclic products in 54% and 87%, respectively (**15**,**16**). Incorporation of different alkyl (**17–19**), silyl ether (**20**) and aryl groups (**21**, **22**) at□*γ*‐position of vinyldiazo esters also proved to be successful. Note that, the method could be further extended to the synthesis of fully substituted pyrazole by using **23** as an example. More complex bioactive molecules, as well as drug derivatives were examined with no influence on this pyrazole formation reaction, including naturally occurring alcohols such as *cholesterol* (**24**), *tetrahydrogeraniol* (**25**), *stigmasterol* (**26**) and a protected glucose derivative (**27**) as well as derivatives of drugs such as *vitamin E* (**28**), and *quinine* (**29**).

**Scheme 3:**
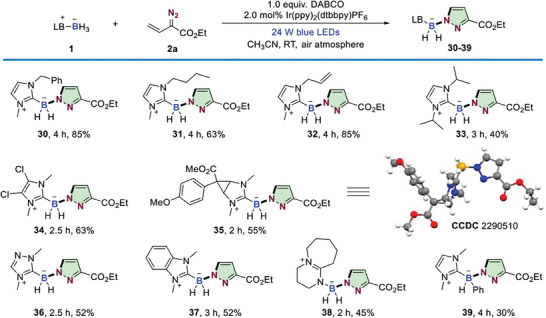
Scope of LB‐BH_3_. ^a^ **1** (0.3 mmol), **2** (0.9 mmol), DABCO (0.3 mmol), and Ir(ppy)_2_(dtbbpy)PF_6_ (2.0 mol%) in MeCN (1.0 mL), with 24 W blue LEDs irradiation at rt under air atmosphere. ^b^ isolated yield.

Next, we tested the reactivity of other types of LBRs through different commonly used Lewis base boranes. As shown in **Scheme** **3**, either structure modification of the *N,N*‐alkyl substituents or incorporation of two chlorine atoms on the imidazole ring of NHC–BH_3_ complexes proved to be compatible with the present reaction conditions (**31–34**). Note that, reduced efficiency was observed by the introduction of two isopropyl groups which should be attributed to the steric effect. We also studied the reaction of NHC–BH_3_ with cyclopropanation of the imidazolium backbone,^[^ ^19^ ^]^ yielding product **35** with 55% yield. Importantly, the current method allowed the construction of different kind of Lewis base borane‐modified pyrazoles, such as products derived from triazole borane (**36**), benzimidazole borane (**37**), and DBU‐borane (**38**). Adding a phenyl group on boron site still could give the target heterocycle, albeit with relatively low yield (**39**).

### 2.3 Mechanism Studies

**Scheme 4:**
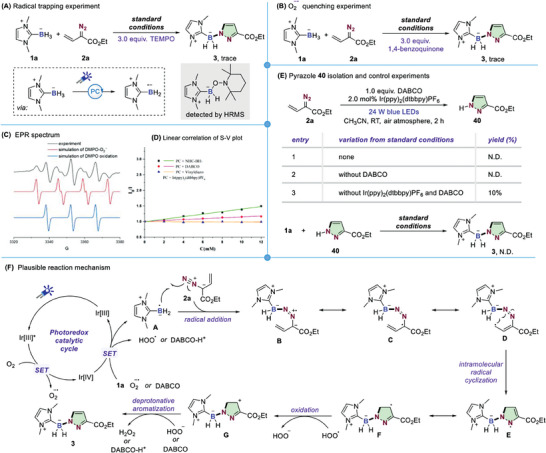
Mechanism studies and the proposed reaction mechanism. A) Radical trapping experiment with TEMPO. B) Superoxide radical anion quenching experiment with 1,4‐benzoquinone. C) Probing involvement of O_2_^⚫^¯ species by EPR studies. D) Stern−Volmer experiments with photoexcited Ir(ppy)_2_(dtbbpy)PF_6_ in CH_3_CN. I_0_ and I are the respective luminescence intensities in the absence and presence of the indicated concentrations of the corresponding quencher. E) Pyrazole **40** isolation and control experiments. F) Plausible reaction mechanism.

To elucidate the mechanism of this photocatalytic B─N bond formation process, some mechanistic investigations were conducted in **Scheme** **4**. It was found that the reaction could be intercepted by a radical capturing experiment with the addition stoichiometric amount of TEMPO. The adduct of TEMPO and NHC–boryl radical could be detected by HRMS (Scheme 4A). The result indicated that the reaction should proceed through an NHC–boryl radical‐involved radical process. As we know, the 1,4‐benzoquinone is a well‐known superoxide radical anion (O_2_^⚫−^) quencher. The reaction failed to produce **3** when 3.0 equivalents of 1,4‐benzoquinone was added, implying that O_2_^⚫−^ was generated during in the reaction (Scheme 4B).^[^ ^20^ ^]^ Additional evidences supporting the intermediacies of O_2_^⚫−^ species was confirmed by the electron paramagnetic resonance (EPR) studies with 5,5‐dimethyl‐pyrroline N‐oxide (DMPO) as a free radical spin‐trapping agent (Scheme 4C). Next, we conducted the Stern–Volmer luminescence quenching experiments to identify which reaction component quenched the excited iridium‐based photocatalyst (Scheme 4D). It was observed that NHC–BH_3_ and DABCO could slightly quench the excited photoredox catalyst [k_q_ (NHC‐BH_3_) = 7.3 × 10^7^ M^−1^ S^−1^; k_q_ (DABCO) = 2.5 × 10^7^ M^−1^ S^−1^]. Vinyldiazo compound **2a** did not show an obvious quenching effect. The low quenching efficiency, together with the mismatched redox potentials revealed that reductive quenching of Ir(III)* [*E*_1/2_^red^ (III*/II) = +0.66 V vs.SCE)]^[^ ^21^ ^]^ by NHC–BH_3_ (*E*_1/2_^ox^ = + 0.76 V vs. SCE)^[^ ^22^ ^]^ should not be the initiation step. Instead, single electron oxidation of Ir(III)* by oxygen to form Ir(IV) species was thermodynamically favorable {[*E*_1/2_^ox^ (III*/IV) = −0.96 V vs. SCE); *E*_1/2_^red^ (O_2_/O_2_^⚫−^) = −0.75 V vs. SCE]}.^[^ ^23^ ^]^ The key NHC–BH_2_^⚫^ would then generate through a single electron oxidation of NHC–BH_3_ by Ir(IV), followed by deprotonation.

To gain more mechanistic understanding on the B─N bond formation step, some control experiments were performed in Scheme 4E. It is well known that vinyldiazo compound could transfer to pyrazole **40** through 1,5‐electrocyclization.^[^ ^7^, ^18^ ^]^ When vinyldiazo compound **2a** was irradiated with blue LED for 2 h under the best reaction conditions with or without the addition of DABCO, no **40** was detected (Scheme 4D, entry 1 and entry 2). Only 10% of **40** was isolated when **2a** was irradiated under sole blue LED irradiation without any photoredox catalyst and base (Scheme 4D, entry 3). In addition, **3** was not observed by reacting of **40** with NHC–BH_3_ under standard reaction conditions. These results suggested that **40** should not be the intermediate for the formation of the final boron‐handled pyrazoles.

Base on the above mechanistic investigations, a plausible reaction mechanism was proposed in Scheme 4G. After photoexcitation of Ir(III) photoreodx catalyst, the formed Ir(III)* [*E*_1/2_^ox^ (III*/IV) = − 0.96 V vs. SCE)]^[^ ^21^ ^]^ was quenched by O_2_ [*E*_1/2_^red^ (O_2_/O_2_^⚫−^) = −0.75 V vs. SCE] to generate Ir(IV) and superoxide radical anion. Then, single electron oxidation of NHC–BH_3_ (*E*_1/2_^ox^ = + 0.76 V vs. SCE) was electronically matched oxidative state Ir(IV) [*E*_1/2_^red^ (IV/III) = + 1.21 V vs. SCE)] to close the photoredox catalytic cycle and generate the NHC–boryl radical cation, subsequently deprotonation to give NHC–boryl radical **A**. Selective radical addition of **A** to the N‐atom of vinyldiazoacetate **2a** afforded intermediate **B** which resonated to terminal radical species **D**. Then, the intramolecular radical cyclization with C–N bond formation process took place to afford intermediate **F**. Rapid oxidation of **F** provided the cation intermediate **G**, which subsequently underwent deprotonation process to produce the final product **3**.

### 2.4 DFT Calculation

**Figure 1:**
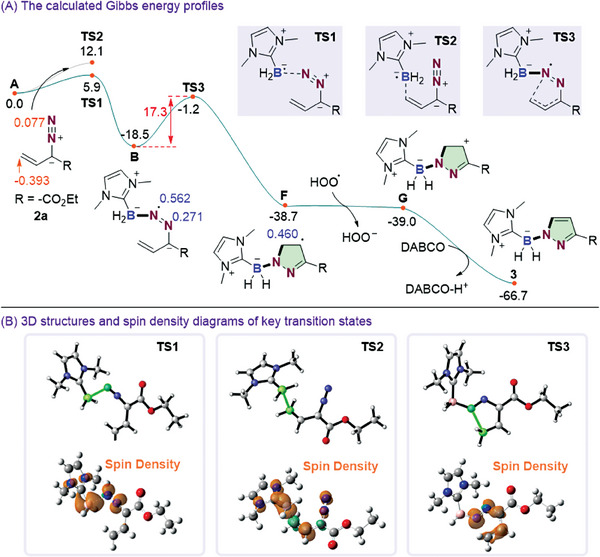
A) The calculated Gibbs energy profiles for the reaction mechanism (in kcal mol^−1^), spin density in blue and NBO charge in orange; B) 3D structures and spin density diagrams of key transition states.

Density functional theory (DFT) calculations were also performed to provide further understanding of the reaction mechanism. As shown in **Figure** **1**, the reaction starts with the radical addition process. NBO charge analysis indicates that the terminal carbon atom of the C═C bond is more negative charged compared to the terminal N atom, rendering the high reactivity of N atom with the nucleophilicity boryl radical,^[^ ^11^ ^]^ in accordance with the calculated favorable B─N bond formation process via transition state **TS1**. A lower Gibbs free energy barrier of 5.9 kcal mol^−1^ is needed for this process to supply the stable intermediate **B**, which is an exothermic process of −18.5 kcal mol^−1^. Then, the intramolecular radical addition with C─N bond formation process takes place via **TS3** to afford the more stable intermediate **F**. The reaction barrier for this step is 17.3 kcal mol^−1^, which is the rate‐determining transition state. From **F**, the barrierless oxidation and deprotonation process take place to produce the final product **3**. In summary, the rate‐determining step for this reaction is the intramolecular radical cyclization process, with a Gibbs free energy barrier of 17.3 kcal mol^−1^, in accordance with the current reaction conditions.

### 2.5 Synthetic Applications

**Scheme 5:**
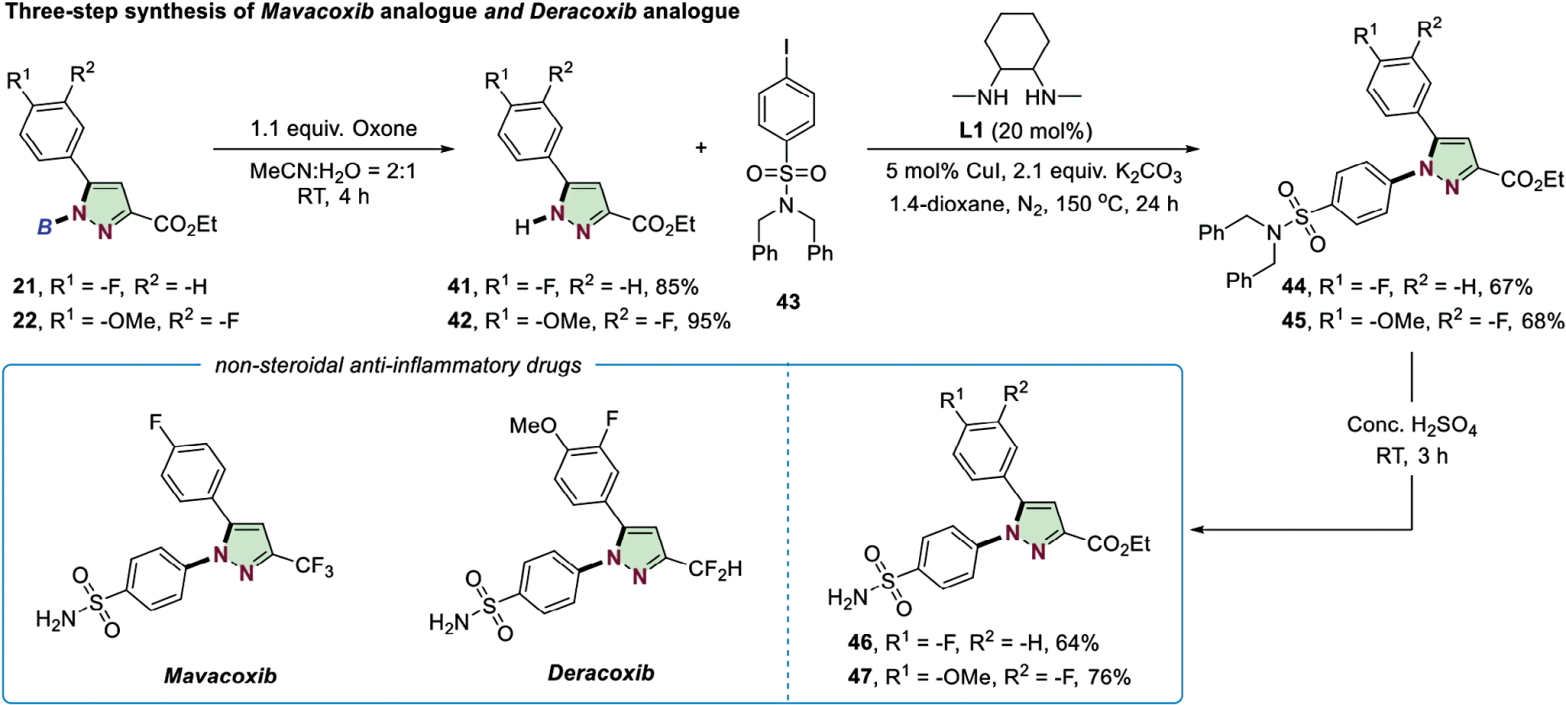
Follow‐up chemistry.

The synthetic value of the obtained pyrazoles was further showcased by a three‐step preparation of nonsteroidal anti‐inflammatory drugs *Mavacoxib* and *Deracoxib* analogues (**Scheme** **5**).^[^ ^16^, ^24^ ^]^ Deboration of the formed aryl substituted pyrazole **21** and **22** by Oxone provided **41** and **42** in excellent yields. Then, copper‐mediated Ullman couplings, followed by acid‐promoted deprotection afforded the *Mavacoxib* analogue **46** and *Deracoxib* analogue **47**.

### 2.6 Evaluation of the In Vitro Antitumor Activities of Boron‐Handled Pyrazoles

**Figure 2:**
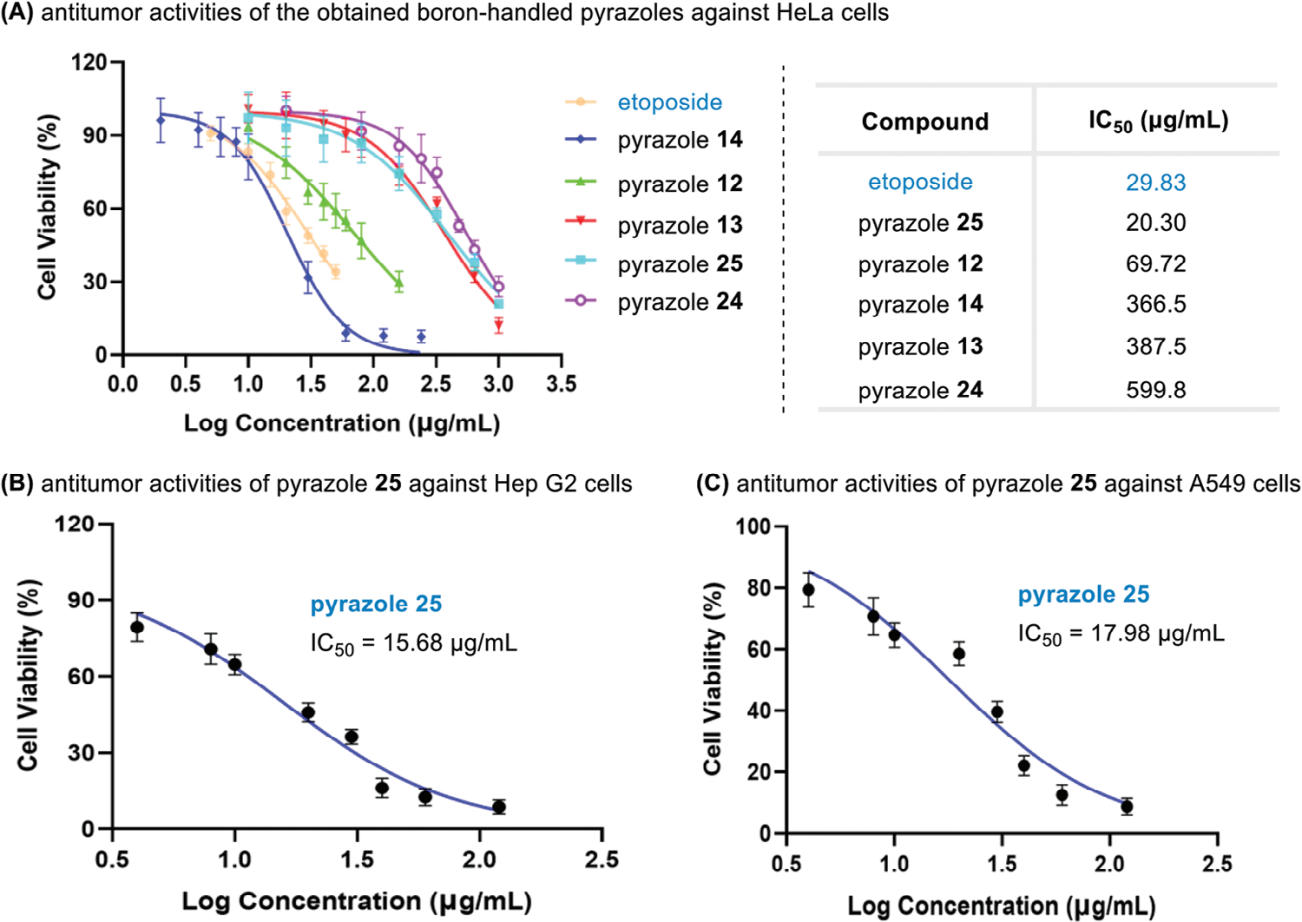
The antiproliferative activity of the obtained boron‐handled pyrazoles in cancer cell lines.

Considering the potential bioactivity of the formed boron‐handled pyrazoles,^[^ ^8^, ^16^ ^]^ we investigated the antitumor activities of those obtained compounds against HeLa cells (human cervical cancer cells), a typical human cervical cancer cell line. Here, IC_50_ (the half maximal inhibitory concentration) was selected to evaluate the antitumor activity. As shown in **Figure** **2**, the obtained pyrazole **25** had excellent antitumor ability with IC_50_ = 20.3 µg mL^−1^, better than the performance of antitumor drug etoposide. In order to verify the universality of its antitumor ability, we further chose two other cell lines, Hep G2 (human liver cancer) and A549 (human lung cancer) cells (Figure 2), and the results showed that IC_50_ values were 15.68 and 17.98 µg mL^−1^ for Hep G2 and A549 cells, respectively. indicating the excellent antitumor activity of compound **25**. In contrast, *IC*_50_ values of compound **25** for HL‐7702 (normal hepatic) cells was 36.62 µg mL^−1^, approximately two folds for cancer cells (Figure S2, Supporting Information), suggesting the selectivity towards cancerous cells.

## 3 Conclusion

In summary, we have developed a photoredox‐mediated cascade radical cyclization of LBRs with vinyldiazo compounds. The reaction started with the addition of LBRs at diazo site, followed by intramolecular radical cyclization to access a wide range of boron‐handled pyrazoles in good to excellent yields. This was a significant advancement comparing to previous report where photochemical transformation of vinyldiazo compounds mainly occurred at C═C bond site, where diazo moiety leaved as nitrogen gas. The detailed mechanism studies and computational studies were performed to elucidate the reaction mechanism and to rationalize the observed reaction outcome. The scale‐up reaction, and the discovery of hit compounds for potential antitumor drugs further rendered the method attractive and valuable.

## 4 Experimental Section

### General Procedure

To a 10 mL Schlenk flask equipped with a magnetic stir bar was added **1a** (33.0 mg, 0.3 mmol, 1.0 equiv.), **2a** (126.0 mg, 0.9 mmol, 3.0 equiv.), Ir(ppy)_2_(dtbbpy)PF_6_ (5.4 mg, 0.006 mmol, 0.02 equiv.), dry CH_3_CN (1.0 mL), under an air atmosphere. After that, the solution was stirred at a distance of ≈3 cm from a 24 W blue LED at room temperature for 2 h. The solvent was removed by vacuum and the crude product was purified by flash chromatography on silica gel silica: 200–300 using ethyl acetate as eluant to provide pure boronated product **3** as a white solid in 71% yield (52.8 mg). ^1^H NMR (400 MHz, CDCl_3_, 300 K): δ (ppm) = 7.50 (d, *J* = 2.1 Hz, 1H), 6.84 (s, 2H), 6.71 (d, *J* = 2.1 Hz, 1H), 4.31 (q, *J* = 7.1 Hz, 2H), 3.63 (s, 6H), 1.33 (t, *J* = 7.1 Hz, 3H). ^13^C NMR (100 MHz, CDCl_3_, 300 K): δ (ppm) = 163.6, 144.4, 136.6, 121.1, 107.8, 60.1, 35.9, 14.4. ^11^B NMR (128.4 MHz, CDCl_3_, 300 K): δ (ppm) = −19.05 (t, *J* = 96.4 Hz). HRMS (ESI) m/z: [M+H]^+^ Calcd for C_11_H_18_BN_4_O_2_^+^: 249.1517; Found: 249.1517.

[CCDC 2 290 496 and 2 290 510 contain the supplementary crystallographic data for this paper. These data can be obtained free of charge from The Cambridge Crystallographic Data Centre via www.ccdc.cam.ac.uk/data_request/cif.]

## Conflict of Interest

The authors declare no conflict of interest.

## Supporting information

Supporting Information

## Data Availability

The data that support the findings of this study are available from the corresponding author upon reasonable request.
