# Supplementary material for: Photocatalytic Boryl Radicals Triggered Sequential B─N/C─N Bond Formation to Assemble Boron‐Handled Pyrazoles
Source: Adv Sci (Weinh). 2023 Nov 29;11(3):2306728. doi: 10.1002/advs.202306728 (PMC10797447; doi:10.1002/advs.202306728)

ADVANCED SCIENCE
Open Access

Supporting Information

for *Adv. Sci.*, DOI 10.1002/advs.202306728

Photocatalytic Boryl Radicals Triggered Sequential B—N/C—N Bond Formation to Assemble Boron-Handled Pyrazoles

Yang Xie, Ruilong Zhang, Ze-Le Chen, Mengtao Rong, Hui He, Shaofei Ni, Xiang-Kui He, Wen-Jing Xiao* and Jun Xuan**

Photocatalytic Boryl Radicals Triggered Sequential B-N/C-N Bond Formation to Assemble Boron-Handled Pyrazoles

Yang Xie,[1†] Rui-Long Zhang,[1†] Ze-Le Chen,[1†] Meng-Tao Rong,[1] Hui He,[2] Shao-Fei Ni,*[2] Wen-Jing Xiao*[3] and Jun Xuan*[1]

[1] Anhui Province Key Laboratory of Chemistry for Inorganic/Organic Hybrid Functionalized Materials, College of Chemistry & Chemical Engineering, Anhui University, Hefei, Anhui 230601, China.

[2] Department of Chemistry and Key Laboratory for Preparation and Application of Ordered Structural Materials of Guangdong Province, Shantou University, Shantou, Guangdong 515063

[3] Key Laboratory of Pesticide and Chemical Biology, Ministry of Education, College of Chemistry, Central China Normal University, Wuhan, Hubei 430079.

[†] These authors contributed equally

Table of contents

1. General.. S2
2. Preparation and Spectral Data of Starting Materials………………………... S3
3. The Condition Optimization and General Procedure………………………. S8
4. Synthetic applications and Gram-scale Synthesis…………….………….. S10
5. Mechanistic Studies…………………………..………………………….. S14
6. Spectral Data of Products…………………….…..……...…………….. S20
7. Crystal data……………………………………………………………. S34
8. Schemes of computed reaction pathways…………………………………... S38
9. References……………………………………………………………... S47
10. Copies of ^{1}H NMR, ^{13}C NMR and ^{11}B NMR ^{19}F NMR Spectra………...… S48

1 General

All reactions involving air- or moisture-sensitive reagents or intermediates were carried out in pre-heated glassware under an argon atmosphere using standard Schlenk techniques. All other solvents and reagents were purified according to standard procedures or were used as received from chemical suppliers. The starting materials were synthesized according to literature procedures. The light employed in this work was bought from GeAo Chemical: model H106062, 24 W blue LEDs, λ = 450 ~ 460 nm. All photo-reactions were performed in borosilicate glass irradiation vessel at a distance of ~3 cm from light source. All reactions involving heating are carried out in an oil bath.

Chromatography: Analytical thin layer chromatography was performed using Qingdao Puke Parting Materials Co. silica gel plates (Silica gel 60 F254). Visualisation was by ultraviolet fluorescence (λ = 254 nm) and/or staining with phosphomolybdic acid or potassium permanganate(KMnO4). Flash column chromatography was performed using 200-300 mesh silica gel.

^{1}H NMR, **^{13}C NMR**, **^{11}B NMR and ^{19}F NMR** spectra were recorded on a JEOL JNM ECZ400R and ECZ600R at 300 K. Spectra were calibrated relative to solvent's residual proton and carbon chemical shift: CHCl3 (δ = 7.26 for ^{1}H NMR and δ = 77.0 for ^{13}C NMR), d_6-DMSO (δ = 2.50 for ^{1}H NMR and δ = 39.5 for ^{13}C NMR). Data are reported as follows: chemical shift δ/ppm, integration (^{1}H only), multiplicity (s = singlet, d = doublet, t = triplet, q = quartet, dd= doublet of doublets, m = multiplet or combinations thereof; ^{13}C signals are singlets unless otherwise stated), coupling constants *J* in Hz, assignment.

High Resolution Mass Spectrometry (HRMS): All were recorded on Thermo Fisher Scientific LTQ Orbitrap XL using an atmospheric-pressure chemical ionization (APCI^{+}) or positive electrospray ionization (ESI^{+}). Measured values are reported to 4 decimal places of the calculated value. The calculated values are based on the most abundant isotope.

X-ray Crystallography were collected at 100 K on a Rigaku Oxford Diffraction Supernova Dual Source, Cu at Zero equipped with an AtlasS2 CCD using Cu Kα radiation. The data were collectedand processed using CrysAlisPro.

EPR spectra were recorded on Bruker Magnettech ESR5000.

Computational studies Computational details: All calculations were performed using Gaussian 16, Revision A.03 package. [1] All of the reactants, intermediates, transition states, products were optimized by the DFT with the M06-2X functional. [2] For geometry optimizations and frequency calculations, BS-I basis set system was employed. In BS-I, we employed 6-311G(d) basis sets for C, H, O, N and B. All the stationary structures were characterized with no imaginary frequency and the transition state structures (TSs) were characterized with a single imaginary frequency. Intrinsic reaction coordinate (IRC) calculations were performed on the TSs. The solvent effect of Acetonitrile was evaluated through the SMD method, [3] in which a better basis system BS-II was used. In BSII, we employed 6-311++G(d, p) basis sets for all atoms. All reported energies are free energies at a concentration of 1 M and a temperature of 298.15 K.

2 Preparation and Spectral Data of Starting Materials

The Vinyl diazoacetates **2** are prepared according to the known procedures.[4-14]

The boranes compounds **1** are prepared by the literature reports.[15-20]

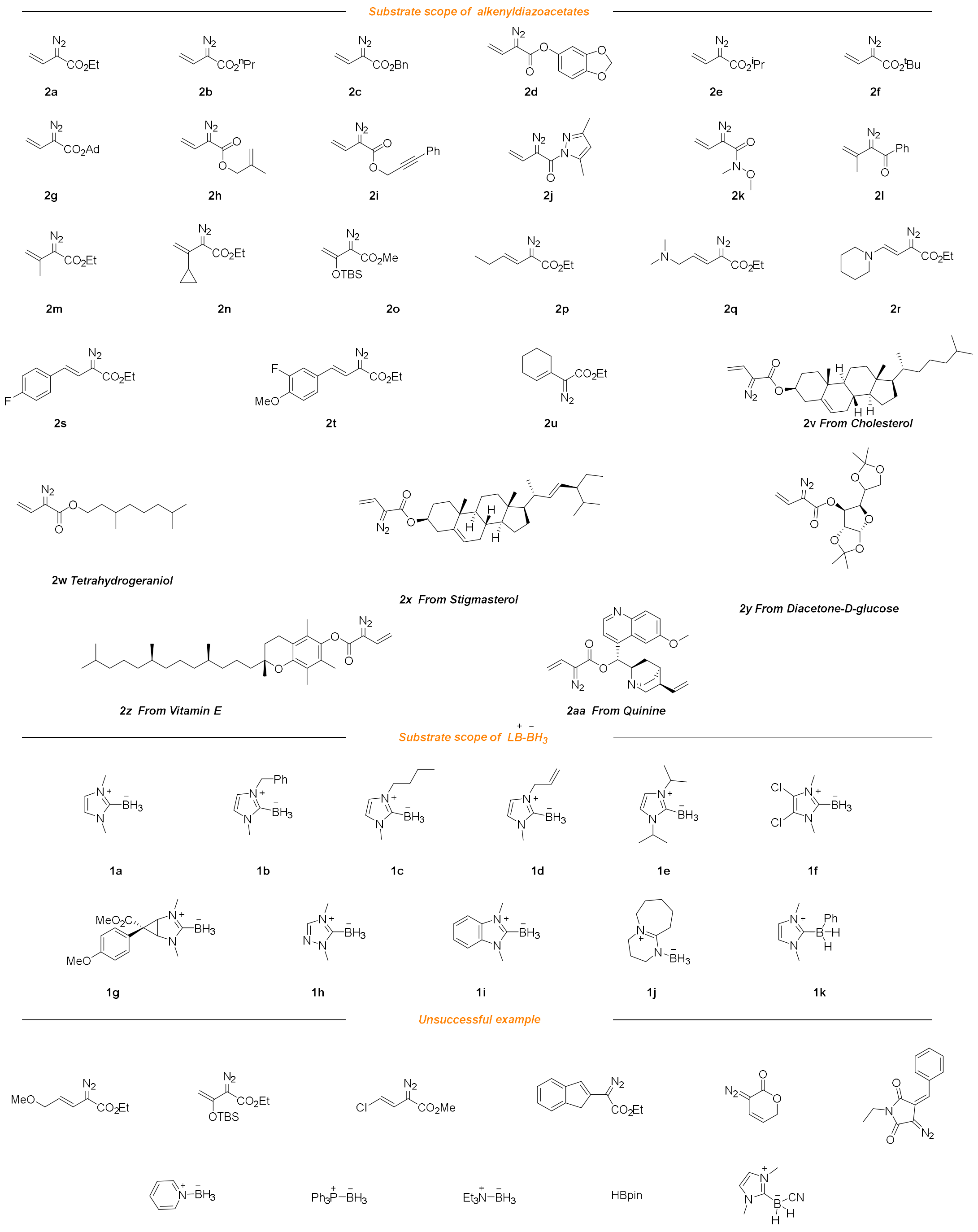

Method[2] : Preparation of vinyl diazoacetates 2b, 2d, 2h, 2i, 2k, 2q, 2t, 2w, 2x, 2aa.

Alcohol (10 mmol) and 2,2,6-trimethyl-1,3-dioxene-4-one (12 mmol, 1.2 equiv) were dissolved in xylene (2.0 M) and refluxed at 140 °C under argon for 2 h. The solvent was removed from the reaction by vacuum leaving a black oil. The crude mixture was purified by column chromatography (PE/EA = 10:1) to give acetoacetate (**S-1**). Then, acetoacetate was dissolved in MeCN (0.4 M) and cooled to 0 °C. *p*-Acetamidobenzenesulfonyl azide (*p*-ABSA, 1.1 equiv), followed by triethylamine (1.5 equiv) were added and the reaction warmed to rt for 2 h. The pale yellow solid precipitate was filtered and the residue concentrated and purified by column chromatography (PE/EA = 10:1) to give 2-diazo-3-oxobutanoate (**S-2)**.

The solution of 2-diazo-3-oxobutanoate **(S-2)** in MeOH (0.6 M) at 0 °C was slowly added $NaBH_4$ (1.5 equiv). The resulting solution was warmed to room temperature and stirred for 1 h. Then the MeOH was evaporated and the residue was diluted with water and extracted with ethyl acetate and dried over anhydrous Na_2SO_4. After the solvent was evaporated, the crude product was purified by column chromatography (PE/EA = 5:1) to give 2-diazo-3-hydroxybutanoate (**S-3**) as a yellow oil. To a solution of 2-diazo-3-hydroxybutanoate and Et_3N (4.0 equiv) in CH_2Cl_2 (0.33 M) at 0 °C was slowly added a solution of $POCl_3$ (1.5 equiv) in CH_2Cl_2 (1.0 M) over 20 minutes. The resulting solution was warmed to room temperature and stirred for 2 h. The solution was washed with water and dried over anhydrous $NaSO_4$. The crude product was purified by flash chromatography (PE/EA = 50:1) to afford vinyldiazoacetate (**S-4**).

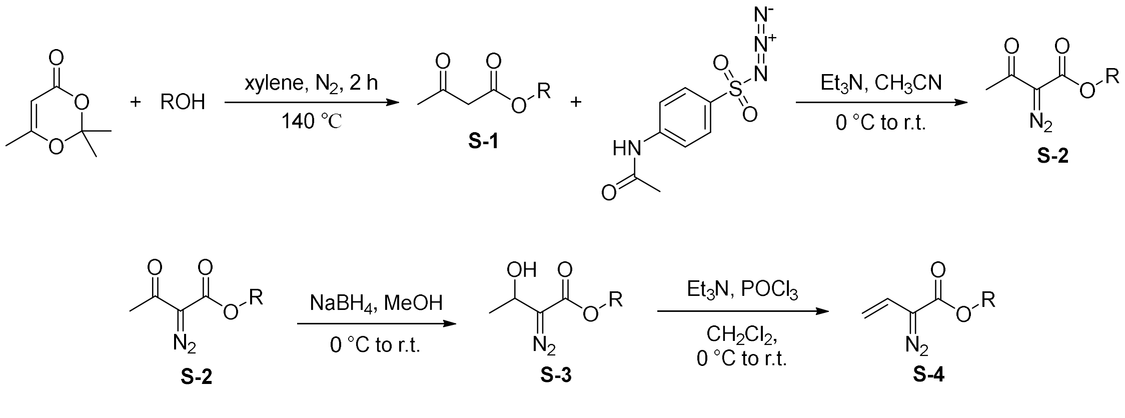

Propyl 2-diazobut-3-enoate (2b)

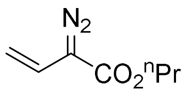

Red oil. **^{1}H NMR** (400 MHz, $CDCl_3$, 300 K): δ (ppm) = 6.15 (dd, *J* = 17.4, 11.0 Hz, 1H), 5.09 (d, *J* = 10.9 Hz, 1H), 4.84 (d, *J* = 17.4 Hz, 1H), 4.15 (t, *J* = 6.7 Hz, 2H), 1.67 (q, *J* = 7.1 Hz, 2H), 0.93 (t, *J* = 7.5 Hz, 3H). **^{13}C NMR** (100 MHz, $CDCl_3$, 300 K): δ (ppm) = 164.9, 120.5, 107.3, 66.6, 22.1, 10.2. **HRMS** (ESI) m/z: $[M+H]^+$ Calcd for $C_7H_{11}N_2O_2^+$: 155.0815; Found: 155.0814.

Benzo[d][1,3]dioxol-5-yl 2-diazobut-3-enoate (2d)

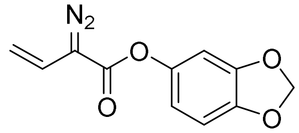

Red oil. **^{1}H NMR** (400 MHz, $CDCl_3$, 300 K): δ (ppm) = δ 6.86 – 6.82 (m, 2H), 6.80 – 6.76 (m, 1H), 6.21 – 6.12 (m, 1H), 5.96 (s, 2H), 5.14 (s, 2H), 5.11 (d, *J* = 11.0 Hz, 1H), 4.85 (d, *J* = 17.4 Hz, 1H). **^{13}C NMR** (100 MHz, $CDCl_3$, 300 K): δ (ppm) = 164.7, 147.8, 147.7, 129.5, 122.3, 120.3, 109.0, 108.2, 107.5, 101.2, 66.7. **HRMS** (APCI) m/z: $[M+H]^+$ Calcd for $C_{12}H_{11}N_2O_4^+$:247.0713; Found: 247.0722.

2-methylallyl 2-diazobut-3-enoate (2h)

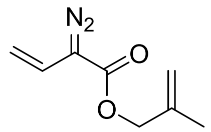

Red oil. **^{1}H NMR** (400 MHz, $CDCl_3$, 300 K): δ (ppm) = 6.24 – 6.12 (m, 1H), 5.12 (d, *J* = 11.0 Hz, 1H), 4.96 (d, *J* = 13.8 Hz, 2H), 4.87 (d, *J* = 17.4 Hz, 1H), 4.63 (s, 2H), 1.76 (s, 3H). **^{13}C NMR** (100 MHz, $CDCl_3$, 300 K): δ (ppm) = 164.5, 139.7, 120.3, 113.0, 107.6, 68.1, 68.0, 19.3. **HRMS** (APCI) m/z: $[M+H]^+$ Calcd for $C_8H_{11}N_2O_2^+$:167.0815; Found: 167.0815.

3-phenylprop-2-yn-1-yl 2-diazobut-3-enoate (2i)

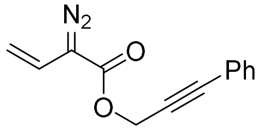

Red oil. **^{1}H NMR** (400 MHz, $CDCl_3$, 300 K): δ (ppm) = 7.48 – 7.44 (m, 2H), 7.35 – 7.31 (m, 3H), 6.24 – 6.15 (m, 1H), 5.15 (d, *J* = 11.0 Hz, 1H), 5.05 (s, 2H), 4.89 (d, *J* = 17.4 Hz, 1H). **^{13}C NMR** (100 MHz, $CDCl_3$, 300 K): δ (ppm) = 164.5, 139.7, 120.3, 113.0, 107.6, 68.1, 68.0, 19.3. **HRMS** (APCI) m/z: $[M+H]^+$ Calcd for $C_8H_{11}N_2O_2^+$:167.0815; Found: 167.0815.

2-diazo-N-methoxy-N-methylbut-3-enamide (2k)

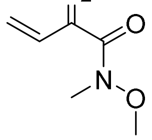

Red oil. **^{1}H NMR** (400 MHz, $CDCl_3$, 300 K): δ (ppm) = 6.44 (dd, *J* = 17.2, 10.7 Hz, 1H), 5.09 (d, *J* = 10.7 Hz, 1H), 4.64 (d, *J* = 17.2 Hz, 1H), 3.65 (s, 3H), 3.20 (s, 3H). **^{13}C NMR** (100 MHz, $CDCl_3$, 300 K): δ (ppm) = 165.1, 122.5, 105.9, 61.3, 34.1. **HRMS** (ESI) m/z: $[M+H]^+$ Calcd for $C_6H_{10}N_3O_2^+$: 156.0768; Found: 156.0775.

Ethyl (E)-2-diazo-5-(dimethylamino)pent-3-enoate (2q)

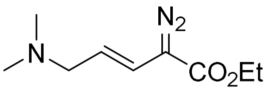

Red oil. **^{1}H NMR** (400 MHz, $CDCl_3$, 300 K): δ (ppm) = 5.92 (d, *J* = 16.7 Hz, 1H), 5.38 – 5.27 (m, 1H), 4.25 (q, *J* = 7.1 Hz, 2H), 3.00 (d, *J* = 6.9 Hz, 2H), 2.21 (s, 6H), 1.27 (t, *J* = 7.1 Hz, 3H). **^{13}C NMR** (100 MHz, $CDCl_3$, 300 K): δ (ppm) = 165.1, 121.3, 115.7, 61.5, 61.1, 45.0, 14.4. **HRMS** (ESI) m/z: $[M+H]^+$ Calcd for $C_9H_{16}N_3O_2^+$: 198.1237; Found: 198.1243.

Ethyl (E)-2-diazo-4-(3-fluoro-4-methoxyphenyl)but-3-enoate (2t)

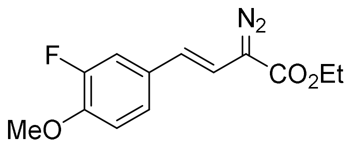

Red oil. **^{1}H NMR** (400 MHz, $CDCl_3$, 300 K): δ (ppm) = δ(m, 1H), 7.02 (d, *J* = 8.5 Hz, 1H), 6.89 (t, *J* = 8.6 Hz, 1H), 6.31 (d, *J* = 16.2 Hz, 1H), 6.09 (d, *J* = 16.3 Hz, 1H), 4.31 (q, *J* = 7.2 Hz, 2H), 3.87 (s, 3H), 1.32 (t, *J* = 7.1 Hz, 3H). **^{13}C NMR** (100 MHz, $CDCl_3$, 300 K): δ (ppm) = 165.1, 152.6 (d, *J* = 245.5 Hz), 146.7 (d, *J* = 11.0 Hz), 130.5 (d, *J* = 6.7 Hz), 122.0 (d, *J* = 3.3 Hz), 121.6, 113.4 (d, *J* = 2.3 Hz), 112.9 (d, *J* = 19.1 Hz), 110.1, 61.4, 56.3, 16.3. **HRMS** (APCI) m/z: $[M+H]^+$ Calcd for $C_{13}H_{14}FN_2O_3^+$: 265.0983; Found: 265.0984.

3,7-dimethyloctyl 2-diazobut-3-enoate (2w)

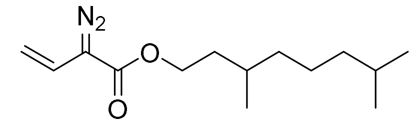

Red oil. **^{1}H NMR** (400 MHz, $CDCl_3$, 300 K): δ (ppm) = 6.21 – 6.10 (m, 1H), 5.10 (d, *J* = 11.0 Hz, 1H), 4.85 (d, *J* = 17.4 Hz, 1H), 4.28 – 4.21 (m, 2H), 1.73 – 1.64 (m, 1H), 1.57 – 1.42 (m, 3H), 1.32 – 1.22 (m, 3H), 1.17 – 1.09 (m, 3H), 0.90 (d, *J* = 6.5 Hz, 3H), 0.87 (s, 3H), 0.85 (s, 3H). **^{13}C NMR** (100 MHz, $CDCl_3$, 300 K): δ (ppm) = δ 165.1, 120.6, 107.4, 77.5, 77.1, 76.8, 63.9, 39.3, 37.2, 35.7, 29.9, 28.0, 24.7, 22.7, 19.7. **HRMS** (APCI) m/z: $[M+H]^+$ Calcd for $C_{14}H_{25}N_2O_2^+$: 253.1911; Found: 253.1915.

(3S,8S,9S,10R,13R,14S,17R)-17-((2R,5S,E)-5-ethyl-6-methylhept-3-en-2-yl)-10,13-dimethyl-2,3,4,7,8,9,10,11,12,13,14,15,16,17-tetradecahydro-1H-cyclopenta[a]phenanthren-3-yl 2-diazobut-3-enoate (2x)

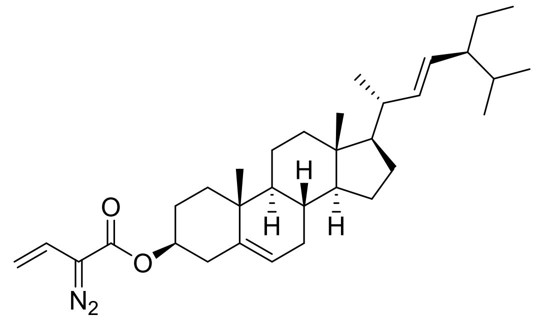

Red solid. **^{1}H NMR** (400 MHz, $CDCl_3$, 300 K): δ (ppm) = 6.22 – 6.12 (m, 1H), 5.41 – 5.36 (m, 1H), 5.19 – 5.08 (m, 2H), 5.06 – 4.97 (m, 1H), 4.84 (d, *J* = 17.4 Hz, 1H), 4.78 – 4.68 (m, 1H), 2.42 – 2.29(m, 2H), 2.10 – 1.94 (m, 3H), 1.93 – 1.82 (m, 2H), 1.74 – 1.66 (m, 1H), 1.61 – 1.40 (m, 9H),

1.21 – 1.11 (m, 4H), 1.07 – 0.97 (m, 9H), 0.88 – 0.77 (m, 10H), 0.70 (s, 3H). **^{13}C NMR** (100 MHz, $CDCl_3$, 300 K): δ (ppm) = 138.3, 129.3, 122.9, 120.7, 107.2, 77.3, 77.0, 76.7, 74.9, 56.8, 55.9, 51.2, 50.0, 42.2, 40.5, 39.6, 38.3, 36.9, 36.6, 31.9, 28.9, 28.0, 25.4, 24.3, 21.2, 21.1, 21.0, 19.3, 19.0, 12.2, 12.0. **HRMS** (APCI) m/z: $[M+H]^+$ Calcd for $C_{33}H_{51}N_2O_2^+$: 507.3945; Found: 507.3941.

(R)-(6-methoxyquinolin-4-yl)((1S,2R,4S,5R)-5-vinylquinuclidin-2-yl)methyl 2-diazobut-3-enoate (2aa)

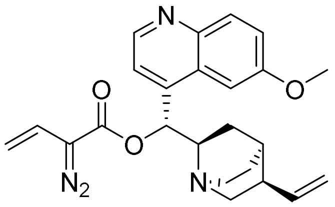

Red oil. **^{1}H NMR** (400 MHz, $CDCl_3$, 300 K): δ (ppm) = 8.72 (d, J = 3.2 Hz, 1H), 8.00 (d, J = 9.1 Hz, 1H), 7.39 (s, 1H), 7.37 - 7.30 (m, 2H), 6.58 (d, J = 6.9 Hz, 1H), 6.16 – 6.02 (m, 1H), 5.84 – 5.74 (m, 1H), 5.11 (d, J = 11.0 Hz, 1H), 5.02 – 4.93 (m, 2H), 4.87 (d, J = 17.4 Hz, 1H), 3.92 (s, 3H), 3.40 (q, J = 7.7 Hz, 1H), 3.11 (s, 1H), 3.06 – 2.97 (m, 1H), 2.71 – 2.55 (m, 2H), 2.25 (s, 1H), 1.86 (d, J = 18.3 Hz, 2H), 1.71 – 1.61 (m, 1H), 1.58 – 1.47 (m, 2H). **^{13}C NMR** (100 MHz, $CDCl_3$, 300 K): δ (ppm) = 163.7, 157.8, 147.3, 144.7, 141.4, 131.7, 126.7, 121.8, 119.6, 118.8, 114.4, 108.2, 101.1, 77.3, 77.0, 76.7, 74.5, 59.0, 56.4, 55.5, 42.3, 39.4, 27.6, 27.3, 24.1. **HRMS** (ESI) m/z: $[M+H]^+$ Calcd for $C_{24}H_{27}N_4O_3^+$: 419.2078; Found: 419.2078.

3 The Condition Optimization[a]

Table S1. The Condition Optimization of the Reaction Conditions

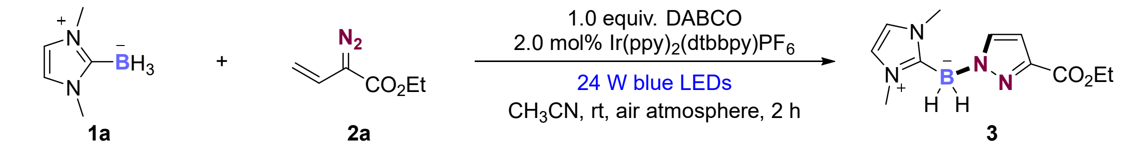

Entry	Photocatalyst (2 mol%)	Solvent	Molar of 1a	Molar of 2a	Volume	Yield[b]
1	$Ir(ppy)_2(dtbbpy)PF_6$	MeCN	0.1 mmol	0.3 mmol	1 mL	50%
2	Rhodamine 6G	MeCN	0.1 mmol	0.3 mmol	1 mL	Trace
3	4CzIPN	MeCN	0.1 mmol	0.3 mmol	1 mL	Trace
4	$Ru(bpy)_3Cl_2$ $6H_2O$	MeCN	0.1 mmol	0.3 mmol	1 mL	33%
5	*fac*-$Ir(ppy)_3$	MeCN	0.1 mmol	0.3 mmol	1 mL	35%
6	$Ir(ppy)_2(dtbbpy)PF_6$	MeCN	0.3 mmol	0.6 mmol	1 mL	61%
7	$Ir(ppy)_2(dtbbpy)PF_6$	MeCN	0.3 mmol	0.6 mmol	2 mL	56%
8[c]	$Ir(ppy)_2(dtbbpy)PF_6$	MeCN	0.3 mmol	0.9 mmol	1 mL	59%
9	$Ir(ppy)_2(dtbbpy)PF_6$	MeCN	0.3 mmol	0.9 mmol	1 mL	71%
10	$Ir(ppy)_2(dtbbpy)PF_6$	DMC	0.3 mmol	0.9 mmol	1 mL	54%
11	$Ir(ppy)_2(dtbbpy)PF_6$	EtOH	0.3 mmol	0.9 mmol	1 mL	60%
12	$Ir(ppy)_2(dtbbpy)PF_6$	H_2O	0.3 mmol	0.9 mmol	1 mL	22%
13[d]	$Ir(ppy)_2(dtbbpy)PF_6$	MeCN	0.3 mmol	0.9 mmol	1 mL	54%
14[e]	$Ir(ppy)_2(dtbbpy)PF_6$	MeCN	0.3 mmol	0.9 mmol	1 mL	15%
15[f]	$Ir(ppy)_2(dtbbpy)PF_6$	MeCN	0.3 mmol	0.9 mmol	1 mL	0
16[g]	$Ir(ppy)_2(dtbbpy)PF_6$	MeCN	0.3 mmol	0.9 mmol	1 mL	0

[a] Reaction conditions: **1a** (0.3 mmol), **2a** (0.9 mmol), DABCO (0.3 mmol), and $Ir(ppy)_2(dtbbpy)PF_6$ (2.0 mol%) in MeCN (1.0 mL), with 24 W Blue LEDs irradiation at rt for 2 h under an air atmosphere. [b] isolated yield [c] 1.0 mol% $Ir(ppy)_2(dtbbpy)PF_6$. [d] without DABCO. [e] under an argon atmosphere. [f] without $Ir(ppy)_2(dtbbpy)PF_6$. [g] in dark.

Et = ethyl; DABCO = 1,4-diaza[2.2.2]bicyclooctane; ppy, 2-phenylpyridine; dtbbpy, 4,4'-Di-tert-butyl-2,2'-bipyridine; DMC, dimethyl carbonate; bpy, 2,2'-bipyridine; 4CzIPN, 1,2,3,5-tetrakis(carbazol-9-yl)-4,6-dicyanobenzene.

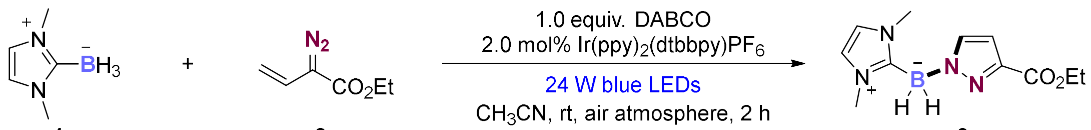

1a **2a** **3**

General procedure: To a 10 mL Schlenk flask equipped with a magnetic stir bar was added **1a** (33.0 mg, 0.3 mmol, 1.0 equiv.), **2a** (126.0 mg, 0.9 mmol, 3.0 equiv.), Ir(ppy)$_2$(dtbbpy)PF$_6$ (5.4 mg, 0.006 mmol, 0.02 equiv.), DABCO (33.6 mg, 0.3 mmol, 1.0 equiv.), dry MeCN (1.0 mL), under an air atmosphere. After the solution was stirred at a distance of ~3 cm from a 24 W blue LED at room temperature for 2 h. The solvent was removed by vacuum and the crude product was purified by flash chromatography on silica gel silica: 200~300 using ethyl acetate as eluant to provide pure boronated product **3** and as a white solid in 71% yield (52.8 mg). **^{1}H NMR** (400 MHz, $CDCl_3$, 300 K): δ (ppm) = 7.50 (d, J = 2.1 Hz, 1H), 6.84 (s, 2H), 6.71 (d, J = 2.1 Hz, 1H), 4.31 (q, J = 7.1 Hz, 2H), 3.63 (s, 6H), 1.33 (t, J = 7.1 Hz, 3H). **^{13}C NMR** (100 MHz, $CDCl_3$, 300 K): δ (ppm) = 163.6, 144.4, 136.6, 121.1, 107.8, 60.1, 35.9, 14.4. **^{11}B NMR** (128.4 MHz, $CDCl_3$, 300 K): δ (ppm) = -19.05 (t, J = 96.4 Hz). **HRMS** (ESI) m/z: $[M+H]^+$ Calcd for $C_{11}H_{18}BN_4O_2^+$: 249.1517; Found: 249.1517.

4. Synthetic applications and Gram-scale Synthesis

a) Gram-scale synthesis

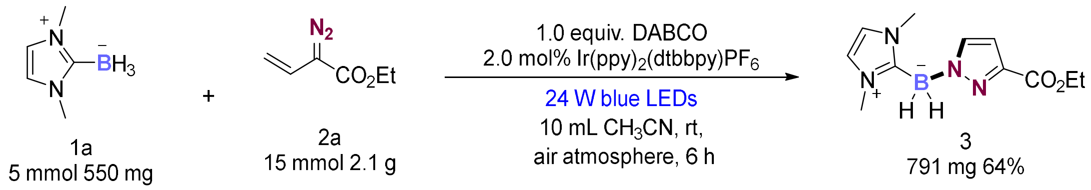

At air atmosphere, a 50 mL round-bottom-flask was added **1a** (0.55 g, 5.0 mmol, 1.0 equiv.), **2a** (2.1 g, 15.0 mmol, 3.0 equiv.), $Ir(ppy)_2(dtbbpy)PF_6$ (91.3 mg, 0.1 mmol, 0.02 equiv.), DABCO (0.56 g, 5 mmol, 1.0 equiv.), in dry MeCN (10 mL). After the solution was stirred at a distance of ~3 cm from 5 meters long blue light strip equipped with a fan cooling. After 6 hours, the solvent was removed by vacuum and the crude product were purified by flash chromatography on silica gel silica: 200~300; eluant: petroleum ether/ethyl acetate (1:1 to 0:1) to provide pure product **3** as a white solid in 64% yield (0.79 g).

b) Synthetic drugs *Mavacoxib* and *Deracoxib* analogues

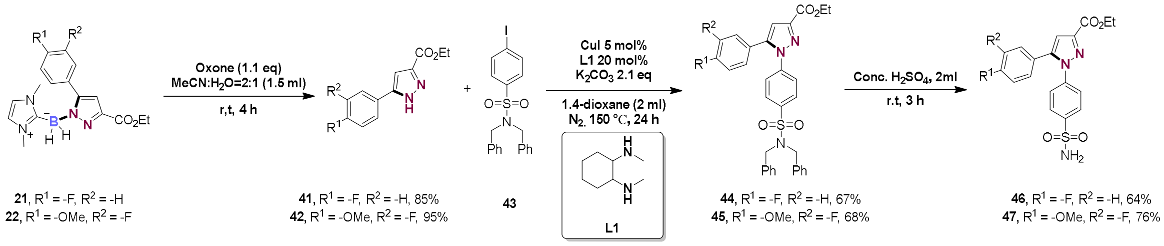

The following procedure is modified from the reported literatures.[21] To a 10 mL Schlenk flask equipped with a magnetic stir bar was added **21** (0.1 mmol, 1.0 equiv.), **Oxone** (68.0 mg, 1.1 mmol, 1.1 equiv.), MeCN (1.0 mL), H_2O (0.5 mL), under an air atmosphere After the solution was stirred at room temperature for 4 h, The solvent was removed by vacuum and the crude product was purified by flash chromatography on silica gel silica: 200~300; eluant: petroleum ether/ethyl acetate (3:1) to provide pure product **41** and as a white solid in 85% yield (20 mg).

To a 10 mL Schlenk flask equipped with a magnetic stir bar was added **41** (0.4 mmol, 1.0 equiv.), **43** (1.6 mmol, 4.0 equiv.), CuI (5 mol%, 0.02 mmol, 3.8 mg), L1 (20 mol%, 0.08 mmol, 3.8 mg), K_2CO_3 (0.84 mmol, 2.1 equiv.), dry 1,4-dioxane (2 mL), under an N_2 atmosphere. After the solution was stirred at 150 ℃ for 24 h, The solvent was removed by vacuum and the crude product was purified by flash chromatography on silica gel silica: 200~300; eluant: petroleum ether/ethyl acetate (5:1 to 3:1) to provide pure product in 67% yield (152 mg).

To a 25 mL round-bottom flash equipped with a magnetic stir bar was added **44** (0.2 mmol, 1.0 equiv.), Conc.H_2SO_4 (2 mL, slow and dropwise), Solutions were carefully stirred for 3 h at RT, at which point the solution

became dark. At this time, the reaction was diluted with H_2O (~20 mL), and the desired compounds extracted with Et_2O (3 x 30 mL). The organic layer was washed with 1 M $NaHCO_3$, dried with Na_2SO_4, and filtered. The solvent was removed under reduced pressure to afford a colourless liquid that was purified via column chromatography petroleum ether/ethyl acetate (1:1) to afford white powders product. in 64% yield (50 mg).

Ethyl 1-(4-(N,N-dibenzylsulfamoyl)phenyl)-5-(4-fluorophenyl)-1H-pyrazole-3-carboxylate (44)

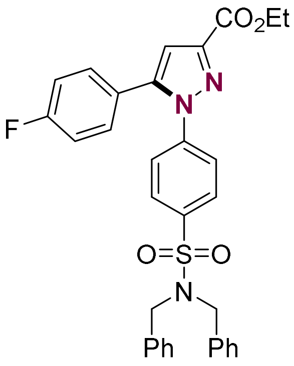

^{1}**H NMR** (400 MHz, $CDCl_3$, 300 K): δ (ppm) = 7.84 (d, J = 8.3 Hz, 2H), 7.79 – 7.74 (m, 2H), 7.57 (d, J = 8.3 Hz, 2H), 7.24 (s, 1H), 7.16 – 7.15 (m, 6H), 7.06 – 6.97 (m, 6H), 4.29 – 4.20 (m, 6H), 1.24 (t, J = 7.1 Hz, 3H). ^{13}**C NMR** (100 MHz, $CDCl_3$, 300 K): δ (ppm) = 163.1 (d, J = 246.8 Hz), 158.8, 151.4, 143.2, 140.4, 135.4, 134.9, 128.6, 128.5, 128.0 (d, J = 3.2 Hz), 127.8, 127.6, 127.6, 126.4, 115.8 (d, J = 21.7 Hz), 110.3, 61.5, 50.6, 14.1. ^{19}**F NMR** (376 MHz, $CDCl_3$, 300 K): δ (ppm) = -112.51 - -112.63 (m, 1F). **HRMS** (ESI) m/z: $[M+H]^+$ Calcd for $C_{32}H_{29}FN_3O_4S^+$: 570.1857; Found: 570.1850.

Ethyl 5-(4-fluorophenyl)-1-(4-sulfamoylphenyl)-1H-pyrazole-3-carboxylate (46)

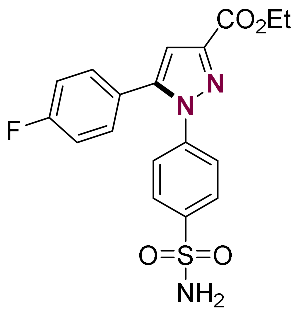

^{1}**H NMR** (400 MHz, d_6-DMSO, 300 K): δ (ppm) = δ 7.14 (dd, J = 8.5, 5.4 Hz, 2H), 7.09 (d, J = 8.2 Hz, 2H), 6.92 (d, J = 8.2 Hz, 2H), 6.81 (s, 1H), 6.67 (s, 2H), 6.44 (t, J = 8.6 Hz, 2H), 3.39 (q, J = 7.1 Hz, 2H), 0.37 (t, J = 7.0 Hz, 3H). ^{13}**C NMR** (100 MHz, d_6-DMSO, 300 K): δ (ppm) = 163.6, 161.2, 158.4, 150.5, 143.9, 142.2, 134.9, 128.0, 127.75 (d, J = 8.4 Hz), 126.23 (d, J = 11.0 Hz), 115.82 (d, J = 21.7 Hz), 110.1, 61.3, 13.9. ^{19}**F NMR** (376 MHz, d_6-DMSO, 300 K): δ (ppm) = -112.86 – 113.0 (m, 1F). **HRMS** (ESI) m/z: $[M+H]^+$ Calcd for $C_{18}H_{17}FN_3O_4S^+$: 390.0918; Found: 390.0917.

Ethyl 1-(4-(N,N-dibenzylsulfamoyl)phenyl)-5-(3-fluoro-4-methoxyphenyl)-1H-pyrazole-3-carboxylate (45)

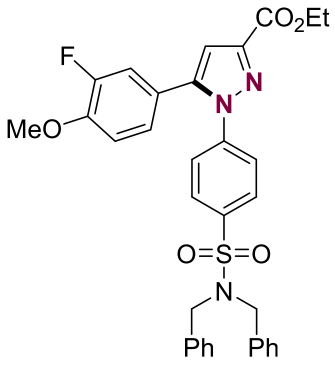

^{1}**H NMR** (400 MHz, $CDCl_3$, 300 K): δ (ppm) = 7.93 (d, J = 8.6 Hz, 2H), 7.65 (d, J = 8.7 Hz, 2H), 7.61 – 7.56 (m, 1H), 7.29 (s, 1H), 7.26 – 7.21 (m, 7H), 7.10 – 7.07 (m, 4H), 7.01 (t, J = 8.5 Hz, 1H), 4.37 – 4.29 (m, 6H), 3.92 (s, 3H), 1.33 (t, J = 7.1 Hz, 3H). ^{13}**C NMR** (100 MHz, $CDCl_3$, 300 K): δ (ppm) = 158.8, 152.5, (d, J = 244.4 Hz), 151.2, 148.1 (d, J = 10.6 Hz), 143.2, 140.3, 135.3, 134.8, 128.6, 128.5, 127.7, 127.5, 126.4, 125.0 (d, J = 7.0 Hz), 121.7 (d, J = 3.5 Hz), 118.4, 114.1 (d, J =19.7 Hz), 113.5, 110.1, 61.5, 56.2, 50.6, 14.0. ^{19}**F NMR** (376 MHz, $CDCl_3$, 300 K): δ (ppm) = -134.90 – -134.99 (m,

1F). **HRMS** (ESI) m/z: $[M+H]^+$ Calcd for $C_{33}H_{31}FN_3O_5S+^+$: 600.1963; Found: 600.1968.

Ethyl 5-(3-fluoro-4-methoxyphenyl)-1-(4-sulfamoylphenyl)-1H-pyrazole-3-carboxylate (47)

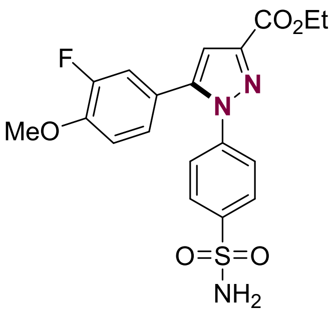

^{1}H NMR (400 MHz, *d*$_6$-DMSO, 300 K): δ (ppm) = 7.95 (d, *J* = 8.3 Hz, 2H), 7.81 – 7.72 (m, 4H), 7.65 (s, 1H), 7.53 (s, 2H), 7.24 (t, *J* = 8.7 Hz, 1H), 4.24 (q, *J* = 7.1 Hz, 2H), 3.88 (s, 3H), 1.22 (t, *J* = 7.1 Hz, 3H). **^{13}C NMR** (100 MHz, *d*$_6$-DMSO, 300 K): δ (ppm) = 158.4, 151.7 (d, *J* = 244.4 Hz), 150.3, 147.5 (d, *J* = 10.5 Hz), 143.8, 142.2, 134.8, 126.2 (d, *J* = 10.3 Hz), 124.5(d, *J* = 7.2 Hz), 122.1 (d, *J* = 3.4 Hz), 114.1, 113.1 (d, *J* = 19.5 Hz), 110.0, 61.3, 56.1, 13.9. **^{19}F NMR** (376 MHz, *d*$_6$-DMSO, 300 K): δ (ppm) = -134.79 – -134.94 (m, 1F). **HRMS** (ESI) m/z: $[M+H]^+$ Calcd for $C_{19}H_{19}FN_3O_5S^+$: 420.1024; Found: 420.1026.

c) Cell culture and cell viability test.

HeLa, Hep G2, A549 and HL-7702 cells were purchased from Shanghai Institute of Biochemistry and Cell Biology (Chinese Academy of Sciences, China). The cells were cultured in a Dulbecco's modified Eagle's medium (DMEM, glucose 4.5 g/L, Gibco) supplemented with 10% fetal bovine serum (FBS, Gibco), 100 mg/mL streptomycin, 100 U/mL penicillin and 4 mM L-glutamine at 37 °C in 5% CO_2/95% air.

The cell viabilities of **etoposide, 12, 13, 14, 24** and **25** were studied by MTT assays. The corresponding cancer cells (10^5 cell/mL, 10 mL) were dispersed with 96-well microtiter plates to a total volume of 100 µL/well. Plates were maintained at 37 °C in 5% CO_2/95% air for 24 h. The cells were incubated for 18 h with different concentrations of probe (0, 1, 5, 8 and 10 µM) in the medium at 37 °C. Then, 10 µL MTT (5 mg/mL) solution was added into each well and incubated for additional 4 h. After removal of supernatant and addition of 100 µL of DMSO into each well, the cells were shaken for 10 min, and then the absorbance in each well was read at 492 nm using a microplate reader. The cell viability (%) was calculated according to the equation: cell viability % = A/B × 100%, where A represents the absorbance of each well treated with probe, and B represents that of the control well.

(A) antitumor activities of the obtained boron-handled pyrazoles against HeLa cells

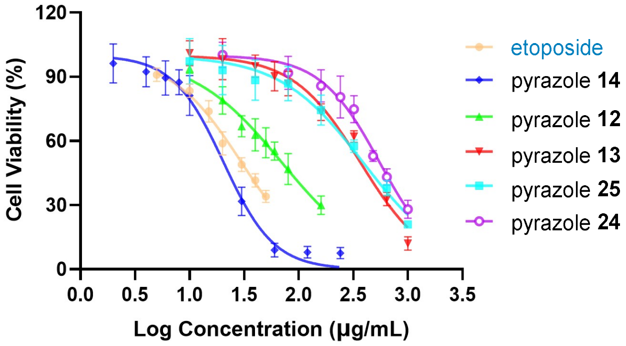

Compound	IC_{50} (µg/mL)
etoposide	29.83
pyrazole **25**	20.30
pyrazole **12**	69.72
pyrazole **14**	366.5
pyrazole **13**	387.5
pyrazole **24**	599.8

(B) antitumor activities of pyrazole **25** against Hep G2 cells

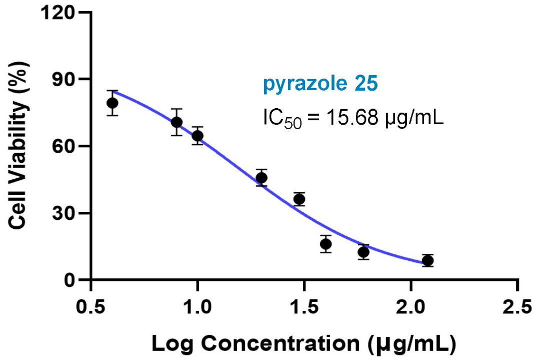

(C) antitumor activities of pyrazole **25** against A549 cells

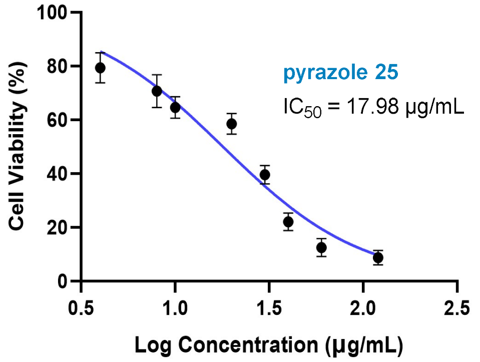

Figure S1. The antiproliferative activity of the obtained boron-handled pyrazoles in cancer cell lines

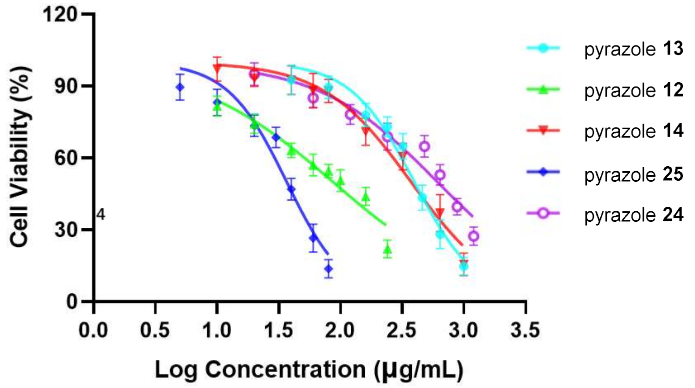

Compound	IC_{50} (µg/mL)
pyrazole **25**	36.63
pyrazole **12**	86.73
pyrazole **14**	372.6
pyrazole **13**	396.7
pyrazole **24**	608.9

Figure S2. The antiproliferative activity of the obtained boron-handled pyrazoles in HL-7702 cells (normal hepatic cells).

5. Mechanism studies

a) Stern-Volmer Fluorescent Quenching

Fluorescence spectra was collected on Hitachi F-7000 Fluorescence Spectrophotometer for all experiments. General procedure: To a solution of $Ir(ppy)_2(dtbbpy)PF_6$ (0.001 mM) in MeCN was added appropriate amount of quencher in a quartz cuvette. Then the emission of the sample was collected.

All $Ir(ppy)_2dtbbpyPF_6$ (τ_0 = 557 ns) solutions (concentration of 1 μM) were excited at 400 nm and the emission intensity was collected at 522 nm.

$$\mathbf{I_0/I = 1 + \mathit{K_{SV}}[Q]}$$

$$\mathbf{\mathit{K_{SV}} = \mathit{k_q}\tau_0}$$

Where $\mathbf{I_0}$ is the luminescence intensity in the absence of the quencher, **I** is the intensity in the presence of the quencher, $\boldsymbol{K_{SV}}$ is the Stern−Volmer constant, $\boldsymbol{k_q}$ is the quenching rate, $\boldsymbol{\tau_0}$ is the life-time of the photoredox catalyst ($\tau_0 = 5.57 \times 10^{-7}$ s) for $Ir(ppy)_2(dtbbpy)PF_6$, and **[Q]** is the concentration of the quencher.

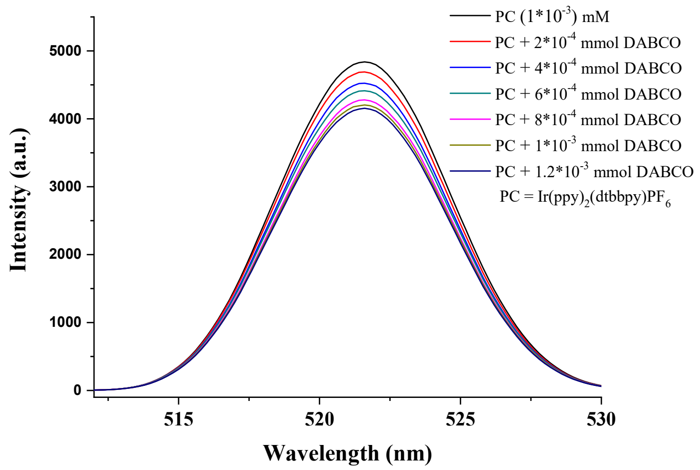

Figure S3. The luminescence spectra of $Ir(ppy)_2(dtbbpy)PF_6$ with different concentration of **DABCO** excited at 400 nm

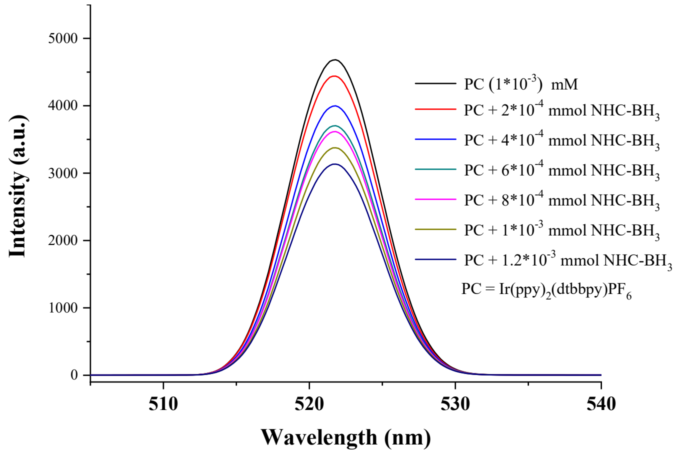

Figure S4. The luminescence spectra of $Ir(ppy)_2(dtbbpy)PF_6$ with different concentration of **$NHC\text{-}BH_3$** excited at 400 nm

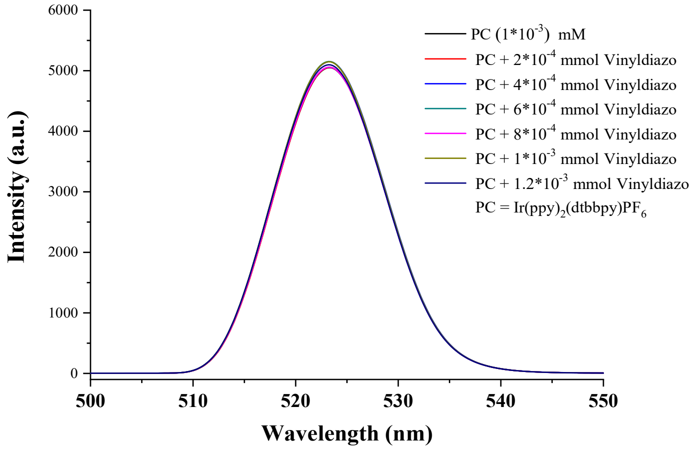

Figure S5. The luminescence spectra of $Ir(ppy)_2(dtbbpy)PF_6$ with different concentration of **Vinyldiazo** excited at 400 nm

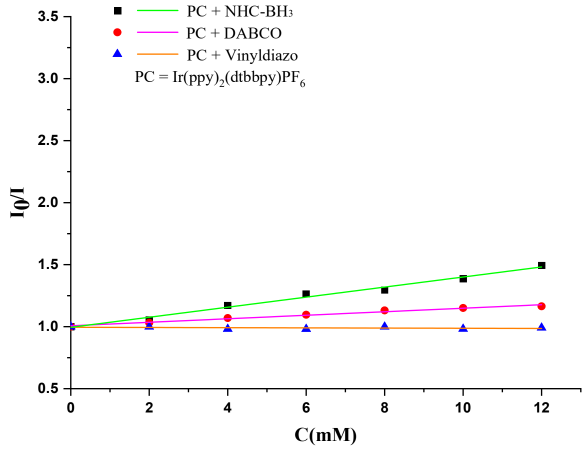

Figure S6. Stern-Volmer plot of **$Ir(ppy)_2(dtbbpy)PF_6$** at different quenchers.

Table S2. k_q calculations of **DABCO**, **NHC-BH**$_3$, **Vinyldiazo**.

**Quencher**	k_q (10^8 $M^{-1}S^{-1}$)
**DABCO**	**0.73**
**NHC-BH**$_3$	**0.25**
**Vinyldiazo**	**0**

b) Radical-blocking

In order to ensure whether the putative radical was trapped by TEMPO , APCI-MS analysis of the crude reaction mixture was performed. The resulting mass spectrum clearly shows a peak corresponding to the coupled product between TEMPO radical and the expected ·BH$_2$-NHC (HRMS (APCI): $C_{14}H_{29}BN_3O^+$ $[M+H]^+$ Calcd 266.2398 ,Found: 266.2398.

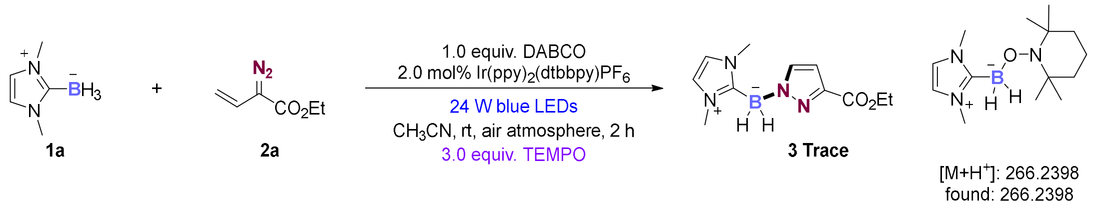

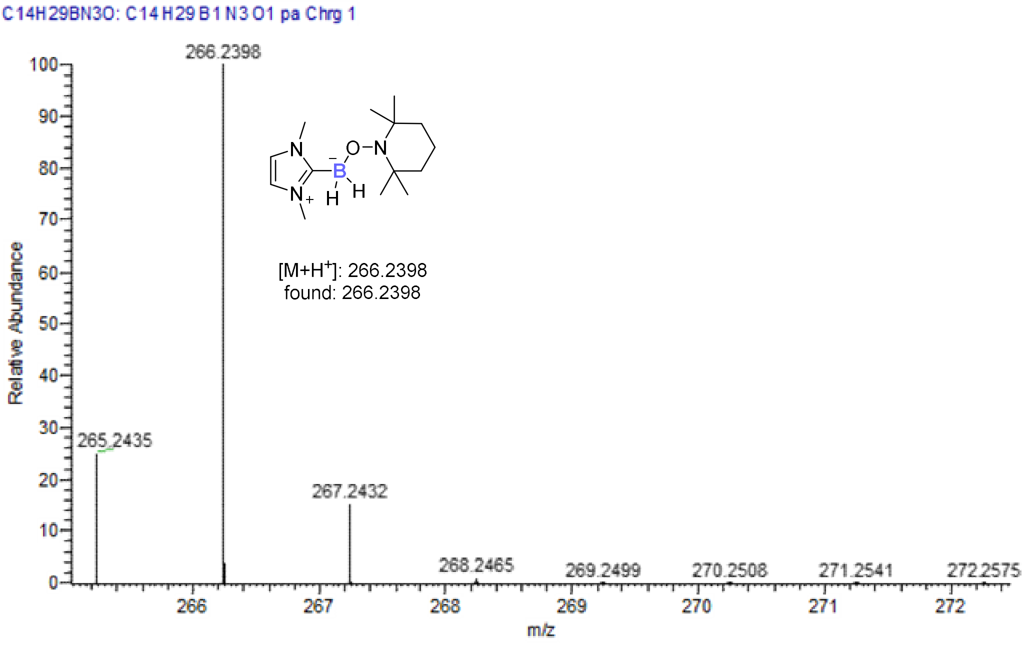

c) Radical-clocking

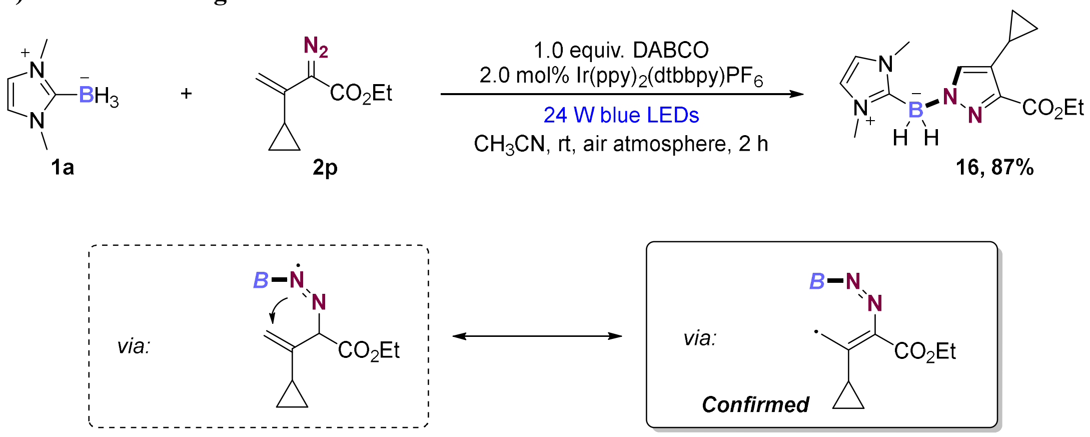

To a 10 mL Schlenk flask equipped with a magnetic stir bar was added **1a** (33.0 mg, 0.3 mmol, 1.0 equiv.), **2p** (162.0 mg, 0.9 mmol, 3.0 equiv.), Ir(ppy)$_2$(dtbbpy)PF$_6$ (5.4 mg, 0.006 mmol, 0.02 equiv.), DABCO (33.6 mg, 0.3 mmol, 1.0 equiv.), dry MeCN (1.0 mL), under an air atmosphere. After the solution was stirred at a distance of ~3 cm from a 24 W blue LED at room temperature for 2 h. The solvent was removed by vacuum and the crude product was purified by flash chromatography on silica gel silica: 200~300; eluant: petroleum ether/ethyl acetate (0:1) to provide pure boronated product **3** and as a white solid.

d) $^{\bullet-}O_2$-quenching experiment

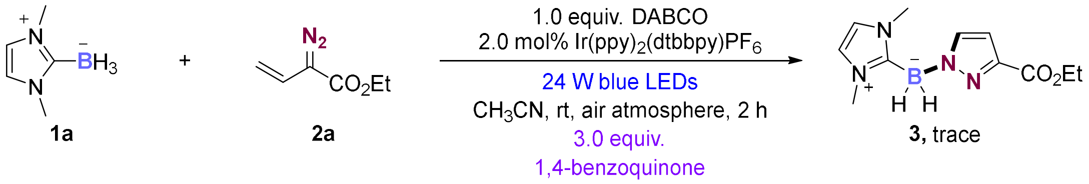

To a 10 mL Schlenk flask equipped with a magnetic stir bar was added **1a** (33.0 mg, 0.3 mmol, 1.0 equiv.), **2a** (126.0 mg, 0.9 mmol, 3.0 equiv.), Ir(ppy)$_2$(dtbbpy)PF$_6$ ((5.4 mg, 0.006 mmol, 0.02 equiv.), DABCO (33.6 mg, 0.3 mmol, 1.0 equiv.), dry MeCN (1.0 mL), 1,4-benzoquinone (97.2 mg, 0.9 mmol, 3.0 equiv) under an air atmosphere. After the solution was stirred at a distance of ~3 cm from a 24 W blue LED at room temperature for 2 h. The solvent was removed by vacuum and the crude product was purified by flash chromatography on silica gel silica: 200~300; eluant: petroleum ether/ethyl acetate (0:1) to provide pure boronated product **3** and as a white solid in trace.

e) Pyrazole 40 isolation and control experiments

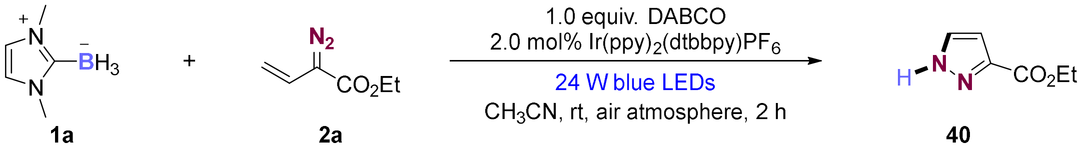

To a 10 mL Schlenk flask equipped with a magnetic stir bar was added **1a** (33.0 mg, 0.3 mmol, 1.0 equiv.), **2a** (126.0 mg, 0.9 mmol, 3.0 equiv.), Ir(ppy)2(dtbbpy)PF6 ((5.4 mg, 0.006 mmol, 0.02 equiv.), DABCO (33.6 mg, 0.3 mmol, 1.0 equiv.), dry MeCN (1.0 mL), under an air atmosphere. After the solution was stirred at a distance of ~3 cm from a 24 W blue LED at room temperature for 2 h. The solvent was removed by vacuum and the crude product was purified by flash chromatography on silica gel silica: 200~300; eluant: petroleum ether/ethyl acetate (3:1) to provide pure product **40** .

entry	variation from standard conditions	yield (%)
1	none	N.D.
2	without DABCO	N.D.
3	without Ir(ppy)2(dtbbpy)PF6 and DABCO	10 %

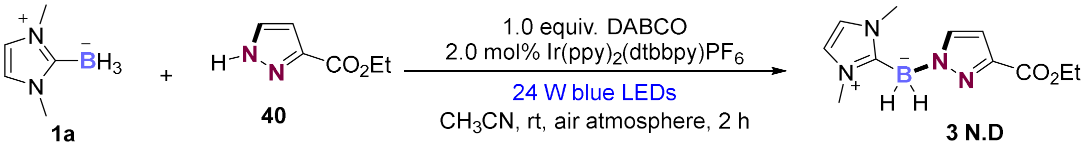

To a 10 mL Schlenk flask equipped with a magnetic stir bar was added **1a** (33.0 mg, 0.3 mmol, 1.0 equiv.), **40** (126 mg, 0.9 mmol, 3.0 equiv.), Ir(ppy)2(dtbbpy)PF6 (5.4 mg, 0.006 mmol, 0.02 equiv.), DABCO (33.6 mg, 0.3 mmol, 1.0 equiv.), dry MeCN (1.0 mL), under an air atmosphere. After the solution was stirred at a distance of ~3 cm from a 24 W blue LED at room temperature for 2 h. The solvent was removed by vacuum and the crude product was purified by flash chromatography on silica gel silica: 200~300; eluant: petroleum ether/ethyl acetate (3:1) to provide pure product **40.**

f) EPR experiments [22]

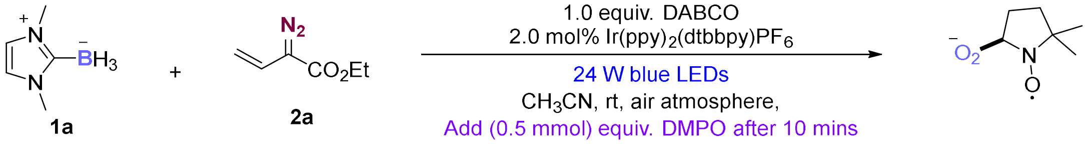

To a 10 mL Schlenk flask equipped with a magnetic stir bar was added 1a (33.0 mg, 0.3 mmol, 1.0 equiv.), 2a (126.0 mg, 0.9 mmol, 3.0 equiv.), Ir(ppy)2(dtbbpy)PF6 ((5.4 mg, 0.006 mmol, 0.02 equiv.), DABCO (33.6 mg, 0.3 mmol, 1.0 equiv.), dry MeCN (1.0 mL), under an air atmosphere. After the solution was stirred at a distance of ~3 cm from a 24 W blue LED at room temperature for 10 min, DMPO (33.9 mg, 0.3 mmol,) was added. Upon completion, the reaction mixture was analysis by EPR.

The EPR experiment was taken on a Bruck EPR5000 instrument. $O_2^{\cdot -}$ centered radical[22] measurement conditions: Power 10 mW, Frequency 9.41618 GHz, B from 330 mT to 345 mT, Sweep width 50 mT, Sweep time 60 s, 1 time, Modulation 0.20000 mT, Temperature 302.4 K. (g = 2.0063, A_N = 14 G,A_H = 12.4 G).

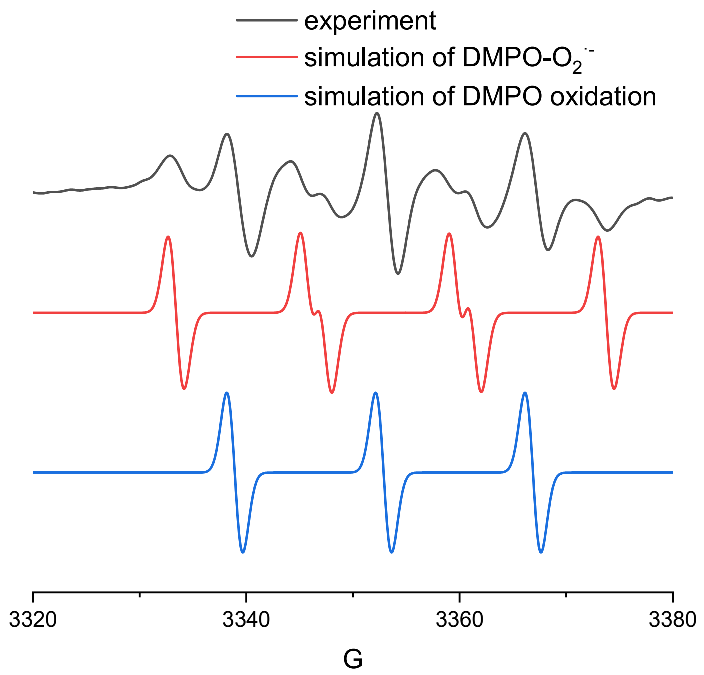

Figure S7 EPR spectrum

6. Spectral Data of Boronated Products

(1,3-dimethyl-1H-imidazol-3-ium-2-yl)(3-(ethoxycarbonyl)-1H-pyrazol-1-yl)dihydroborate (3)

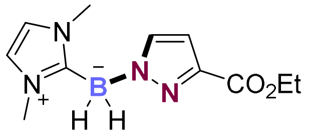

According to ***GP*** with **1a** (33.0 mg, 0.3 mmol, 1.0 equiv.), **2a** (126 mg, 0.9 mmol, 3.0 equiv.), Ir(ppy)$_2$(dtbbpy)PF$_6$ (5.4 mg, 0.006 mmol, 0.02 equiv.), DABCO (33.6 mg, 0.3 mmol, 1.0 equiv.), in 1.0 mL MeCN for 2 h. Purification by silica gel chromatography afforded the desired **3** and as a white solid in 71% yield (52.8 mg). ^{1}**H NMR** (400 MHz, CDCl$_3$, 300 K): δ (ppm) = 7.50 (d, J = 2.1 Hz, 1H), 6.84 (s, 2H), 6.71 (d, J = 2.1 Hz, 1H), 4.31 (q, J = 7.1 Hz, 2H), 3.63 (s, 6H), 1.33 (t, J = 7.1 Hz, 3H). ^{13}**C NMR** (100 MHz, CDCl$_3$, 300 K): δ (ppm) = 163.6, 144.4, 136.6, 121.1, 107.8, 60.1, 35.9, 14.4. ^{11}**B NMR** (128.4 MHz, CDCl$_3$, 300 K): δ (ppm) = -19.05 (t, J = 96.4 Hz). **HRMS** (ESI) m/z: [M+H]$^+$ Calcd for $C_{11}H_{18}BN_4O_2^+$: 249.1517; Found: 249.1517.

(1,3-dimethyl-1H-imidazol-3-ium-2-yl)(3-(propoxycarbonyl)-1H-pyrazol-1-yl)dihydroborate (4)

According to ***GP*** with **1a** (33.0 mg, 0.3 mmol, 1.0 equiv.), **2b** (139 mg, 0.9 mmol, 3.0 equiv.),

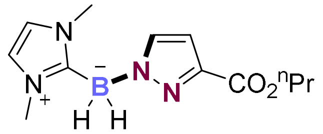

Ir(ppy)$_2$(dtbbpy)PF$_6$ (5.4 mg, 0.006 mmol, 0.02 equiv.), DABCO (33.6 mg, 0.3 mmol, 1.0 equiv.), in 1.0 mL MeCN for 3 h. Purification by silica gel chromatography afforded the desired **4** and as a white solid in 70% yield (55 mg). ^{1}**H NMR** (400 MHz, CDCl$_3$, 300 K): δ (ppm) = 7.50 (d, J = 2.1 Hz, 1H), 6.84 (s, 2H), 6.69 (d, J = 2.1 Hz, 1H), 4.20 (t, J = 6.9 Hz, 2H), 3.64 (s, 6H), 1.78 – 1.68 (m, 2H), 0.96 (t, J = 7.4 Hz, 3H). ^{13}**C NMR** (100 MHz, CDCl$_3$, 300 K): δ (ppm) = 163.7, 144.4, 136.5, 121.1, 107.7, 65.6, 35.9, 22.1, 10.4. ^{11}**B NMR** (128.4 MHz, CDCl3, 300 K): δ (ppm) = -19.07 (t, J = 93.0 Hz). **HRMS** (ESI) m/z: [M+H]$^+$ Calcd for $C_{12}H_{20}BN_4O_2^+$: 263.1674; Found: 249.1675.

(3-((benzyloxy)carbonyl)-1H-pyrazol-1-yl)(1,3-dimethyl-1H-imidazol-3-ium-2-yl)dihydroborate (5)

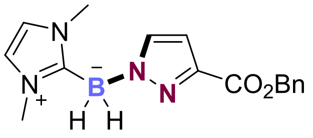

According to ***GP*** with **1a** (33.0 mg, 0.3 mmol, 1.0 equiv.), **2c** (182 mg, 0.9 mmol, 3.0 equiv.), Ir(ppy)$_2$(dtbbpy)PF$_6$ (5.4 mg, 0.006 mmol, 0.02 equiv.), DABCO (33.6 mg, 0.3 mmol, 1.0 equiv.), in 1.0 mL MeCN for 3 h. Purification by silica gel chromatography afforded the desired **5** and as a white solid in 77% yield (72 mg). ^{1}**H NMR** (400 MHz, CDCl$_3$, 300 K): δ (ppm) = 7.52 (d, J = 2.1 Hz, 1H), 7.45 – 7.39 (m, 2H), 7.36 – 7.28 (m, 3H), 6.83 (s, 2H), 6.74 (d, J = 2.1 Hz, 1H), 5.32 (s, 2H), 3.64 (s, 6H). ^{13}**C NMR** (100 MHz, CDCl$_3$, 300 K): δ (ppm) = 163.3, 143.9, 136.5, 128.3, 128.1, 127.8, 121.1, 108.0, 65.6, 35.8. ^{11}**B NMR** (128.4 MHz, CDCl$_3$, 300 K): δ (ppm) = -17.08 – -21.04 (m). **HRMS** (ESI) m/z: [M+H]$^+$ Calcd for $C_{16}H_{20}BN_4O_2^+$: 311.1674; Found: 311.1674.

(3-((benzo[d][1,3]dioxol-5-ylmethoxy)carbonyl)-1H-pyrazol-1-yl)(1,3-dimethyl-1H-imidazol-3-ium-2-yl)dihydroborate (6)

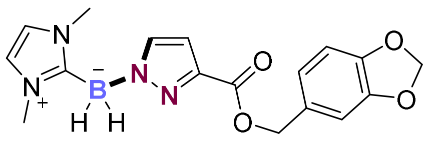

According to ***GP*** with **1a** (33.0 mg, 0.3 mmol, 1.0 equiv.), **2d** (209 mg, 0.9 mmol, 3.0 equiv.), $Ir(ppy)_2(dtbbpy)PF_6$ (5.4 mg, 0.006 mmol, 0.02 equiv.), DABCO (33.6 mg, 0.3 mmol, 1.0 equiv.), in 1.0 mL MeCN for 3 h. Purification by silica gel chromatography afforded the desired **6** and as a white solid in 72% yield (77 mg). **^{1}H NMR** (400 MHz, $CDCl_3$, 300 K): δ (ppm) = 7.51 (d, J = 2.1 Hz, 1H), 6.94 (s, 1H), 6.92 – 6.88 (m, 1H), 6.84 (s, 2H), 6.78 – 6.75 (m, 1H), 6.72 (d, J = 2.1 Hz, 1H), 5.94 (s, 2H), 5.22 (s, 2H), 3.66 (s, 6H). **^{13}C NMR** (100 MHz, $CDCl_3$, 300 K): δ (ppm) = 163.3, 147.6, 147.3, 143.9, 136.6, 130.4, 122.1, 121.1, 109.0, 108.0, 101.0, 65.6, 35.9. **^{11}B NMR** (128.4 MHz, $CDCl_3$, 300 K): δ (ppm) = -16.39 – -21.93 (m). **HRMS** (ESI) m/z: $[M+H]^+$ Calcd for $C_{17}H_{20}BN_4O_4^+$: 355.1572; Found: 355.1574.

(1,3-dimethyl-1H-imidazol-3-ium-2-yl)(3-(isopropoxycarbonyl)-1H-pyrazol-1-yl)dihydroborate (7)

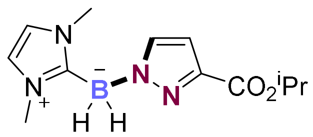

According to ***GP*** with **1a** (33.0 mg, 0.3 mmol, 1.0 equiv.), **2c** (139 mg, 0.9 mmol, 3.0 equiv.), $Ir(ppy)_2(dtbbpy)PF_6$ (5.4 mg, 0.006 mmol, 0.02 equiv.), DABCO (33.6 mg, 0.3 mmol, 1.0 equiv.), in 1.0 mL MeCN for 3 h. Purification by silica gel chromatography afforded the desired **7** and as a white solid in 75% yield (59 mg). **^{1}H NMR** (400 MHz, $CDCl_3$, 300 K): δ (ppm) = 7.50 (d, J = 2.1 Hz, 1H), 6.84 (s, 2H), 6.67 (d, J = 2.1 Hz, 1H), 5.24 – 5.14 (m, 1H), 3.65 (s, 6H), 1.31 (d, J = 6.3 Hz, 6H). **^{13}C NMR** (100 MHz, $CDCl_3$, 300 K): δ (ppm) = 163.2, 144.8, 136.4, 121.1, 107.6, 67.2, 35.9, 22.0. **^{11}B NMR** (128.4 MHz, $CDCl_3$, 300 K): δ (ppm) = -19.10 (t, J = 88.7 Hz). **HRMS** (ESI) m/z: $[M+H]^+$ Calcd for $C_{12}H_{20}BN_4O_2^+$: 263.1674; Found: 249.1674.

(3-(tert-butoxycarbonyl)-1H-pyrazol-1-yl)(1,3-dimethyl-1H-imidazol-3-ium-2-yl)dihydroborate, hydrogen salt (8)

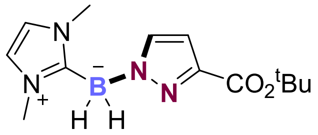

According to ***GP*** with **1a** (33.0 mg, 0.3 mmol, 1.0 equiv.), **2f** (151 mg, 0.9 mmol, 3.0 equiv.), $Ir(ppy)_2(dtbbpy)PF_6$ (5.4 mg, 0.006 mmol, 0.02 equiv.), DABCO (33.6 mg, 0.3 mmol, 1.0 equiv.), in 1.0 mL MeCN for 2 h. Purification by silica gel chromatography afforded the desired **8** and as a white solid in 60% yield (49.7 mg). **^{1}H NMR** (400 MHz, $CDCl_3$, 300 K): δ (ppm) = 7.48 (d, J = 2.1 Hz, 1H), 6.84 (s, 2H), 6.60 (d, J = 2.1 Hz, 1H), 3.69 (s, 6H), 1.53 (s, 9H). **^{13}C NMR** (100 MHz, $CDCl_3$, 300 K): δ (ppm) = 162.9, 145.6, 136.2, 121.1, 107.3, 79.9, 36.0, 28.3. **^{11}B NMR** (128.4 MHz, $CDCl_3$, 300 K): δ (ppm) = -19.35 (t, J = 74.4 Hz). **HRMS** (ESI) m/z: $[M+H]^+$ Calcd for $C_{13}H_{22}BN_4O_2^+$: 277.1830; Found: 277.1831.

(3-((((3s,5s,7s)-adamantan-1-yl)oxy)carbonyl)-1H-pyrazol-1-yl)(1,3-dimethyl-1H-imidazol-3-ium-2-yl)dihydroborate (9)

According to ***GP*** with **1a** (33.0 mg, 0.3 mmol, 1.0 equiv.), **2g** (221 mg, 0.9 mmol, 3.0 equiv.), $Ir(ppy)_2(dtbbpy)PF_6$ (5.4 mg, 0.006 mmol, 0.02 equiv.), DABCO (33.6 mg, 0.3 mmol, 1.0 equiv.), in 1.0 mL

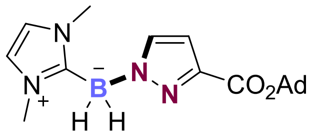

MeCN for 3 h. Purification by silica gel chromatography afforded the desired **9** and as a white solid in 65% yield (69 mg). **^{1}H NMR** (400 MHz, $CDCl_3$, 300 K): δ (ppm) = 7.48 (d, J = 2.1 Hz, 1H), 6.84 (s, 2H), 6.59 (d, J = 2.1 Hz, 1H), 3.69 (s, 6H), 2.22 (d, J = 2.5 Hz, 6H), 2.16 (s, 3H), 1.71 – 1.62 (m, 6H). **^{13}C NMR** (100 MHz, $CDCl_3$, 300 K): δ (ppm) = 162.6, 145.7, 136.1, 121.1, 107.3, 80.0, 41.4, 36.3, 36.1, 30.8. **^{11}B NMR** (128.4 MHz, $CDCl_3$, 300 K): δ (ppm) = -16.93 – -21.58 (m). **HRMS** (ESI) m/z: $[M+H]^+$ Calcd for $C_{17}H_{20}BN_4O_4^+$: 355.2300; Found: 355.2301.

(1,3-dimethyl-1H-imidazol-3-ium-2-yl)(3-(((2-methylallyl)oxy)carbonyl)-1H-pyrazol-1-yl)dihydroborate (10)

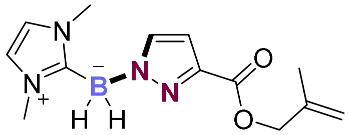

According to ***GP*** with **1a** (33.0 mg, 0.3 mmol, 1.0 equiv.), **2h** (150 mg, 0.9 mmol, 3.0 equiv.), $Ir(ppy)_2(dtbbpy)PF_6$ (5.4 mg, 0.006 mmol, 0.02 equiv.), DABCO (33.6 mg, 0.3 mmol, 1.0 equiv.), in 1.0 mL MeCN for 3 h. Purification by silica gel chromatography afforded the desired **10** and as a white solid in 78% yield (64 mg). **^{1}H NMR** (400 MHz, $CDCl_3$, 300 K): δ (ppm) = 7.51 (d, J = 2.1 Hz, 1H), 6.84 (s, 2H), 6.72 (d, J = 2.1 Hz, 1H), 5.02 (s, 1H), 4.90 (s, 1H), 4.68 (s, 2H), 3.65 (s, 6H), 1.78 (s, 3H). **^{13}C NMR** (100 MHz, $CDCl_3$, 300 K): δ (ppm) = 163.1, 143.9, 140.4, 136.5, 121.1, 112.2, 107.8, 67.0, 35.9, 19.5. **^{11}B NMR** (128.4 MHz, $CDCl_3$, 300 K): δ (ppm) = -19.13 (t, J = 90.8 Hz). **HRMS** (ESI) m/z: $[M+H]^+$ Calcd for $C_{13}H_{20}BN_4O_2^+$: 275.1674; Found: 275.1674.

(1,3-dimethyl-1H-imidazol-3-ium-2-yl)(3-(((3-phenylprop-2-yn-1-yl)oxy)carbonyl)-1H-pyrazol-1-yl)dihydroborate (11)

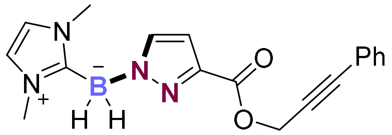

According to ***GP*** with **1a** (33.0 mg, 0.3 mmol, 1.0 equiv.), **2i** (203 mg, 0.9 mmol, 3.0 equiv.), $Ir(ppy)_2(dtbbpy)PF_6$ (5.4 mg, 0.006 mmol, 0.02 equiv.), DABCO (33.6 mg, 0.3 mmol, 1.0 equiv.), in 1.0 mL MeCN for 4 h. Purification by silica gel chromatography afforded the desired **11** and as a white solid in 75% yield (75 mg). **^{1}H NMR** (400 MHz, $CDCl_3$, 300 K): δ (ppm) = 7.54 (d, J = 2.1 Hz, 1H), 7.45 – 7.41 (m, 2H), 7.32 – 7.29 (m, 3H), 6.84 (s, 2H), 6.79 (d, J = 2.1 Hz, 1H), 5.09 (s, 2H), 3.65 (s, 6H). **^{13}C NMR** (100 MHz, $CDCl_3$, 300 K): δ (ppm) = 162.7, 143.5, 136.8, 131.8, 128.5, 128.2, 122.4, 121.2, 108.3, 86.0, 83.7, 52.4, 36.0. **^{11}B NMR** (128.4 MHz, $CDCl_3$, 300 K): δ (ppm) = -17.09 – -21.16 (m). **HRMS** (ESI) m/z: $[M+H]^+$ Calcd for $C_{18}H_{20}BN_4O_2^+$: 335.1674; Found: 335.1674.

(1,3-dimethyl-1H-imidazol-3-ium-2-yl)(3-(3,5-dimethyl-1H-pyrazole-1-carbonyl)-1H-pyrazol-1-yl)dihydroborate (12)

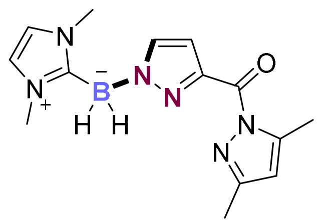

According to ***GP*** with **1a** (33.0 mg, 0.3 mmol, 1.0 equiv.), **2j** (171 mg, 0.9 mmol, 3.0 equiv.), Ir(ppy)$_2$(dtbbpy)PF$_6$ (8.1 mg, 0.009 mmol, 0.03 equiv.), DABCO (33.6 mg, 0.3 mmol, 1.0 equiv.), in 1.0 mL MeCN for 4.5 h. Purification by silica gel chromatography afforded the desired **12** and as a white solid in 60 % yield (54 mg). **^{1}H NMR** (400 MHz, CDCl$_3$, 300 K): δ (ppm) = 7.56 (d, *J* = 1.9 Hz, 1H), 7.17 (d, *J* = 1.8 Hz, 1H), 6.84 (s, 2H), 5.97 (s, 1H), 3.75 (s, 6H), 2.58 (s, 3H), 2.25 (s, 3H). **^{13}C NMR** (100 MHz, CDCl$_3$, 300 K): δ (ppm) = 162.3, 151.3, 145.0, 144.9, 135.9, 121.1, 111.7, 110.2, 36.1, 14.4, 13.9. **^{11}B NMR** (128.4 MHz, CDCl$_3$, 300 K) : δ (ppm) = -16.26 – -22.02 (m). **HRMS** (ESI) m/z: [M+H]$^+$ Calcd for $C_{14}H_{20}BN_6O^+$: 299.1786; Found: 299.1785.

(1,3-dimethyl-1H-imidazol-3-ium-2-yl)(3-(methoxy(methyl)carbamoyl)-1H-pyrazol-1-yl)dihydroborate (13)

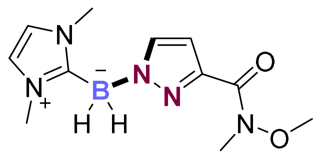

According to ***GP*** with **1a** (33.0 mg, 0.3 mmol, 1.0 equiv.), **2k** (140 mg, 0.9 mmol, 3.0 equiv.), Ir(ppy)$_2$(dtbbpy)PF$_6$ (8.1 mg, 0.009 mmol, 0.03 equiv.), DABCO (33.6 mg, 0.3 mmol, 1.0 equiv.), in 1.0 mL MeCN for 11 h. Purification by silica gel chromatography afforded the desired **13** and as a white solid in 40% yield (32 mg). **^{1}H NMR** (400 MHz, CDCl$_3$, 300 K): δ (ppm) = 7.49 (d, *J* = 1.6 Hz, 1H), 6.85 (s, 2H), 6.66 (d, *J* = 1.1 Hz, 1H), 3.69 (s, 3H), 3.68 (s, 6H), 3.41 (s, 3H). **^{13}C NMR** (100 MHz, CDCl$_3$, 300 K): δ (ppm) = 164.2, 145.5, 135.8, 121.0, 107.5, 60.9, 35.9, 35.9. **^{11}B NMR** (128.4 MHz, CDCl$_3$, 300 K): δ (ppm) = -19.31 (t, *J* = 94.2 Hz). **HRMS** (ESI) m/z: [M+H]$^+$ Calcd for $C_{11}H_{19}BN_5O_2^+$: 264.1626; Found: 264.1626.

(3-benzoyl-4-methyl-1H-pyrazol-1-yl)(1,3-dimethyl-1H-imidazol-3-ium-2-yl)dihydroborate (14)

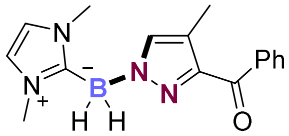

According to ***GP*** with **1a** (33.0 mg, 0.3 mmol, 1.0 equiv.), **2l** (167 mg, 0.9 mmol, 3.0 equiv.), Ir(ppy)$_2$(dtbbpy)PF$_6$ (5.4 mg, 0.006 mmol, 0.02 equiv.), DABCO (33.6 mg, 0.3 mmol, 1.0 equiv.), in 1.0 mL MeCN for 2 h. Purification by silica gel chromatography afforded the desired **14** and as a white solid in 53% yield (46.7 mg). **^{1}H NMR** (400 MHz, CDCl$_3$, 300 K): δ (ppm) = 8.14 (s, 1H), 8.12 (s, 1H), 7.47 – 7.37 – 7.32 (m 1H), 7.35 (m, 3H), 6.84 (s, 2H), 3.74 (s, 6H), 2.34 (s, 3H). **^{13}C NMR** (100 MHz, CDCl$_3$, 300 K): δ (ppm) = 190.0, 148.8, 139.4, 136.1, 131.2, 130.7, 127.4, 121.0, 120.6, 36.1, 10.3. **^{11}B NMR** (128.4 MHz, CDCl$_3$, 300 K): δ (ppm) = -16.43 – -22.55 (m). **HRMS** (ESI) m/z: [M+H]$^+$ Calcd for $C_{16}H_{20}BN_4O^+$: 295.1725; Found: 295.1727.

(3-benzoyl-4-methyl-1H-pyrazol-1-yl)(1,3-dimethyl-1H-imidazol-3-ium-2-yl)dihydroborate (15)

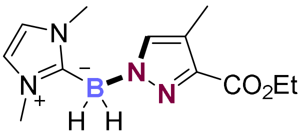

According to ***GP*** with **1a** (33.0 mg, 0.3 mmol, 1.0 equiv.), **2m** (139 mg, 0.9 mmol, 3.0 equiv.), Ir(ppy)$_2$(dtbbpy)PF$_6$ (5.4 mg, 0.006 mmol, 0.02 equiv.), DABCO (33.6 mg, 0.3 mmol, 1.0 equiv.), in 1.0 mL MeCN for 3 h. Purification

by silica gel chromatography afforded the desired **15** and as a white solid in 54% yield (42.4 mg). **^{1}H NMR** (400 MHz, $CDCl_3$, 300 K): δ (ppm) = 7.30 (s, 1H), 6.82 (s, 2H), 4.31 (q, *J* = 7.0 Hz, 2H), 3.63 (s, 6H), 2.25 (s, 3H), 1.33 (t, *J* = 7.1 Hz, 3H). **^{13}C NMR** (100 MHz, $CDCl_3$, 300 K): δ (ppm) = 164.2, 141.6, 136.7, 121.1, 119.6, 59.7, 35.9, 14.4, 10.0. **^{11}B NMR** (128.4 MHz, $CDCl_3$, 300 K): δ (ppm) = -19.14 (t, *J* = 93.4 Hz). **HRMS** (ESI) m/z: $[M+H]^+$ Calcd for $C_{12}H_{20}BN_4O_2^+$: 263.1674; Found: 263.1674.

(4-cyclopropyl-3-(ethoxycarbonyl)-1H-pyrazol-1-yl)(1,3-dimethyl-1H-imidazol-3-ium-2-yl)dihydroborate (16)

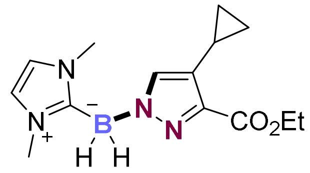

According to ***GP*** with **1a** (33.0 mg, 0.3 mmol, 1.0 equiv.), **2n** (162 mg, 0.9 mmol, 3.0 equiv.), $Ir(ppy)_2(dtbbpy)PF_6$ (5.4 mg, 0.006 mmol, 0.02 equiv.), DABCO (33.6 mg, 0.3 mmol, 1.0 equiv.), in 1.0 mL MeCN for 2 h. Purification by silica gel chromatography afforded the desired **16** and as a white solid in 87% yield (75 mg). **^{1}H NMR** (400 MHz, $CDCl_3$, 300 K): δ (ppm) = 7.07 (s, 1H), 6.82 (s, 2H), 4.31 (q, *J* = 7.0 Hz, 2H), 3.59 (s, 6H), 2.26 – 2.16 (m, 1H), 1.32 (t, *J* = 7.0 Hz, 3H), 0.85 (d, *J* = 8.2 Hz, 2H), 0.45 (d, *J* = 5.0 Hz, 2H). **^{13}C NMR** (100 MHz, $CDCl_3$, 300 K): δ (ppm) = 164.1, 142.0, 133.1, 127.8, 121.1, 59.7, 35.8, 14.4, 8.4, 6.0. **^{11}B NMR** (128.4 MHz, $CDCl_3$, 300 K): δ (ppm) = -17.79 – -20.37 (m). **HRMS** (ESI) m/z: $[M+H]^+$ Calcd for $C_{14}H_{22}BN_4O_2^+$: 289.1830; Found: 289.1830.

(1,3-dimethyl-1H-imidazol-3-ium-2-yl)(3-(ethoxycarbonyl)-5-ethyl-1H-pyrazol-1-yl)dihydroborate (17)

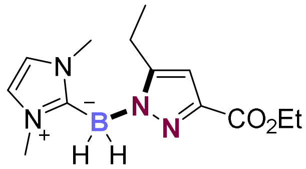

According to ***GP*** with **1a** (33.0 mg, 0.3 mmol, 1.0 equiv.), **2p** (151 mg, 0.9 mmol, 3.0 equiv.), $Ir(ppy)_2(dtbbpy)PF_6$ (5.4 mg, 0.006 mmol, 0.02 equiv.), DABCO (33.6 mg, 0.3 mmol, 1.0 equiv.), in 1.0 mL MeCN for 4 h. Purification by silica gel chromatography afforded the desired **17** and as a colorless oil in 62% yield (51 mg). **^{1}H NMR** (400 MHz, $CDCl_3$, 300 K): δ (ppm) = 6.84 (s, 2H), 6.52 (s, 1H), 4.28 (q, *J* = 7.1 Hz, 2H), 3.55 (s, 6H), 2.68 (q, *J* = 7.5 Hz, 2H), 1.31 (t, *J* = 7.1 Hz, 3H), 1.21 (t, *J* = 7.5 Hz, 3H). **^{13}C NMR** (100 MHz, $CDCl_3$, 300 K): δ (ppm) = 163.8, 150.5, 142.8, 121.1, 105.7, 59.8, 35.7, 20.3, 14.4, 13.1. **^{11}B NMR** (128.4 MHz, $CDCl_3$, 300 K): δ (ppm) = -20.78 (t, *J* = 40.3 Hz). **HRMS** (ESI) m/z: $[M+H]^+$ Calcd for $C_{13}H_{22}BN_4O_2^+$: 277.1830; Found: 277.1823.

(1,3-dimethyl-1H-imidazol-3-ium-2-yl)(5-((dimethylamino)methyl)-3-(ethoxycarbonyl)-1H-pyrazol-1-yl)dihydroborate (18)

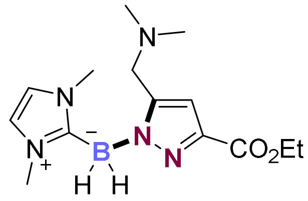

According to ***GP*** with **1a** (33.0 mg, 0.3 mmol, 1.0 equiv.), **2q** (177 mg, 0.9 mmol, 3.0 equiv.), $Ir(ppy)_2(dtbbpy)PF_6$ (5.4 mg, 0.006 mmol, 0.02 equiv.), DABCO (33.6 mg, 0.3 mmol, 1.0 equiv.), in 1.0 mL MeCN for 5 h. Purification by silica gel chromatography afforded the desired **18** and as a white solid in 50% yield (45.7 mg). **^{1}H NMR** (400 MHz, $CDCl_3$, 300 K): δ (ppm) = 6.83 (s, 2H), 6.66 (s,

1H), 4.28 (q, J = 7.1 Hz, 2H), 3.59 (s, 6H), 3.57 (s, 2H), 2.23 (s, 6H), 1.31 (t, J = 7.1 Hz, 3H). **^{13}C NMR** (100 MHz, $CDCl_3$, 300 K): δ (ppm) = 163.7, 145.1, 142.9, 121.0, 108.8, 59.9, 55.1, 45.1, 35.8, 14.4. **^{11}B NMR** (128.4 MHz, $CDCl_3$, 300 K): δ (ppm) = -19.18 – -21.70 (m). **HRMS** (ESI) m/z: $[M+H]^+$ Calcd for $C_{14}H_{25}BN_5O_2^+$: 306.2096; Found: 306.2095.

(5-(1-(tert-butoxycarbonyl)piperidin-4-yl)-3-(ethoxycarbonyl)-1H-pyrazol-1-yl)(1,3-dimethyl-1H-imidazol-3-ium-2-yl)dihydroborate (19)

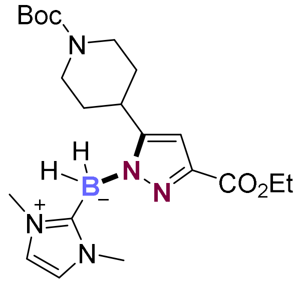

According to ***GP*** with **1a** (33.0 mg, 0.3 mmol, 1.0 equiv.), **2r** (201 mg, 0.9 mmol, 3.0 equiv.), $Ir(ppy)_2(dtbbpy)PF_6$ (5.4 mg, 0.006 mmol, 0.02 equiv.), DABCO (33.6 mg, 0.3 mmol, 1.0 equiv.), in 1.0 mL MeCN for 4 h. Purification by silica gel chromatography afforded the desired **19** and as a white solid in 62% yield (80 mg). **^{1}H NMR** (400 MHz, $CDCl_3$, 300 K): δ (ppm) = 6.86 (s, 2H), 6.51 (s, 1H), 4.28 (q, J = 7.1 Hz, 2H), 4.18 (s, 2H), 3.56 (s, 6H), 3.11 – 3.03 (m, 1H), 2.85 – 2.74 (m, 2H), 1.96 – 1.90 (m, 2H), 1.57 – 1.48 (m, 2H), 1.46 (s, 9H), 1.31 (t, J = 7.1 Hz, 3H). **^{13}C NMR** (100 MHz, $CDCl_3$, 300 K): δ (ppm) = 163.6, 154.8, 152.4, 143.0, 121.1, 104.6, 79.4, 59.9, 35.8, 34.4, 32.0, 28.4, 14.4. **^{11}B NMR** (128.4 MHz, $CDCl_3$, 300 K): δ (ppm) = -19.63 – -21.55 (m). **HRMS** (APCI) m/z: $[M+H]^+$ Calcd for $C_{21}H_{35}BN_5O_4^+$: 432.2777; Found: 432.2775.

(5-((tert-butyldimethylsilyl)oxy)-3-(methoxycarbonyl)-1H-pyrazol-1-yl)(1,3-dimethyl-1H-imidazol-3-ium-2-yl)dihydroborate (20)

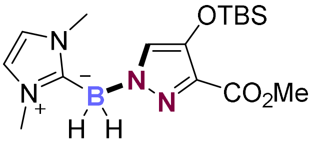

According to ***GP*** with **1a** (33.0 mg, 0.3 mmol, 1.0 equiv.), **2o** (230 mg, 0.9 mmol, 3.0 equiv.), $Ir(ppy)_2(dtbbpy)PF_6$ (5.4 mg, 0.006 mmol, 0.02 equiv.), DABCO (33.6 mg, 0.3 mmol, 1.0 equiv.), in 1.0 mL MeCN for 2 h. Purification by silica gel chromatography afforded the desired **20** and as a white solid in 70% yield (55 mg). **^{1}H NMR** (400 MHz, $CDCl_3$, 300 K): δ (ppm) = 7.14 (s, 1H), 6.82 (s, 2H), 3.79 (s, 3H), 3.58 (s, 6H), 0.98 (s, 9H), 0.13 (s, 6H). **^{13}C NMR** (100 MHz, $CDCl_3$, 300 K): δ (ppm) = 163.2, 141.8, 133.3, 126.5, 121.1, 50.8, 35.8, 25.7, 18.2, -5.1. **^{11}B NMR** (128.4 MHz, $CDCl_3$, 300 K): δ (ppm) = δ -17.45 – -19.87 (m). **HRMS** (ESI) m/z: $[M+H]^+$ Calcd for $C_{16}H_{30}0BN_4O_3Si^+$: 365.2175; Found: 365.2170.

(1,3-dimethyl-1H-imidazol-3-ium-2-yl)(3-(ethoxycarbonyl)-5-(4-fluorophenyl)-1H-pyrazol-1-yl)dihydroborate (21)

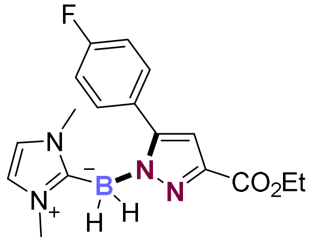

According to ***GP*** with **1a** (33.0 mg, 0.3 mmol, 1.0 equiv.), **2s** (210 mg, 0.9 mmol, 3.0 equiv.) in three times with an hour interval, Ir(ppy)$_2$(dtbbpy)PF$_6$ (5.4 mg, 0.006 mmol, 0.02 equiv.), DABCO (33.6 mg, 0.3 mmol, 1.0 equiv.), in 1.0 mL EtOH for 3 h. Purification by silica gel chromatography afforded the desired **11** and as a white solid in 60% yield (60 mg). **^{1}H NMR** (400 MHz, CDCl$_3$, 300 K): δ (ppm) = δ 7.48 – 7.42 (m, 2H), 7.07 – 7.01 (m, 2H), 6.78 (s, 2H), 6.75 (s, 1H), 4.33 (q, *J* = 7.1 Hz, 2H), 3.48 (s, 6H), 1.34 (t, *J* = 7.1 Hz, 3H). **^{13}C NMR** (100 MHz, CDCl$_3$, 300 K): δ (ppm) = 163.6, 162.2 (d, *J* = 246.7 Hz), 148.1, 143.3, 131.0 (d, *J* = 8.0 Hz), 129.4 (d, *J* = 3.3 Hz), 120.9, 114.6 (d, *J* = 21.5 Hz), 108.5, 60.1, 35.7, 14.4. **^{11}B NMR** (128.4 MHz, CDCl$_3$, 300 K): δ (ppm) = -19.24 – -20.72 (m). **HRMS** (APCI) m/z: [M+H]$^+$ Calcd for $C_{17}H_{21}BFN_4O_2^+$: 343.1736; Found: 343.1737.

(1,3-dimethyl-1H-imidazol-3-ium-2-yl)(3-(ethoxycarbonyl)-5-(3-fluoro-4-methoxyphenyl)-1H-pyrazol-1-yl)dihydroborate (22)

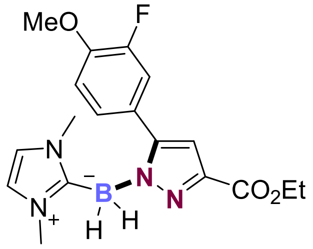

According to ***GP*** with **1a** (33.0 mg, 0.3 mmol, 1.0 equiv.), **2t** (237.6 mg, 0.9 mmol, 3.0 equiv.) in three times with an hour interval, Ir(ppy)$_2$(dtbbpy)PF$_6$ (5.4 mg, 0.006 mmol, 0.02 equiv.), DABCO (33.6 mg, 0.3 mmol, 1.0 equiv.), in 1.0 mL EtOH for 3 h. Purification by silica gel chromatography afforded the desired **22** and as a white solid in 57% yield (64 mg). **^{1}H NMR** (400 MHz, CDCl$_3$, 300 K): δ (ppm) = 7.22 – 7.12 (m, 2H), 6.88 (t, *J* = 8.6 Hz, 1H), 6.73 (s, 2H), 6.67 (s, 1H), 4.24 (q, *J* = 7.2 Hz, 2H), 3.82 (s, 3H), 3.42 (s, 6H), 1.26 (t, *J* = 7.0 Hz, 3H). **^{13}C NMR** (100 MHz, CDCl$_3$, 300 K): δ (ppm) = 163.5, 151.4 (d, *J* = 245.0 Hz), 147.6, 146.9 (d, *J* = 10.7 Hz), 143.2, 126.3 (d, *J* = 7.1 Hz), 125.1 (d, *J* = 3.4 Hz), 120.9, 116.9 (d, *J* = 19.2 Hz), 112.6 (d, *J* = 2.3 Hz), 108.3, 60.0, 56.1, 35.6, 14.3. **^{11}B NMR** (128.4 MHz, CDCl$_3$, 300 K): δ (ppm) = -19.24 – -20.72 (m). **HRMS** (ESI) m/z: [M+H]$^+$ Calcd for $C_{18}H_{23}BFN_4O_3^+$: 373.1842; Found: 373.1847.

(1,3-dimethyl-1H-imidazol-3-ium-2-yl)(3-(ethoxycarbonyl)-4,5,6,7-tetrahydro-1H-indazol-1-yl)dihydroborate(23)

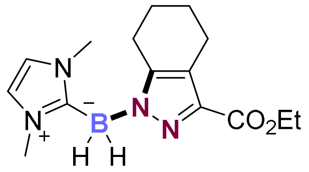

According to ***GP*** with **1a** (33.0 mg, 0.3 mmol, 1.0 equiv.), **2u** (175 mg, 0.9 mmol, 3.0 equiv.), Ir(ppy)$_2$(dtbbpy)PF$_6$ (5.4 mg, 0.006 mmol, 0.02 equiv.), DABCO (33.6 mg, 0.3 mmol, 1.0 equiv.), in 1.0 mL MeCN for 2 h. Purification by silica gel chromatography afforded the desired **23** and as a white solid in 52% yield (47 mg). **^{1}H NMR** (400 MHz, CDCl$_3$, 300 K): δ (ppm) = 6.84 (s, 2H), 4.28 (q, *J* = 7.1 Hz, 2H), 3.58 (s, 6H), 2.73 - 2.68 (m Hz, 2H), 2.63 – 2.56(m, 2H), 1.79 – 1.71 (m, 2H), 1.71 – 1.64 (m, 2H), 1.31 (t, *J* = 7.1 Hz, 3H). **^{13}C NMR** (100 MHz, CDCl$_3$, 300 K): δ (ppm) = 164.3, 144.3, 139.6, 121.1, 119.1, 59.5, 35.8, 23.3, 23.2, 23.1, 22.2, 14.4. **^{11}B NMR** (128.4 MHz, CDCl$_3$, 300 K): δ (ppm) = -21.10 (t, *J* = 98.8 Hz). **HRMS** (ESI) m/z: [M+H]$^+$ Calcd for

$C_{15}H_{24}BN_4O_2^+$: 303.1987; Found: 303.1987.

(3-((((3S,8S,9S,10R,13R,14S,17R)-10,13-dimethyl-17-((R)-6-methylheptan-2-yl)-2,3,4,7,8,9,10,11,12,13,14,15,16,17-tetradecahydro-1H-cyclopenta[a]phenanthren-3-yl)oxy)carbonyl)-1H-pyrazol-1-yl)(1,3-dimethyl-1H-imidazol-3-ium-2-yl)dihydroborate (24)

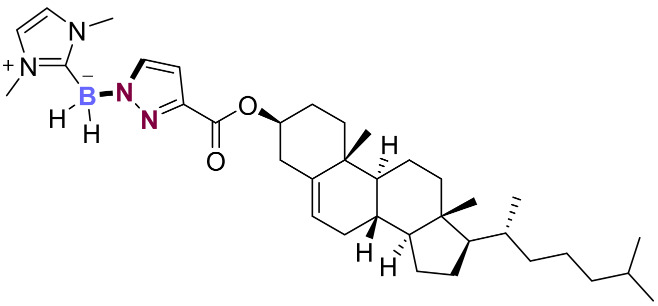

According to ***GP*** with **1a** (33.0 mg, 0.3 mmol, 1.0 equiv.), **2v** (432 mg, 0.9 mmol, 3.0 equiv.), Ir(ppy)$_2$(dtbbpy)PF$_6$ (5.4 mg, 0.006 mmol, 0.02 equiv.), DABCO (33.6 mg, 0.3 mmol, 1.0 equiv.), in 2.0 mL EtOH for 2 h. Purification by silica gel chromatography afforded the desired **24** and as a yellow oil in 62% yield (109 mg). **^{1}H NMR** (400 MHz, $CDCl_3$, 300 K): δ (ppm) = 7.50 (d, *J* = 2.1 Hz, 1H), 6.84 (s, 2H), 6.69 (d, *J* = 2.1 Hz, 1H), 5.36 (d, *J* = 4.1 Hz, 1H), 4.87 – 4.77 (m, 1H), 3.65 (s, 6H), 2.43 (d, *J* = 8.0 Hz, 2H), 2.05 – 1.06 (m, 29H), 1.02 (s, 3H), 0.91 (d, *J* = 6.5 Hz, 3H), 0.87 (d, *J* = 1.8 Hz, 3H), 0.85 (d, *J* = 1.8 Hz, 3H), 0.67 (s, 3H). **^{13}C NMR** (100 MHz, $CDCl_3$, 300 K): δ (ppm) = 163.0, 144.7, 140.0, 136.5, 122.3, 121.1, 107.7, 56.7, 56.1, 50.0, 42.3, 39.7, 39.5, 38.2, 37.1, 36.6, 36.1, 36.0, 35.7, 31.9, 31.8, 28.2, 28.0, 27.8, 24.2, 23.8, 22.8, 22.5, 21.0, 19.3, 18.7, 11.8. **^{11}B NMR** (128.4 MHz, $CDCl_3$, 300 K): δ (ppm) = -17.79 – -20.21 (m). **HRMS** (ESI) m/z: $[M+H]^+$ Calcd for $C_{36}H_{58}BN_4O_2^+$: 589.4647; Found: 589.4642.

(1,3-dimethyl-1H-imidazol-3-ium-2-yl)(3-(((3,7-dimethyloctyl)oxy)carbonyl)-1H-pyrazol-1-yl)dihydroborate (25)

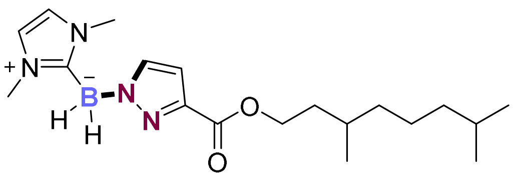

According to ***GP*** with **1a** (33.0 mg, 0.3 mmol, 1.0 equiv.), **2w** (227 mg, 0.9 mmol, 3.0 equiv.), Ir(ppy)$_2$(dtbbpy)PF$_6$ (5.4 mg, 0.006 mmol, 0.02 equiv.), DABCO (33.6 mg, 0.3 mmol, 1.0 equiv.), in 1.0 mL MeCN for 4 h. Purification by silica gel chromatography afforded the desired **25** and as a yellow oil in 64% yield (69 mg). **^{1}H NMR** (400 MHz, $CDCl_3$, 300 K): δ (ppm) = 7.52 (d, *J* = 2.0 Hz, 1H), 6.84 (s, 2H), 6.70 (d, *J* = 2.0 Hz, 1H), 4.29 (t, *J* = 6.6 Hz, 2H), 3.67 (s, 6H), 1.79 – 1.73 (m, 2H), 1.56 – 1.51 (m, 2H), 1.32 – 1.27 (m, 3H), 1.16 – 1.12 (m, 3H), 0.92 (d, *J* = 6.3 Hz, 3H), 0.86 (d, *J* = 6.6 Hz, 6H). **^{13}C NMR** (100 MHz, $CDCl_3$, 300 K): δ (ppm) = 163.7, 144.4, 136.5, 121.1, 107.7, 62.8, 39.2, 37.2, 36.0, 35.6, 30.0, 27.9, 24.6, 22.6, 22.6, 19.6. **^{11}B NMR** (128.4 MHz, $CDCl_3$, 300 K): δ (ppm) = δ -18.51 – -19.63 (m). **HRMS** (ESI) m/z: $[M+H]^+$ Calcd for $C_{19}H_{34}BN_4O_2^+$: 361.2769; Found: 361.2769.

(1,3-dimethyl-1H-imidazol-3-ium-2-yl)(3-((((3S,8S,9S,10R,13R,14S,17R)-17-((2R,5S,E)-5-ethyl-6-methylhept-3-en-2-yl)-10,13-dimethyl-2,3,4,7,8,9,10,11,12,13,14,15,16,17-tetradecahydro-1H-cyclopenta[a]phenanthren-3-yl)oxy)carbonyl)-1H-pyrazol-1-yl)dihydroborate (26)

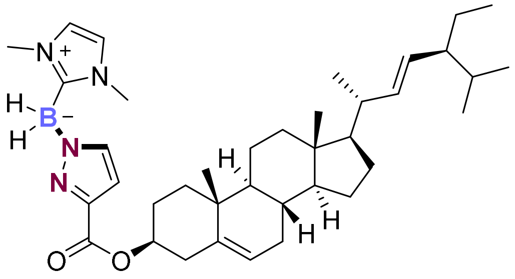

According to ***GP*** with **1a** (33.0 mg, 0.3 mmol, 1.0 equiv.), **2x** (455 mg, 0.9 mmol, 3.0 equiv.), Ir(ppy)$_2$(dtbbpy)PF$_6$ (5.4 mg, 0.006 mmol, 0.02 equiv.), DABCO (33.6 mg, 0.3 mmol, 1.0 equiv.), in 1.0 mL MeCN and 1.0 mL CH_2Cl_2 for 2 h. Purification by silica gel chromatography afforded the desired **26** and as a yellow oil in 64% yield (118 mg). **^{1}H NMR** (400 MHz, $CDCl_3$, 300 K): δ (ppm) = δ 7.49 (d, *J* = 1.9 Hz, 1H), 6.85 (s, 2H), 6.68 (d, *J* = 1.9 Hz, 1H), 5.36 (d, *J* = 3.8 Hz, 1H), 5.15 (dd, *J* = 15.1, 8.5 Hz, 1H), 5.01 (dd, *J* = 15.1, 8.6 Hz, 1H), 4.85 – 4.74 (m, 1H), 3.64 (s, 6H), 2.42 (d, *J* = 7.5 Hz, 2H), 2.05 – 1.90 (m, 5H), 1.89 – 1.82 (m, 1H), 1.73 – 1.64 (m, 2H), 1.56 – 1.46 (m, 7H), 1.44 – 1.38 (m, 2H), 1.28 – 1.22(m, 2H), 1.19 – 1.12 (m, 4H), 1.02 (s, 5H), 1.00 (s, 2H), 0.84 (s, 2H), 0.83 (s, 2H), 0.81 (s, 1H), 0.79 (s, 3H), 0.78 (s, 2H), 0.69 (s, 3H). **^{13}C NMR** (100 MHz, $CDCl_3$, 300 K): δ (ppm) = 163.1, 144.7, 140.0, 138.3, 136.5, 129.2, 122.3, 121.1, 107.7, 73.6, 56.8, 55.9, 51.2, 50.0, 42.2, 40.5, 39.6, 38.2, 37.1, 36.6, 35.9, 31.8, 28.9, 27.8, 25.4, 24.3, 21.2, 21.0, 21.0, 19.3, 18.9, 12.2, 12.0. **^{11}B NMR** (128.4 MHz, $CDCl_3$, 300 K): δ (ppm) = -18.02 – -20.03 (m). **HRMS** (ESI) m/z: $[M+H]^+$ Calcd for $C_{38}H_{60}BN_4O_2^+$: 615.4804; Found: 615.4804.

(3-((((3aR,5R,6S,6aR)-5-(2,2-dimethyl-1,3-dioxolan-4-yl)-2,2-dimethyltetrahydrofuro[2,3-d][1,3]dioxol-6-yl)oxy)carbonyl)-1H-pyrazol-1-yl)(1,3-dimethyl-1H-imidazol-3-ium-2-yl)dihydroborate (27)

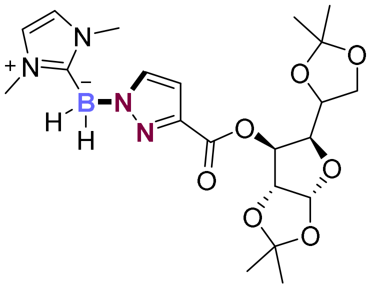

According to ***GP*** with **1a** (33.0 mg, 0.3 mmol, 1.0 equiv.), **2y** (471 mg, 0.9 mmol, 3.0 equiv.), Ir(ppy)$_2$(dtbbpy)PF$_6$ (5.4 mg, 0.006 mmol, 0.02 equiv.), DABCO (33.6 mg, 0.3 mmol, 1.0 equiv.), in 1.0 mL MeCN for 5 h. Purification by silica gel chromatography afforded the desired **27** and as a yellow oil in 53% yield (69 mg). **^{1}H NMR** (400 MHz, $CDCl_3$, 300 K): δ (ppm) = 7.50 (d, *J* = 2.0 Hz, 1H), 6.88 (s, 2H), 6.66 (d, *J* = 2.0 Hz, 1H), 5.89 (d, *J* = 3.6 Hz, 1H), 5.37 (d, *J* = 2.8 Hz, 1H), 4.58 (d, *J* = 3.6 Hz, 1H), 4.42 – 4.38 (m, 1H), 4.30 (q, J = 6.0 Hz, 1H), 4.04 (d, *J* = 6.3 Hz, 2H), 3.70 (s, 6H), 1.50 (s, 3H), 1.38 (s, 3H), 1.26 (s, 3H), 1.24 (s, 3H). **^{13}C NMR** (100 MHz, $CDCl_3$, 300 K): δ (ppm) = 161.9, 142.8, 136.3, 121.1, 111.9, 108.6, 107.9, 105.0, 83.2, 79.5, 75.6, 72.9, 66.2, 35.9, 26.6, 26.5, 26.0, 25.1. **^{11}B NMR** (128.4 MHz, $CDCl_3$, 300 K): δ (ppm) = -18.83 – -20.06 (m). **HRMS** (ESI) m/z: $[M+H]^+$ Calcd for $C_{21}H_{32}BN_4O_7^+$: 463.2359; Found: 463.2353.

(1,3-dimethyl-1H-imidazol-3-ium-2-yl)(3-((((R)-2,5,7,8-tetramethyl-2-((4S,8S)-4,8,12-trimethyltridecyl)chroman-6-yl)oxy)carbonyl)-1H-pyrazol-1-yl)dihydroborate (28)

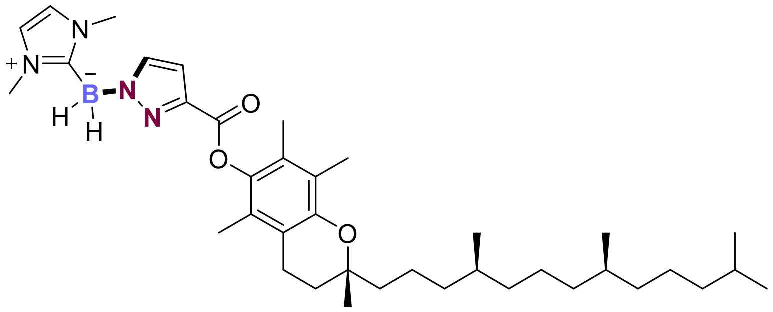

According to ***GP*** with **1a** (33.0 mg, 0.3 mmol, 1.0 equiv.), **2z** (432 mg, 0.6 mmol, 2.0 equiv.), Ir(ppy)$_2$(dtbbpy)PF$_6$ (5.4 mg, 0.006 mmol, 0.02 equiv.), DABCO (33.6 mg, 0.3 mmol, 1.0 equiv.), in 1.0 mL MeCN for 4 h. Purification by silica gel chromatography afforded the desired **28** and as a yellow oil in 40% yield (76 mg). **^{1}H NMR** (400 MHz, CDCl$_3$, 300 K): δ (ppm) = δ 7.60 (d, J = 2.0 Hz, 1H), 6.90 (d, J = 1.3 Hz, 1H), 6.86 (s, 2H), 3.69 (s, 6H), 2.58 (t, J = 6.8 Hz, 2H), 2.08 (s, 3H), 2.05 (s, 3H), 2.01 (s, 3H), 1.84 – 1.71 (m, 2H), 1.59 – 1.48 (m, 3H), 1.42 – 1.35 (m, 3H), 1.29 – 1.22 (d, J = 9.5 Hz, 10H), 1.17 – 1.12 (m, 3H), 1.11 – 1.03 (m, 4H), 0.88 (s, 4H), 0.86 (s, 7H), 0.84 (s, 2H). **^{13}C NMR** (100 MHz, CDCl$_3$, 300 K): δ (ppm) = 161.9, 149.1, 143.4, 140.5, 136.6, 127.2, 125.4, 122.7, 121.2, 117.2, 108.5, 74.9, 39.3, 37.5, 37.4, 37.3, 37.2, 36.0, 32.7, 32.7, 27.9, 24.8, 24.4, 22.7, 22.6, 21.0, 20.6, 19.7, 19.6, 13.1, 12.2, 11.8. **^{11}B NMR** (128.4 MHz, CDCl$_3$, 300 K): δ (ppm) = -18.17 – -20.03 (m). **HRMS** (ESI) m/z: [M+H]$^+$ Calcd for $C_{38}H_{62}BN_4O_3^+$: 633.4909; Found: 633.4910

(1,3-dimethyl-1H-imidazol-3-ium-2-yl)(3-(((R)-2-(6-methoxyquinolin-4-yl)-2-((1S,2R,4S,5R)-5-vinylquinuclidin-2-yl)ethoxy)carbonyl)-1H-pyrazol-1-yl)dihydroborate (29)

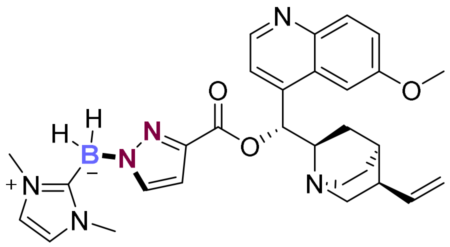

According to ***GP*** with **1a** (33.0 mg, 0.3 mmol, 1.0 equiv.), **2aa** (376 mg, 0.9 mmol, 3.0 equiv.), Ir(ppy)$_2$(dtbbpy)PF$_6$ (5.4 mg, 0.006 mmol, 0.02 equiv.), DABCO (33.6 mg, 0.3 mmol, 1.0 equiv.), in 1.0 mL MeCN for 4 h. Purification by silica gel chromatography afforded the desired **29** and as a yellow oil in 70% yield (110 mg). **^{1}H NMR** (400 MHz, CDCl$_3$, 300 K): δ (ppm) = 8.65 (d, J = 4.3 Hz, 1H), 7.96 (d, J = 9.3 Hz, 1H), 7.52 (s, 1H), 7.50 (s, 1H), 7.41 (d, J = 4.3 Hz, 1H), 7.32 (d, J = 9.2 Hz, 1H), 6.79 (s, 2H), 6.76 (s, 1H), 6.69 (d, J = 5.4 Hz, 1H),5.83 - 5.73 (m, 1H), 5.02 – 4.93 (m, 2H), 3.91 (s, 3H), 3.65 (s, 6H), 3.48 – 3.40 (m, 1H), 3.32 – 3.22(m, 1H), 3.14 – 3.04 (m, 2H), 2.72 – 2.61 (m, 2H), 2.33 – 2.24 (m, 2H), 1.87 – 1.74 (m, 4H), 1.56 – 1.45 (m, 1H).**^{13}C NMR** (100 MHz, CDCl$_3$, 300 K): δ (ppm) = 162.4, 158.0, 147.5, 144.8, 144.0, 143.5, 141.6, 136.6, 131.7, 129.5, 127.0, 126.5, 121.9, 121.2, 119.0, 114.7, 108.0, 101.7, 73.5, 59.4, 56.8, 55.8, 42.8, 39.7, 36.1, 27.8, 27.5, 23.6. **^{11}B NMR** (128.4 MHz, CDCl$_3$, 300 K): δ (ppm) = -17.99 – -20.84 (m). **HRMS** (ESI) m/z: [M+H]$^+$ Calcd for $C_{29}H_{36}BN_6O_3^+$: 527.2936; Found: 527.2935.

(1-benzyl-3-methyl-1H-imidazol-3-ium-2-yl)(3-(ethoxycarbonyl)-1H-pyrazol-1-yl)dihydroborate (30)

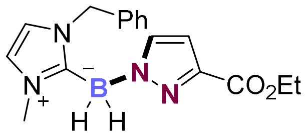

According to ***GP*** with **1b** (55.8 mg, 0.3 mmol, 1.0 equiv.), **2a** (126 mg, 0.9 mmol, 3.0 equiv.), Ir(ppy)2(dtbbpy)PF6 (5.4 mg, 0.006 mmol, 0.02 equiv.), DABCO (33.6 mg, 0.3 mmol, 1.0 equiv.), in 1.0 mL MeCN for 4 h. Purification by silica gel chromatography afforded the desired **30** and as a white solid in 85% yield (82.6 mg). **^{1}H NMR** (400 MHz, $CDCl_3$, 300 K): δ (ppm) = 7.49 (d, *J* = 2.2 Hz, 1H), 7.35 – 7.29 (m, 3H), 7.23 – 7.19 (m, 2H), 6.84 (d, *J* = 1.7 Hz, 1H), 6.73 (d, *J* = 1.7 Hz, 1H), 6.70 (d, *J* = 2.0 Hz, 1H), 5.28 (s, 2H), 4.32 (q, *J* = 7.1 Hz, 2H), 3.68 (s, 3H), 1.34 (t, *J* = 7.1 Hz, 3H).**^{13}C NMR** (100 MHz, $CDCl_3$, 300 K): δ (ppm) = 163.6, 144.4, 136.5, 135.2, 128.9, 128.4, 128.3, 121.7, 119.6, 107.8, 60.0, 52.2, 36.0, 14.4. **^{11}B NMR** (128.4 MHz, $CDCl_3$, 300 K): δ (ppm) = δ -18.45 – -19.91 (m). **HRMS** (APCI) m/z: $[M+H]^+$ Calcd for $C_{17}H_{22}BN_4O_2^+$: 325.1830; Found: 325.1830.

(1-butyl-3-methyl-1H-imidazol-3-ium-2-yl)(3-(ethoxycarbonyl)-1H-pyrazol-1-yl)dihydroborate (31)

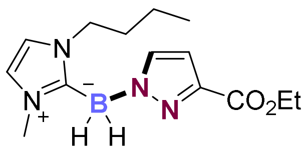

According to ***GP*** with **1c** (45.6 mg, 0.3 mmol, 1.0 equiv.), **2a** (126 mg, 0.9 mmol, 3.0 equiv.), Ir(ppy)2(dtbbpy)PF6 (5.4 mg, 0.006 mmol, 0.02 equiv.), DABCO (33.6 mg, 0.3 mmol, 1.0 equiv.), in 1.0 mL MeCN for 4 h. Purification by silica gel chromatography afforded the desired **31** and as a white solid in 63% yield (55 mg). **^{1}H NMR** (400 MHz, $CDCl_3$, 300 K): δ (ppm) = 7.49 (d, *J* = 2.1 Hz, 1H), 6.89 – 6.82 (m, 2H), 6.71 (d, *J* = 2.2 Hz, 1H), 4.32 (q, *J* = 7.1 Hz, 2H), 4.05 – 4.00 (m, 2H), 3.65 (s, 3H), 1.68 – 1.59 (m, 2H), 1.33 (t, *J* = 7.1 Hz, 3H), 1.30 – 1.23 (m, 2H), 0.89 (t, *J* = 7.4 Hz, 3H). **^{13}C NMR** (100 MHz, $CDCl_3$, 300 K): δ (ppm) = 163.7, 144.4, 136.5, 121.3, 119.7, 107.8, 60.0, 48.7, 36.0, 32.5, 19.6, 14.4, 13.5. **^{11}B NMR** (128.4 MHz, $CDCl_3$, 300 K): δ (ppm) = -19.10 (t, *J* = 84.1 Hz). **HRMS** (ESI) m/z: $[M+H]^+$ Calcd for $C_{14}H_{24}BN_4O_2^+$: 291.1987; Found: 291.1988.

(1-allyl-3-methyl-1H-imidazol-3-ium-2-yl)(3-(ethoxycarbonyl)-1H-pyrazol-1-yl)dihydroborate (32)

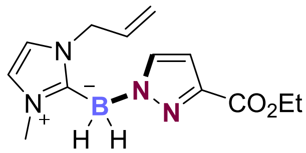

According to ***GP*** with **1d** (40.8 mg, 0.3 mmol, 1.0 equiv.), **2a** (126 mg, 0.9 mmol, 3.0 equiv.), Ir(ppy)2(dtbbpy)PF6 (5.4 mg, 0.006 mmol, 0.02 equiv.), DABCO (33.6 mg, 0.3 mmol, 1.0 equiv.), in 1.0 mL MeCN for 4 h. Purification by silica gel chromatography afforded the desired **32** and as a white solid in 85% yield (70 mg). **^{1}H NMR** (400 MHz, $CDCl_3$, 300 K): δ (ppm) = 7.48 (d, *J* = 2.1 Hz, 1H), 6.88 (d, *J* = 2.2 Hz, 2H), 6.70 (d, *J* = 2.1 Hz, 1H), 5.94 – 5.93 (m, 1H), 5.29 – 5.13 (m, 2H), 4.69 (d, *J* = 6.0 Hz, 2H), 4.35 – 4.29 (m, 2H), 3.67 (s, 3H), 1.33 (t, *J* = 7.1 Hz, 3H). **^{13}C NMR** (100 MHz, $CDCl_3$, 300 K): δ (ppm) = 163.6, 144.4, 136.5, 132.1, 121.5, 119.5, 119.5, 107.8, 60.0, 51.2, 36.0, 14.4. **^{11}B NMR** (128.4 MHz, $CDCl_3$, 300 K): δ (ppm) = -18.26 – -19.96 (m). **HRMS** (ESI) m/z: $[M+H]^+$ Calcd for $C_{13}H_{20}BN_4O_2^+$: 275.1674 ; Found: 275.1688.

(3-(ethoxycarbonyl)-1H-pyrazol-1-yl)(1-isopropyl-3-methyl-1H-imidazol-3-ium-2-yl)dihydroborate (33)

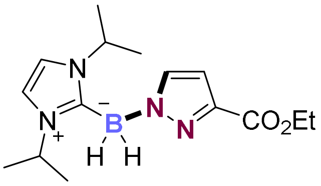

According to ***GP*** with **1e** (49.8 mg, 0.3 mmol, 1.0 equiv.), **2a** (126 mg, 0.9 mmol, 3.0 equiv.), Ir(ppy)2(dtbbpy)PF6 (5.4 mg, 0.006 mmol, 0.02 equiv.), DABCO (33.6 mg, 0.3 mmol, 1.0 equiv.), in 1.0 mL MeCN for 3 h. Purification by silica gel chromatography afforded the desired **33** and as a white solid in 40% yield (36 mg). **^{1}H NMR** (400 MHz, $CDCl_3$, 300 K): δ (ppm) = 7.45 (d, *J* = 2.1 Hz, 1H), 7.01 (s, 2H), 6.69 (d, *J* = 2.1 Hz, 1H), 5.28 – 5.20 (m, 2H), 4.30 (q, *J* = 7.1 Hz, 2H), 1.38 (s, 6H), 1.36 (s, 6H), 1.33 (t, *J* = 7.1 Hz, 3H). **^{13}C NMR** (100 MHz, $CDCl_3$, 300 K): δ (ppm) = 163.7, 144.0, 135.8, 116.2, 107.7, 59.9, 49.9, 23.1, 14.4. **^{11}B NMR** (128.4 MHz, $CDCl_3$, 300 K): δ (ppm) = -18.86 – -20.39 (m). **HRMS** (ESI) m/z: $[M+H]^+$ Calcd for $C_{15}H_{26}BN_4O_2^+$: 305.2143 ; Found: 305.2142.

(4,5-dichloro-1,3-dimethyl-1H-imidazol-3-ium-2-yl)(3-(ethoxycarbonyl)-1H-pyrazol-1-yl)dihydroborate (34)

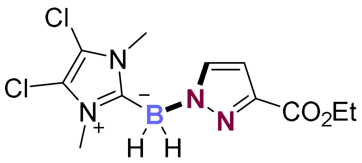

According to ***GP*** with **1f** (53.4 mg, 0.3 mmol, 1.0 equiv.), **2a** (126 mg, 0.9 mmol, 3.0 equiv.), Ir(ppy)2(dtbbpy)PF6 (5.4 mg, 0.006 mmol, 0.02 equiv.), DABCO (33.6 mg, 0.3 mmol, 1.0 equiv.), in 1.0 mL MeCN for 2.5 h. Purification by silica gel chromatography afforded the desired **34** and as a white solid in 63 % yield (59.7 mg). **^{1}H NMR** (400 MHz, $CDCl_3$, 300 K): δ (ppm) = 7.51 (d, *J* = 2.0 Hz, 1H), 6.72 (d, *J* = 2.1 Hz, 1H), 4.32 (q, *J* = 7.1 Hz, 2H), 3.66 (s, 6H), 1.33 (t, *J* = 7.1 Hz, 3H). **^{13}C NMR** (100 MHz, $CDCl_3$, 300 K): δ (ppm) = 163.5, 144.7, 136.6, 117.4, 108.0, 60.2, 33.6, 14.4. **^{11}B NMR** (128.4 MHz, $CDCl_3$, 300 K): δ (ppm) = -18.59 (t, *J* = 98.3 Hz). **HRMS** (ESI) m/z: $[M+H]^+$ Calcd for $C_{11}H1_6BCl_2N_4O_2^+$: 317.0738 ; Found: 317.0736.

(3-(ethoxycarbonyl)-1H-pyrazol-1-yl)((6R)-6-(methoxycarbonyl)-6-(4-methoxyphenyl)-2,4-dimethyl-2,4-diazabicyclo[3.1.0]hex-2-en-2-ium-3-yl)dihydroborate (35)

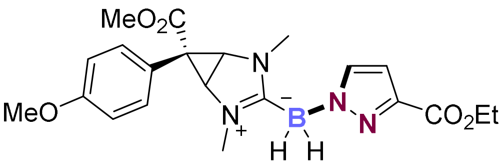

According to ***GP*** with **1g** (86.4 mg, 0.3 mmol, 1.0 equiv.), **2a** (126 mg, 0.9 mmol, 3.0 equiv.), Ir(ppy)2(dtbbpy)PF6 (5.4 mg, 0.006 mmol, 0.02 equiv.), DABCO (33.6 mg, 0.3 mmol, 1.0 equiv.), in 1.0 mL MeCN for 2 h. Purification by silica gel chromatography afforded the desired **35** and as a white solid in 55% yield (70 mg). **^{1}H NMR** (400 MHz, $CDCl_3$, 300 K): δ (ppm) = 6.96 – 6.92 (m, 2H), 6.89 – 6.85 (m, 2H), 6.70 (d, *J* = 2.1 Hz, 1H), 6.58 (d, *J* = 2.1 Hz, 1H), 4.34 (q, *J* = 7.1 Hz, 2H), 4.28 (s, 2H), 3.82 (s, 3H), 3.64 (s, 3H), 3.10 (s, 6H), 1.35 (t, *J* = 7.1 Hz, 3H). **^{13}C NMR** (100 MHz, $CDCl_3$, 300 K): δ (ppm) = 172.3, 163.5, 159.9, 144.2, 136.4, 132.7, 118.9, 114.4, 108.0, 60.1, 55.2, 54.7, 53.0, 35.4, 26.0, 14.5. **^{11}B NMR** (128.4 MHz, $CDCl_3$, 300 K): δ (ppm) = -18.40 – -20.24 (m). **HRMS** (ESI) m/z: $[M+H]^+$ Calcd for $C_{21}H_{28}BN_4O_5^+$: 427.2147 ; Found: 427.2146.

(1,4-dimethyl-1H-1,2,4-triazol-4-ium-5-yl)(3-(ethoxycarbonyl)-1H-pyrazol-1-yl)dihydroborate (36)

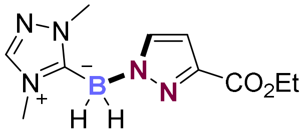

According to ***GP*** with **1h** (33.3 mg, 0.3 mmol, 1.0 equiv.), **2a** (126 mg, 0.9 mmol, 3.0 equiv.), $Ir(ppy)_2(dtbbpy)PF_6$ (5.4 mg, 0.006 mmol, 0.02 equiv.), DABCO (33.6 mg, 0.3 mmol, 1.0 equiv.), in 1.0 mL MeCN for 2.5 h. Purification by silica gel chromatography afforded the desired **36** and as a white solid in 52% yield (38.8 mg). ^{1}**H NMR** (400 MHz, $CDCl_3$, 300 K): δ (ppm) = 8.09 (s, 1H), 7.51 (d, J = 2.1 Hz, 1H), 6.71 (d, J = 2.1 Hz, 1H), 4.30 (q, J = 7.1 Hz, 2H), 3.73 (s, 3H), 3.56 (s, 3H), 1.32 (t, J = 7.1 Hz, 3H). ^{13}**C NMR** (100 MHz, $CDCl_3$, 300 K): δ (ppm) = 163.4, 144.7, 142.3, 137.0, 108.1, 60.2, 38.0, 33.4, 14.3. ^{11}**B NMR** (128.4 MHz, $CDCl_3$, 300 K): δ (ppm) = -18.95 (t, J = 91.5 Hz). **HRMS** (ESI) m/z: $[M+H]^+$ Calcd for $C_{10}H_{17}BN_5O_2^+$: 250.1470 ; Found: 250.1470.

(1,3-dimethyl-1H-benzo[d]imidazol-3-ium-2-yl)(3-(ethoxycarbonyl)-1H-pyrazol-1-yl)dihydroborate (37)

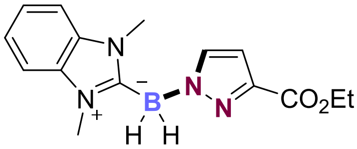

According to ***GP*** with **1i** (48 mg, 0.3 mmol, 1.0 equiv.), **2a** (126 mg, 0.9 mmol, 3.0 equiv.), $Ir(ppy)_2(dtbbpy)PF_6$ (5.4 mg, 0.006 mmol, 0.02 equiv.), DABCO (33.6 mg, 0.3 mmol, 1.0 equiv.), in 1.0 mL MeCN for 3 h. Purification by silica gel chromatography afforded the desired **37** and as a white solid in 52% yield (47.4 mg). ^{1}**H NMR** (400 MHz, $CDCl_3$, 300 K): δ (ppm) = 7.59 (d, J = 2.2 Hz, 1H), 7.49 – 7.40 (m, 4H), 6.76 (d, J = 2.2 Hz, 1H), 4.32 (q, J = 7.1 Hz, 2H), 3.81 (s, 6H), 1.33 (t, J = 7.1 Hz, 3H). ^{13}**C NMR** (100 MHz, $CDCl_3$, 300 K): δ (ppm) = 163.5, 144.7, 136.8, 133.1, 124.7, 111.1, 108.1, 60.1, 32.0, 14.4. ^{11}**B NMR** (128.4 MHz, $CDCl_3$, 300 K): δ (ppm) = -18.51 (t, J = 87.4 Hz). **HRMS** (ESI) m/z: $[M+H]^+$ Calcd for $C_{15}H_{20}BN_4O_2^+$: 299.1647 ; Found: 299.1647.

(3-(ethoxycarbonyl)-1H-pyrazol-1-yl)(2,3,4,6,7,8,9,10-octahydro-1H-pyrido[1,2-a]azepin-5-ium-1-yl)dihydroborate (38)

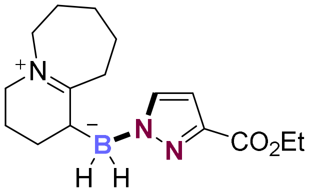

According to ***GP*** with **1j** (49.8 mg, 0.3 mmol, 1.0 equiv.), **2a** (126 mg, 0.9 mmol, 3.0 equiv.), $Ir(ppy)_2(dtbbpy)PF_6$ (5.4 mg, 0.006 mmol, 0.02 equiv.), DABCO (33.6 mg, 0.3 mmol, 1.0 equiv.), in 1.0 mL MeCN for 2 h. Purification by silica gel chromatography afforded the desired **38** and as a white solid in 45% yield (41 mg). ^{1}**H NMR** (400 MHz, $CDCl_3$, 300 K): δ (ppm) = δ 7.45 (d, J = 2.1 Hz, 1H), 6.69 (d, J = 2.1 Hz, 1H), 4.34 (q, J = 7.1 Hz, 2H), 3.47 – 3.44 (m, 2H), 3.41 – 3.37 (m, 2H), 3.33 – 3.28 (m, 2H), 3.14 – 3.09 (m, 2H), 1.89 (m, 3H), 1.73 – 1.65 (m, 2H), 1.66 – 1.57 (m, 4H), 1.35 (t, J = 7.1 Hz, 3H).^{13}**C NMR** (100 MHz, $CDCl_3$, 300 K): δ (ppm) = 167.4, 163.8, 144.1, 135.3, 107.2, 59.9, 53.8, 49.0, 46.9, 30.2, 28.7, 26.9, 23.2, 20.9, 14.3. ^{11}**B NMR** (128.4 MHz, $CDCl_3$, 300 K): δ (ppm) = -6.04 – -8.13 (m). **HRMS** (ESI) m/z: $[M+H]^+$ Calcd for $C_{16}H_{27}BN_3O_2^+$: 305.2143 ; Found: 305.2145.

(1,3-dimethyl-1H-imidazol-3-ium-2-yl)(3-(ethoxycarbonyl)-1H-pyrazol-1-yl)(phenyl)hydroborate (39)

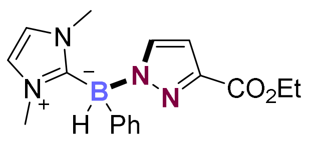

According to ***GP*** with **1k** (55.8 mg, 0.3 mmol, 1.0 equiv.), **2a** (126 mg, 0.9 mmol, 3.0 equiv.), $Ir(ppy)_2(dtbbpy)PF_6$ (5.4 mg, 0.006 mmol, 0.02 equiv.), DABCO (33.6 mg, 0.3 mmol, 1.0 equiv.), in 1.0 mL MeCN for 4 h. Purification by silica gel chromatography afforded the desired **39** and as a white solid in 30% yield (29 mg). **^{1}H NMR** (400 MHz, $CDCl_3$, 300 K): δ (ppm) = δ 7.31 (d, J = 2.2 Hz, 1H), 7.24 – 7.20 (m, 2H), 7.19 – 7.15 (m, 1H), 7.12 – 7.09 (m, 2H), 6.85 (s, 2H), 6.73 (d, J = 2.2 Hz, 1H), 4.38 – 4.32 (m, 2H), 3.46 (s, 6H), 1.35 (t, J = 7.1 Hz, 3H). **^{13}C NMR** (100 MHz, $CDCl_3$, 300 K): δ (ppm) = δ 163.6, 144.5, 136.0, 133.8, 127.7, 125.8, 121.6, 107.8, 60.2, 36.2, 14.4.. **^{11}B NMR** (128.4 MHz, $CDCl_3$, 300 K): δ (ppm) = -10.63 (d, J = 78.8 Hz). **HRMS** (ESI) m/z: $[M+H]^+$ Calcd for $C_{17}H_{22}BN_4O_2^+$: 325.1830 ; Found: 325.1822.

7. Crystal data

Method for single crystals cultivation: The single crystal for compound **23** (CCDC- 2290496) and **35** (CCDC-2290510) were prepared from a mixture solvent of DCM and PE (v/v = 1:1). a pure solid sample (10–20 mg) was dissolved in DCM (2 mL) in a vial at room temperature, and PE (2-3 mL) was added into the above solution slowly while keeping the sample completely dissolved. The vial was properly sealed with parafilm and kept at room temperature to allow the slow evaporation of the solvents until a single crystal was obtained.

(1) Crystal data of 23 (Thermal ellipsoids are shown with 50% probability.)

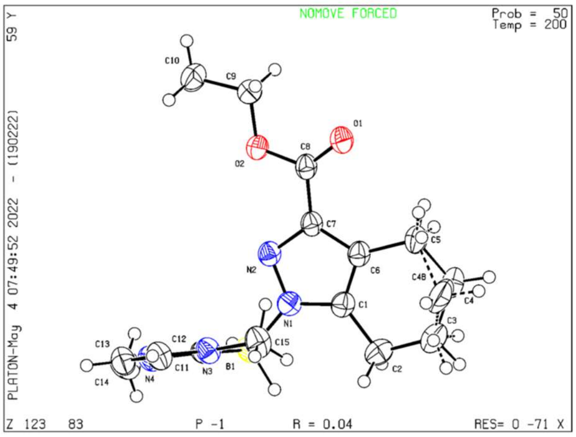

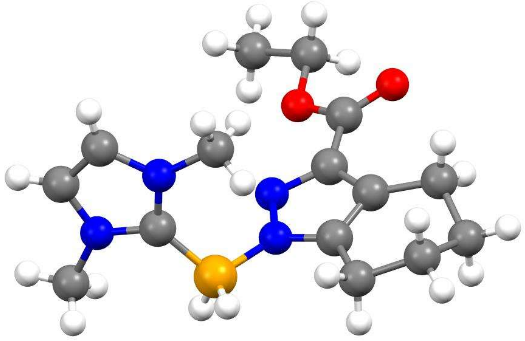

Identification code	23
Empirical formula	$C_{15}H_{23}BN_4O_2$
Formula weight	302.18
Temperature/K	199.99(10)
Crystal system	triclinic
Space group	P-1
a/Å	6.87670(10)

b/Å	11.0894(2)
c/Å	11.7324(2)
α/°	108.110(2)
β/°	93.384(2)
γ/°	100.099(2)
Volume/Å^3	831.01(3)
Z	2
ρ_{calc}g/cm^3	1.208
μ/mm^{-1}	0.651
F(000)	324.0
Crystal size/mm^3	0.14 × 0.12 × 0.1
Radiation	Cu Kα (λ = 1.54184)
2Θ range for data collection/°	7.988 to 143.256
Index ranges	-8 ≤ h ≤ 7, -13 ≤ k ≤ 13, -13 ≤ l ≤ 14
Reflections collected	7181
Independent reflections	3134 [R_{int} = 0.0176, R_{sigma} = 0.0197]
Data/restraints/parameters	3134/3/221
Goodness-of-fit on F^2	1.055
Final R indexes [I>=2σ (I)]	R_1 = 0.0422, wR_2 = 0.1132
Final R indexes [all data]	R_1 = 0.0442, wR_2 = 0.1147
Largest diff. peak/hole / e Å^{-3}	0.22/-0.17

(2) Crystal data of 35 (Thermal ellipsoids are shown with 50% probability).

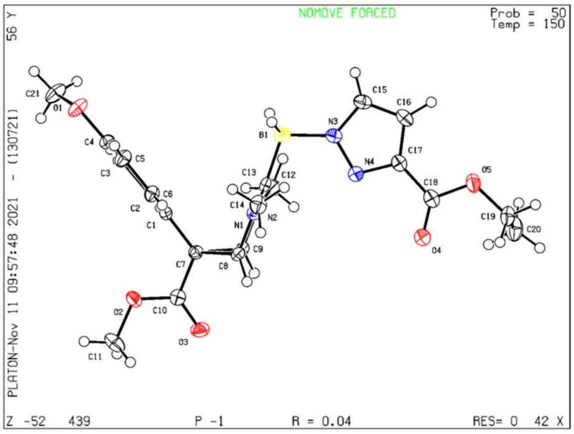

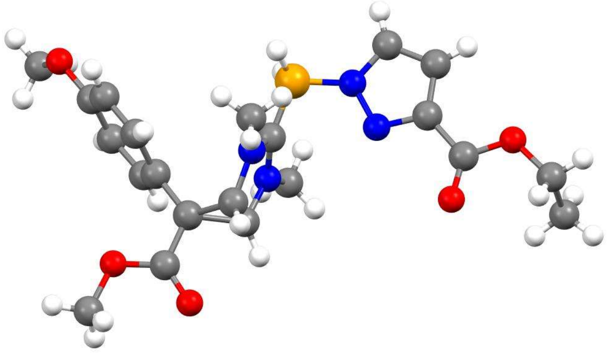

Identification code	35
Empirical formula	$C_{21}H_{27}BN_4O_5$
Formula weight	426.27
Temperature/K	150.00(10)
Crystal system	triclinic
Space group	P-1
a/Å	7.6901(6)
b/Å	11.3087(9)
c/Å	13.3739(11)
α/°	75.394(7)
β/°	79.905(7)
γ/°	72.486(7)
Volume/Å^3	1067.06(16)
Z	2
ρ_{calc}g/cm^3	1.327
μ/mm^{-1}	0.095
F(000)	452.0
Crystal size/mm^3	0.14 × 0.12 × 0.1
Radiation	Mo Kα (λ = 0.71073)
2Θ range for data collection/°	4.446 to 49.996
Index ranges	-9 ≤ h ≤ 7, -13 ≤ k ≤ 13, -15 ≤ l ≤ 14
Reflections collected	6448
Independent reflections	3741 [R_{int} = 0.0254, R_{sigma} = 0.0432]

Data/restraints/parameters	3741/0/293
Goodness-of-fit on F^2	1.071
Final R indexes [I>=2σ (I)]	R_1 = 0.0448, wR_2 = 0.1054
Final R indexes [all data]	R_1 = 0.0568, wR_2 = 0.1134
Largest diff. peak/hole / e $Å^{-3}$	0.20/-0.29

(3) Crystal data of 47 (Thermal ellipsoids are shown with 50% probability.) The single crystal for compound 47 (CCDC- 2296947) were prepared from a mixture solvent of MeOH : CH_2Cl_2 = (v/v = 1:1). a pure solid sample (10–20 mg) was dissolved in DCM (2 mL) in a vial at room temperature, and MeOH (2-3 mL) was added into the above solution slowly while keeping the sample completely dissolved. The vial was properly sealed with parafilm and kept at room temperature to allow the slow evaporation of the solvents until a single crystal was obtained.

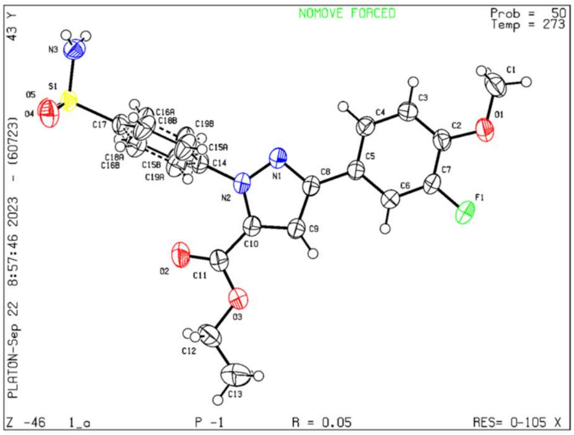

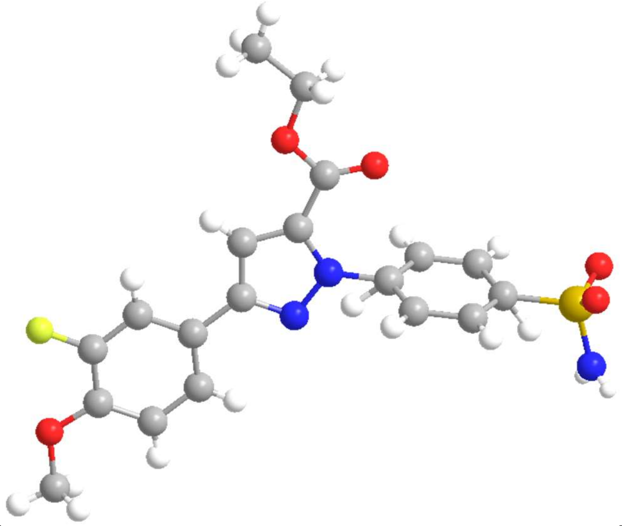

Identification code	47
Empirical formula	$C_{19}H_{18}FN_3O_5S$

Formula weight	419.42
Temperature	273(2) K
Wavelength	1.54178 Å
Crystal system	Triclinic
Space group	P-1
Unit cell dimensions	a = 5.09270(10) Å
	b = 10.7064(3) Å
	c = 18.6668(5) Å
Volume	973.76(4) $Å^3$
Z	2
Density (calculated)	1.430 Mg/m^3
Absorption coefficient	1.895 mm^{-1}
F(000)	436
Crystal size	0.220 x 0.200 x 0.180 mm^3
Theta range for data collection	2.459 to 72.181°.
Index ranges	-6<=h<=5, -13<=k<=13, -22<=l<=22
Reflections collected	11228
Independent reflections	3790 [R(int) = 0.0478]
Completeness to theta = 67.679°	99.1 %
Absorption correction	Semi-empirical from equivalents
Refinement method	Full-matrix least-squares on F^2
Data / restraints / parameters	3790 / 175 / 301
Goodness-of-fit on F^2	1.145
Final R indices [I>2sigma(I)]	R1 = 0.0483, wR2 = 0.1421
R indices (all data)	R1 = 0.0566, wR2 = 0.1522
Extinction coefficient	n/a

8. Schemes of computed reaction pathways

All calculations were performed using Gaussian 16, Revision A.03 package. [1] All of the reactants, intermediates, transition states, products were optimized by the DFT with the M06-2X functional. [2] For geometry optimizations and frequency calculations, BS-I basis set system was employed. In BS-I, we employed 6-311G(d) basis sets for C, H, O, N and B. All the stationary structures were characterized with no imaginary frequency and the transition state structures (TSs) were characterized with a single imaginary frequency. Intrinsic reaction coordinate (IRC) calculations were performed on the TSs. The solvent effect of Acetonitrile was evaluated through the SMD method, [3] in which a better basis system BS-II was used. In BSII, we employed 6-311++G(d, p) basis sets for all atoms. All reported energies are free energies at a concentration of 1 M and a temperature of 298.15 K.

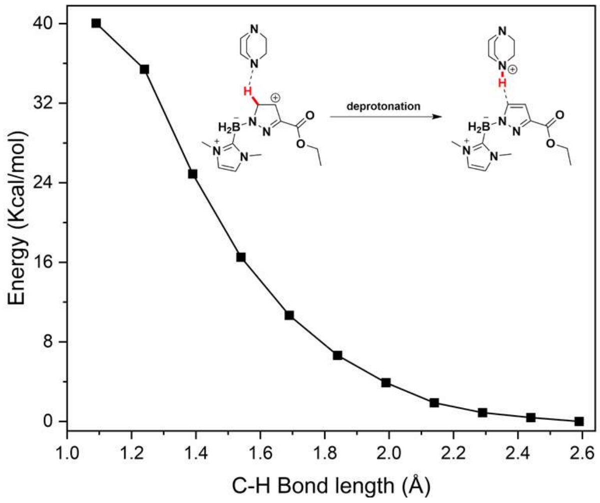

Figure S8. The scanned energy profiles for the deprotonation process

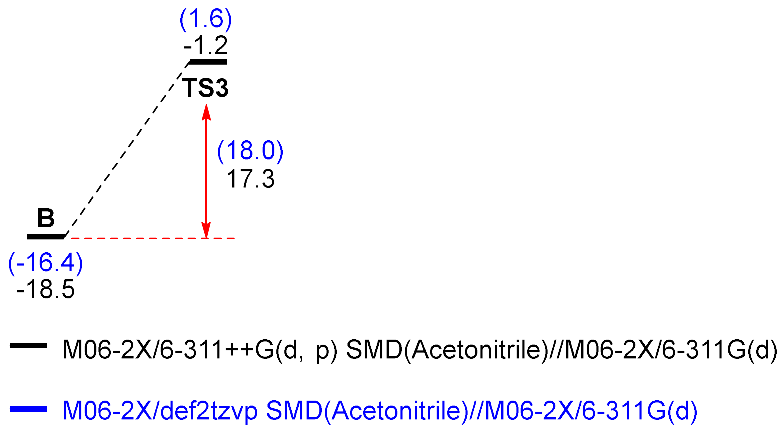

Figure S9. The reaction barriers for the key transition states with two basis sets.

Cartesian coordinates of the optimized structures:

TS1

E = -824.047477791 a.u.

0 2

C	-3.57508500	0.39163500	-0.55993300
C	-2.32079200	-0.04975600	-0.45078200
H	-3.81065000	1.41974800	-0.80982800
H	-4.40838900	-0.28078900	-0.39938300
H	-2.13207800	-1.08975400	-0.20766700
C	-1.10463800	0.73489100	-0.63343300
N	-1.29285500	3.15418400	-1.06108900
N	-1.15132900	2.02913300	-0.87018500
C	0.25256500	0.19186900	-0.58770400
O	1.26964200	0.82141200	-0.74802000
O	0.24471900	-1.13685800	-0.33442600
C	1.53472100	-1.75028200	-0.26770900
C	1.31524100	-3.21822500	0.02130700
H	2.12178800	-1.26157100	0.51297900
H	2.05391000	-1.59196800	-1.21547800
H	2.27236200	-3.73934800	0.08354000
H	0.78854300	-3.34814000	0.96791400
H	0.72071300	-3.67976100	-0.76851000
C	-1.51714600	7.23875000	-1.78232200
C	-1.73335400	7.39047700	-0.45416800
H	-0.99733700	7.86490100	-2.48708900
H	-1.43917100	8.17450400	0.22237900
N	-2.45656900	6.29453700	-0.03717000
C	-2.07222900	5.45207400	-3.47660300
H	-3.08219500	5.36536800	-3.87678100
H	-1.47210400	6.08439800	-4.12761200
H	-1.62536900	4.46110000	-3.40770000
C	-2.85098000	6.00510800	1.33098100
H	-2.58782100	6.85579900	1.95624600
H	-3.92542600	5.83377600	1.37794000
H	-2.33265100	5.11356400	1.68394600
B	-3.32230100	4.05447800	-1.04243600
H	-3.76237500	3.67695000	-0.00146800
H	-3.68058800	3.57536800	-2.07302700
N	-2.11024800	6.05265000	-2.15298400
C	-2.69923700	5.46186600	-1.08238100

TS2

E = -824.045928585 a.u.

0 2

C	-3.53367900	0.20744300	-1.20149900
C	-2.25781800	-0.24504600	-1.05370900
H	-3.73313000	1.20945000	-1.56744900
H	-4.34282600	-0.50123500	-1.32110600
H	-2.04716000	-1.28952000	-0.85610100
C	-1.09506800	0.59832400	-0.96724100
N	-1.40441000	3.02156100	-0.99188000
N	-1.23458800	1.90824600	-0.97031100
C	0.26184400	0.06927600	-0.81644900
O	0.52044400	-1.10425900	-0.77344200
O	1.18140600	1.04825100	-0.73273100
C	2.53725900	0.60661200	-0.57708500
C	3.40048200	1.84471300	-0.50006700
H	2.80014500	-0.02810000	-1.42575900
H	2.60715500	-0.00218100	0.32650800
H	4.44878700	1.56581100	-0.38096000
H	3.30162000	2.43881700	-1.40957000
H	3.10817600	2.46377400	0.34913300
C	-7.61668700	1.53195500	0.10996700
C	-7.07450200	2.73612100	-0.17863100
H	-8.62796700	1.17602900	0.00862800
H	-7.52303200	3.62895000	-0.58016700
N	-5.73346800	2.67018000	0.14291500
C	-6.73872100	-0.63152800	1.06007100
H	-6.48033700	-0.70394600	2.11768400
H	-7.76825000	-0.95290700	0.91212800
H	-6.06862900	-1.28283900	0.49665700
C	-4.77462600	3.74615200	0.01260900
H	-5.30083300	4.65209100	-0.28334100
H	-4.26793200	3.91105000	0.96418200
H	-4.02090700	3.50633600	-0.73972300
B	-4.04156600	0.89158600	1.00495300
H	-3.14232600	1.67374000	1.09845600
H	-3.97516700	-0.17267300	1.54078600
N	-6.60489600	0.73502800	0.60523800
C	-5.41786400	1.42741700	0.63962300

TS3

E = -824.07073431 a.u

0 2

H	2.24906200	8.67173200	-1.76973100

H	0.30867000	8.50958000	-2.18056200
B	1.24843500	8.00347800	-1.61067000
C	-0.11860100	7.98705700	1.95376500
C	1.11667300	7.47781900	2.16687200
H	-0.92853300	8.19028500	2.63310700
H	1.59695500	7.13748000	3.06830800
C	0.93490700	7.89916800	-0.02386300
N	-0.21174800	8.23810000	0.60084000
N	1.74785100	7.43862800	0.94422200
C	-1.38974900	8.78422800	-0.06254200
H	-1.15379800	9.74387100	-0.51733400
H	-1.73076800	8.09759000	-0.83395600
H	-2.17055500	8.91398500	0.68422000
C	3.09333600	6.90775600	0.73894500
H	3.72924400	7.25635700	1.55238000
H	3.05386500	5.82013500	0.69860000
H	3.46802800	7.28416400	-0.20973600
C	1.17191500	5.63105600	-4.01822400
C	1.79611200	4.38766300	-2.09926100
C	1.68458900	4.44674200	-3.49714300
H	0.18314900	5.98606000	-3.74775300
H	1.49224000	5.96242000	-5.00433100
H	2.22950200	3.73247200	-4.10445400
N	1.47919000	6.57718400	-2.16135500
N	1.71794200	5.57845100	-1.45035900
C	2.30719600	3.26773500	-1.30406100
O	2.61505900	3.30879000	-0.13654500
O	2.41816500	2.14629400	-2.04667500
C	2.94998900	1.00764200	-1.36480000
C	2.99542300	-0.12837800	-2.36162600
H	2.31222100	0.77676500	-0.50858600
H	3.94137700	1.25373800	-0.97731200
H	3.38992900	-1.03059600	-1.89064600
H	1.99578900	-0.34410300	-2.74139000
H	3.63523800	0.12825700	-3.20724000

A

E = -330.786752566 a.u

0 2

C	4.13794000	1.88357200	0.24478800
C	2.92209200	2.45730600	0.09260000
H	5.12457400	2.27910500	0.07186400
H	2.65162600	3.44606300	-0.23759000
N	1.97683700	1.52073900	0.45031600

C	4.95689700	-0.37759300	0.99247600
H	4.82932100	-1.26012500	0.36308300
H	5.93308000	0.06655600	0.80479100
H	4.88987800	-0.68570100	2.03739700
C	0.54402900	1.70507500	0.44029700
H	0.32019800	2.71606600	0.10380300
H	0.07695400	0.98371500	-0.23261500
H	0.13916000	1.55502800	1.44270000
B	1.92577600	-0.93008900	1.29551400
H	0.73468300	-0.98175400	1.35747400
H	2.60633900	-1.86499900	1.59210600
N	3.93321600	0.59745500	0.69509000
C	2.58363100	0.34129300	0.83349800

B

E = -824.098404476 a.u

0 2

H	2.57169200	9.04531500	-1.27809400
H	0.72191000	8.94338300	-2.00363600
B	1.59745600	8.34420300	-1.43147100
C	-0.27652600	7.21991600	1.69497400
C	0.98345300	6.84088100	2.01438600
H	-1.20419300	7.11943300	2.23180600
H	1.37336600	6.33379700	2.88031300
C	1.06544300	7.81780400	0.00417900
N	-0.20545100	7.81596200	0.45396900
N	1.79069900	7.22189000	0.96694200
C	-1.34623900	8.34044600	-0.28855300
H	-1.23242400	9.41103000	-0.44444400
H	-1.41620300	7.84245000	-1.25332200
H	-2.24703800	8.14694200	0.29020500
C	3.22238700	6.93588800	0.88635300
H	3.67393800	7.14939100	1.85465100
H	3.36730600	5.89416700	0.60037900
H	3.65633700	7.58372200	0.12885300
C	0.79969900	5.39510100	-4.36945500
C	2.04632200	4.76562600	-2.30735100
C	1.33851000	4.48815800	-3.54655000
H	0.85231000	6.45989900	-4.17897800
H	0.29966300	5.07440500	-5.27594300
H	1.25908300	3.43443400	-3.79201900
N	1.99729900	7.09610000	-2.28705600
N	2.19895600	5.98625300	-1.82136000
C	2.60335900	3.72029300	-1.44630000

O	3.15189900	3.89163400	-0.37920400
O	2.44009900	2.48919300	-1.97418500
C	2.96431500	1.40938500	-1.19700200
C	2.68885300	0.13478200	-1.96233200
H	2.48446000	1.41079000	-0.21570300
H	4.03294600	1.57067900	-1.03969000
H	3.07226300	-0.72693400	-1.41295100
H	1.61649600	0.00053600	-2.11178100
H	3.17085500	0.16365200	-2.94052600

F

E = -824.131278258 a.u

0 2

H	2.24775900	8.39080100	-2.11993100
H	0.29251400	8.32636100	-2.49329000
B	1.20420700	7.80264700	-1.89101700
C	-0.12927600	8.12210500	1.66720100
C	1.14618500	7.74839700	1.92192800
H	-0.94884400	8.34371900	2.32899300
H	1.65872600	7.56643800	2.85103800
C	0.91451700	7.86847400	-0.30019300
N	-0.25175400	8.18834400	0.29548400
N	1.76978400	7.60496700	0.70336900
C	-1.47733000	8.53037000	-0.41568100
H	-1.36225500	9.48192600	-0.93094800
H	-1.70371400	7.75451900	-1.14311500
H	-2.28474100	8.59939400	0.31071600
C	3.13583500	7.11981200	0.53727500
H	3.76345200	7.58084700	1.29952100
H	3.14315700	6.03400000	0.61189700
H	3.48112700	7.40419100	-0.45314300
C	1.29345400	5.80593000	-3.63381800
C	1.68882800	4.37916900	-3.42473100
H	0.30221600	5.94548800	-4.09196300
H	2.00338800	6.37550300	-4.25867500
H	1.79069400	3.61875400	-4.18171500
N	1.30289400	6.31149100	-2.26414700
C	2.40209200	3.06980800	-1.33791800
O	2.72029100	3.05681400	-0.17833700
O	2.45930100	1.98285900	-2.12942700
C	2.94066400	0.79028900	-1.50160800
C	2.93761000	-0.29860700	-2.55034600
H	2.29268300	0.54859500	-0.65610500
H	3.94129200	0.97687400	-1.10510500

H	3.29556500	-1.23699100	-2.12301800
H	1.92936100	-0.45605700	-2.93594800
H	3.58619500	-0.02874300	-3.38498000
N	1.71000800	5.39849600	-1.38755100
C	1.92341700	4.25407500	-2.08191300

G

E = -823.943394615 a.u

1 1

H	2.07662900	8.36410800	-2.19160500
H	0.12671200	8.10671200	-2.48155200
B	1.08867200	7.75974400	-1.85379900
C	-0.11035300	8.25693900	1.70510900
C	1.16022900	7.85082200	1.94082900
H	-0.89460300	8.55062200	2.38180300
H	1.69925700	7.71162900	2.86259000
C	0.84964100	7.85355600	-0.27017000
N	-0.28360600	8.24929300	0.34002700
N	1.73505600	7.61476100	0.71410800
C	-1.51859800	8.64561500	-0.33721000
H	-1.34516400	9.53322200	-0.94167300
H	-1.87257700	7.83373800	-0.96816500
H	-2.26557300	8.86358600	0.42181500
C	3.10261200	7.13492900	0.53293900
H	3.74259400	7.61906700	1.26766300
H	3.13267000	6.05300500	0.65435000
H	3.43927300	7.40677700	-0.46522800
C	1.39842100	5.78618700	-3.63600000
C	1.79604300	4.36952900	-3.47302100
H	0.40604400	5.92953000	-4.07628300
H	2.10446100	6.39774900	-4.20776200
H	1.93835900	3.64936300	-4.26494600
N	1.37202900	6.25541100	-2.24945600
C	2.38558200	2.97379500	-1.37659700
O	2.61861500	3.02133800	-0.20373600
O	2.48925600	1.91820200	-2.16513500
C	2.94019100	0.69342200	-1.52793800
C	2.99836400	-0.37056500	-2.59553100
H	2.23559000	0.45159400	-0.73112800
H	3.91316700	0.88914800	-1.07515400
H	3.33224500	-1.31171400	-2.15718200
H	2.01527000	-0.53004700	-3.03935200
H	3.69940000	-0.09328800	-3.38335800
N	1.66843900	5.35489600	-1.44132900

C	1.94495400	4.16838500	-2.15512300

3

E = -823.59644796 a.u

0 1

H	2.37470200	8.38082000	-2.06521300
H	0.43097600	8.34686500	-2.52189800
B	1.31501900	7.82498700	-1.88704300
C	-0.19195800	8.02421500	1.60970400
C	1.09093300	7.72489400	1.92025200
H	-1.05358400	8.18588500	2.23443800
H	1.56954500	7.56128300	2.87063200
C	0.95728100	7.86511200	-0.30776500
N	-0.25354100	8.10399900	0.23515600
N	1.77955100	7.63729200	0.73171900
C	-1.46275500	8.37866700	-0.53172100
H	-1.38236600	9.34232000	-1.03092800
H	-1.60594700	7.59929000	-1.27677600
H	-2.30700800	8.38845200	0.15472000
C	3.18119900	7.23725400	0.63279200
H	3.73296700	7.71214300	1.44343300
H	3.25050500	6.15231800	0.67748600
H	3.56615400	7.56746800	-0.32767500
C	1.02939100	5.70262800	-3.41272400
C	1.37931800	4.37162000	-3.31566700
H	0.55044600	6.25998400	-4.20261900
H	1.23443700	3.58263600	-4.03456500
N	1.40468200	6.31705400	-2.26639500
C	2.55423100	3.11376400	-1.35682500
O	3.06694100	3.12174400	-0.26854500
O	2.44649300	2.00205900	-2.11101300
C	2.99408100	0.81296000	-1.53429200
C	2.78141400	-0.30478800	-2.52995800
H	2.49420400	0.61658400	-0.58303100
H	4.05265800	0.97688000	-1.32040100
H	3.18233300	-1.24185200	-2.13937500
H	1.71797300	-0.44074800	-2.73194500
H	3.28357900	-0.07921700	-3.47178800
N	1.97872900	5.46111900	-1.42773400
C	1.97333000	4.27986600	-2.04295900

9. References:

1. Gaussian 16, Revision A.03, M. J. Frisch, G. W. Trucks, H. B. Schlegel, G. E. Scuseria, M. A. Robb, J. R. Cheeseman, G. Scalmani, V. Barone, G. A. Petersson, H. Nakatsuji, X. Li, M. Caricato, A. V. Marenich, J. Bloino, B. G. Janesko, R. Gomperts, B. Mennucci, H. P. Hratchian, J. V. Ortiz, A. F. Izmaylov, J. L. Sonnenberg, D. Williams-Young, F. Ding, F. Lipparini, F. Egidi, J. Goings, B. Peng, A. Petrone, T. Henderson, D. Ranasinghe, V. G. Zakrzewski, J. Gao, N. Rega, G. Zheng, W. Liang, M. Hada, M. Ehara, K. Toyota, R. Fukuda, J. Hasegawa, M. Ishida, T. Nakajima, Y. Honda, O. Kitao, H. Nakai, T. Vreven, K. Throssell, J. A. Montgomery, Jr., J. E. Peralta, F. Ogliaro, M. J. Bearpark, J. J. Heyd, E. N. Brothers, K. N. Kudin, V. N. Staroverov, T. A. Keith, R. Kobayashi, J. Normand, K. Raghavachari, A. P. Rendell, J. C. Burant, S. S. Iyengar, J. Tomasi, M. Cossi, J. M. Millam, M. Klene, C. Adamo, R. Cammi, J. W. Ochterski, R. L. Martin, K. Morokuma, O. Farkas, J. B. Foresman, and D. J. Fox, Gaussian, Inc., Wallingford CT, **2016**.

2. Y. Zhao, D. G. Truhlar, Theor Chem Account., **2008**, *120*, 215.

3. A. V. Marenich, C. J. Cramer, D. G. Truhlar, J. Phys. Chem. B., **2009**, *113*, 6378.

4. N. -P. Khot, P. -J. Nagtilak, N. -K. Deo, M. Kapur, *Chem Commun*. **2023**, *59*, 6076.

5. W. -Y. Li; X. -Y. Z; T. –B. Xiao; Z. -F. Ke; L. Zhou, *CCS. Chem.* **2022**, *4*, 638.

6. F. -J. Sarabia, Q. -K. Li, E.-M. Ferreira, *Angew. Chem. Int. Ed*. **2018**, *57*, 11015.

7. Z. -Y. Yu, M. -S. Eno, A. -H. Annis, J.-P. Morken, *Org. Lett*. **2015**, *17*, 3264.

8. A. S. K. -Raj , R. -S. Liu, *Angew. Chem. Int. Ed*. **2019**, *58*, 10980.

9. B. -K. Gall, A. -K. Smith, E. -M. Ferreira, *Angew. Chem. Int. Ed*. **2022**, *61*, e202212187.

10. A. S. K. -Raj, R. -S. Liu, *Adv. Synth. Catal.* **2020**, *362*, 2517.

11. S. T. R. -Müller, T. -Hokamp, S. -Ehrmann, P. -Hellier, T. -Wirth, *Chem. Eur. J.* **2016**, *22*, 11940.

12. B. -D. Schwartz, J. -R. Denton, Y. -J. Lian, H. M. -L. Davies, C. -M. Williams, *J. Am. Chem. Soc.* **2009**, *131*, 8329.

13. V. -V. Pagar, A. -M. Jadhav, R. -S. Liu, *J. Am. Chem. Soc.* **2011**, *133*, 20728.

14. H. -F. Zheng, K. -Wang, I. Faghihi, W. -P. Griffith, H. Arman, M. -P. Doyle, *ACS Catal.* **2021**, *11*, 9869.

15. A. Solovyev, S.-H. Ueng, J. Monot, L. Fensterbank, M Malacria, E. Lacôte, D. P. Curran, *Org. Lett.* **2010**, *12*, 2998.

16 M. M. Brahmi, J. Monot, M. D.-E. Murr, D. P. Curran, L. Fensterbank, E. Lacôte, M. Malacria, *J. Org. Chem.* **2010**, *75*, 6983.

17. S. Gardner, T. Kawamoto, D. P. Curran, *J. Org. Chem.* **2015**, *80*, 9794.

18 M. Toure, O. Chuzel, J.-L. Parrain, *J. Am. Chem. Soc.* **2012**, *134*, 17892.

19. D. A. Bolt, D. P. Curran, *J. Org. Chem.* **2017**, *82*, 13746.

20. Z.-L. Chen, C. Empel, K. Wang, P.-P. Wu, B.-G. Cai, L. Li, R. M. Koenigs, J. Xuan, *Org. Lett.* **2022**, *24*, 2232.

21. J. Britton, T. -F. Jamison,. *Eur. J. Org. Chem.* **2017**, *44*, 6566.

22. a) Z Hu, S.-J. Liu, H. -Y. Qin, J. -H. Zhou, X. -G. Peng, *J. Am. Chem. Soc.* **2020**, *142*, 4254. b) C.-H. Rao, H.-R. Wei, X.-L. Miao, M.-Z. Jia, X.-R. Yao, X.-Y. Zheng, J. Zhang, *Green Chem*., **2023**, *25*, 3974.

9. Copies of ^{1}H NMR, ^{13}C NMR and ^{11}B NMR Spectra

^{1}H NMR (400 MHz) Spectrum of 2b in $CDCl_3$

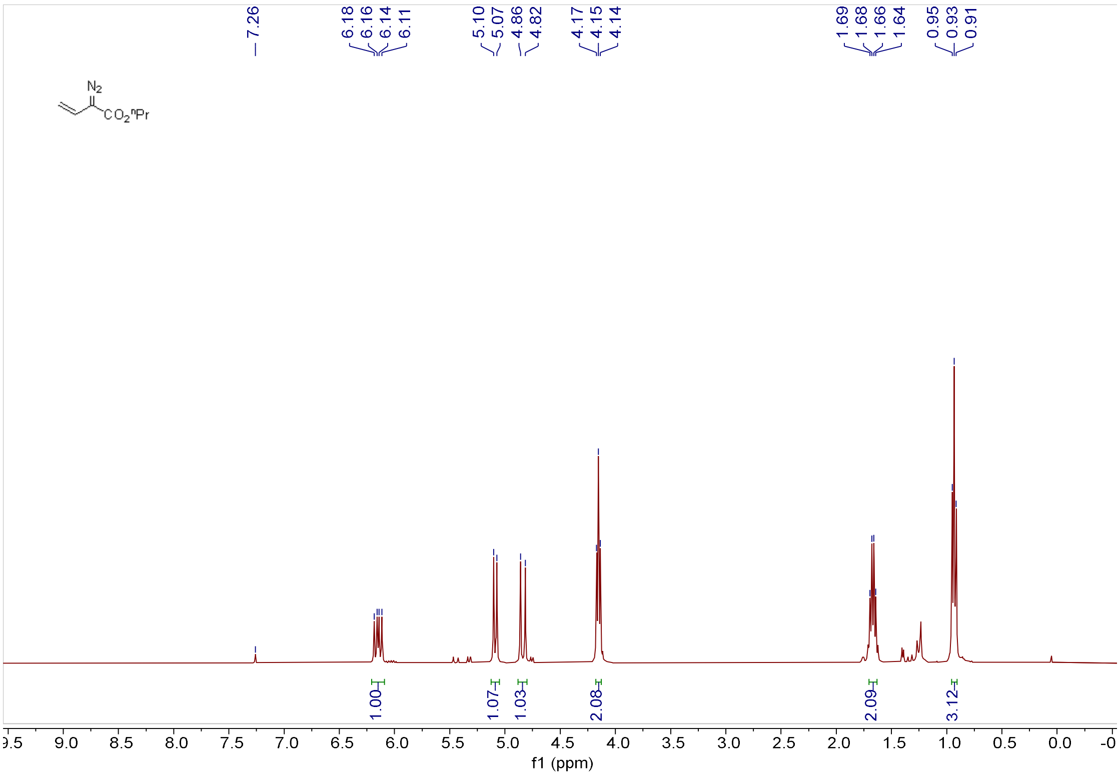

^{13}C NMR (100 MHz) Spectrum of 2b in $CDCl_3$、

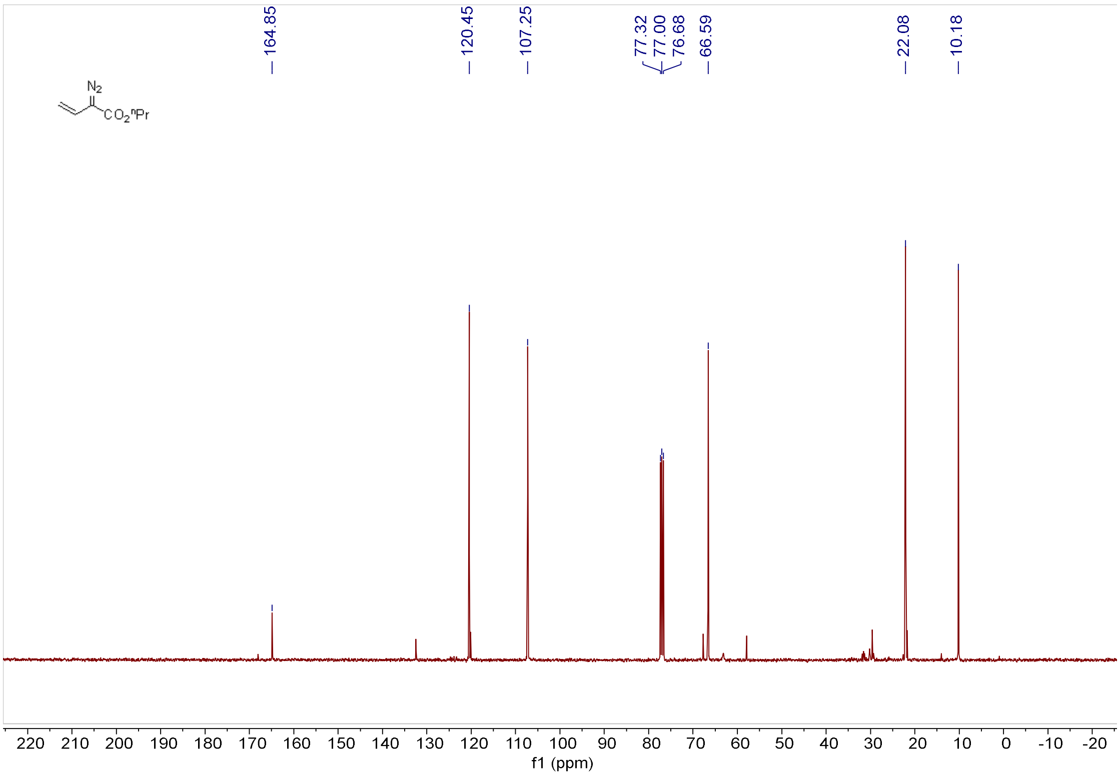

^{1}H NMR (400 MHz) Spectrum of 2d in $CDCl_3$

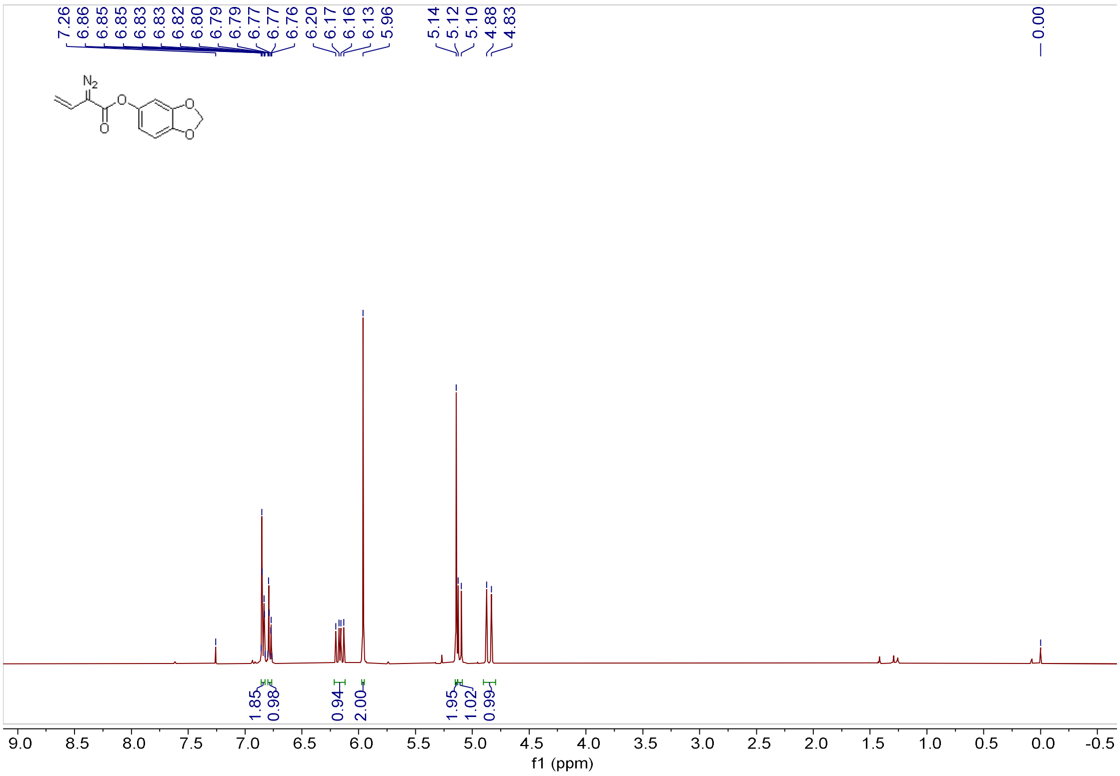

^{13}C NMR (100 MHz) Spectrum of 2d in $CDCl_3$

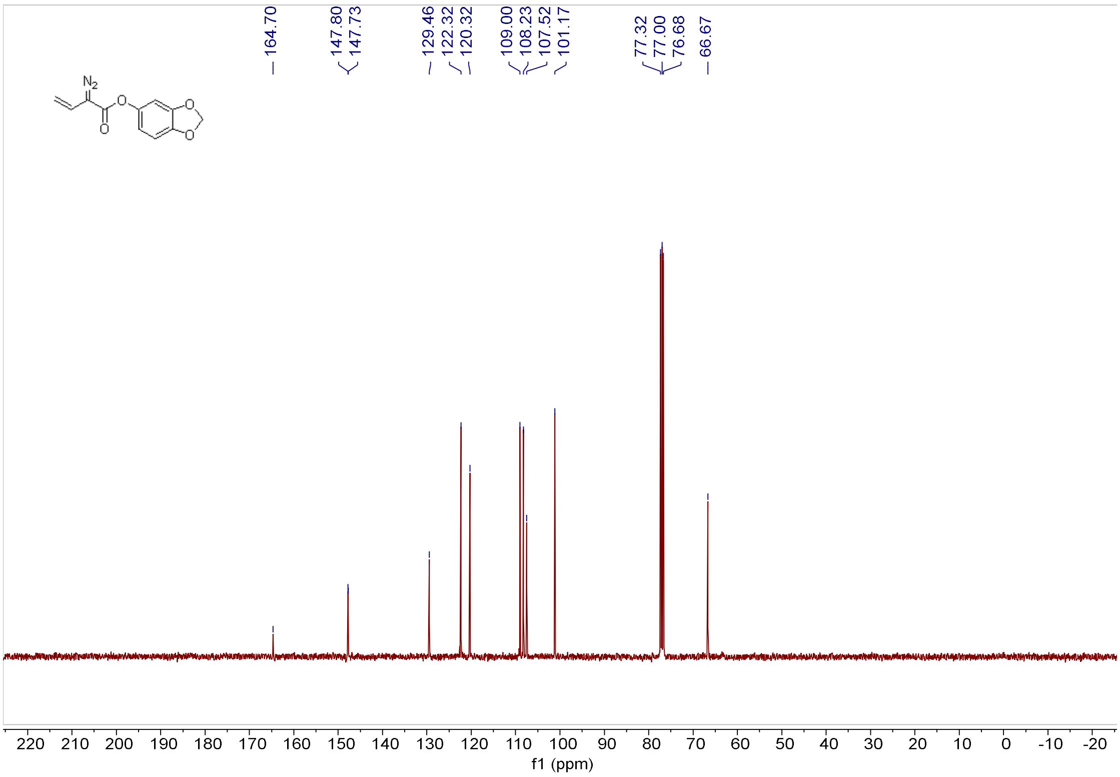

^{1}H NMR (400 MHz) Spectrum of 2h in $CDCl_3$

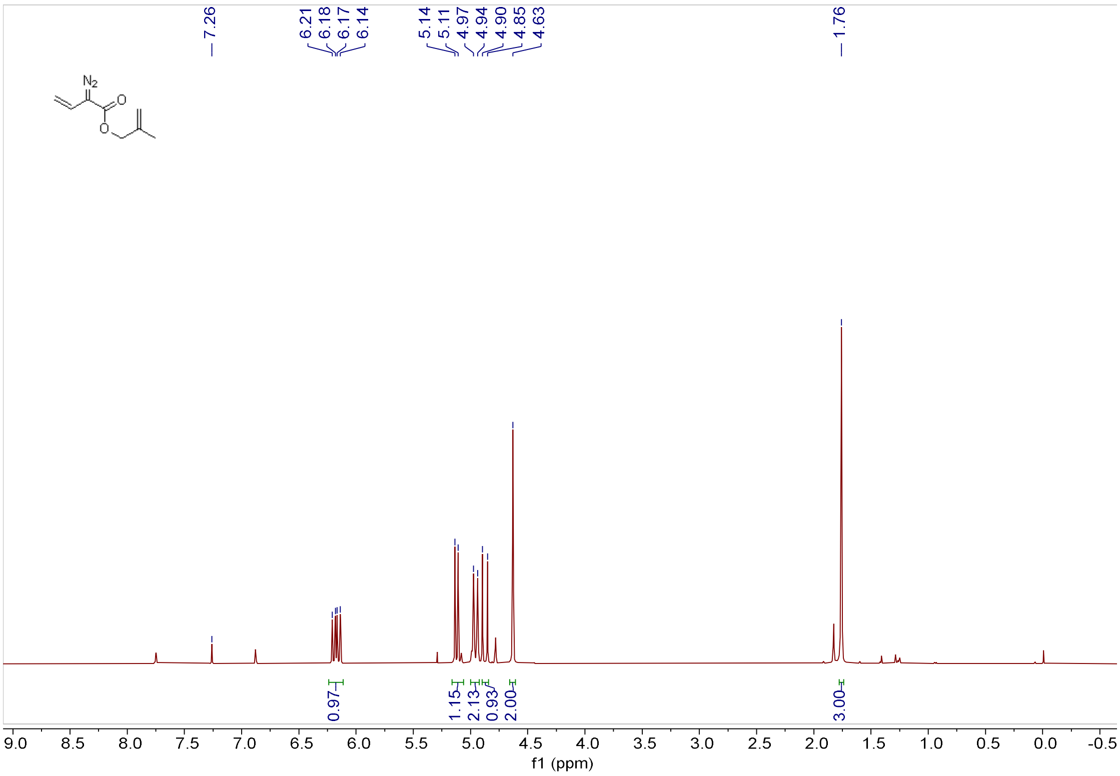

^{13}C NMR (100 MHz) Spectrum of 2h in $CDCl_3$

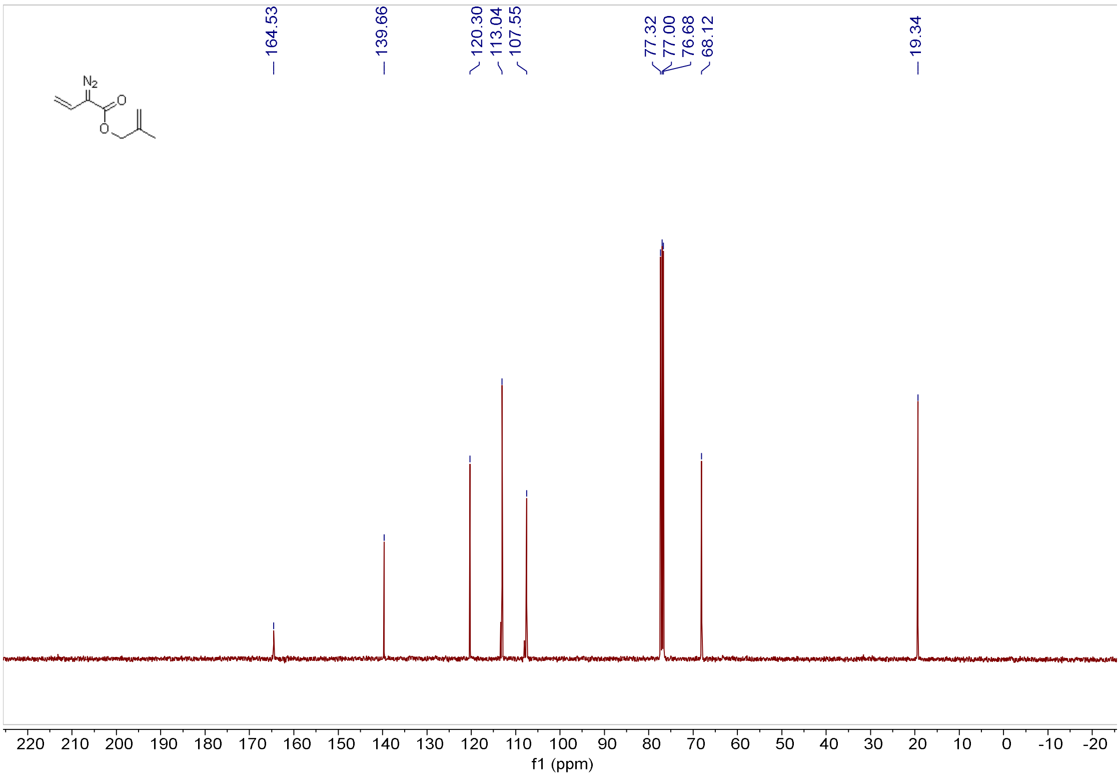

^{1}H NMR (400 MHz) Spectrum of 2i in $CDCl_3$

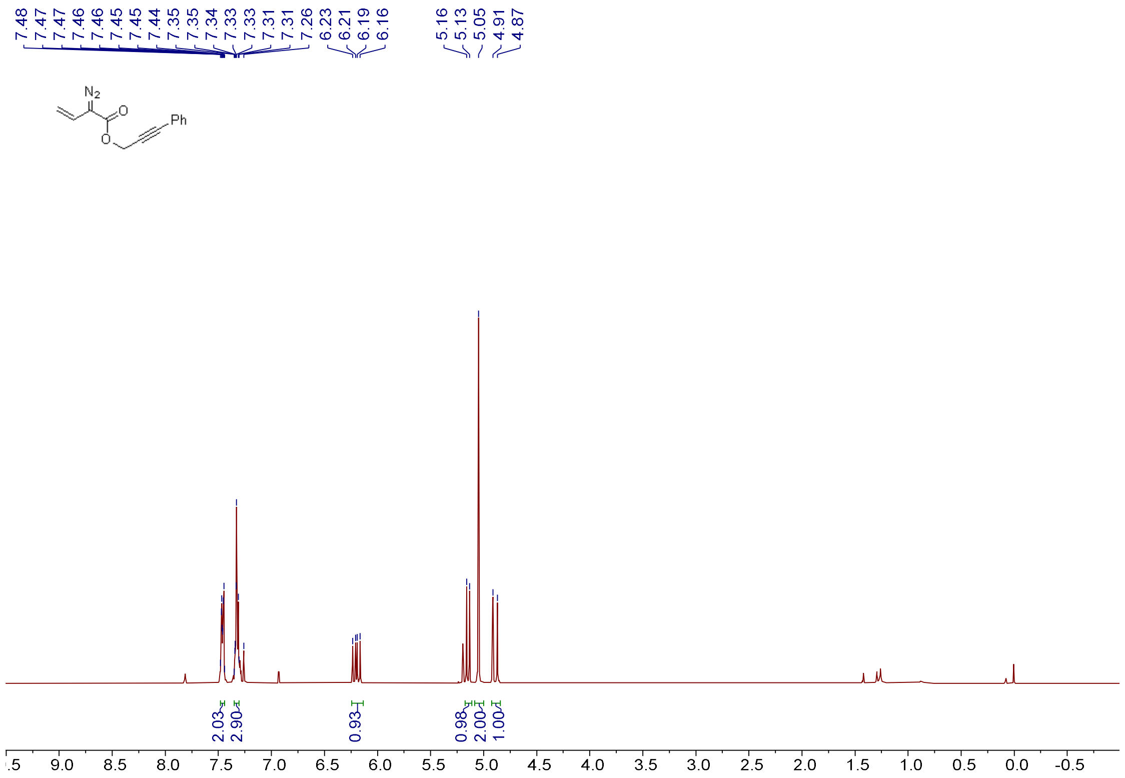

^{13}C NMR (100 MHz) Spectrum of 2i in $CDCl_3$

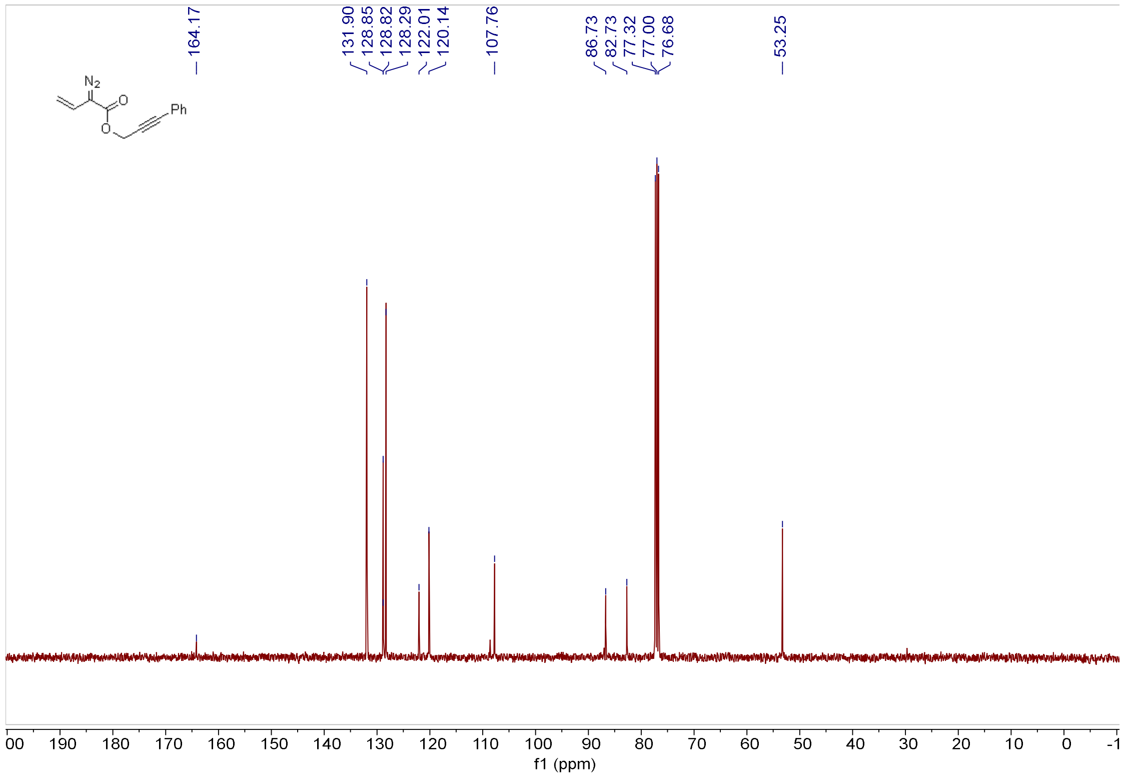

^{1}H NMR (400 MHz) Spectrum of 2k in $CDCl_3$

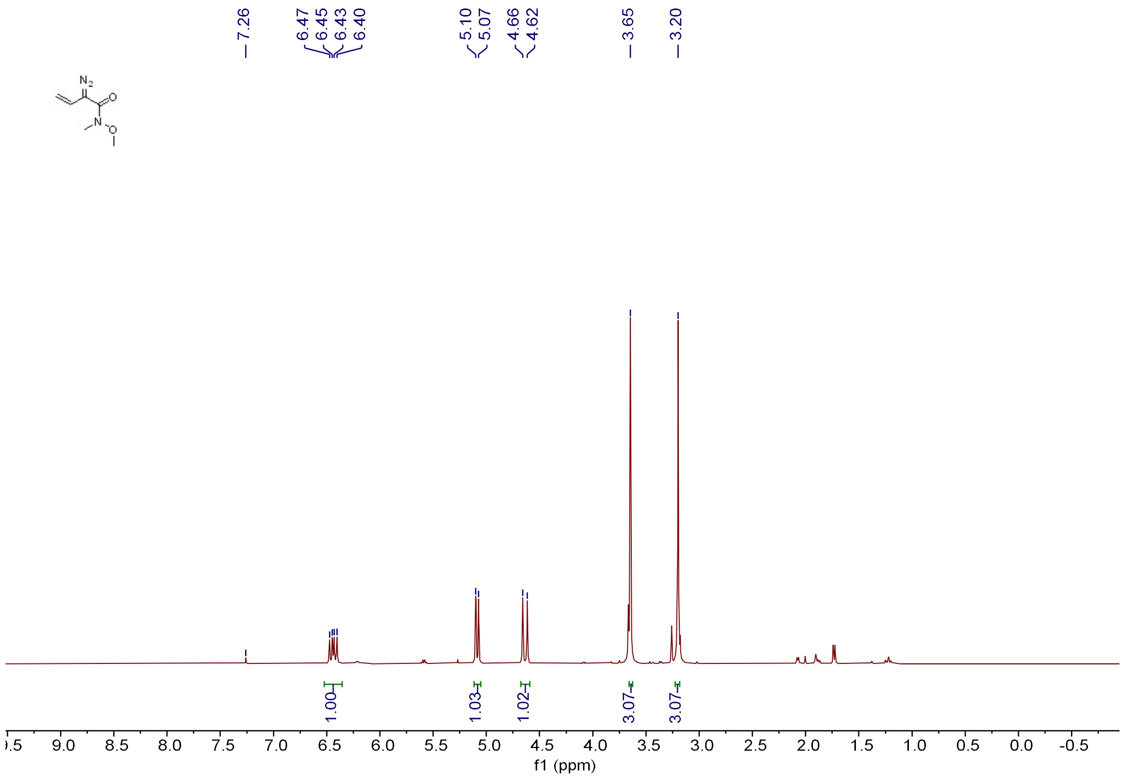

^{13}C NMR (100 MHz) Spectrum of 2k in $CDCl_3$

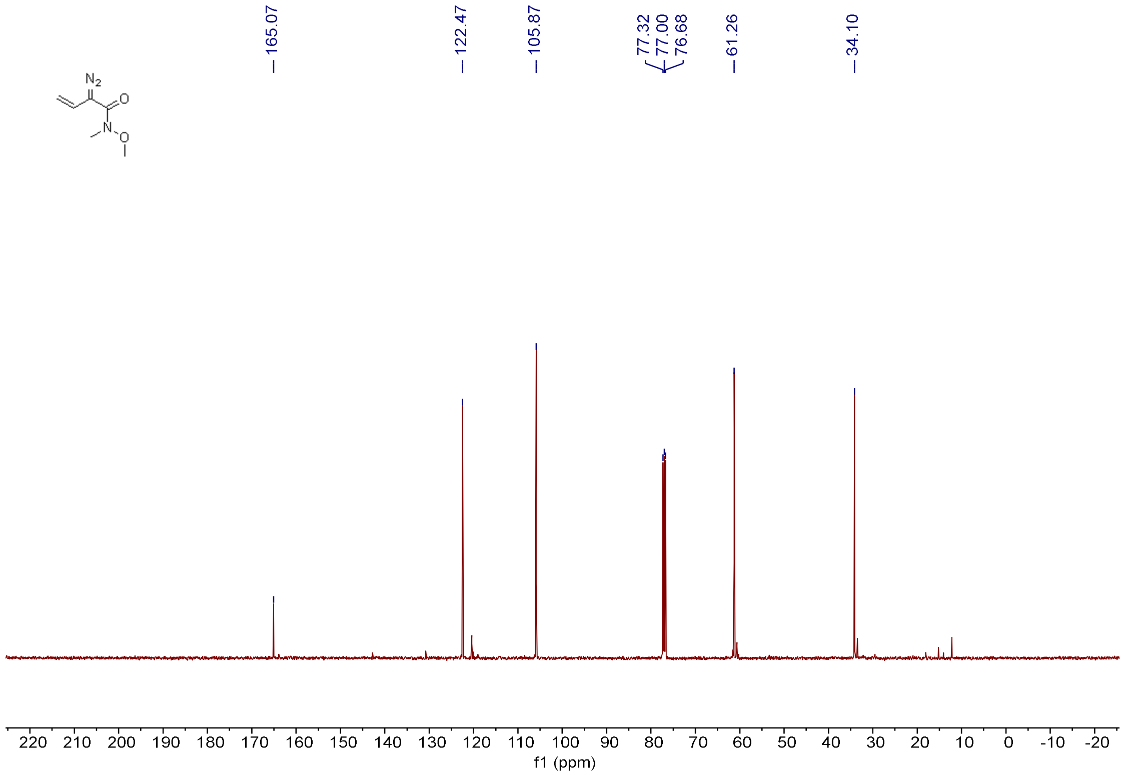

^{1}H NMR (400 MHz) Spectrum of 2q in CDCl$_3$

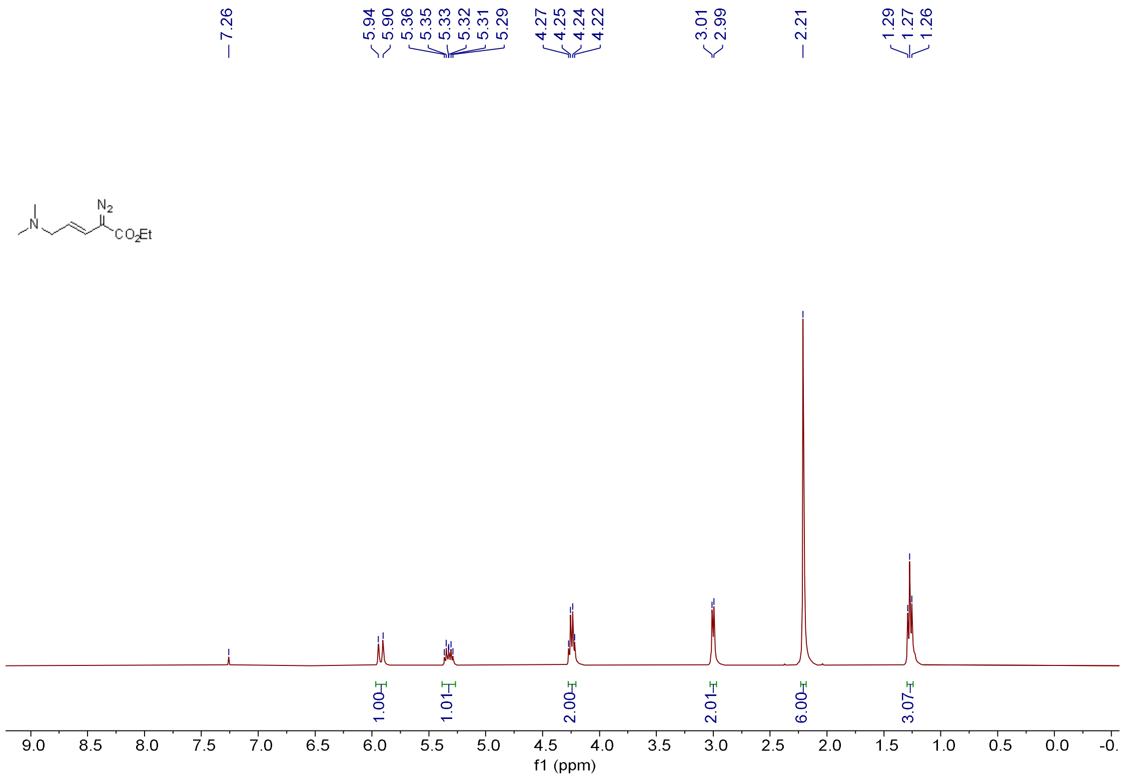

^{13}C NMR (100 MHz) Spectrum of 2q in CDCl$_3$

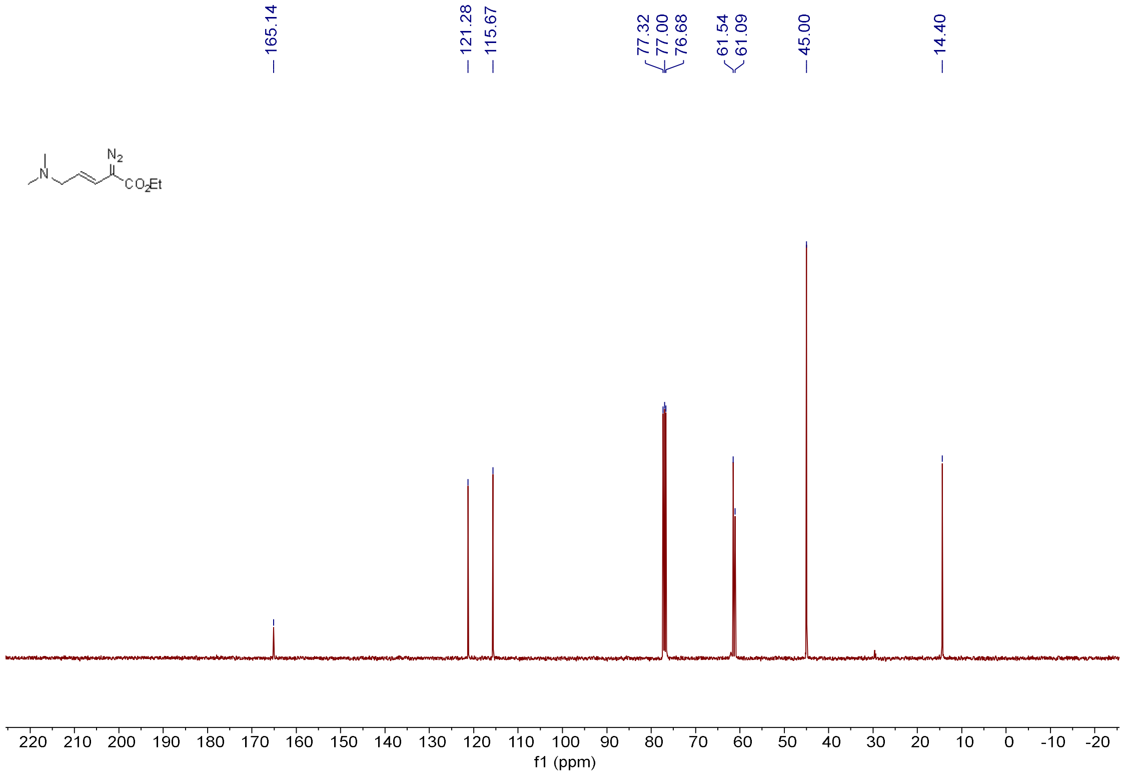

^{1}H NMR (400 MHz) Spectrum of 2t in $CDCl_3$

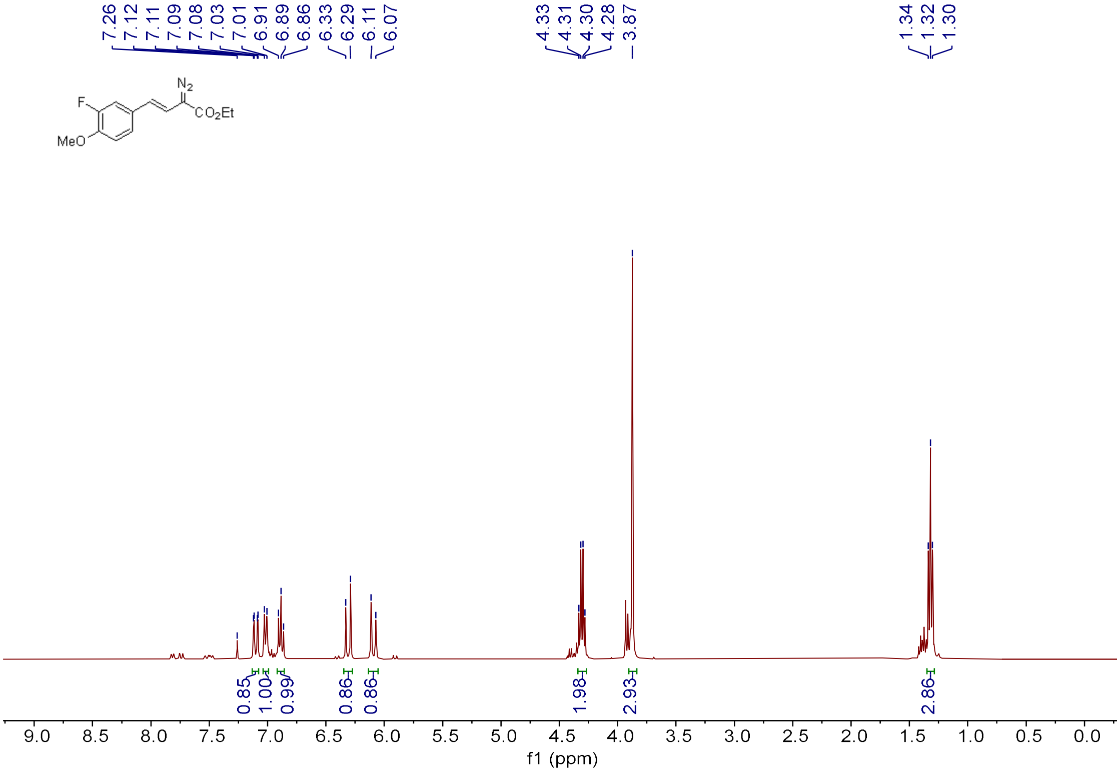

^{13}C NMR (100 MHz) Spectrum of 2t in $CDCl_3$

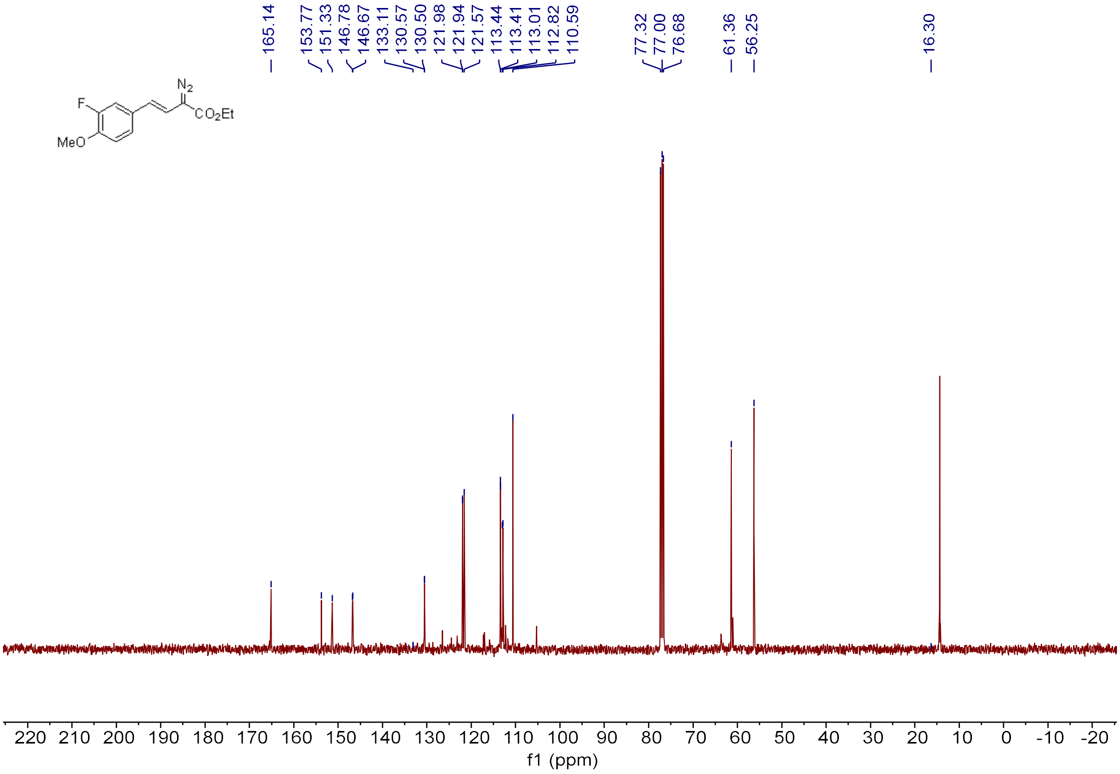

^{1}H NMR (400 MHz) Spectrum of 2w in $CDCl_3$

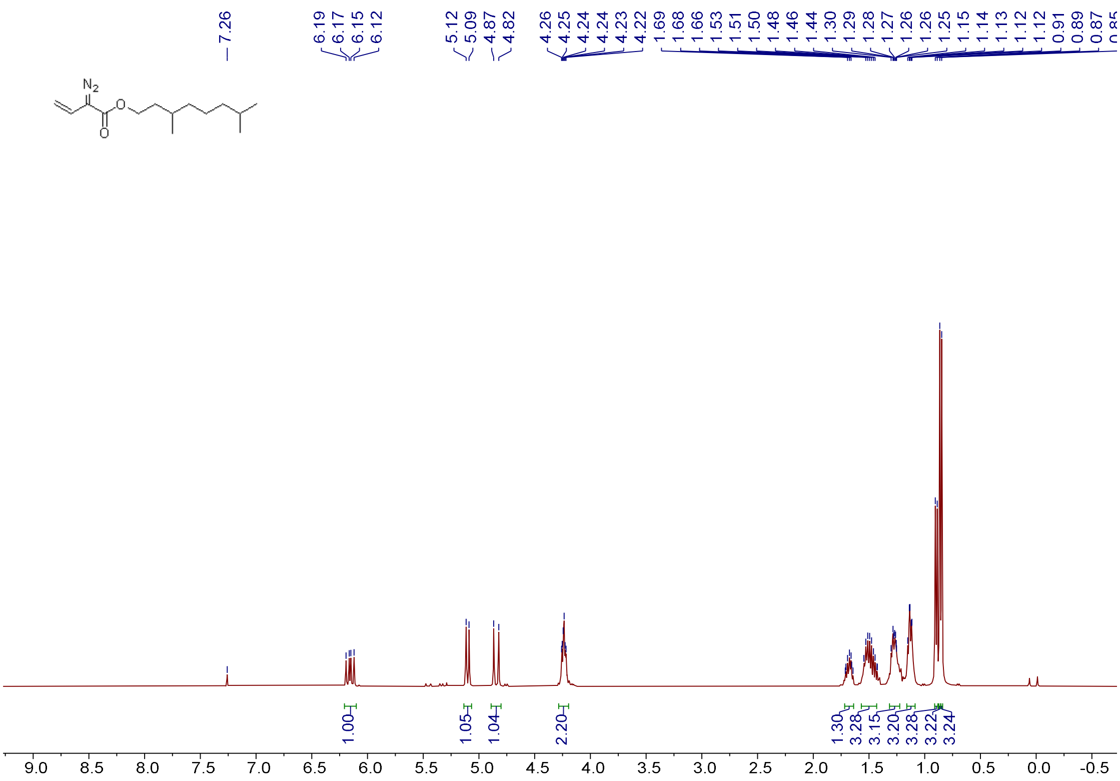

^{13}C NMR (100 MHz) Spectrum of 2w in $CDCl_3$

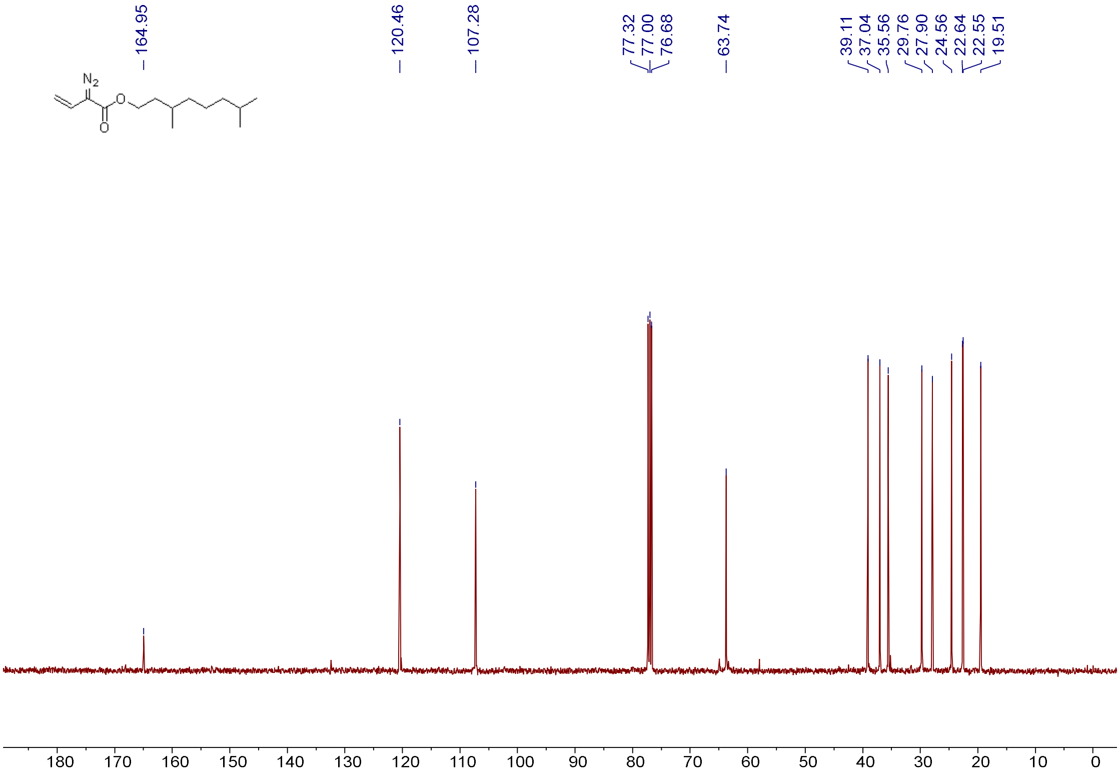

^{1}H NMR (400 MHz) Spectrum of 2x in $CDCl_3$

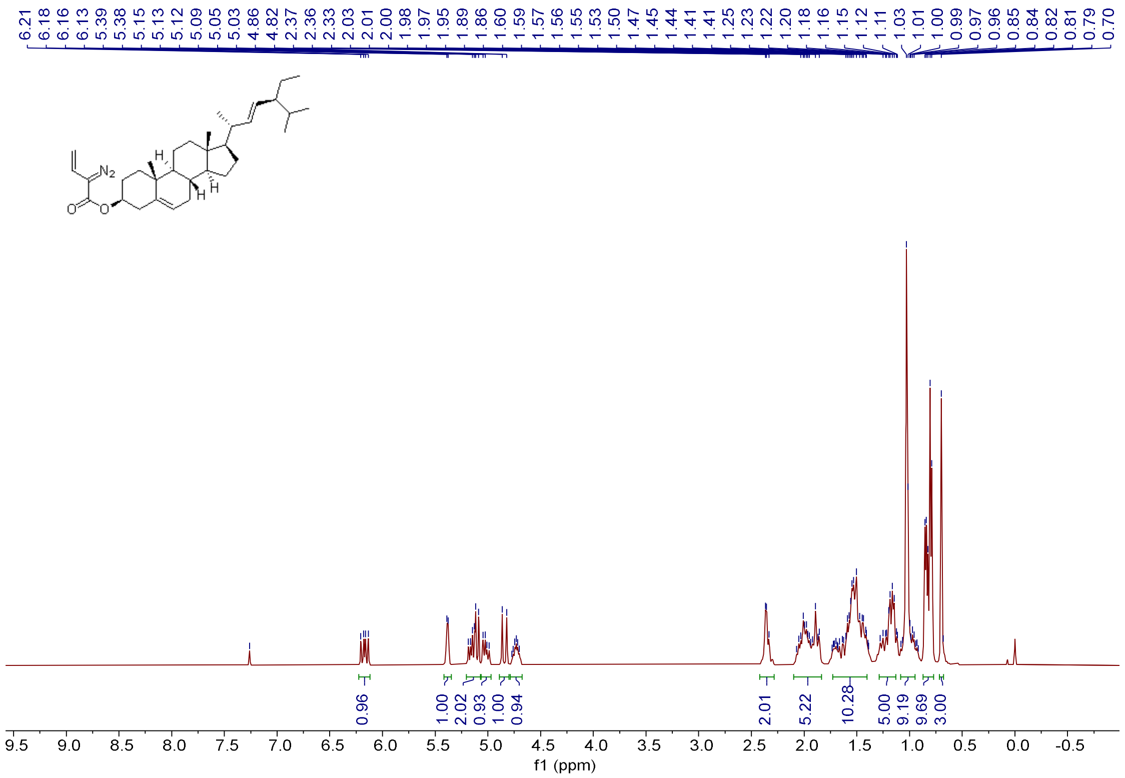

^{13}C NMR (100 MHz) Spectrum of 2x in $CDCl_3$

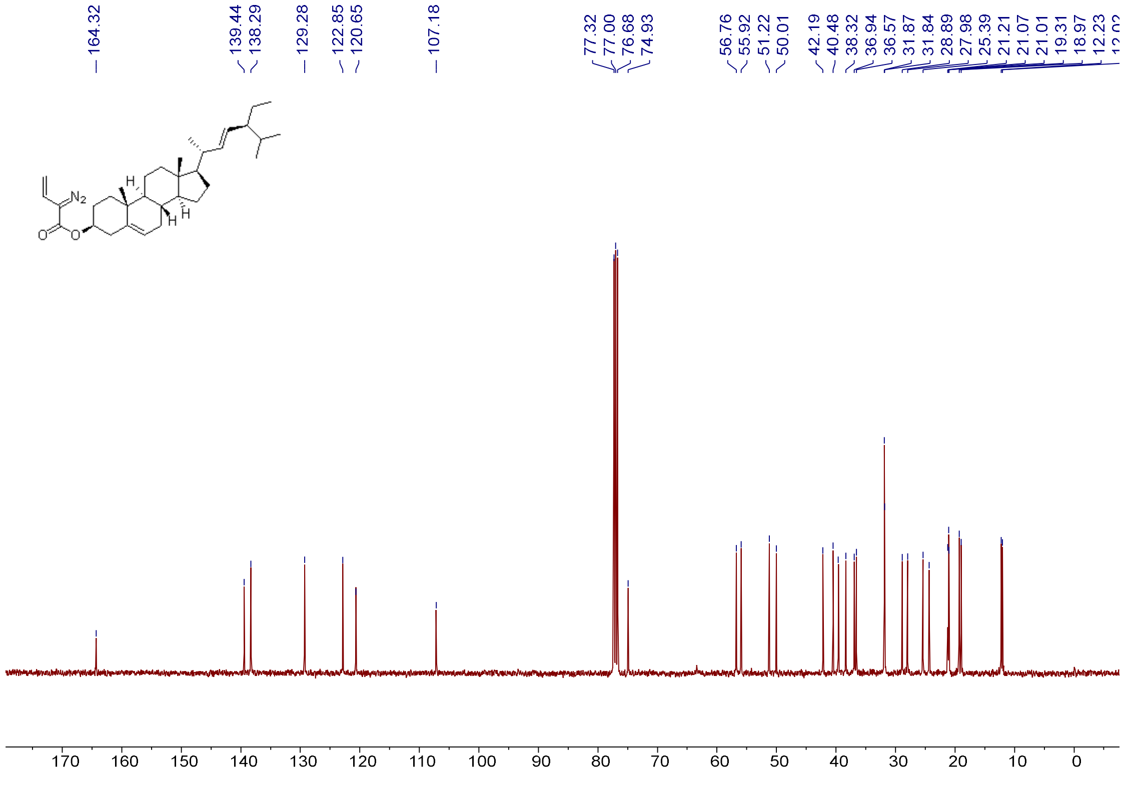

^{1}H NMR (400 MHz) Spectrum of 2aa in $CDCl_3$

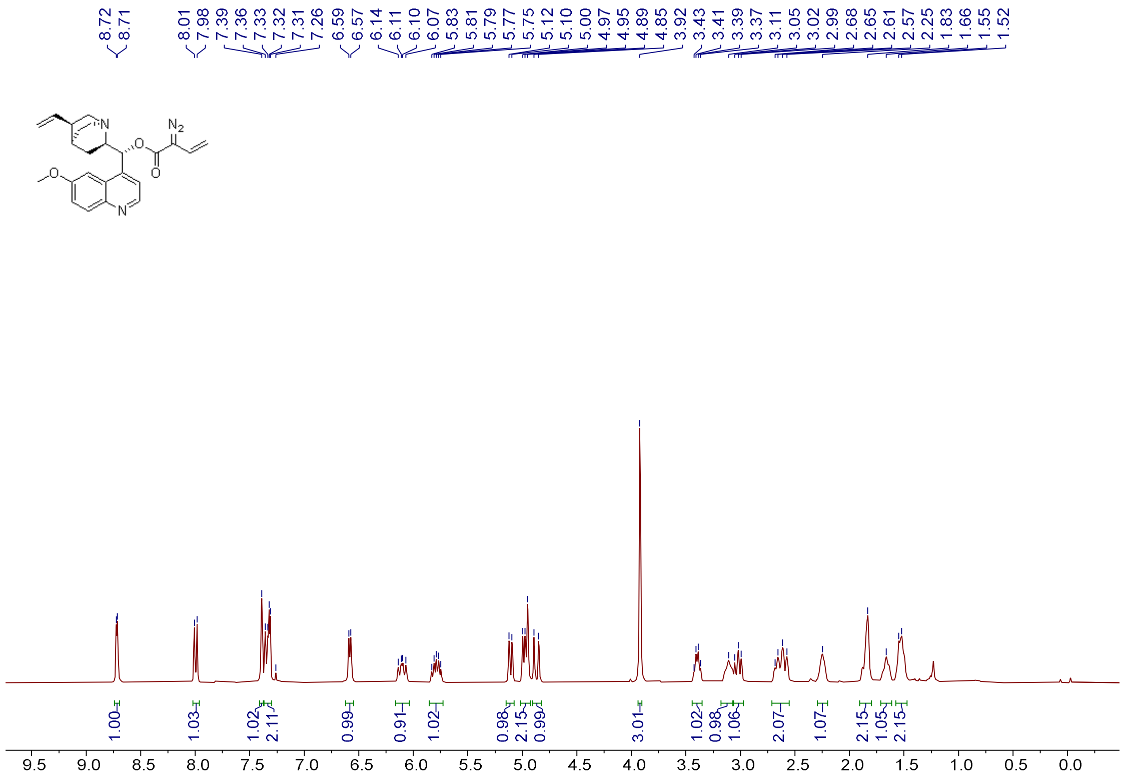

^{13}C NMR (100 MHz) Spectrum of 2aa in $CDCl_3$

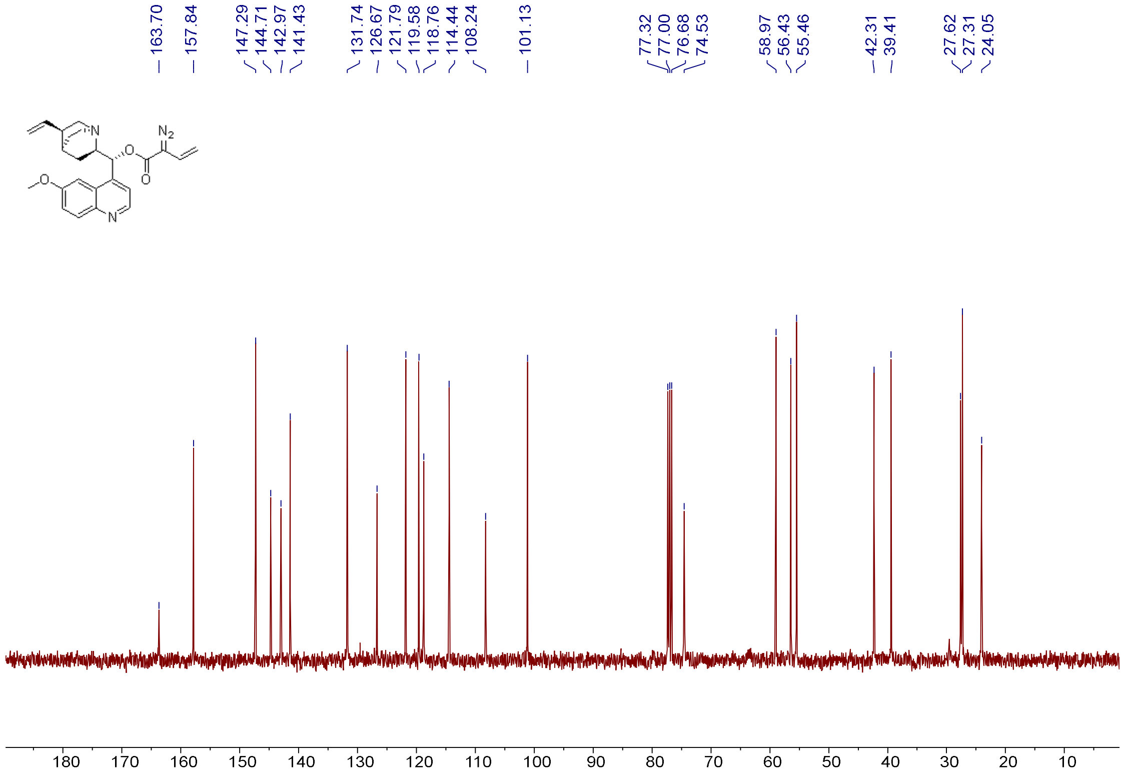

^{1}H NMR (400 MHz) Spectrum of 3 in $CDCl_3$

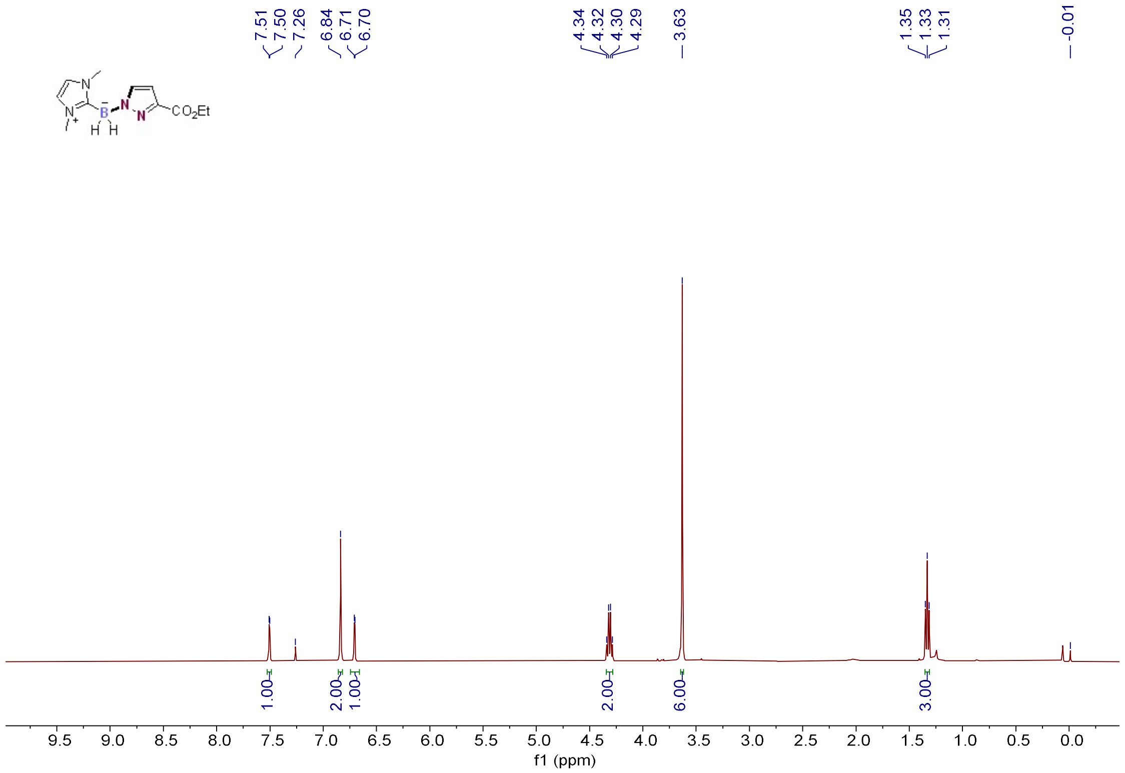

^{13}C NMR (100 MHz) Spectrum of 3 in $CDCl_3$

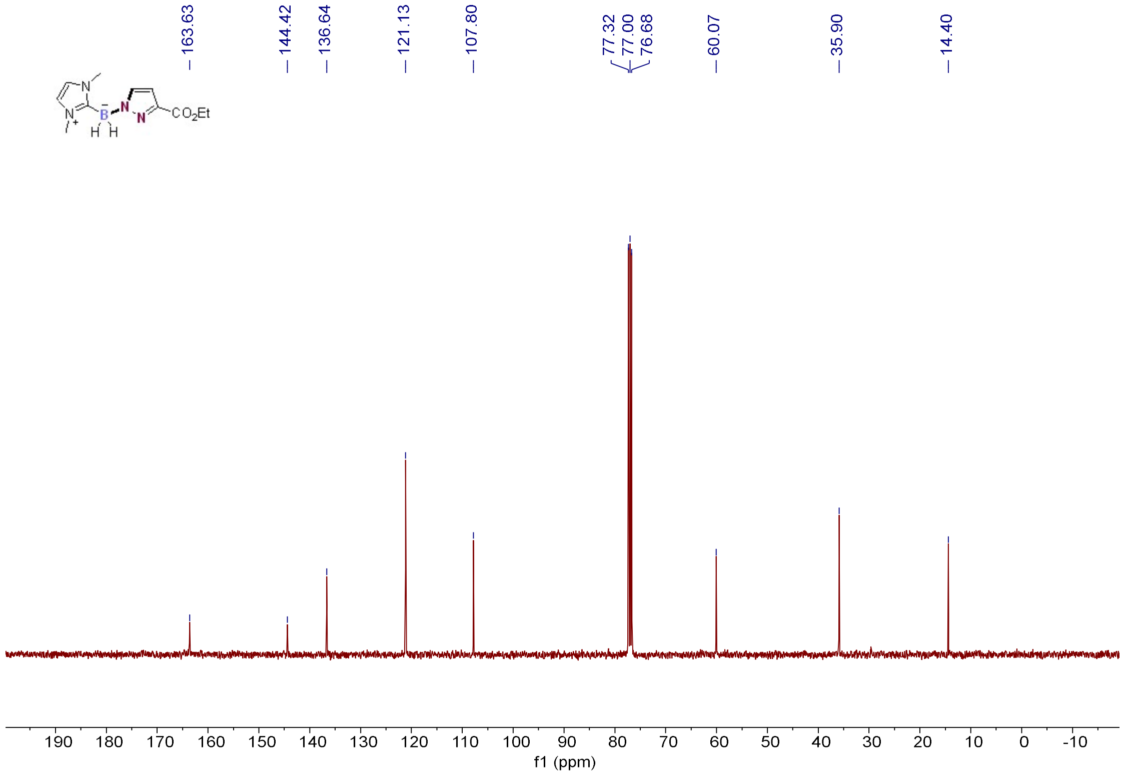

^{11}B NMR (128.4 MHz) Spectrum of 3 in $CDCl_3$

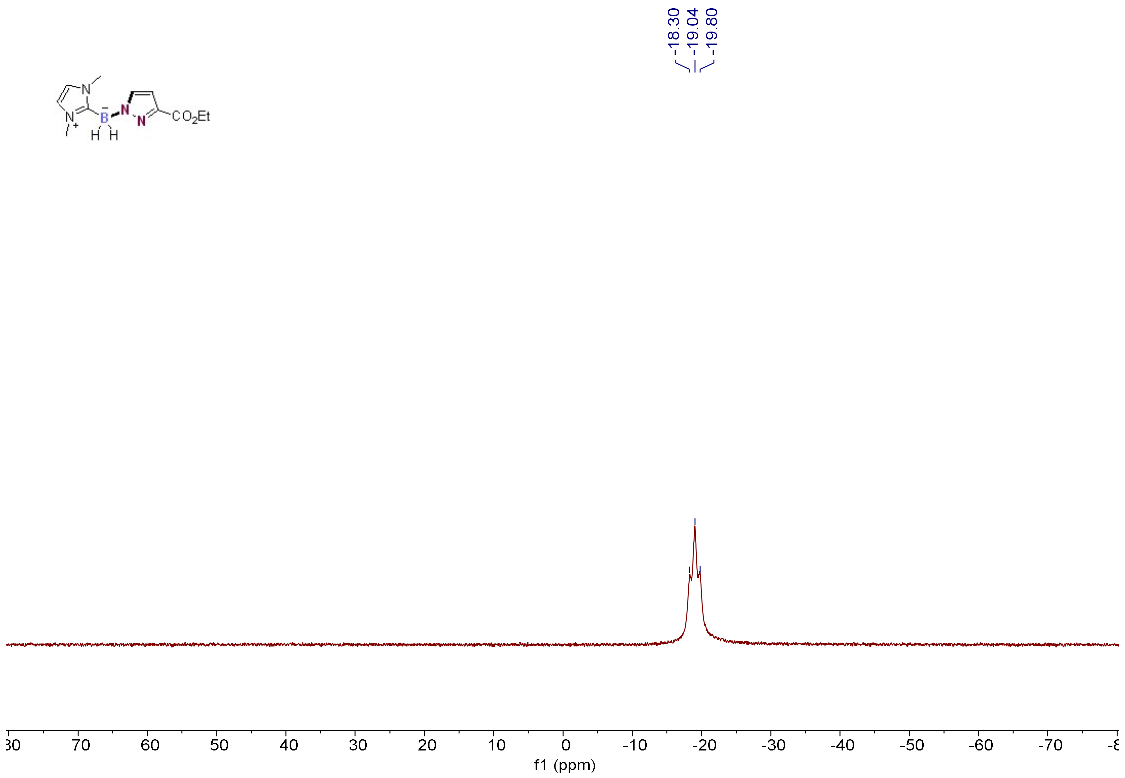

^{1}H NMR (400 MHz) Spectrum of 4 in $CDCl_3$

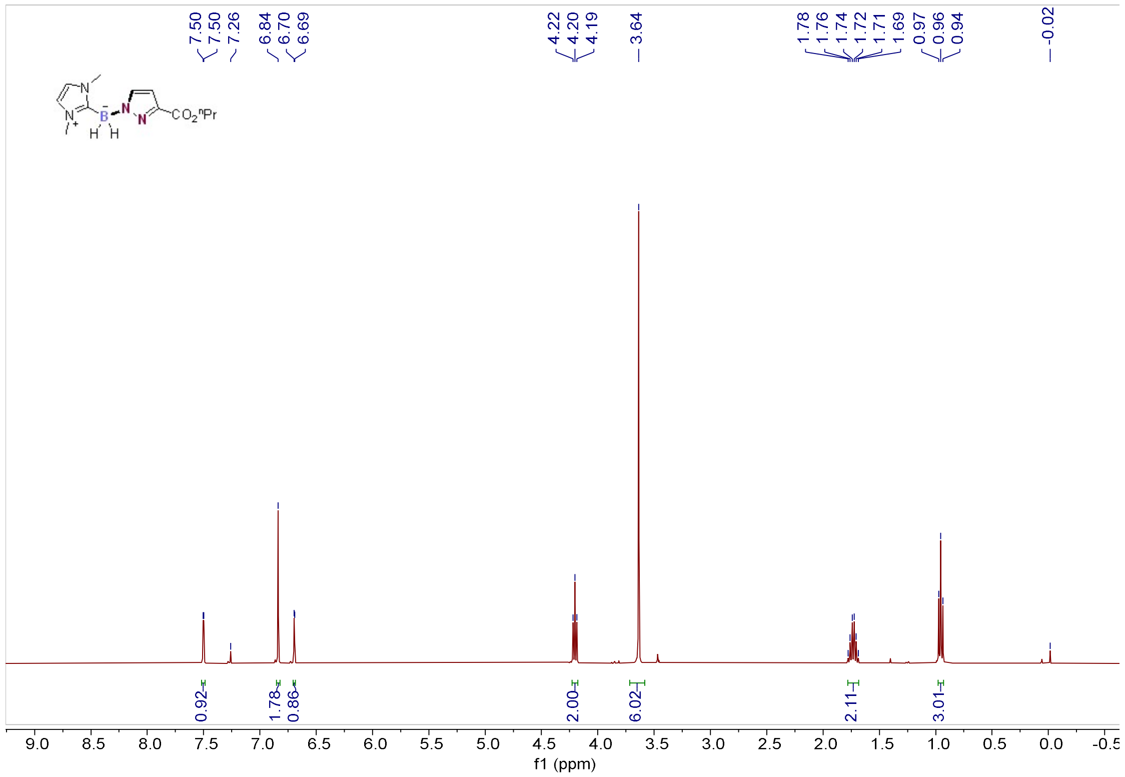

^{13}C NMR (100 MHz) Spectrum of 4 in $CDCl_3$

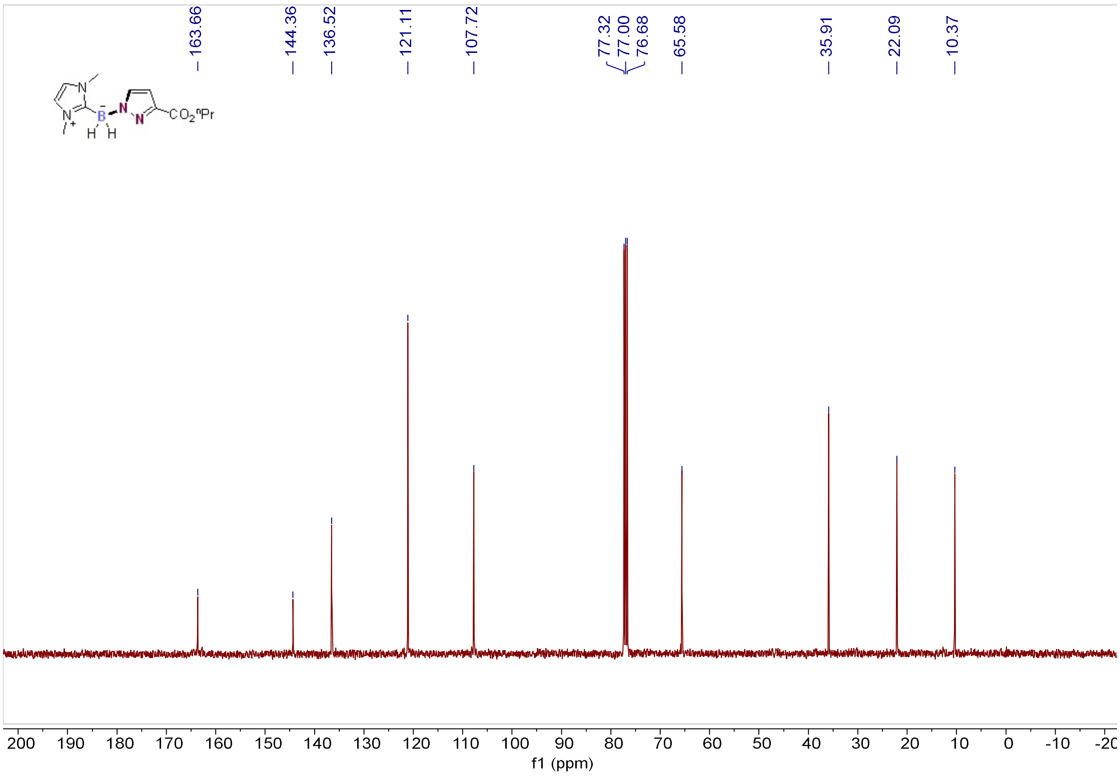

^{11}B NMR (128.4 MHz) Spectrum of 4 in $CDCl_3$

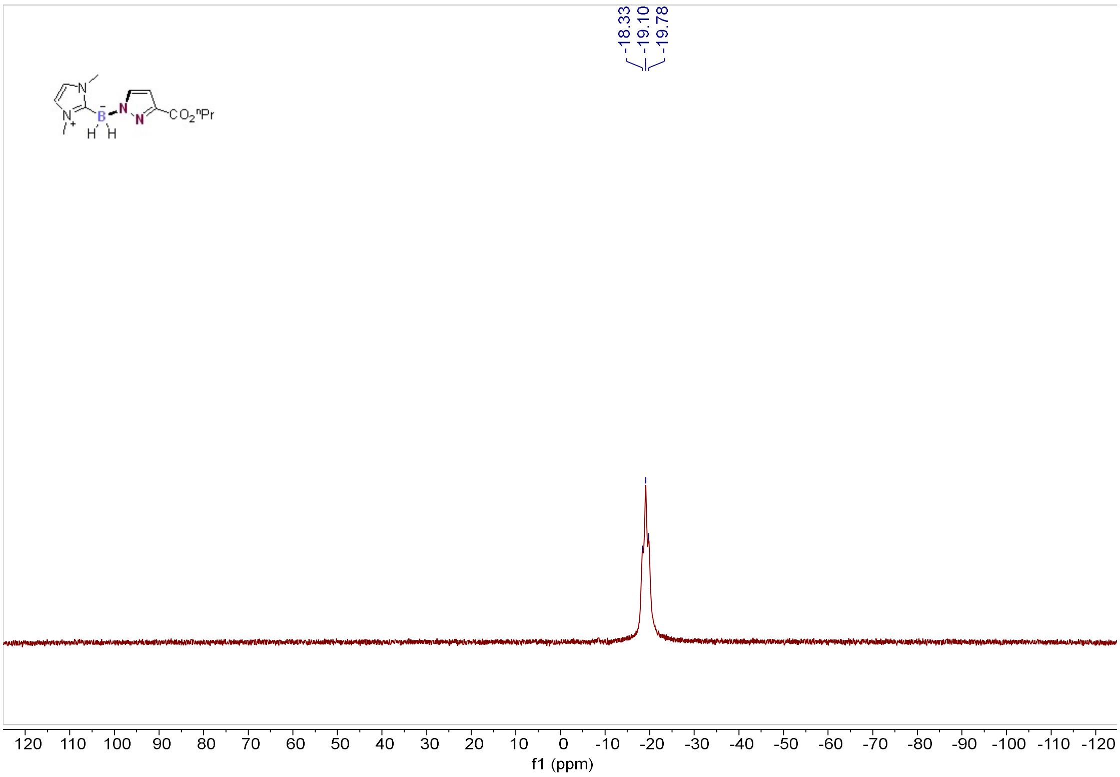

^{1}H NMR (400 MHz) Spectrum of 5 in $CDCl_3$

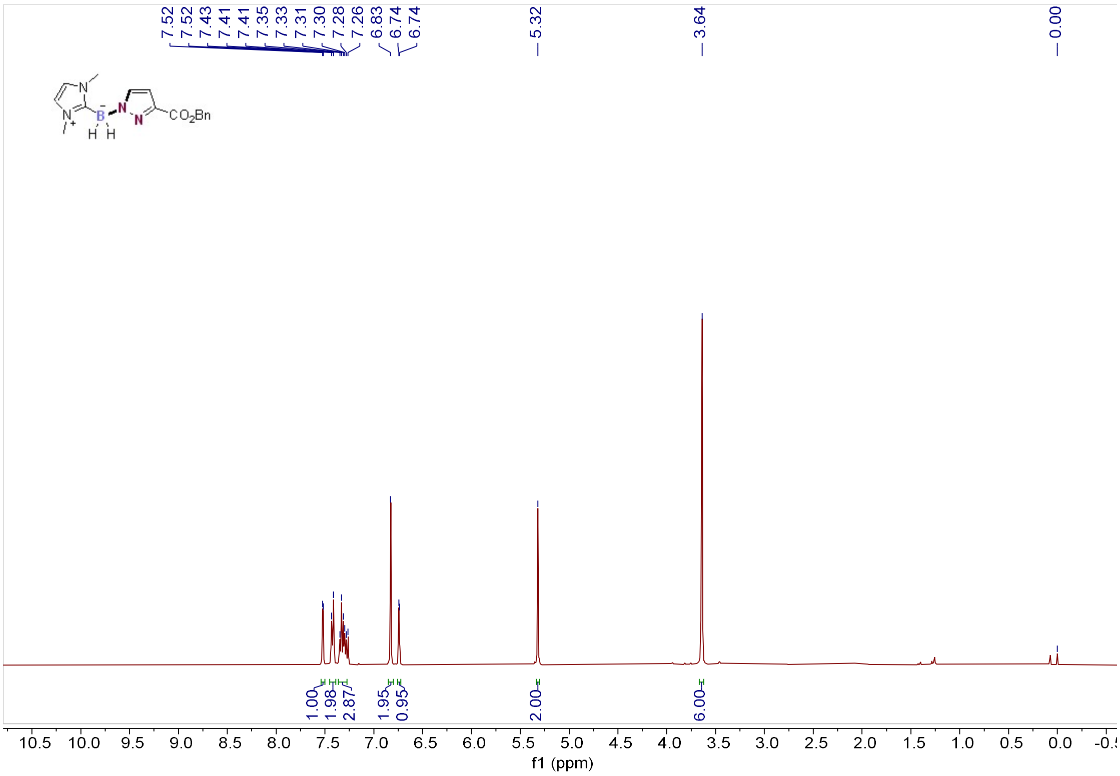

^{13}C NMR (100 MHz) Spectrum of 5 in $CDCl_3$

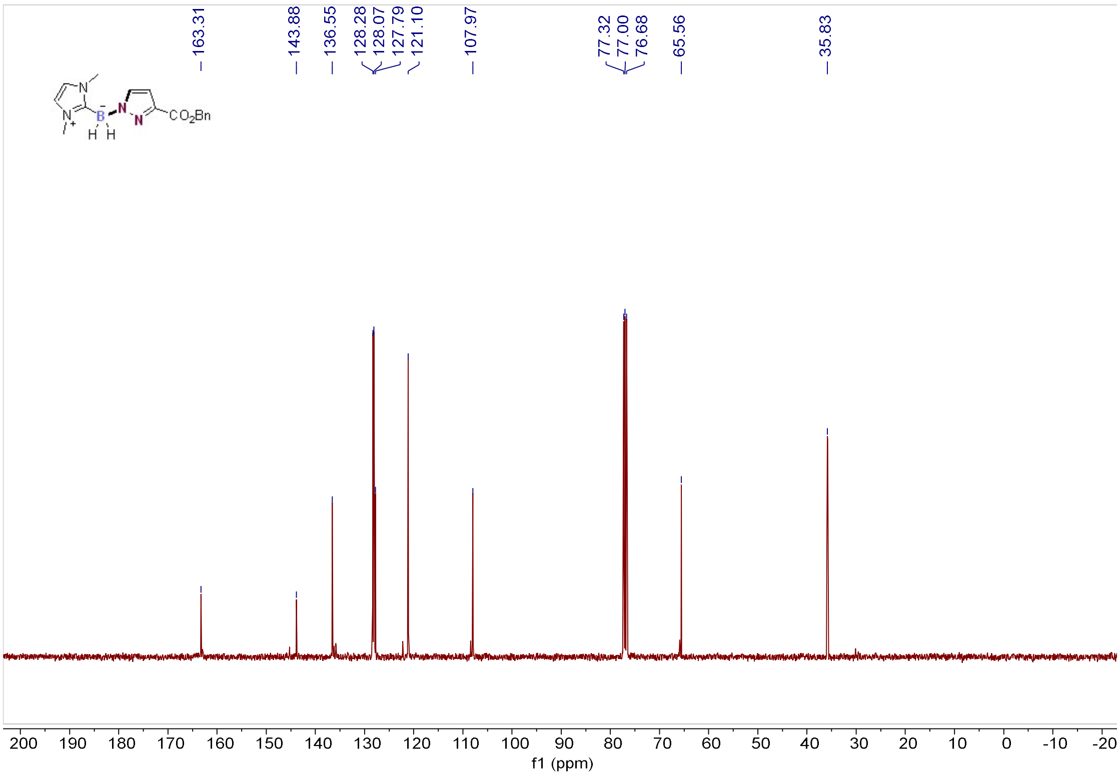

^{11}B NMR (128.4 MHz) Spectrum of 5 in $CDCl_3$

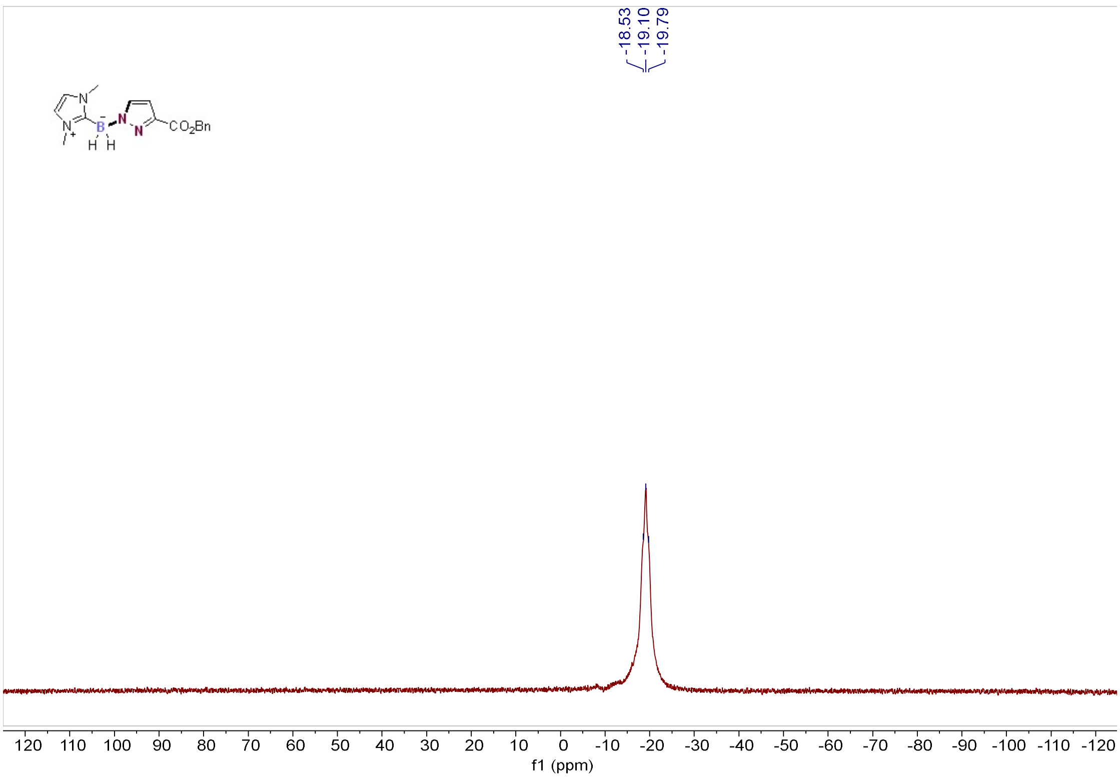

^{1}H NMR (400 MHz) Spectrum of 6 in $CDCl_3$

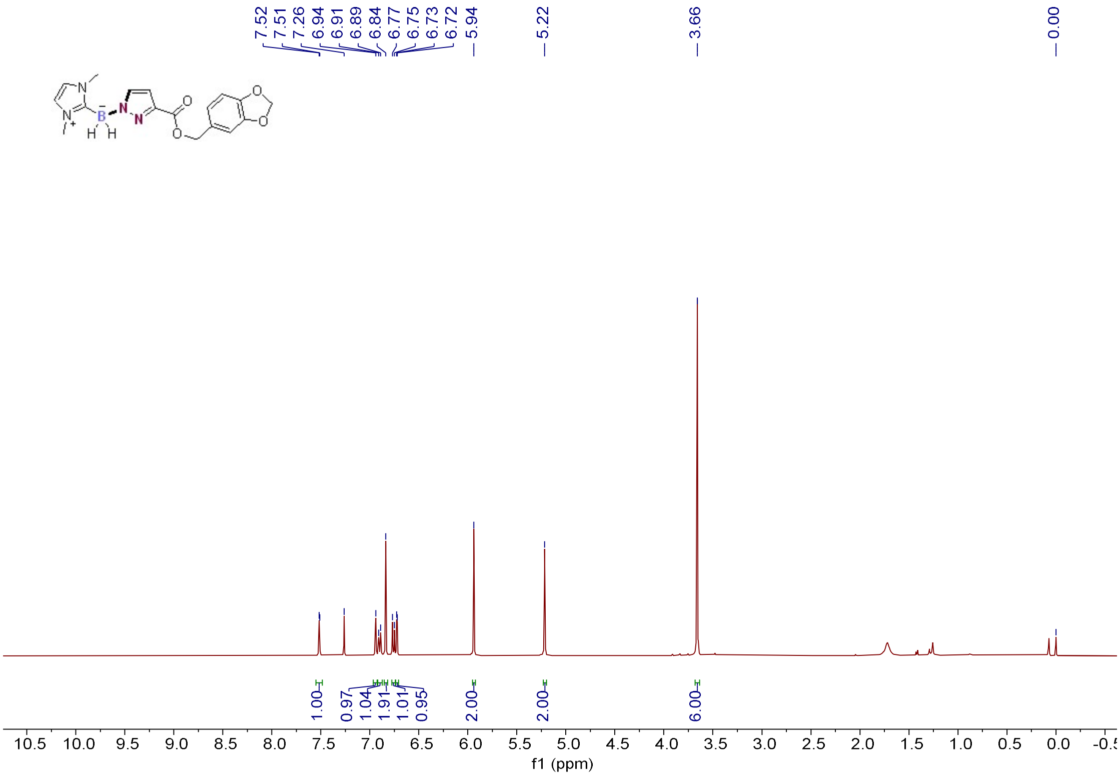

^{13}C NMR (100 MHz) Spectrum of 6 in $CDCl_3$

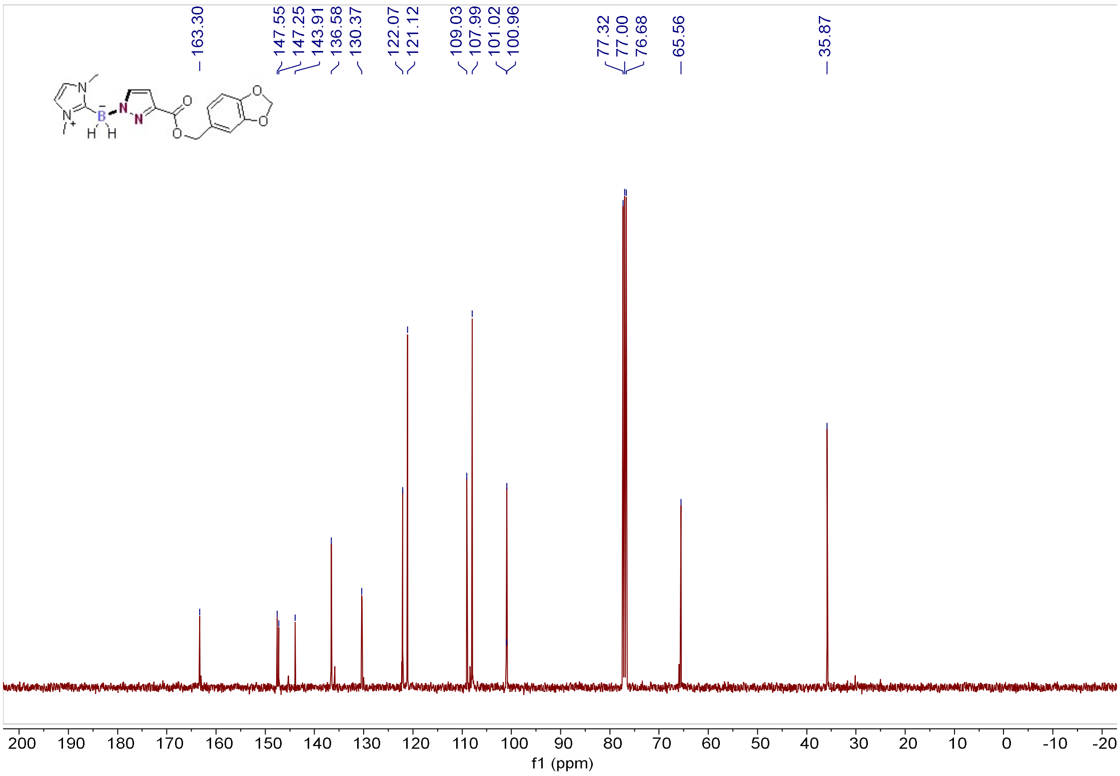

^{11}B NMR (128.4 MHz) Spectrum of 6 in $CDCl_3$

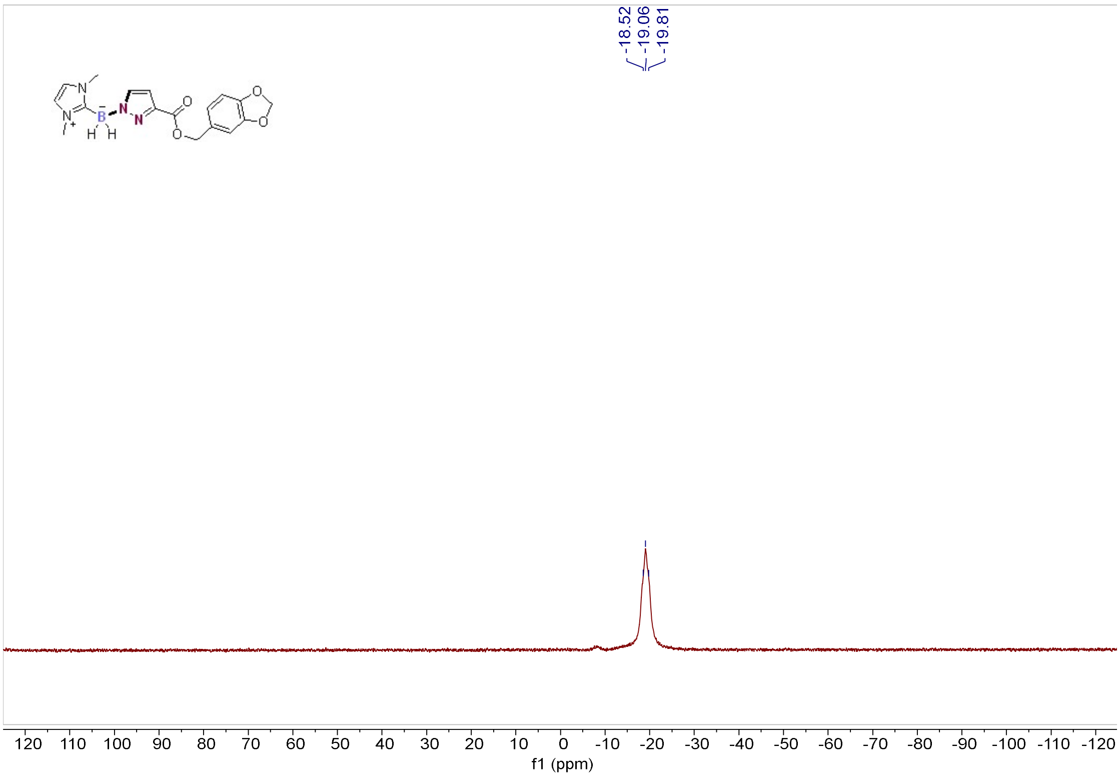

^{1}H NMR (400 MHz) Spectrum of 7 in CDCl₃

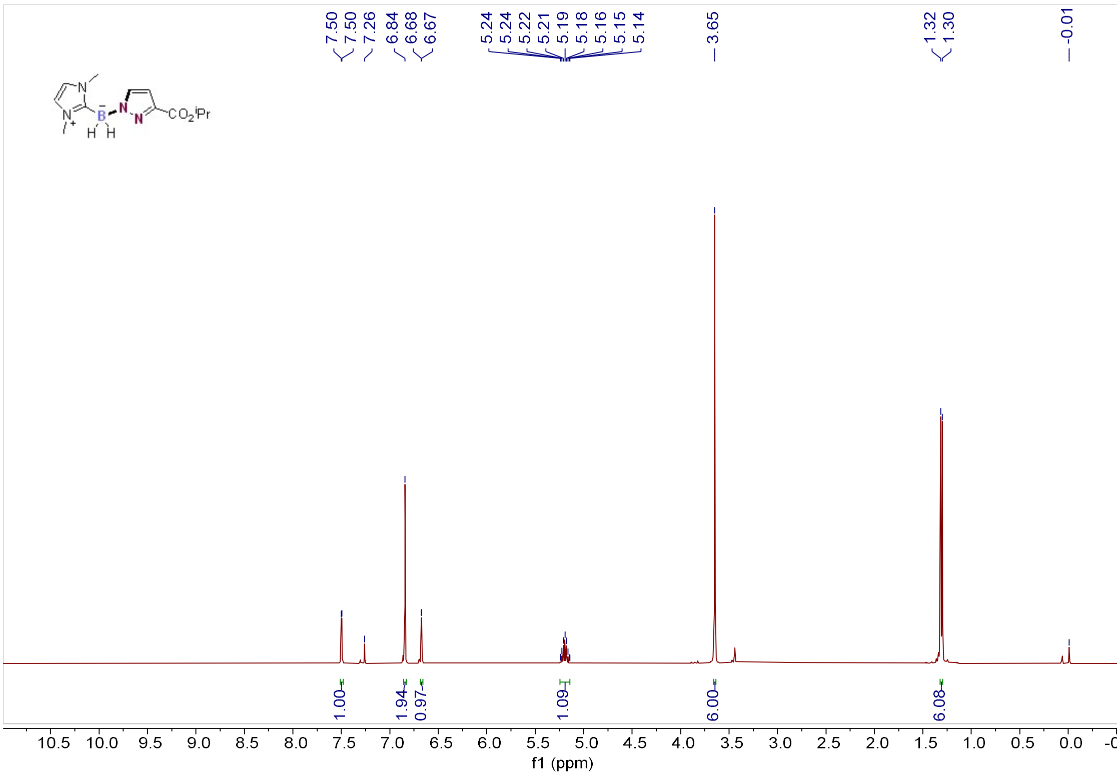

^{13}C NMR (100 MHz) Spectrum of 7 in CDCl₃

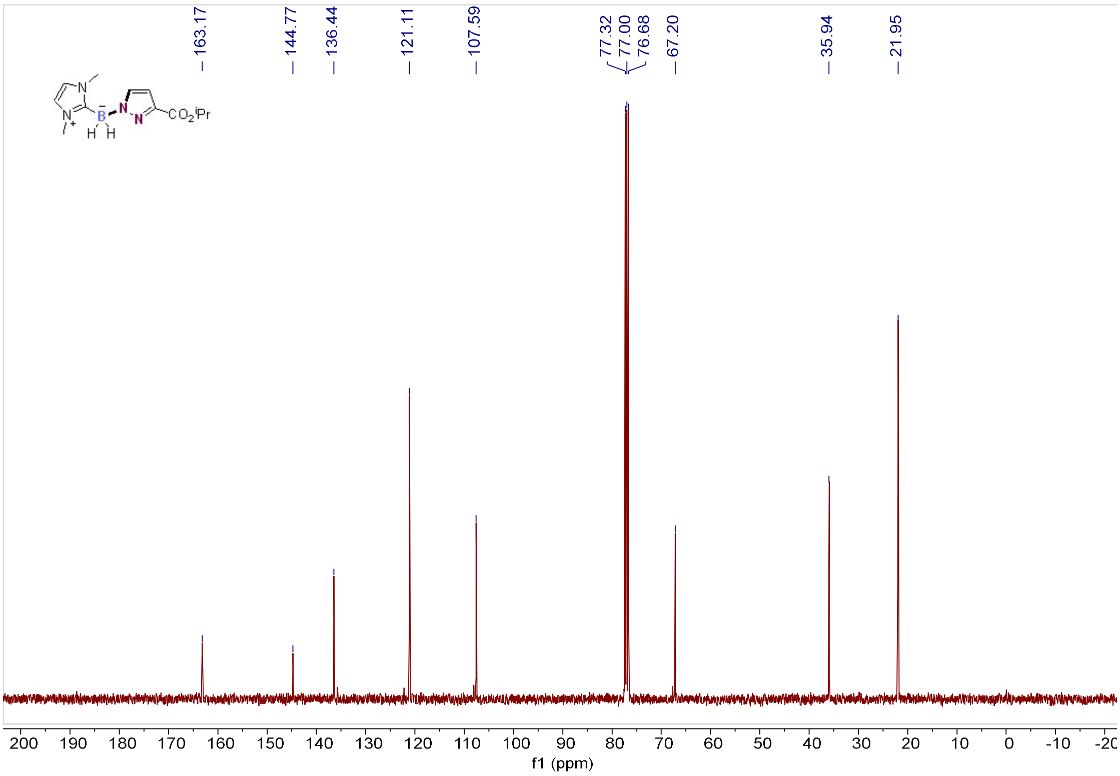

^{11}B NMR (128.4 MHz) Spectrum of 7 in $CDCl_3$

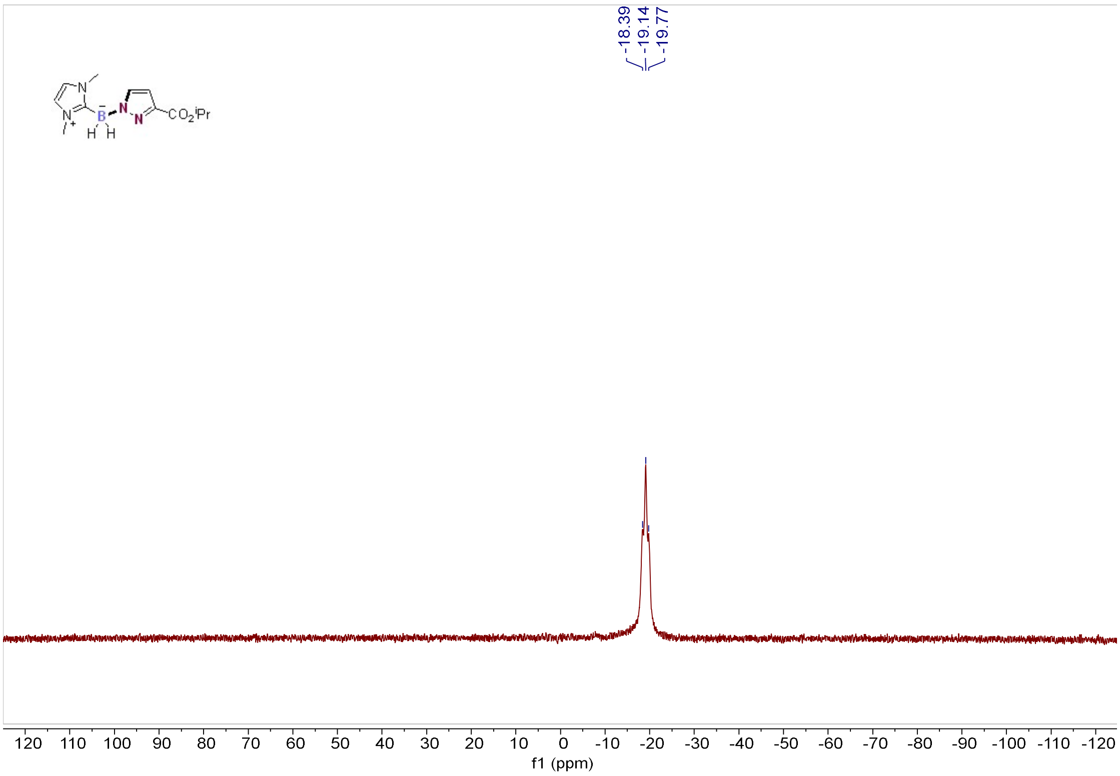

^{1}H NMR (400 MHz) Spectrum of 8 in $CDCl_3$

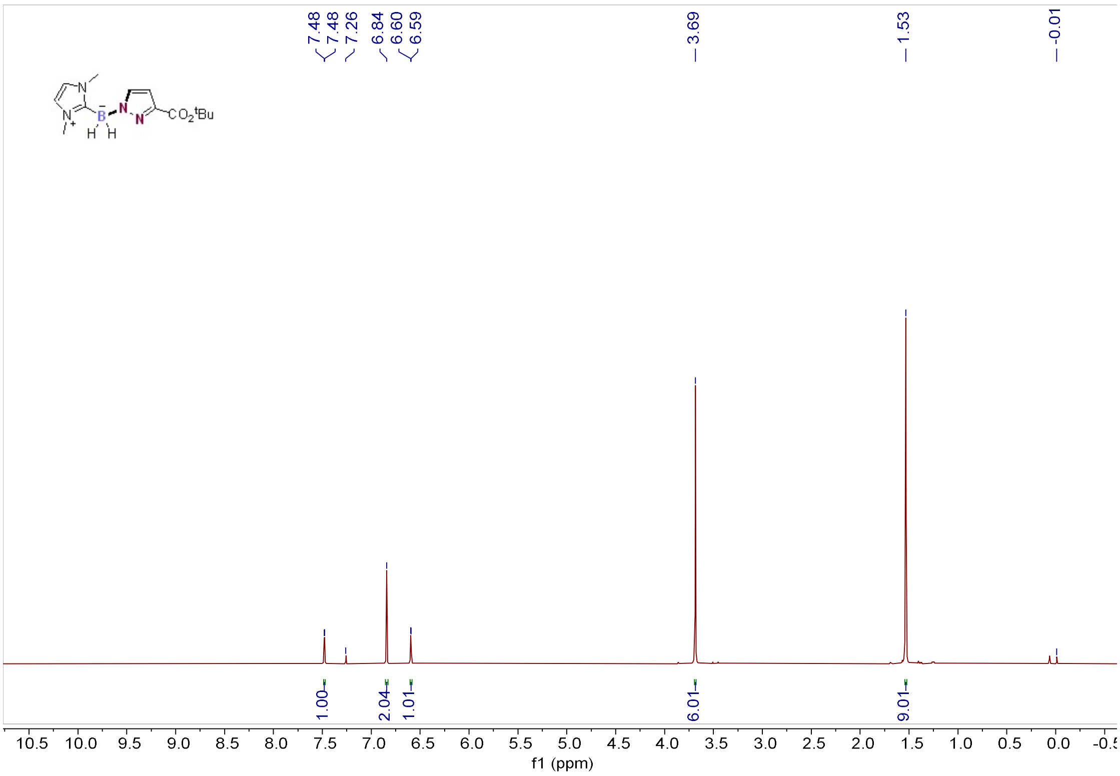

^{13}C NMR (100 MHz) Spectrum of 8 in $CDCl_3$

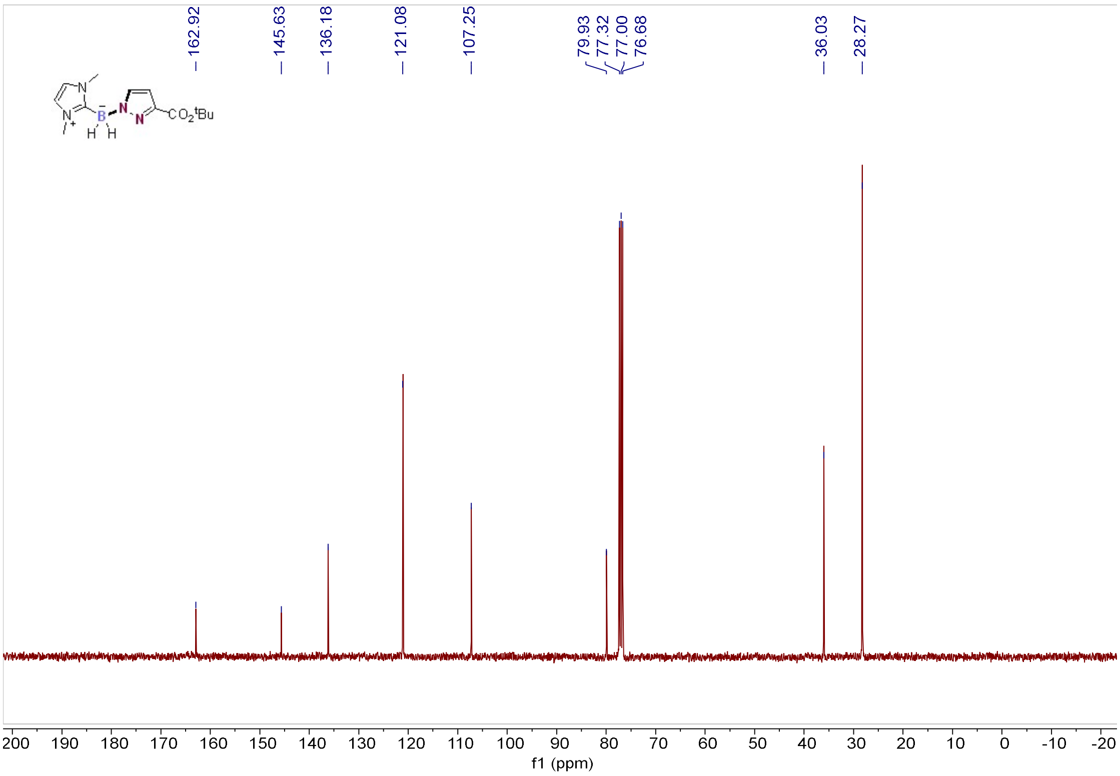

^{11}B NMR (128.4 MHz) Spectrum of 8 in $CDCl_3$

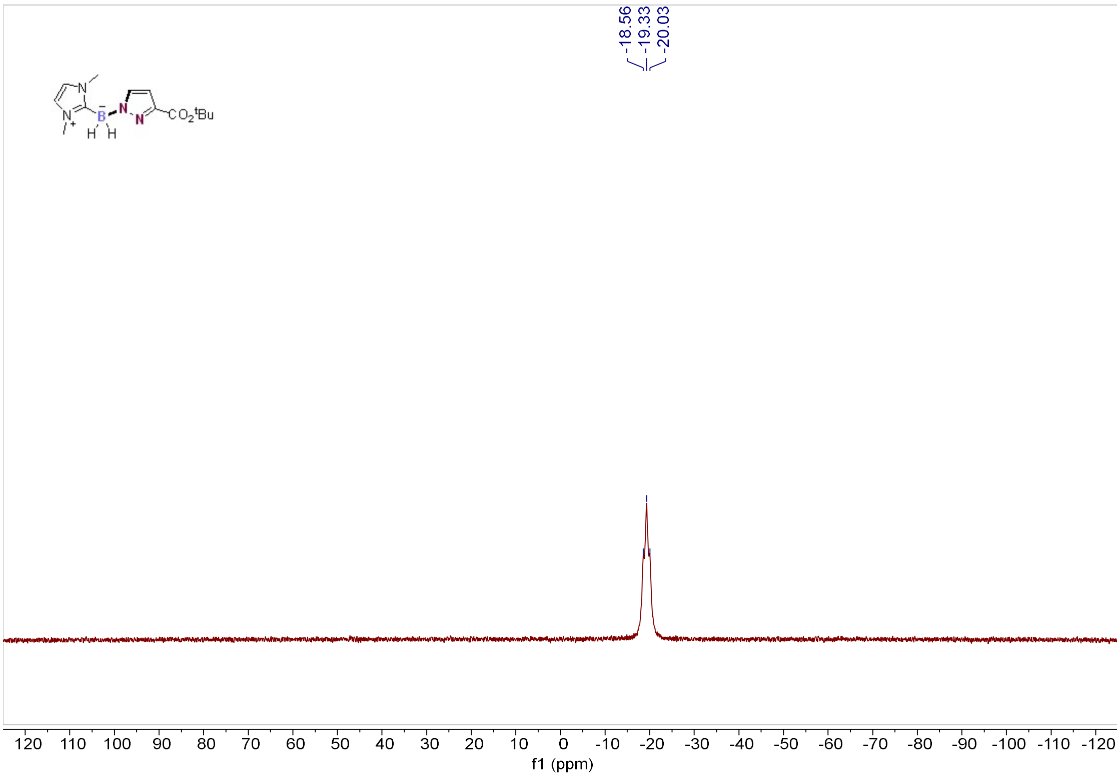

^{1}H NMR (400 MHz) Spectrum of 9 in $CDCl_3$

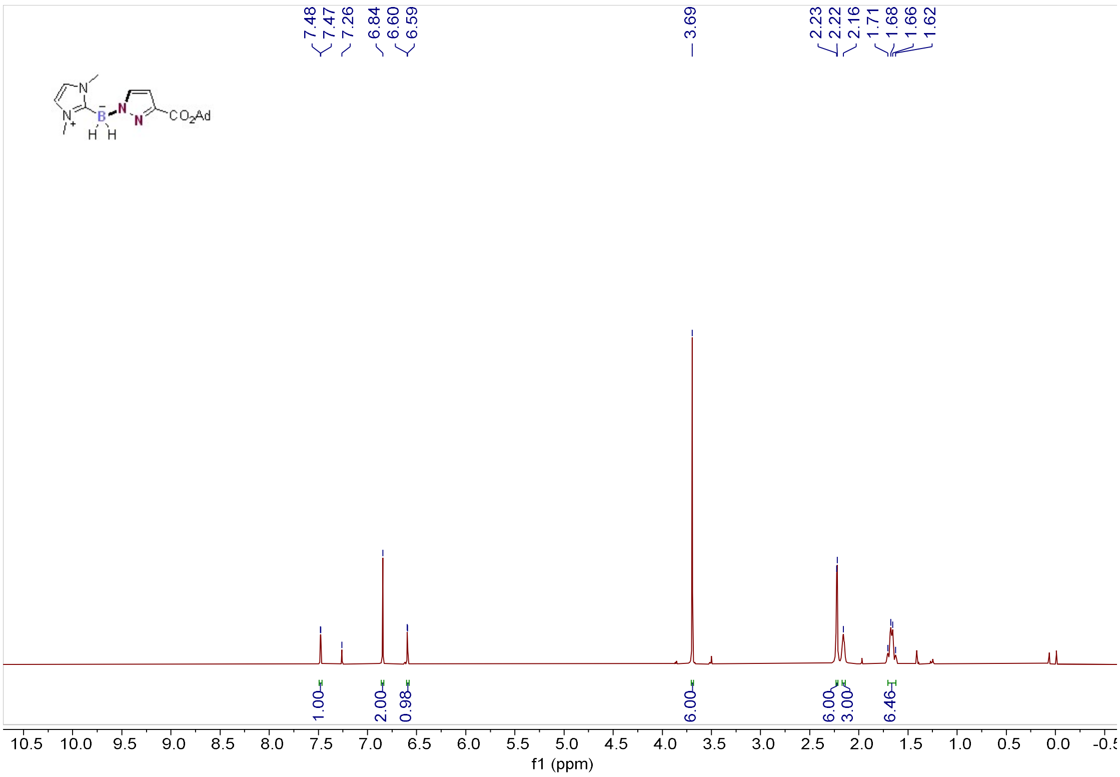

^{13}C NMR (100 MHz) Spectrum of 9 in $CDCl_3$

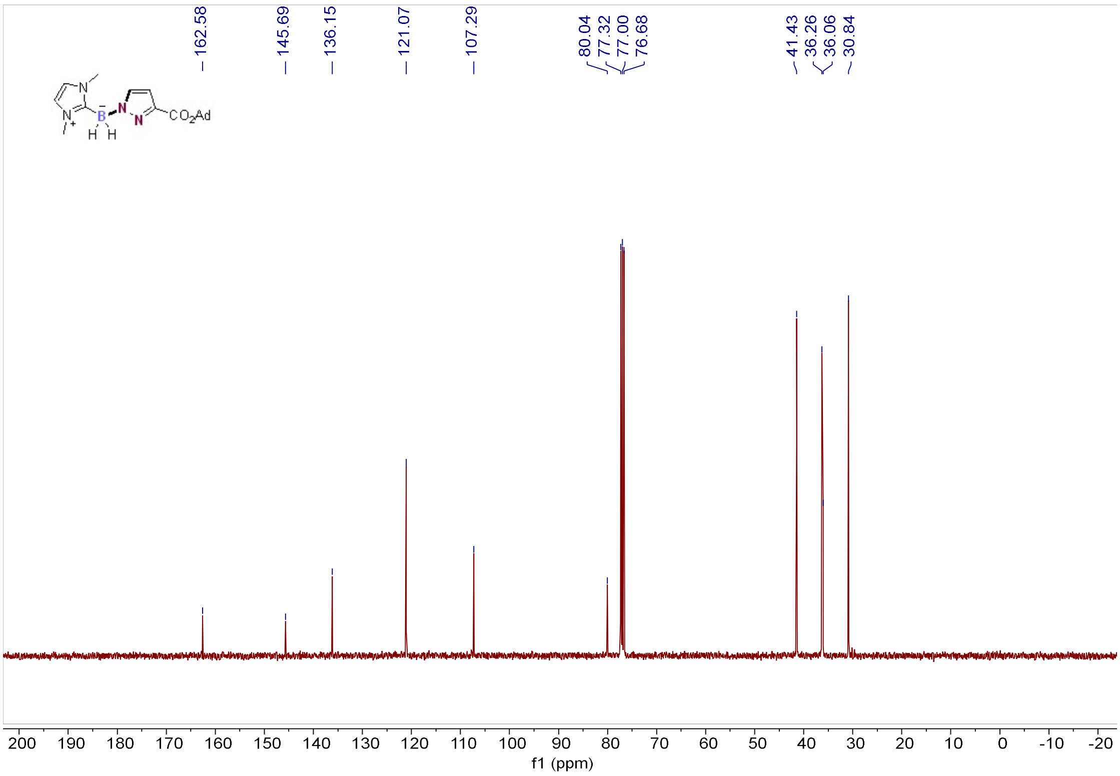

^{11}B NMR (128.4 MHz) Spectrum of 9 in CDCl$_3$

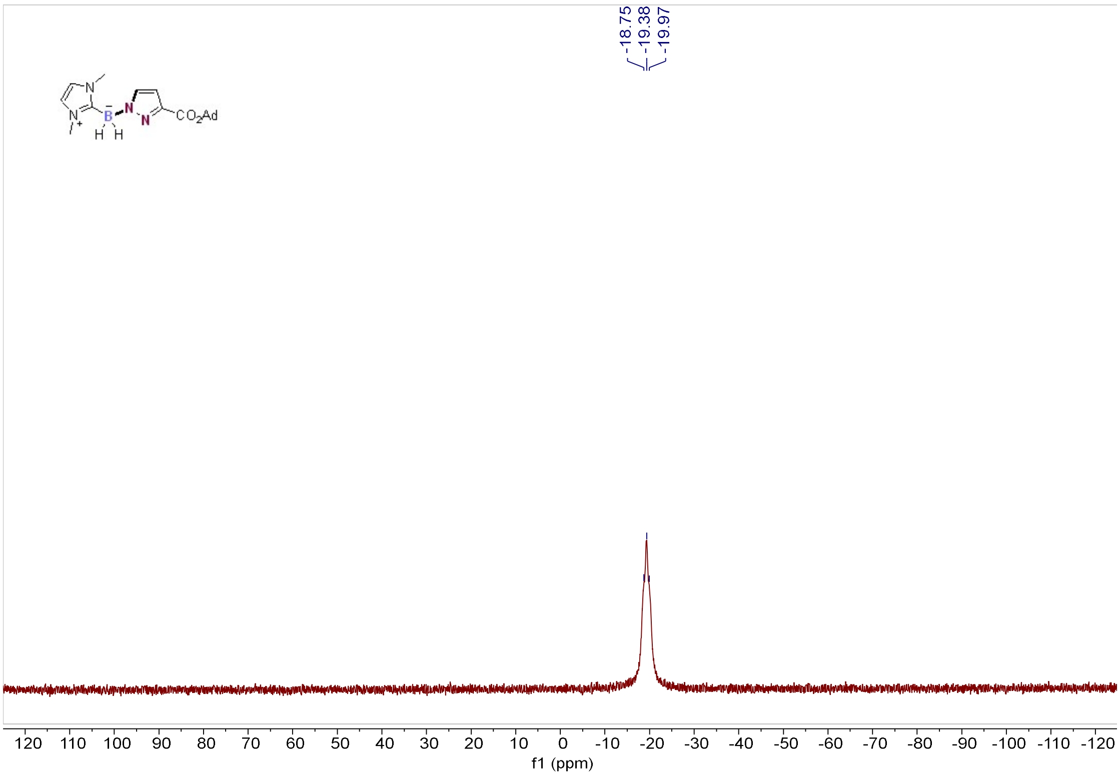

^{1}H NMR (400 MHz) Spectrum of 10 in CDCl$_3$

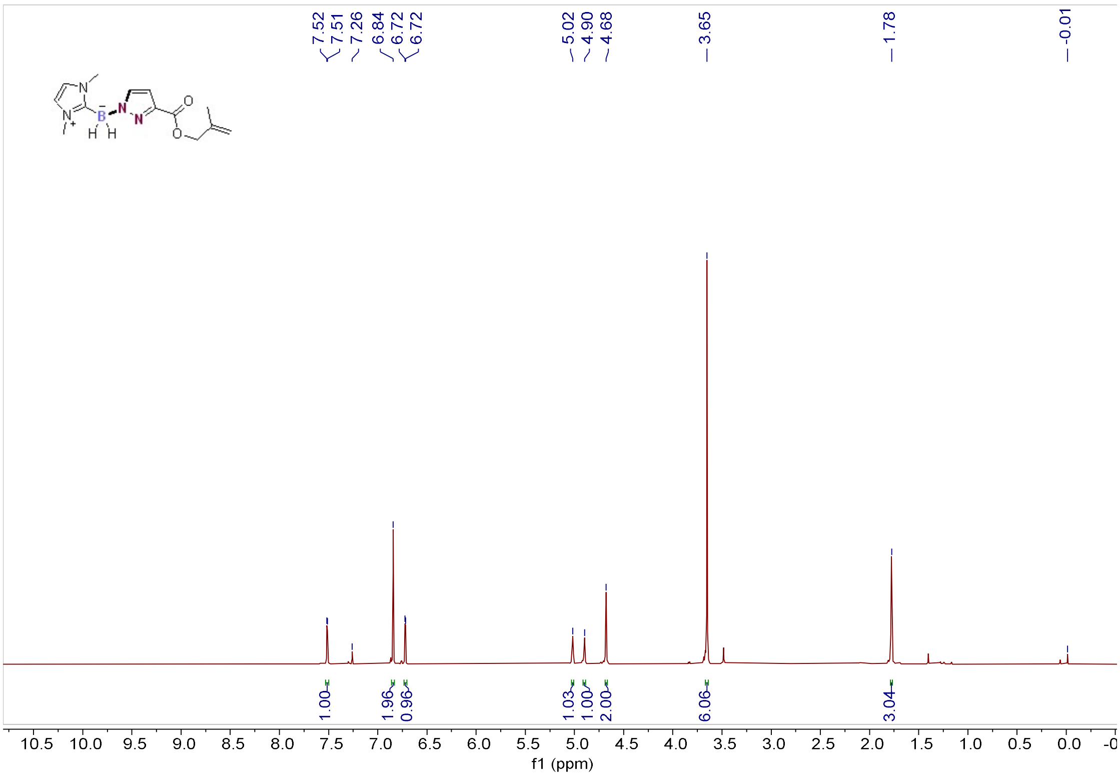

^{13}C NMR (100 MHz) Spectrum of 10 in $CDCl_3$

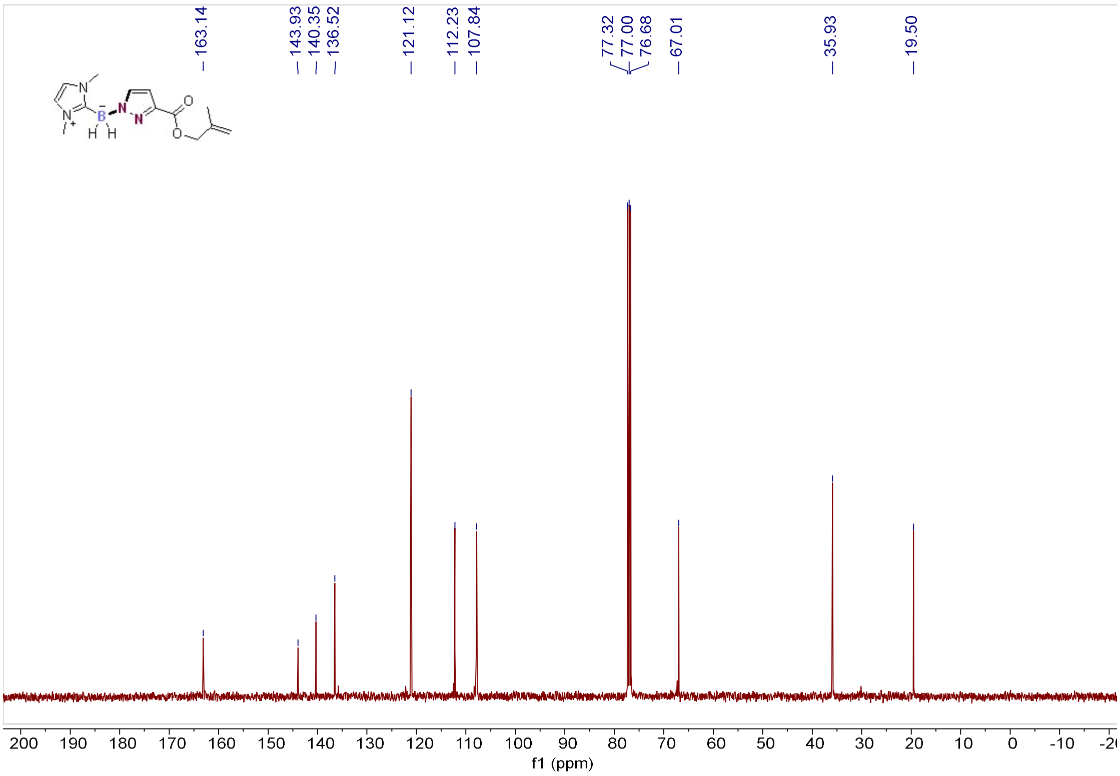

^{11}B NMR (128.4 MHz) Spectrum of 10 in $CDCl_3$

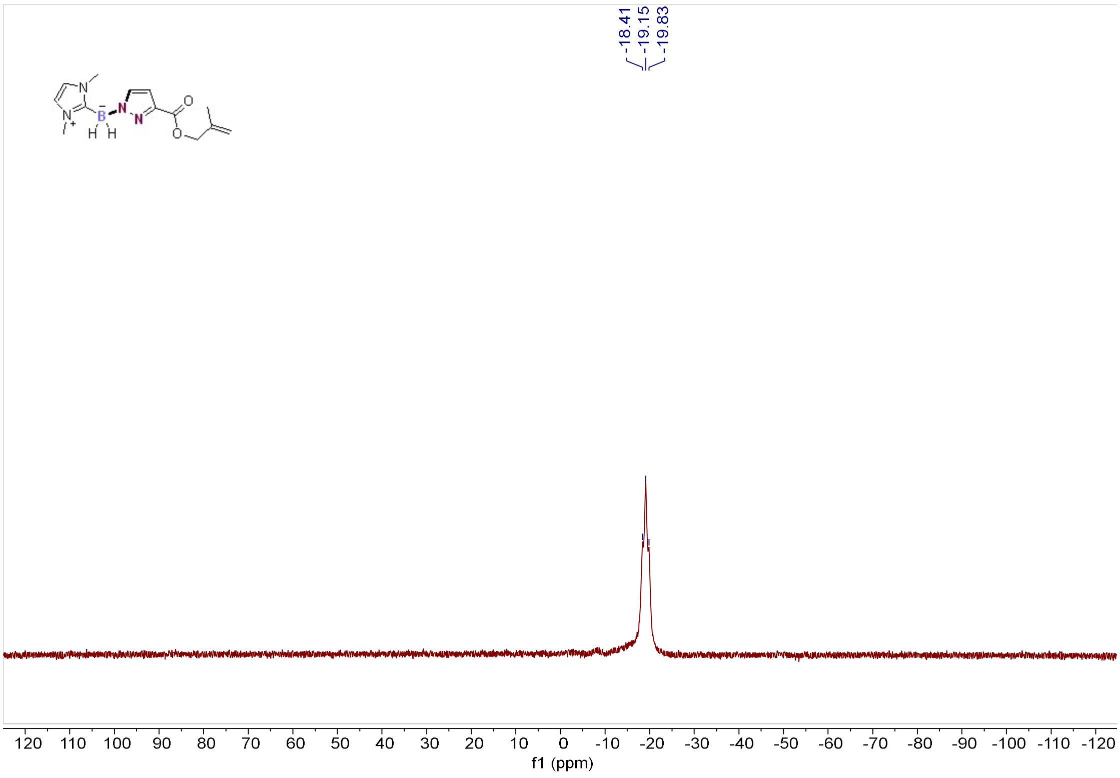

^{1}H NMR (400 MHz) Spectrum of 11 in $CDCl_3$

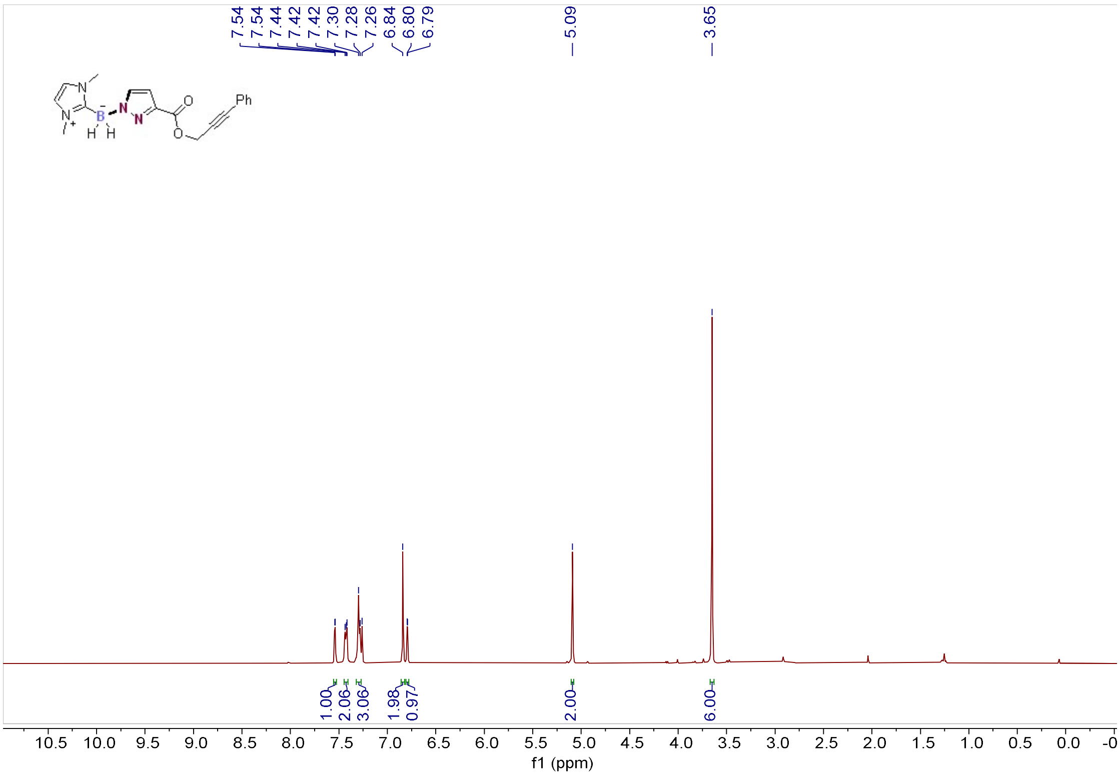

^{13}C NMR (100 MHz) Spectrum of 11 in $CDCl_3$

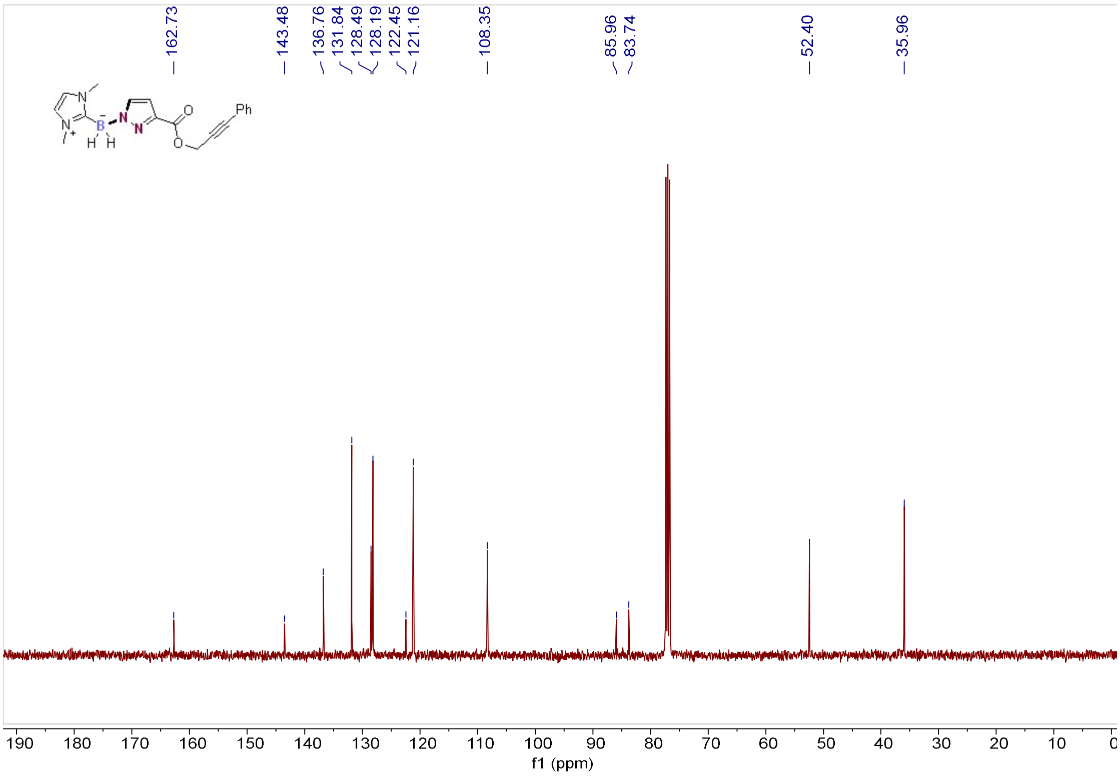

^{11}B NMR (128.4 MHz) Spectrum of 11 in $CDCl_3$

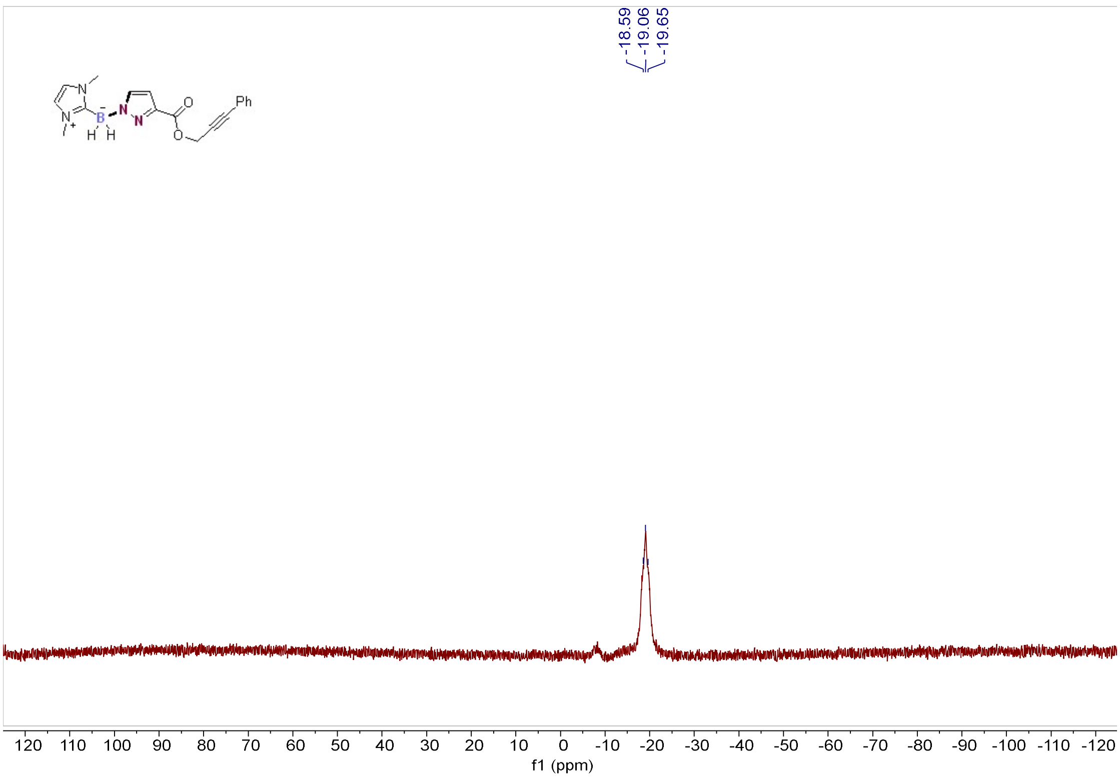

^{1}H NMR (400 MHz) Spectrum of 12 in $CDCl_3$

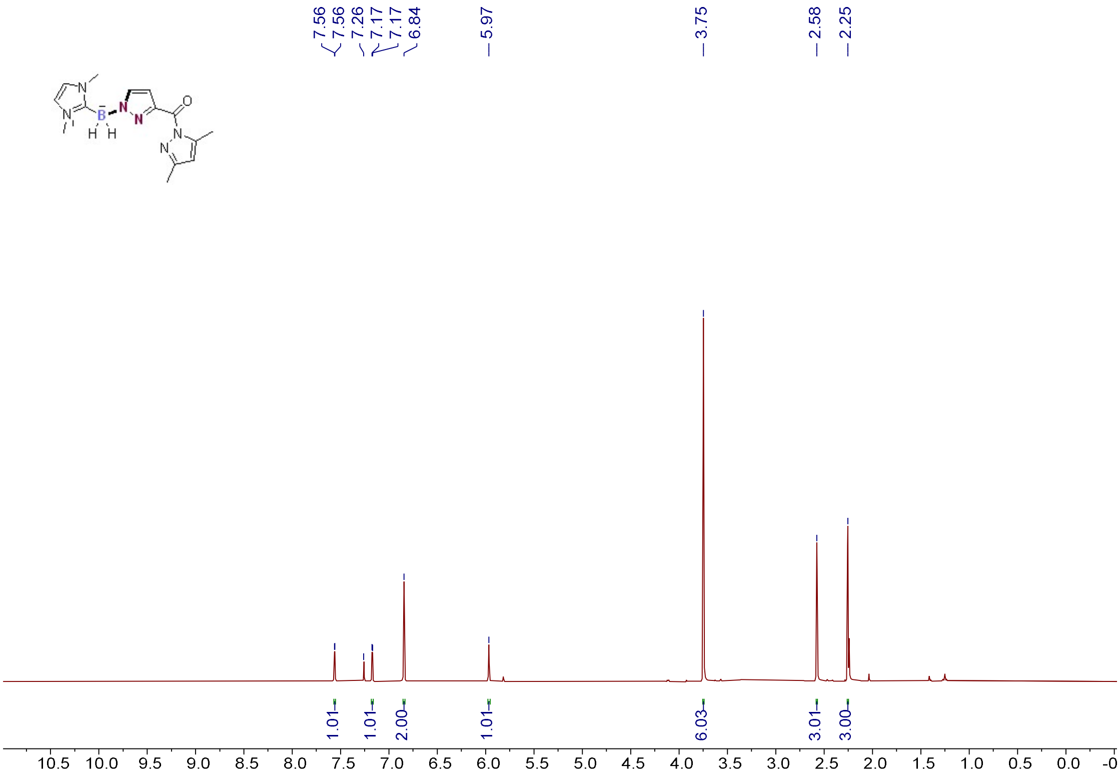

^{13}C NMR (100 MHz) Spectrum of 12 in $CDCl_3$

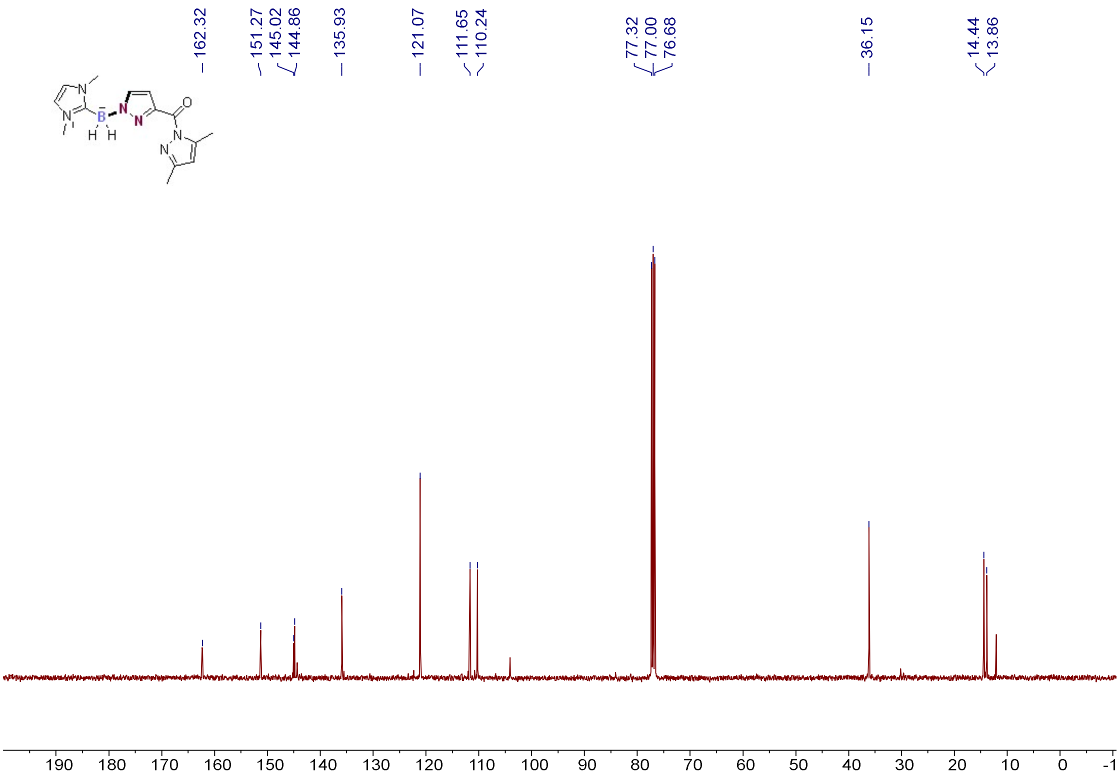

^{11}B NMR (128.4 MHz) Spectrum of 12 in $CDCl_3$

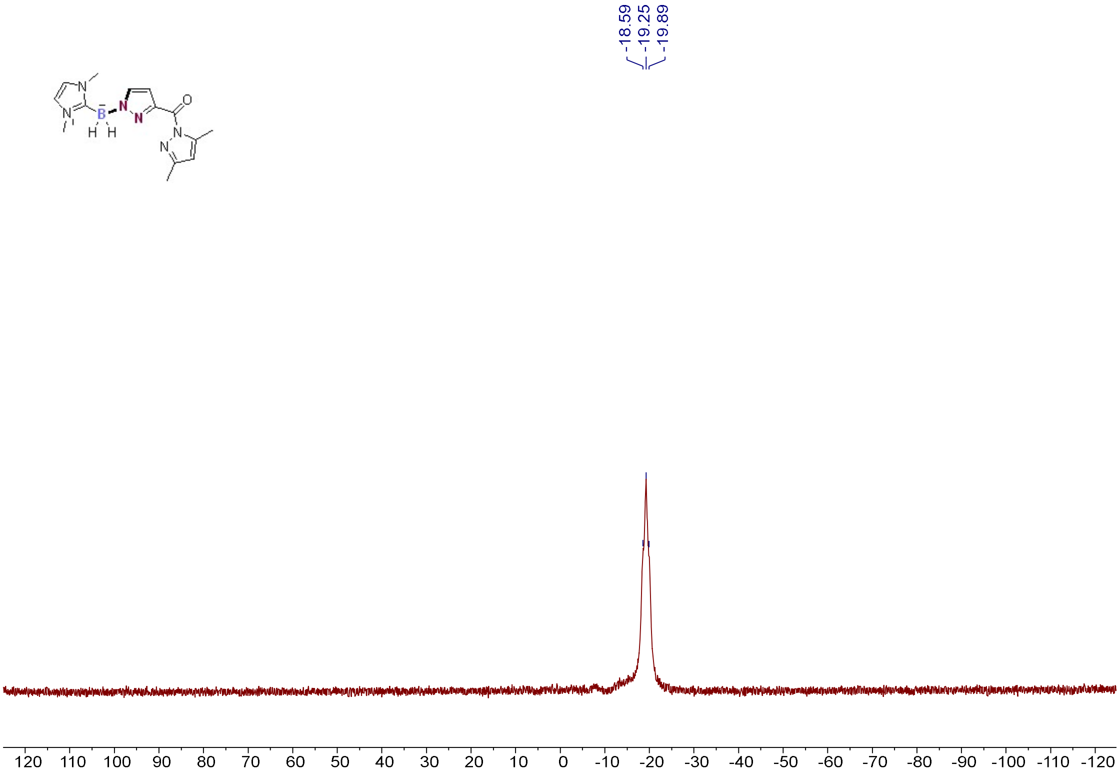

^{1}H NMR (400 MHz) Spectrum of 13 in $CDCl_3$

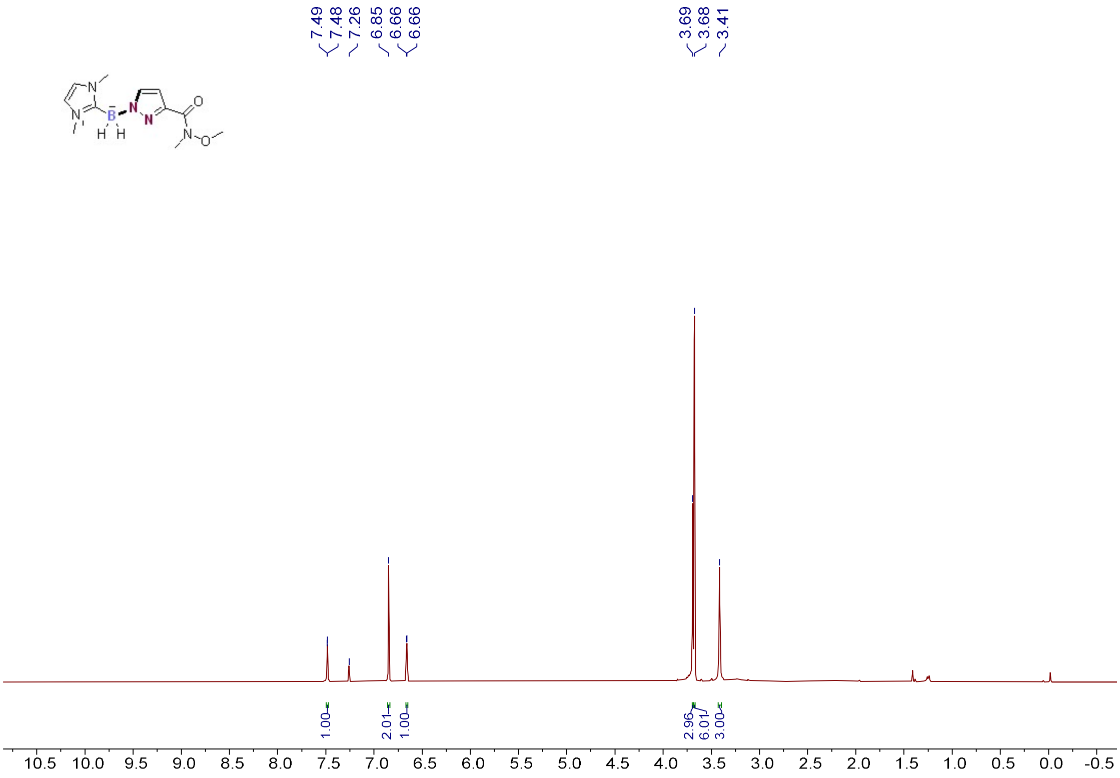

^{13}C NMR (100 MHz) Spectrum of 13 in $CDCl_3$

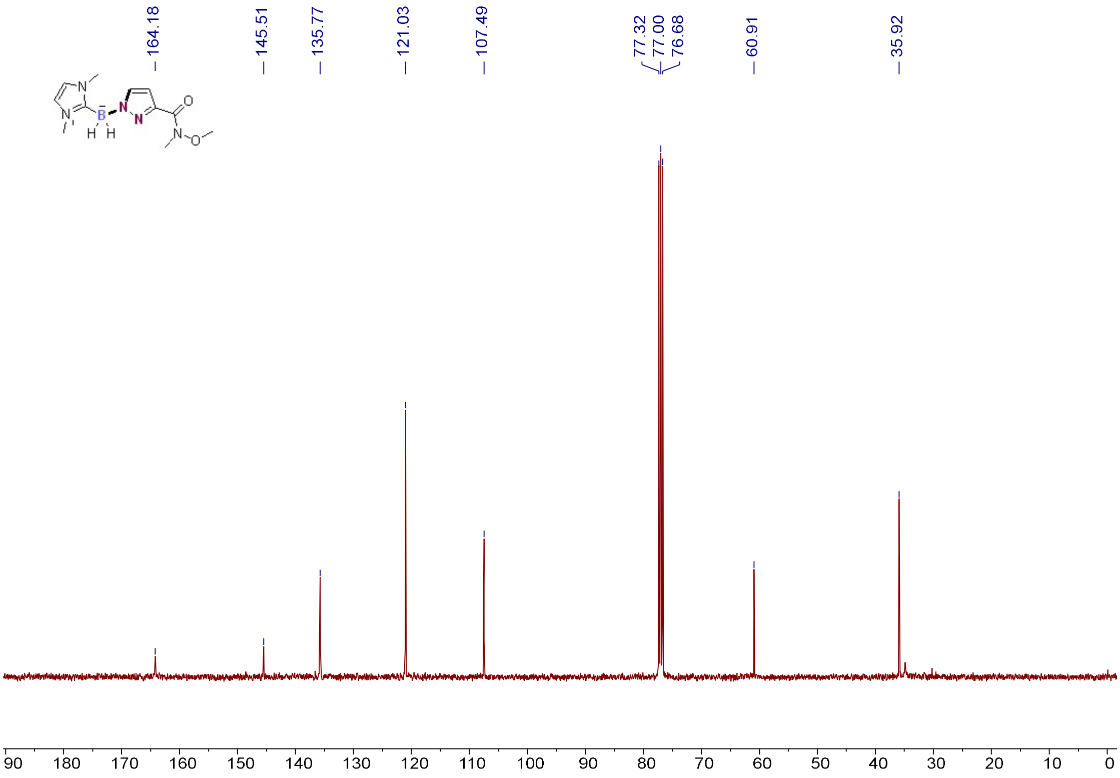

^{11}B NMR (128.4 MHz) Spectrum of 13 in $CDCl_3$

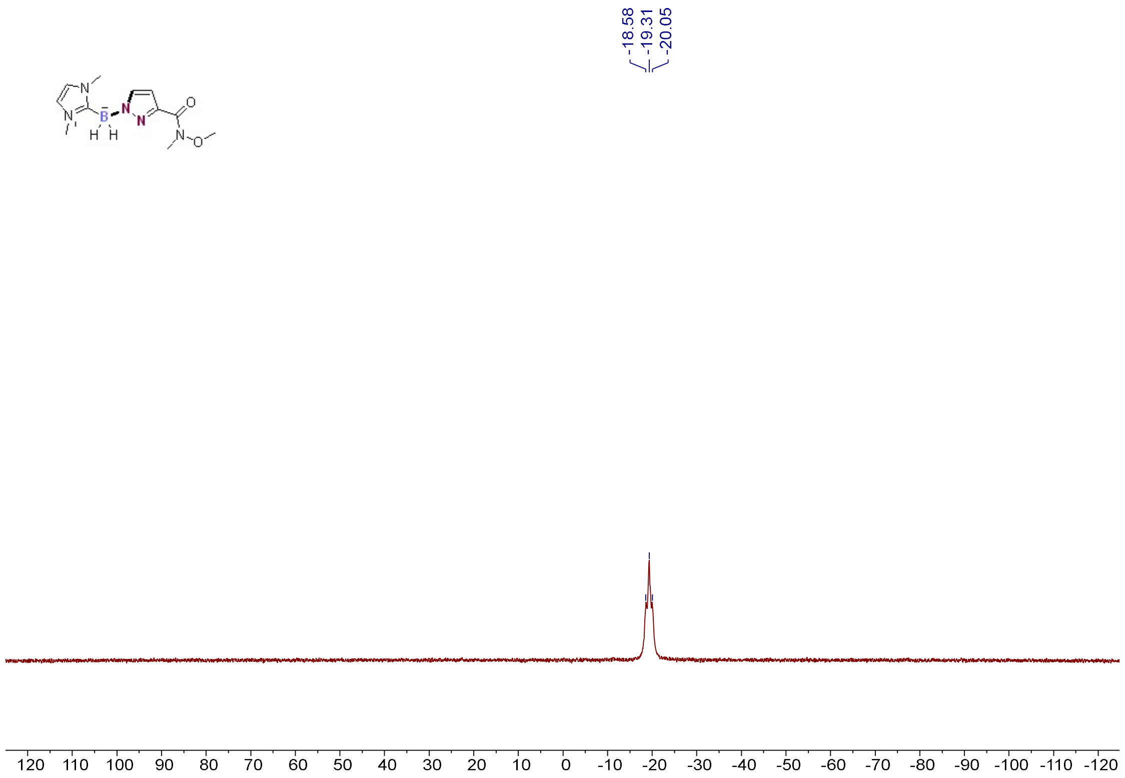

^{1}H NMR (400 MHz) Spectrum of 14 in $CDCl_3$

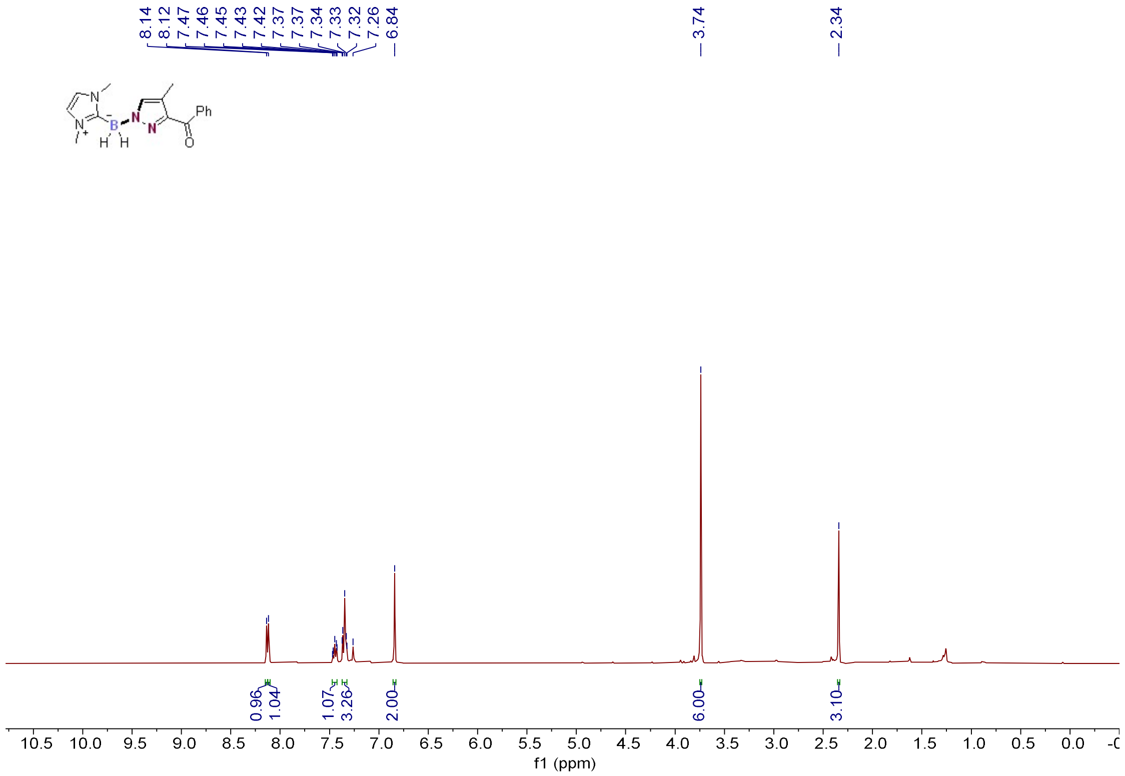

^{13}C NMR (100 MHz) Spectrum of 14 in $CDCl_3$

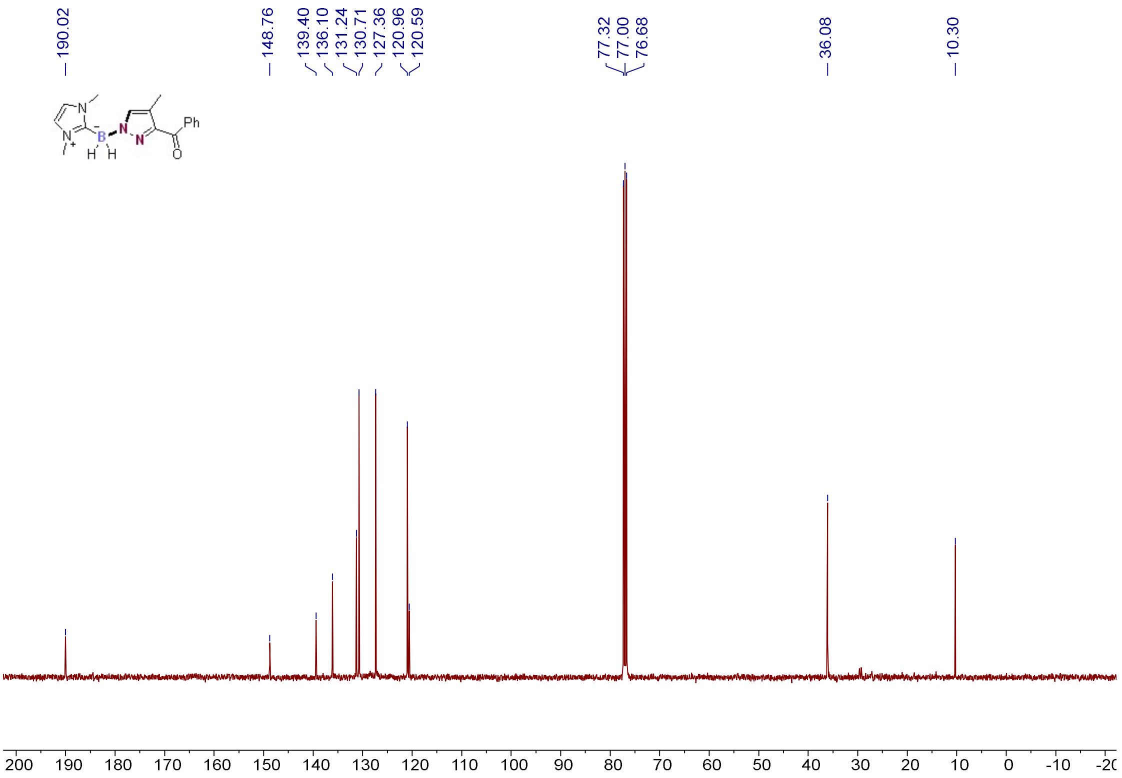

^{11}B NMR (128.4 MHz) Spectrum of 14 in $CDCl_3$

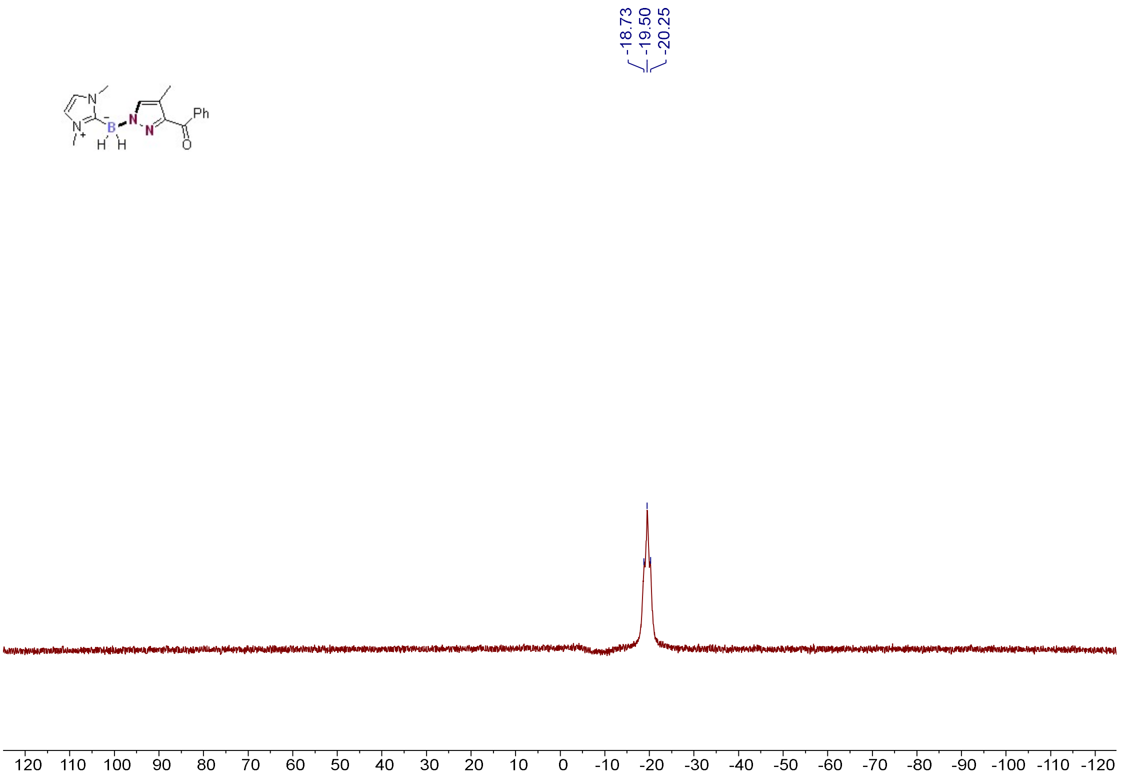

^{1}H NMR (400 MHz) Spectrum of 15 in $CDCl_3$

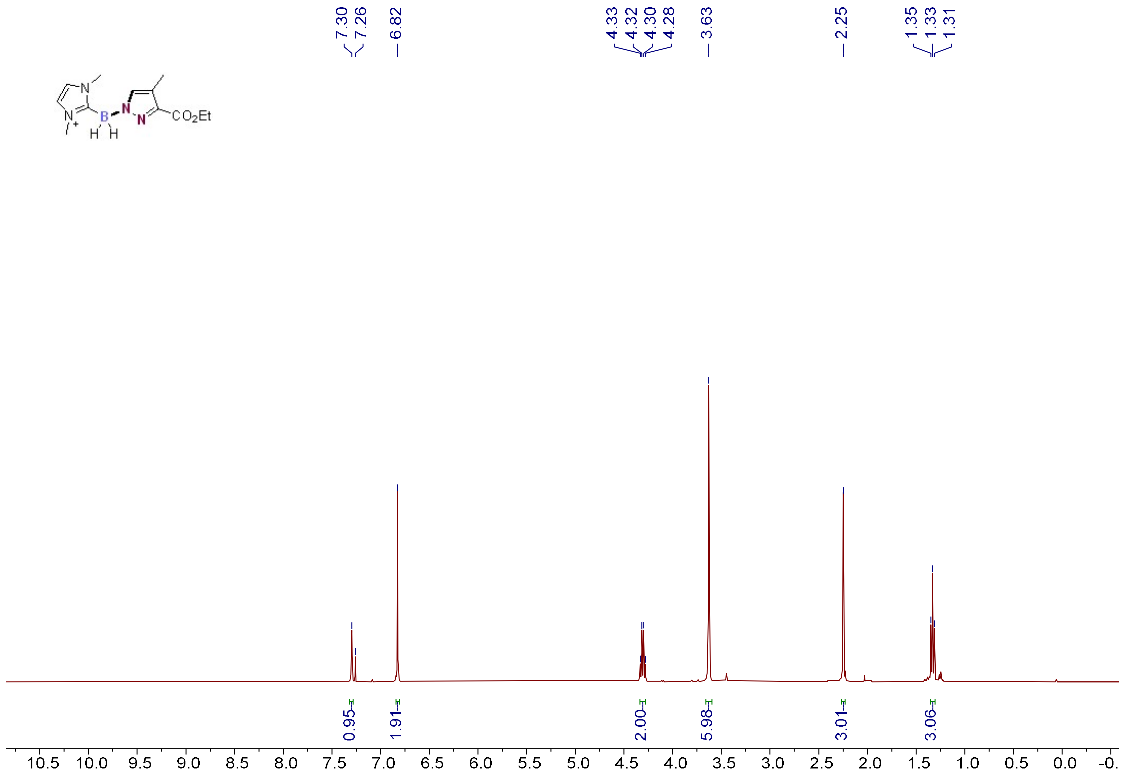

^{13}C NMR (100 MHz) Spectrum of 15 in $CDCl_3$

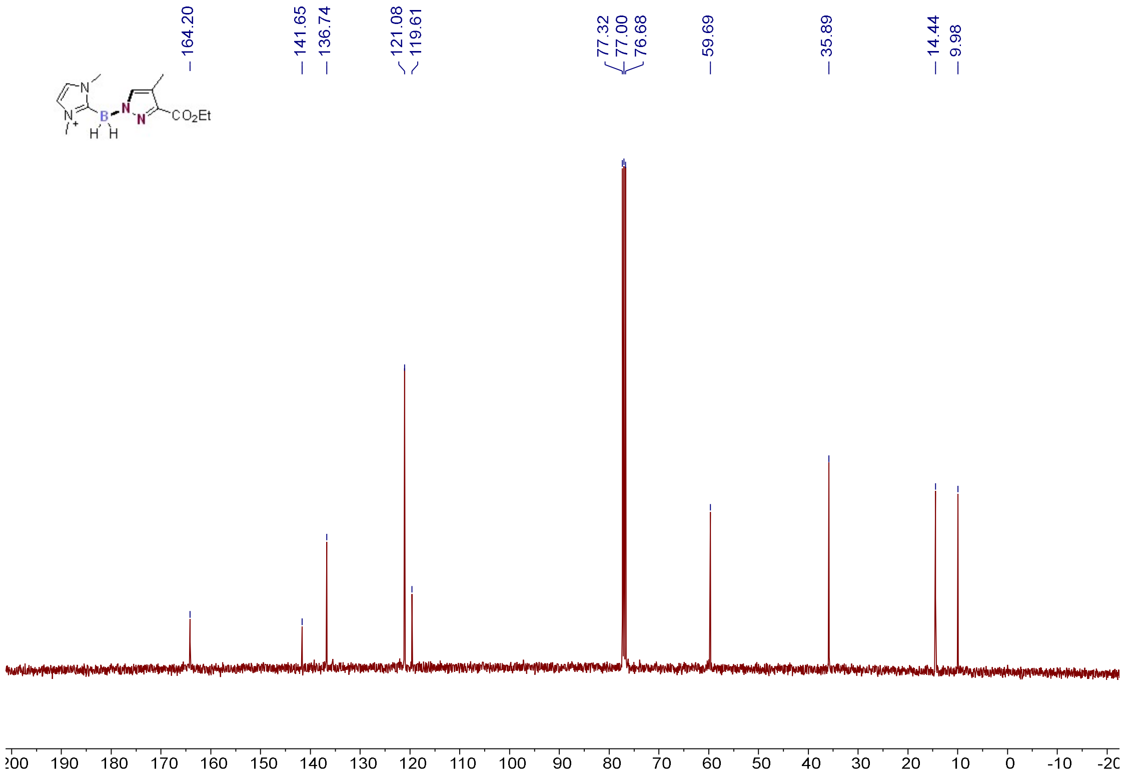

^{11}B NMR (128.4 MHz) Spectrum of 15 in $CDCl_3$

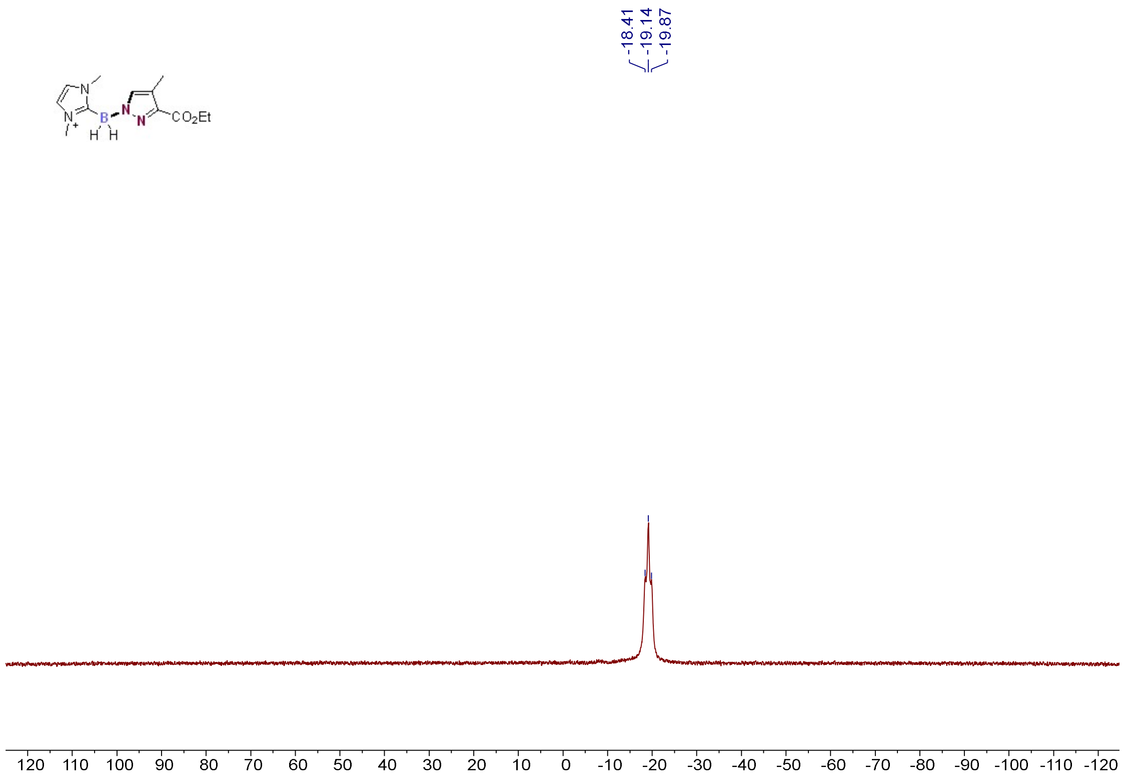

^{1}H NMR (400 MHz) Spectrum of 16 in $CDCl_3$

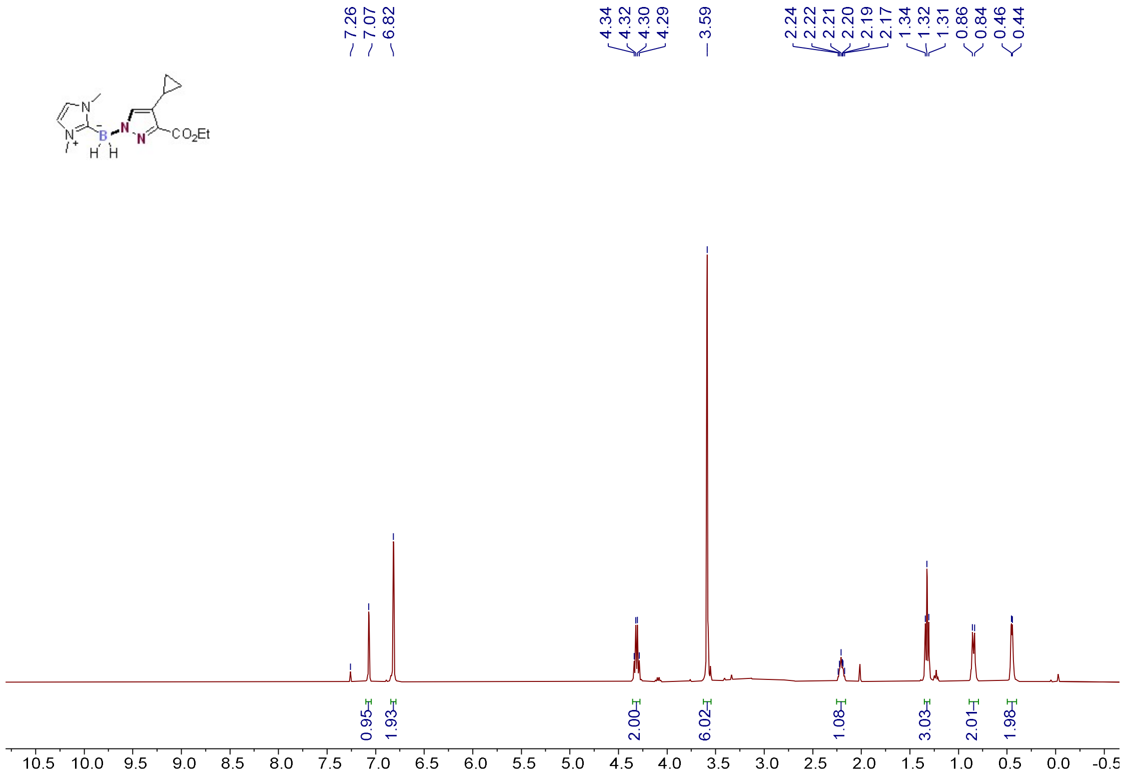

^{13}C NMR (100 MHz) Spectrum of 16 in $CDCl_3$

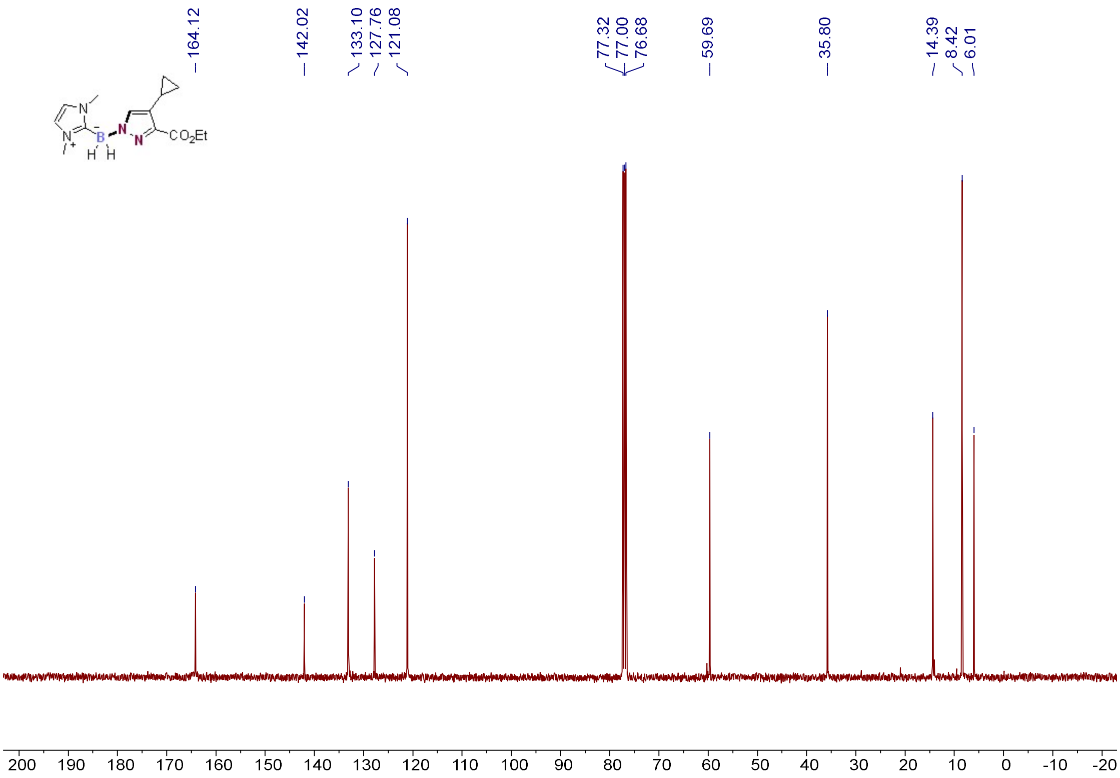

^{11}B NMR (128.4 MHz) Spectrum of 16 in $CDCl_3$

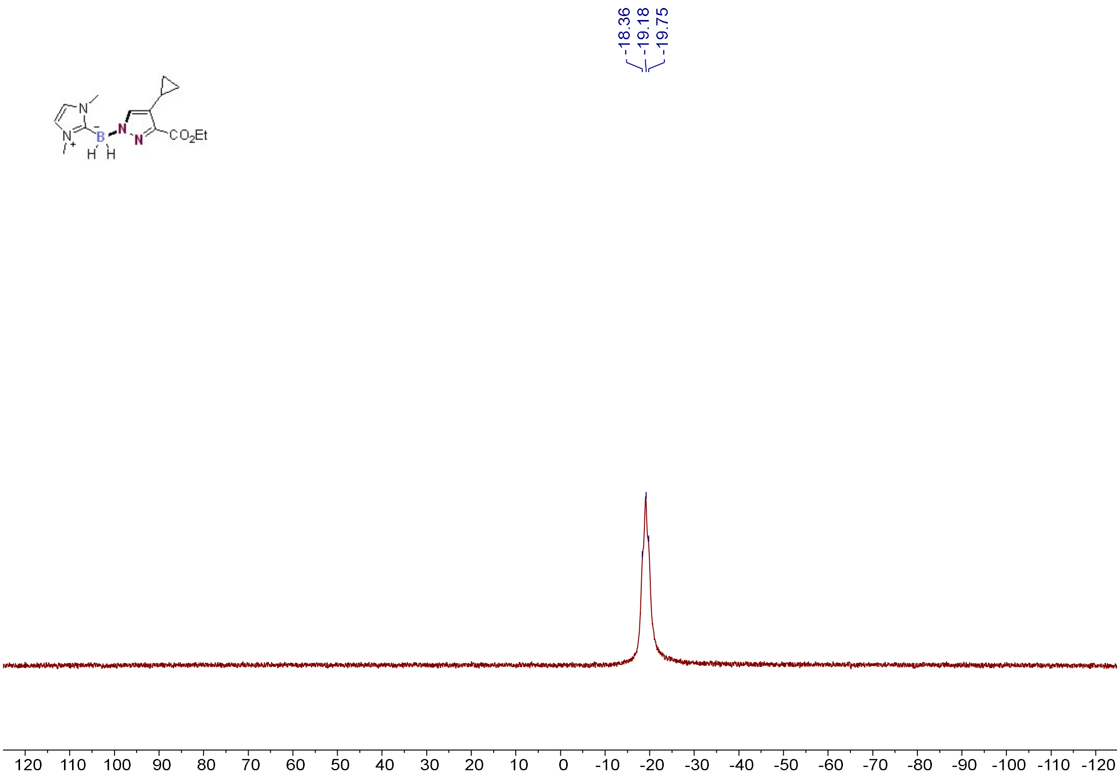

^{1}H NMR (400 MHz) Spectrum of 17 in $CDCl_3$

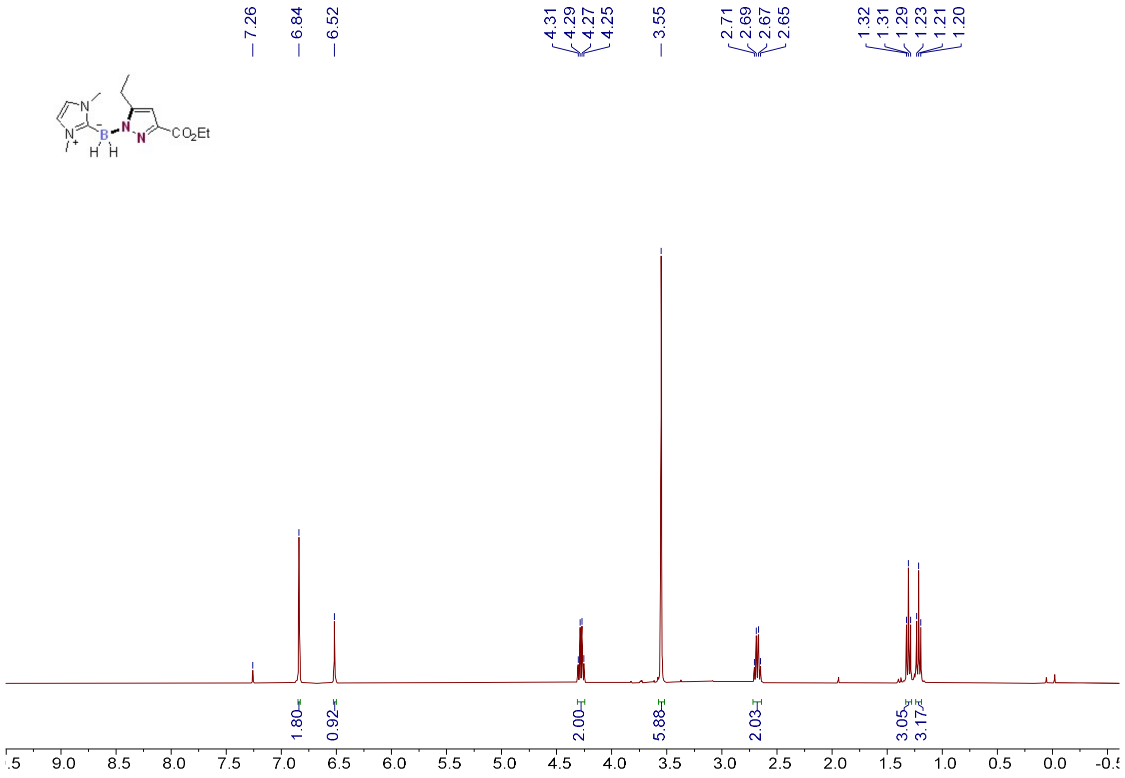

^{13}C NMR (100 MHz) Spectrum of 17 in $CDCl_3$

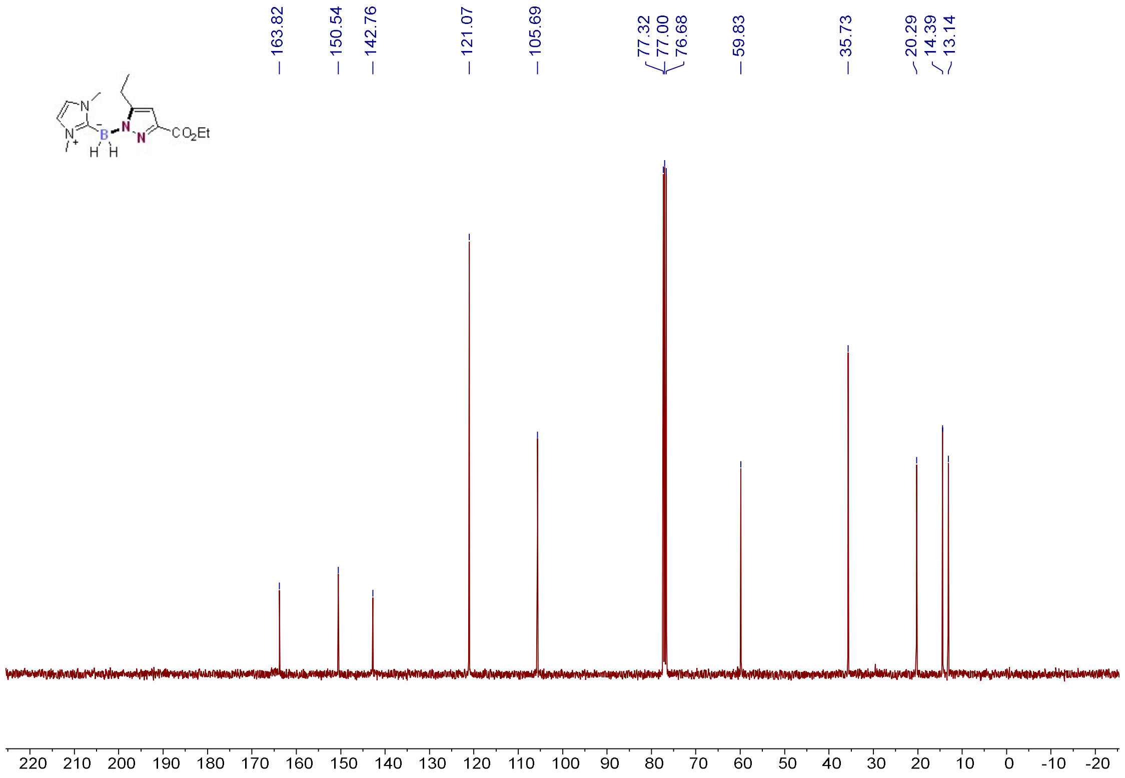

^{11}B NMR (128.4 MHz) Spectrum of 17 in $CDCl_3$

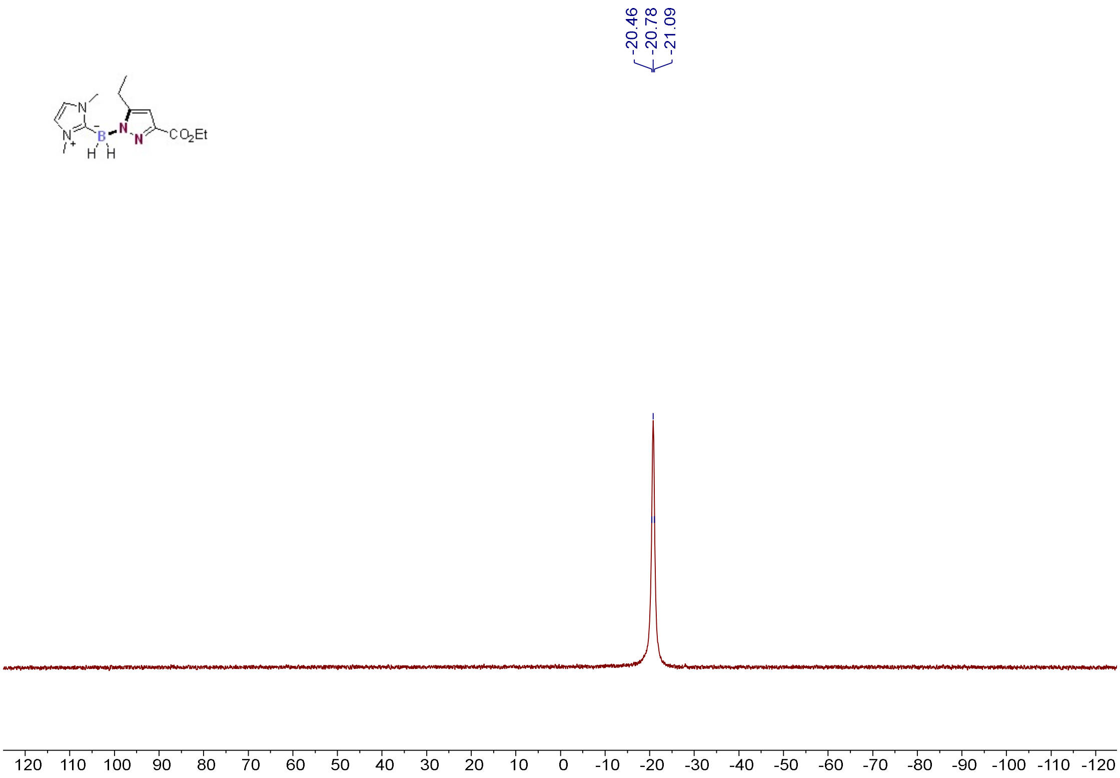

^{1}H NMR (400 MHz) Spectrum of 18 in $CDCl_3$

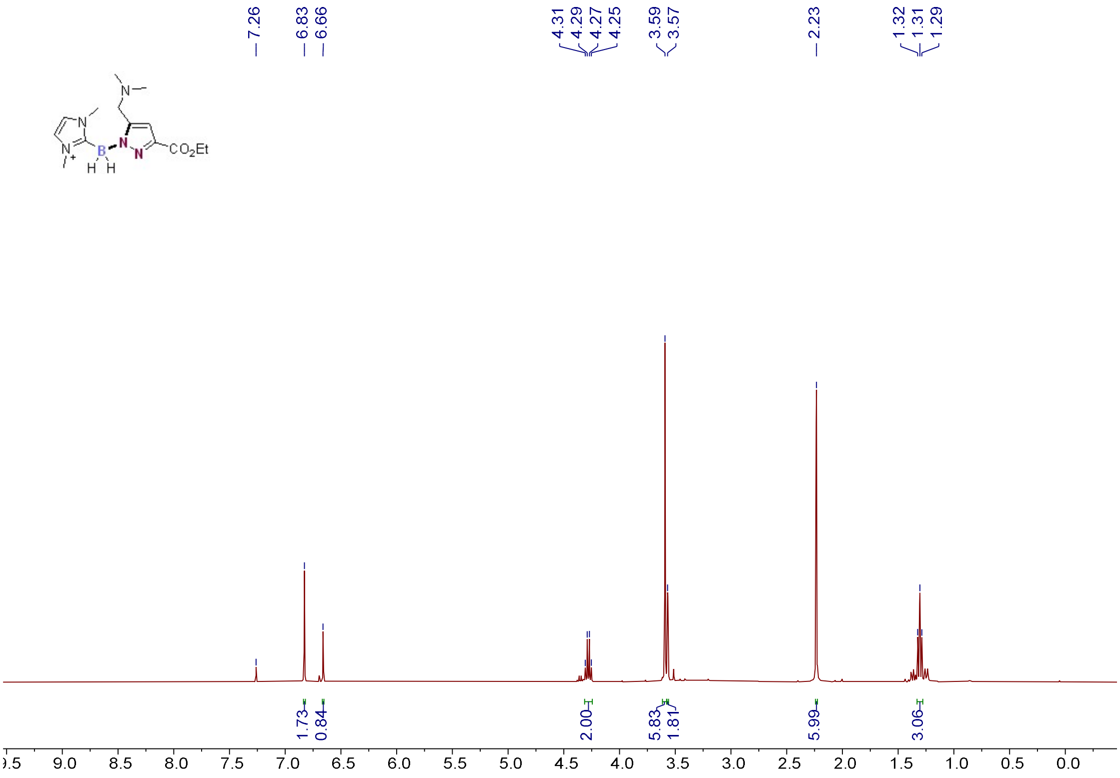

^{13}C NMR (100 MHz) Spectrum of 18 in $CDCl_3$

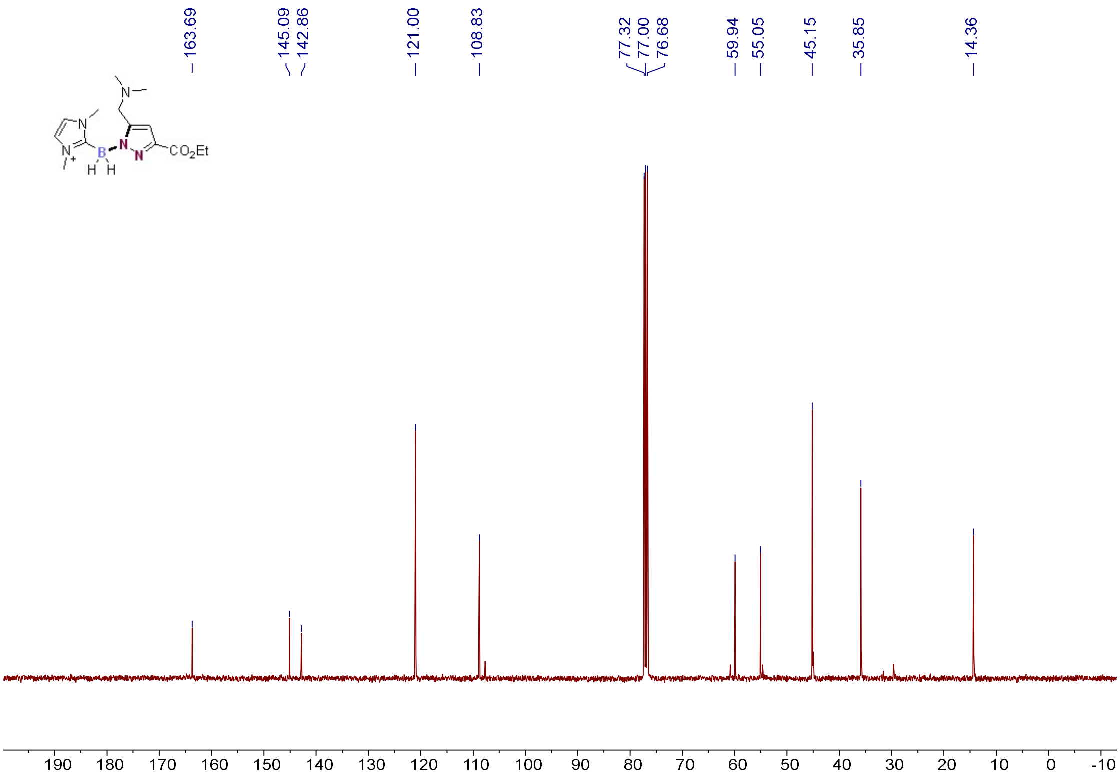

^{11}B NMR (128.4 MHz) Spectrum of 18 in $CDCl_3$

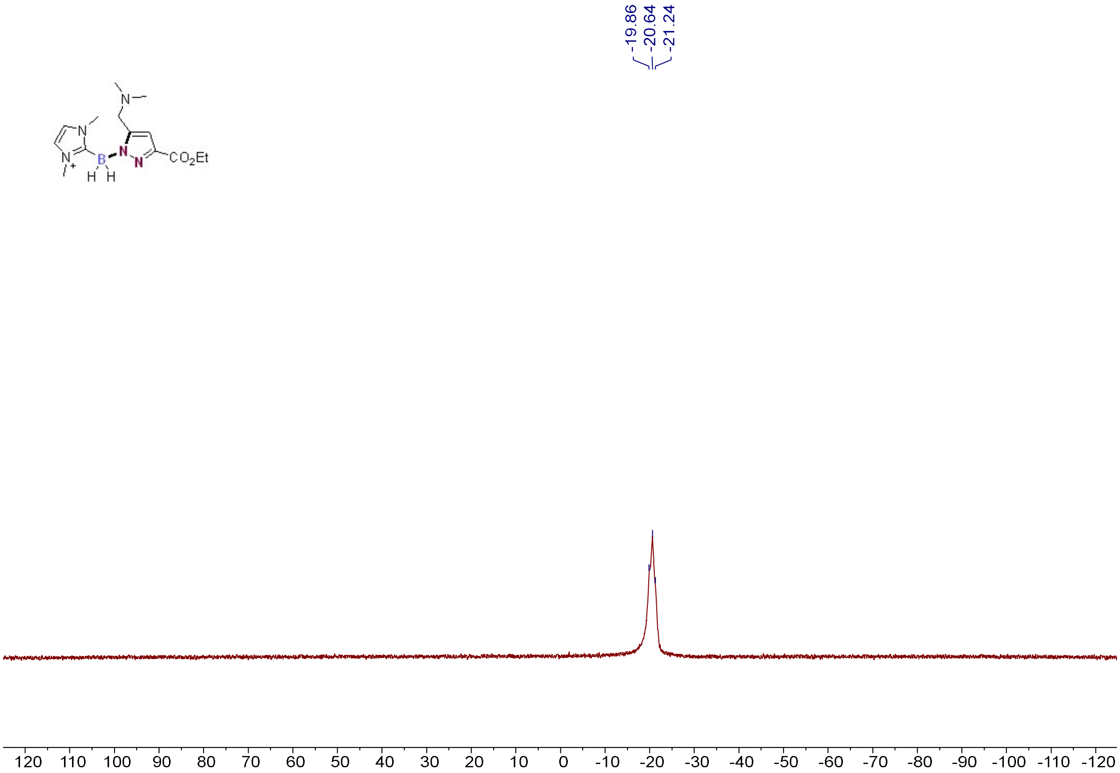

^{1}H NMR (400 MHz) Spectrum of 19 in $CDCl_3$

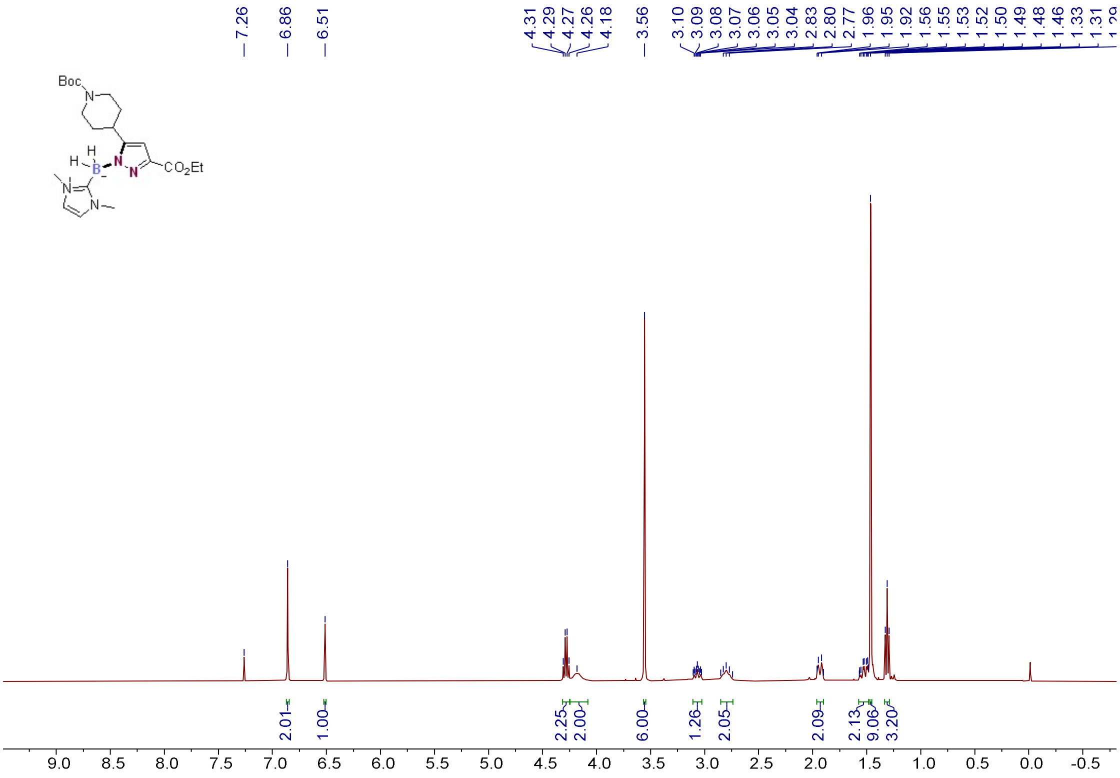

^{13}C NMR (100 MHz) Spectrum of 19 in $CDCl_3$

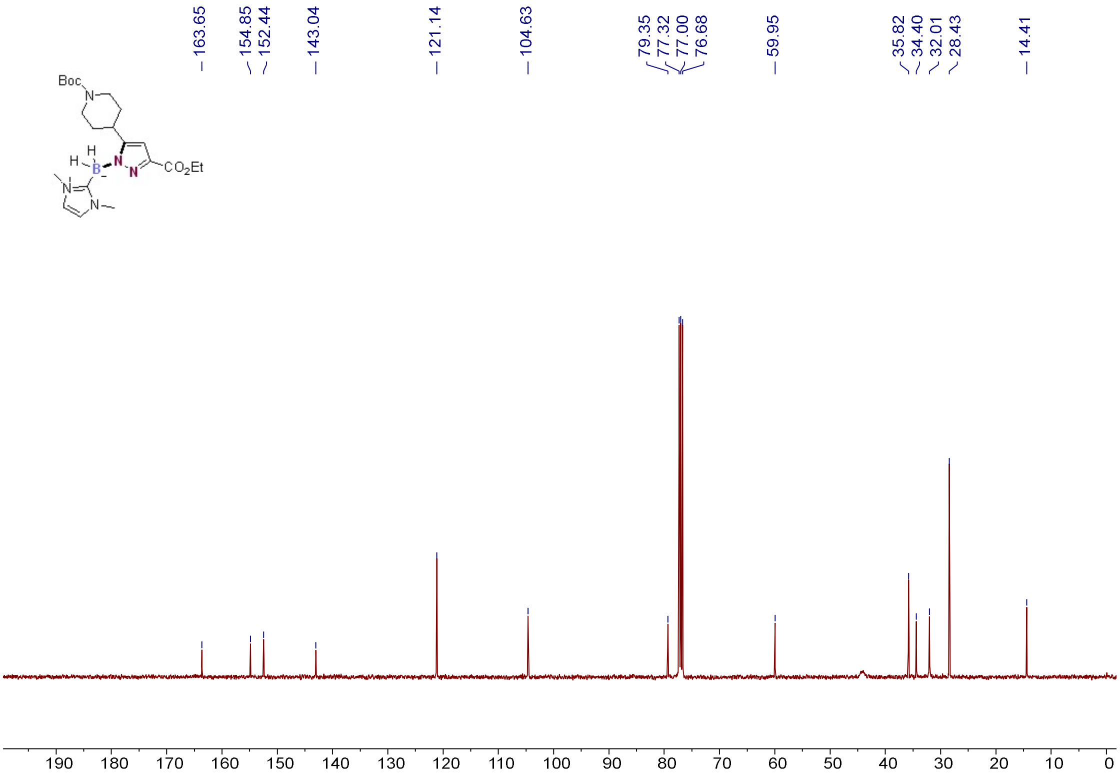

^{11}B NMR (128.4 MHz) Spectrum of 19 in $CDCl_3$

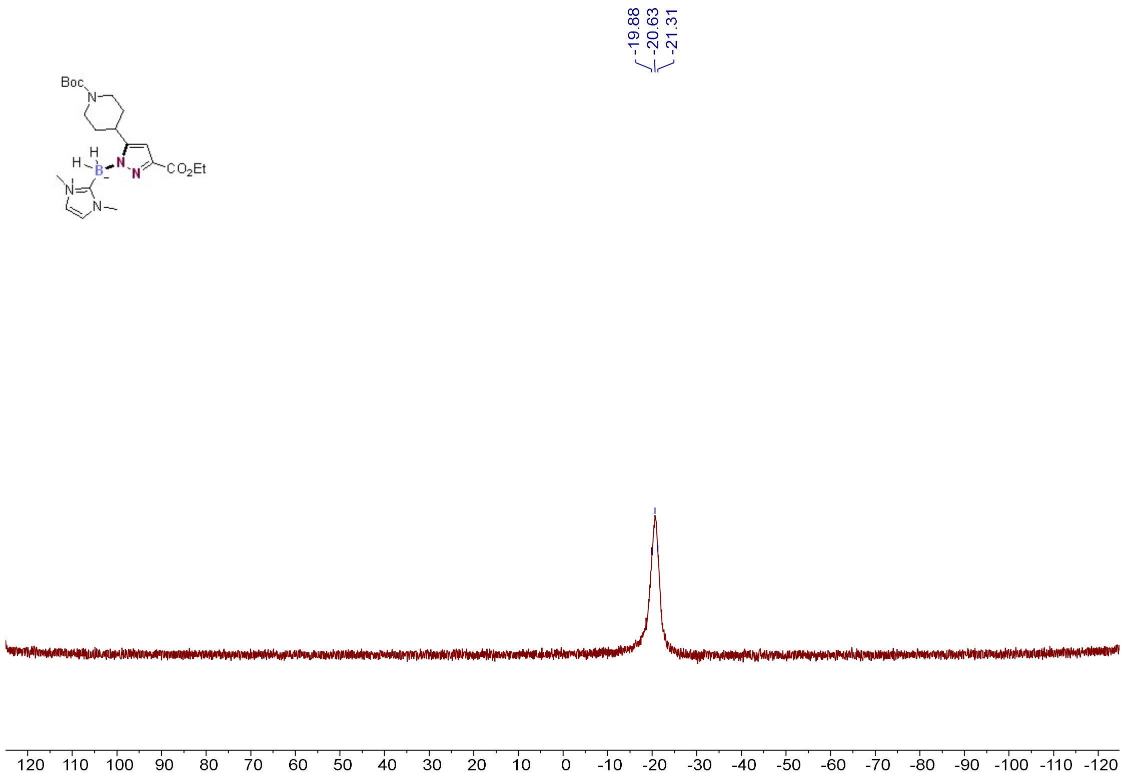

^{1}H NMR (400 MHz) Spectrum of 20 in $CDCl_3$

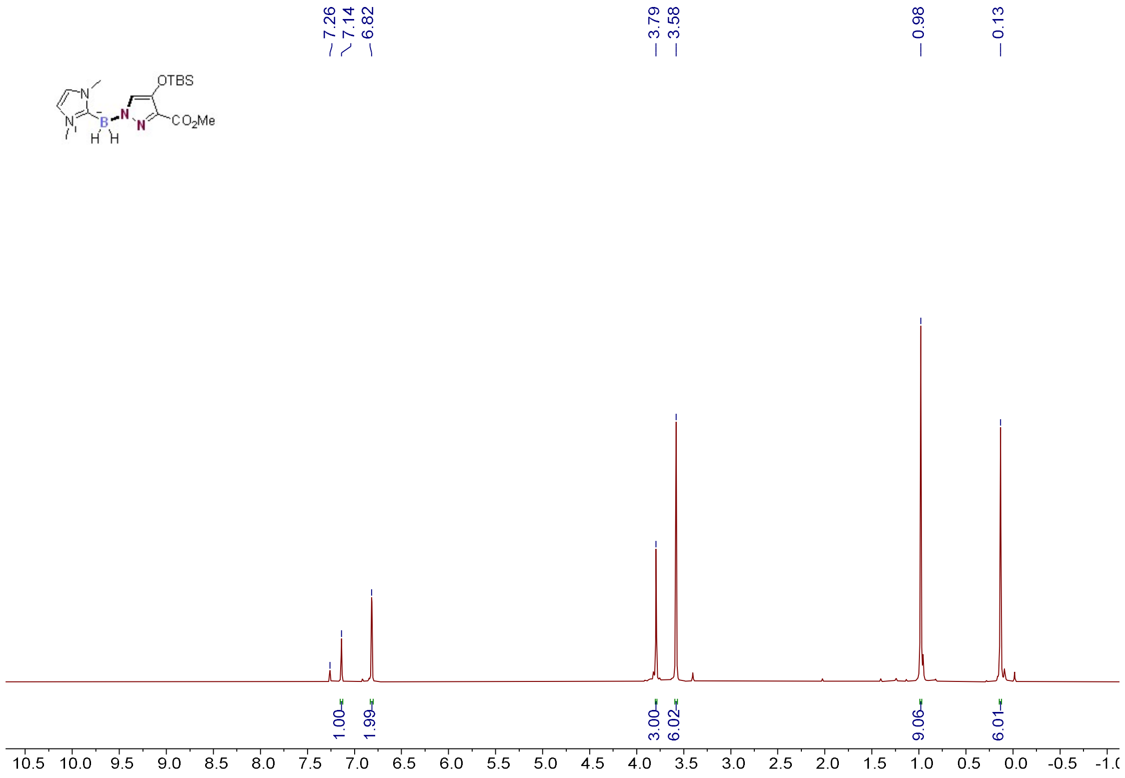

^{13}C NMR (100 MHz) Spectrum of 20 in $CDCl_3$

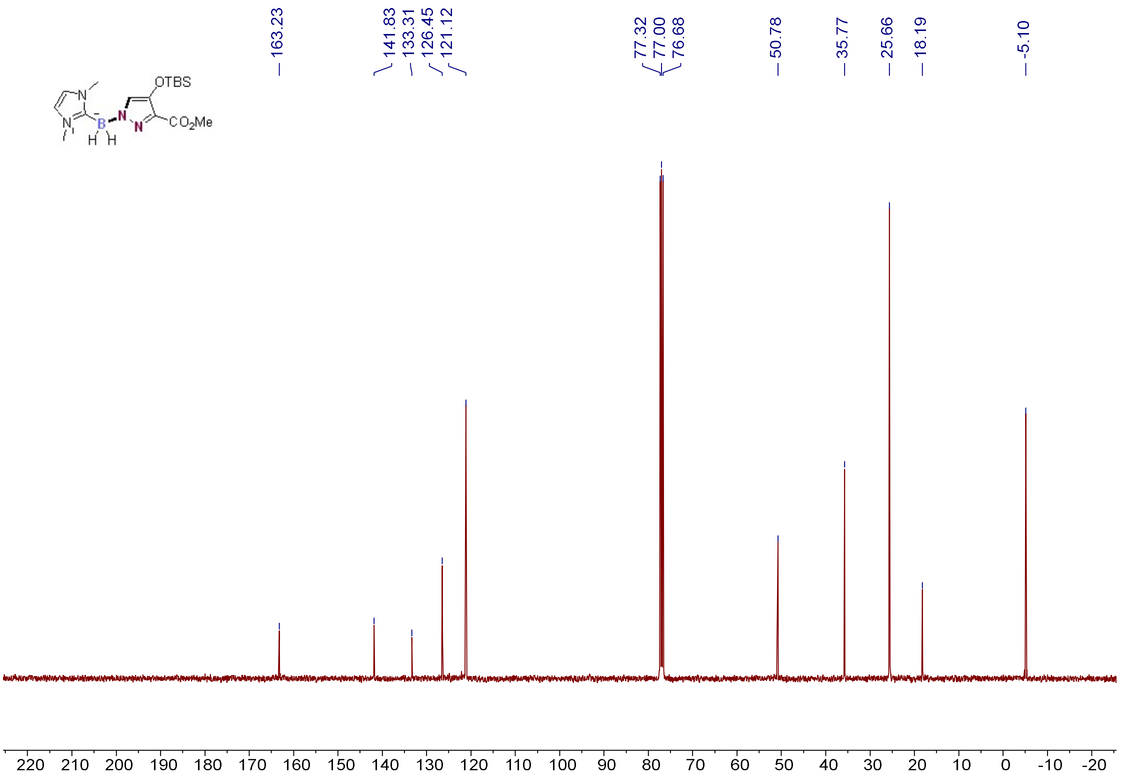

^{11}B NMR (128.4 MHz) Spectrum of 20 in $CDCl_3$

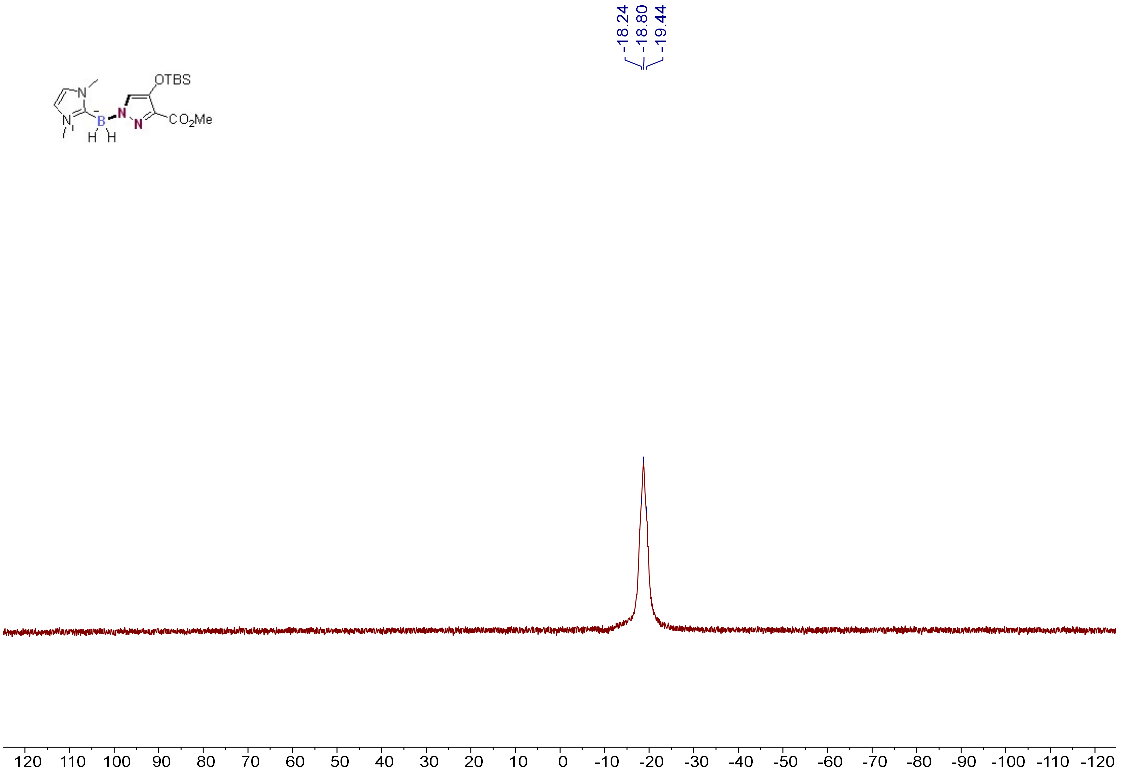

^{1}H NMR (400 MHz) Spectrum of 21 in CDCl$_3$

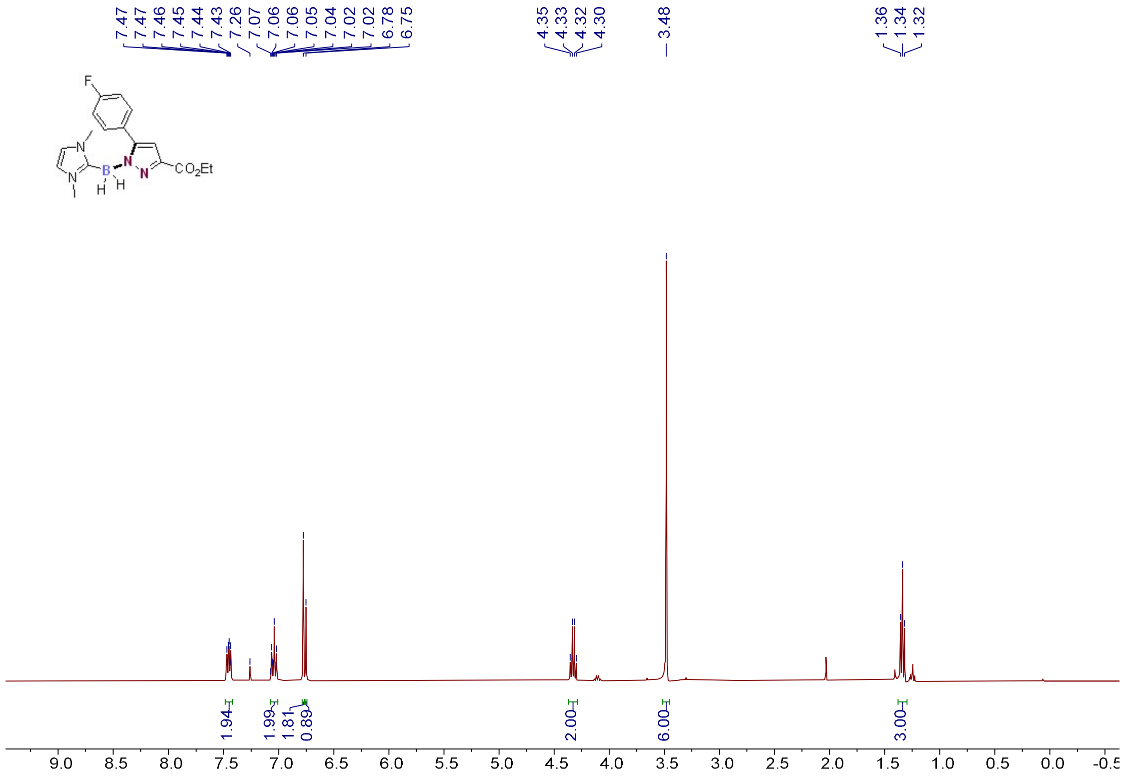

^{13}C NMR (100 MHz) Spectrum of 21 in CDCl$_3$

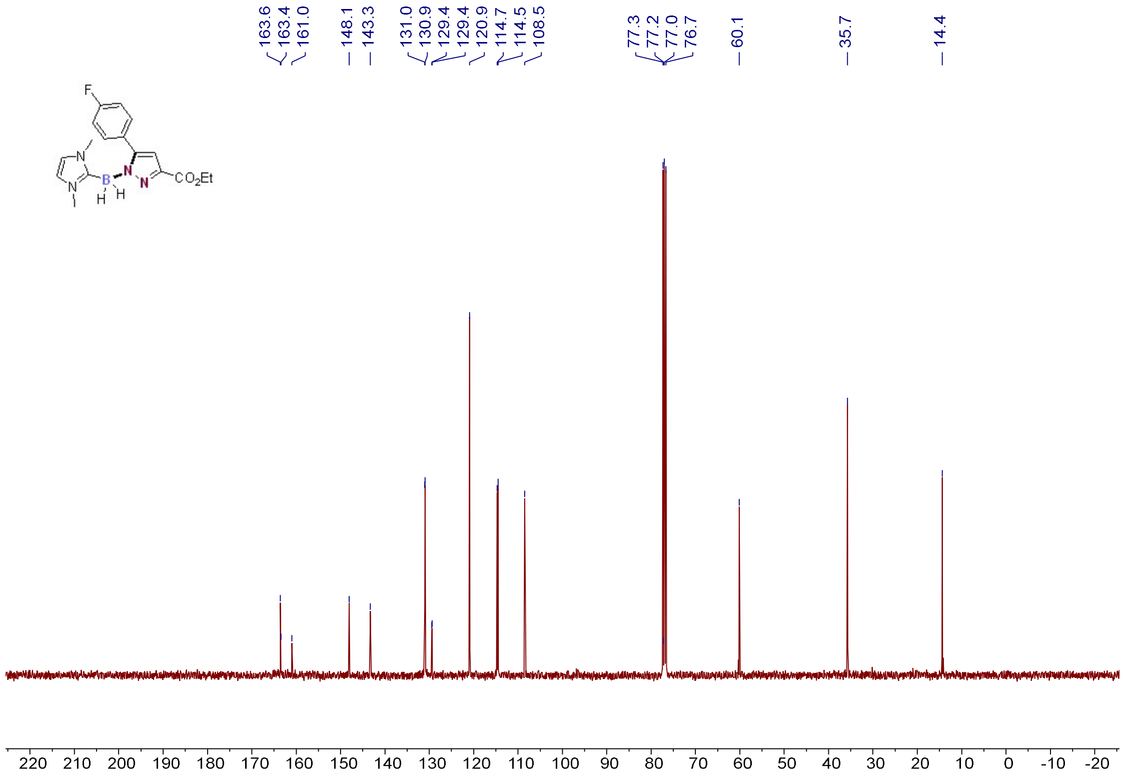

^{11}B NMR (128.4 MHz) Spectrum of 21 in $CDCl_3$

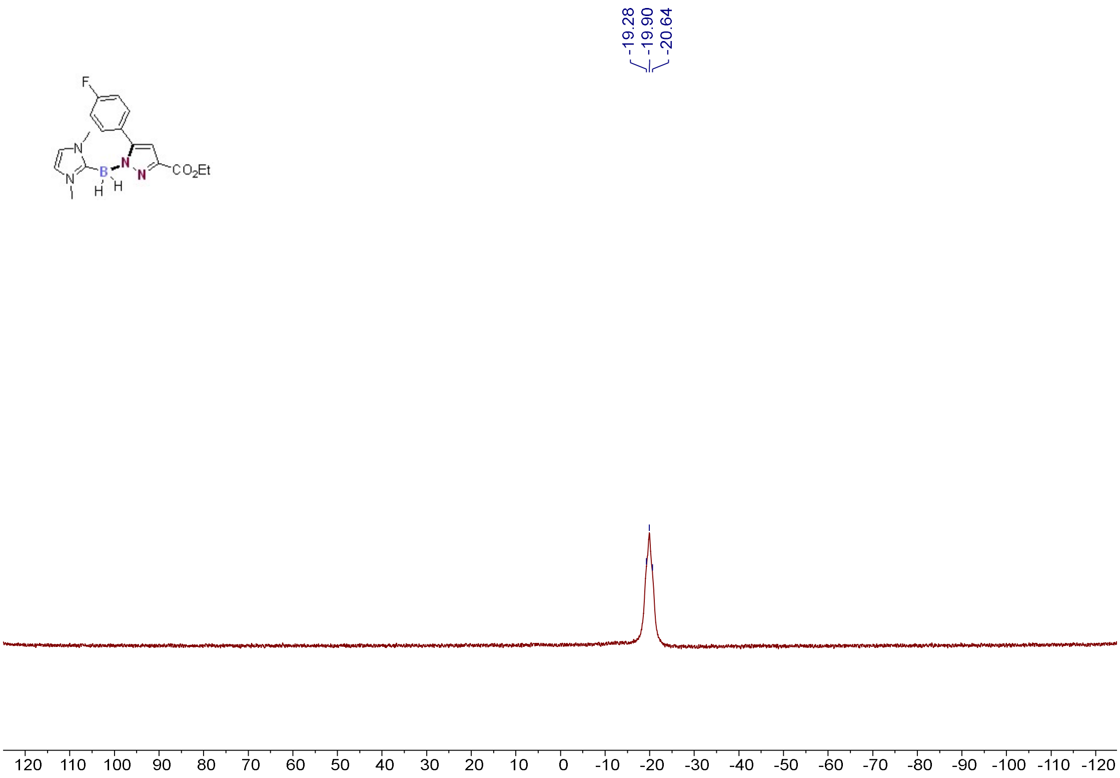

^{1}H NMR (400 MHz) Spectrum of 22 in $CDCl_3$

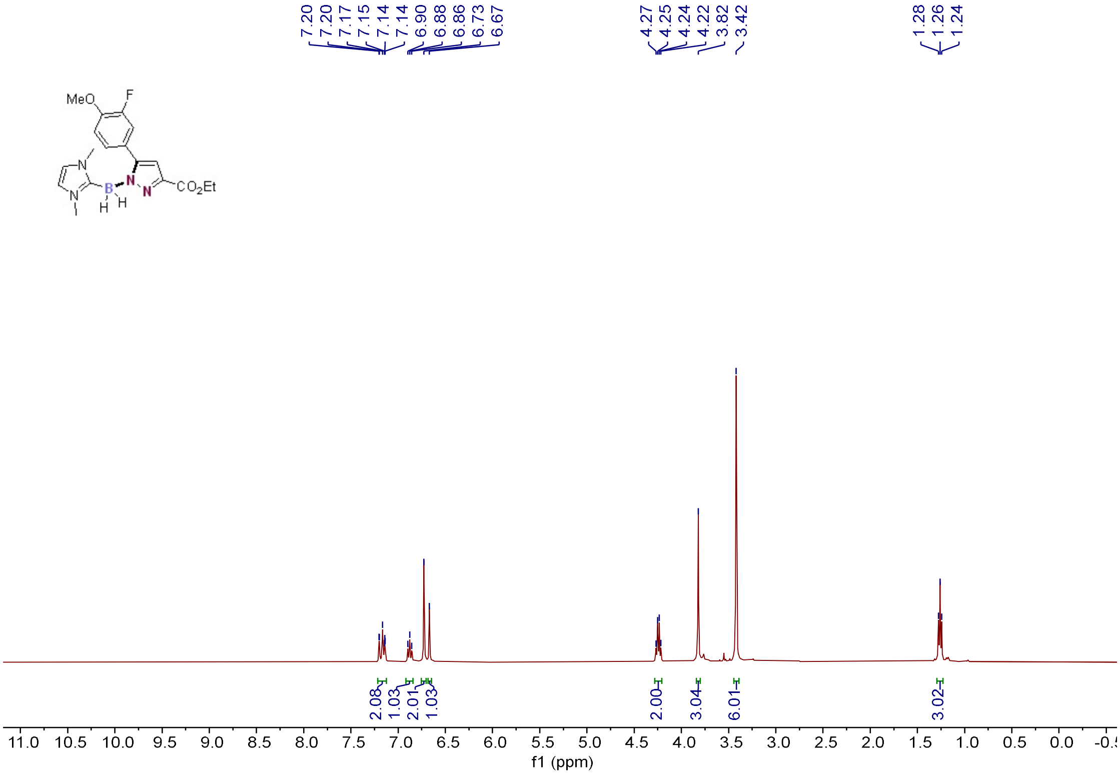

^{13}C NMR (100 MHz) Spectrum of 22 in $CDCl_3$

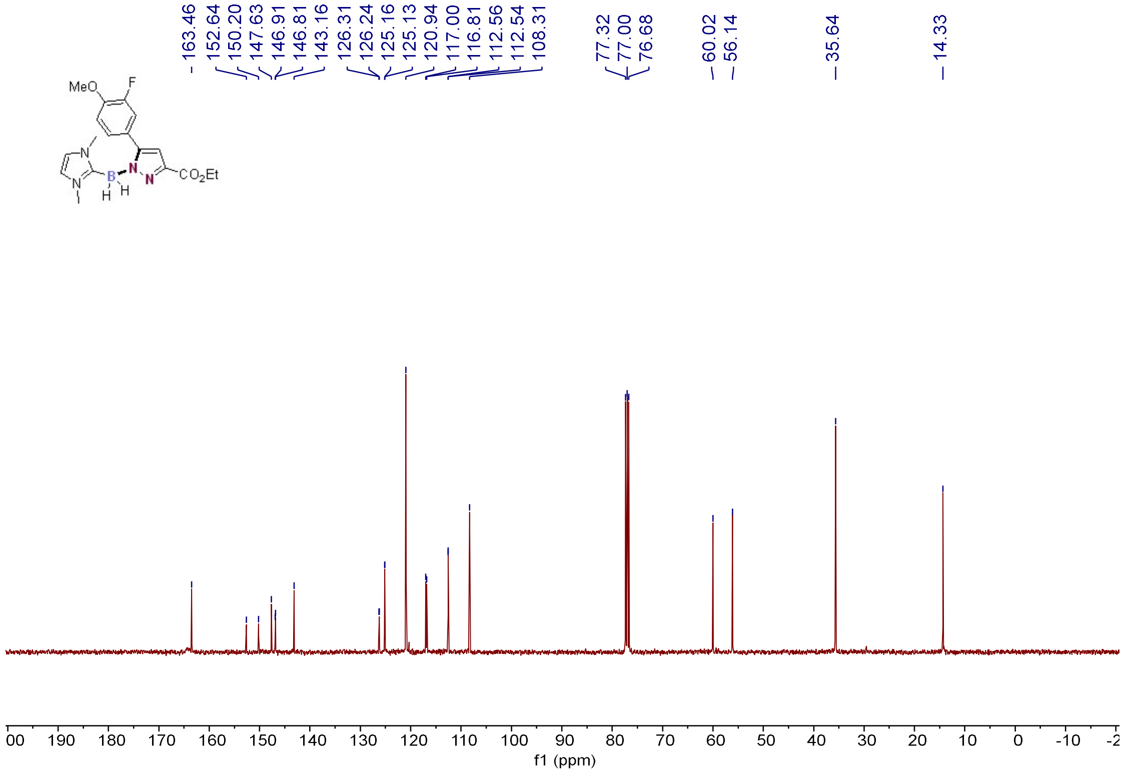

^{11}B NMR (128.4 MHz) Spectrum of 22 in $CDCl_3$

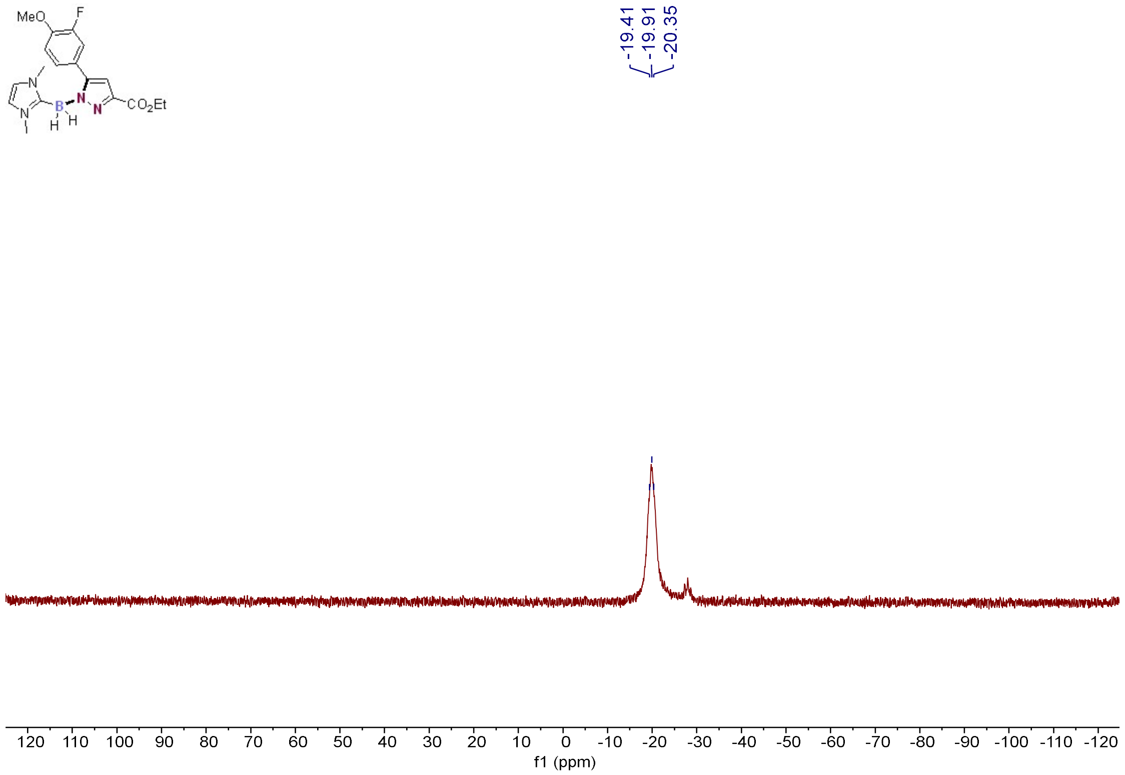

^{1}H NMR (400 MHz) Spectrum of 23 in $CDCl_3$

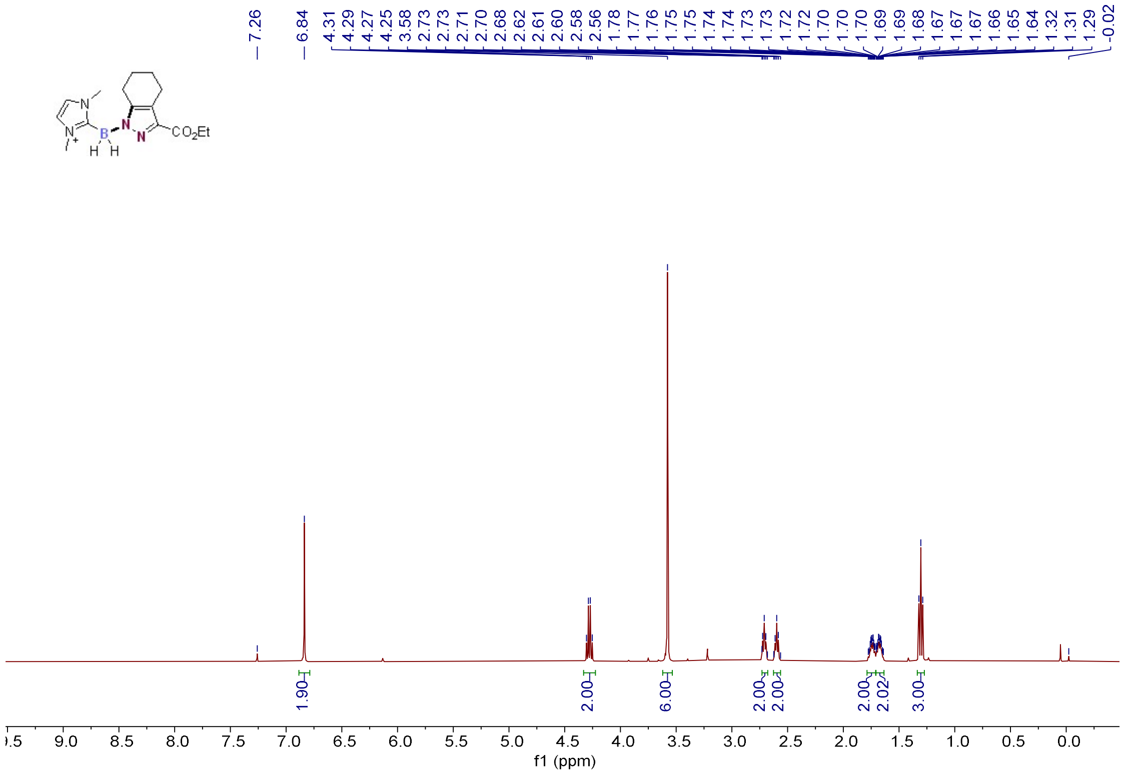

^{13}C NMR (100 MHz) Spectrum of 23 in $CDCl_3$

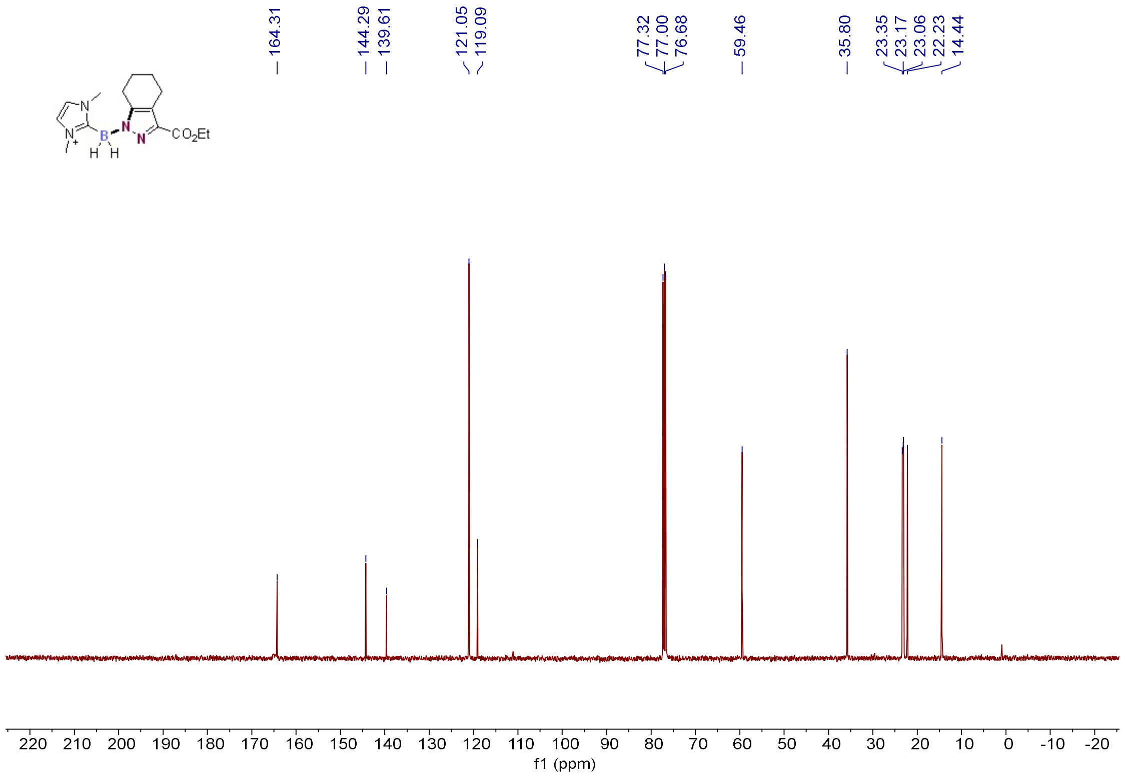

^{11}B NMR (128.4 MHz) Spectrum of 23 in $CDCl_3$

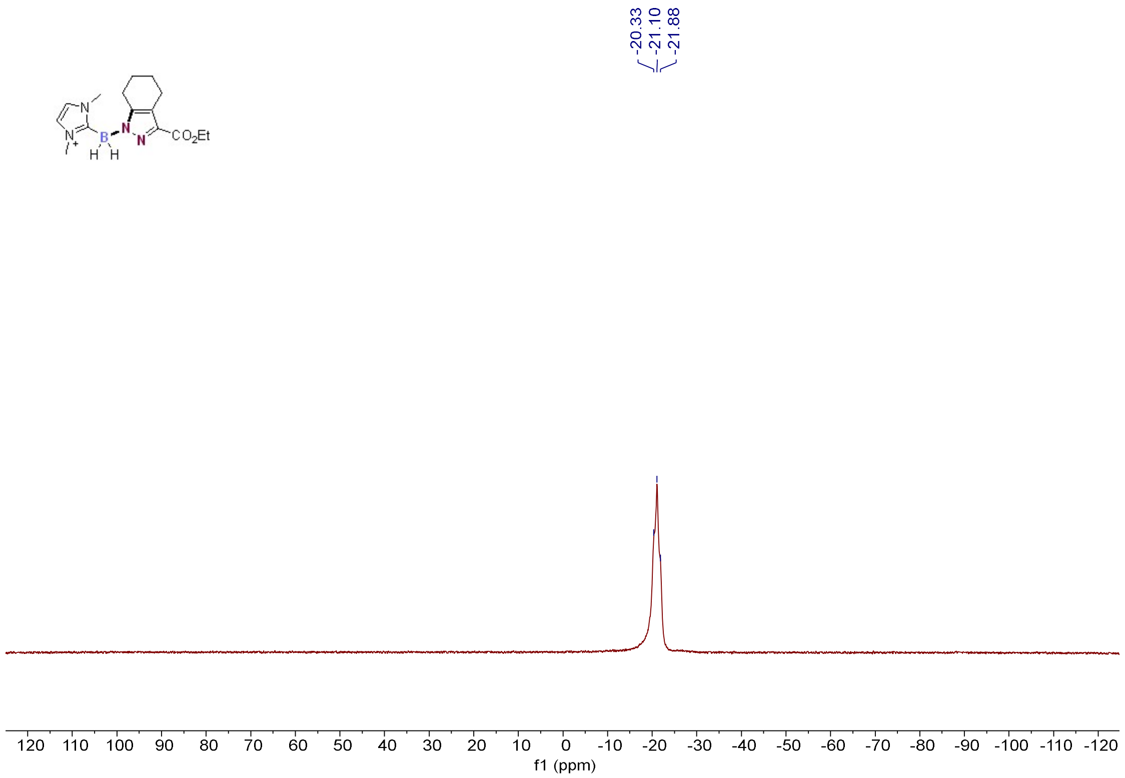

^{1}H NMR (400 MHz) Spectrum of 24 in $CDCl_3$

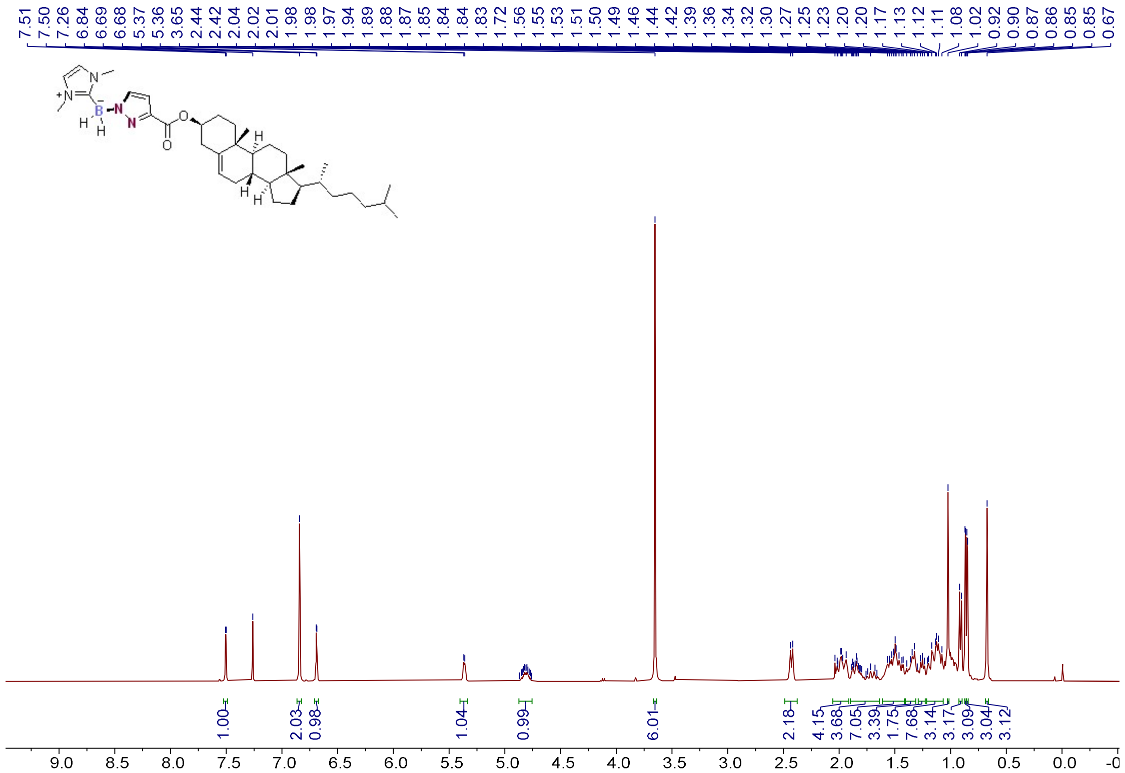

^{13}C NMR (100 MHz) Spectrum of 24 in $CDCl_3$

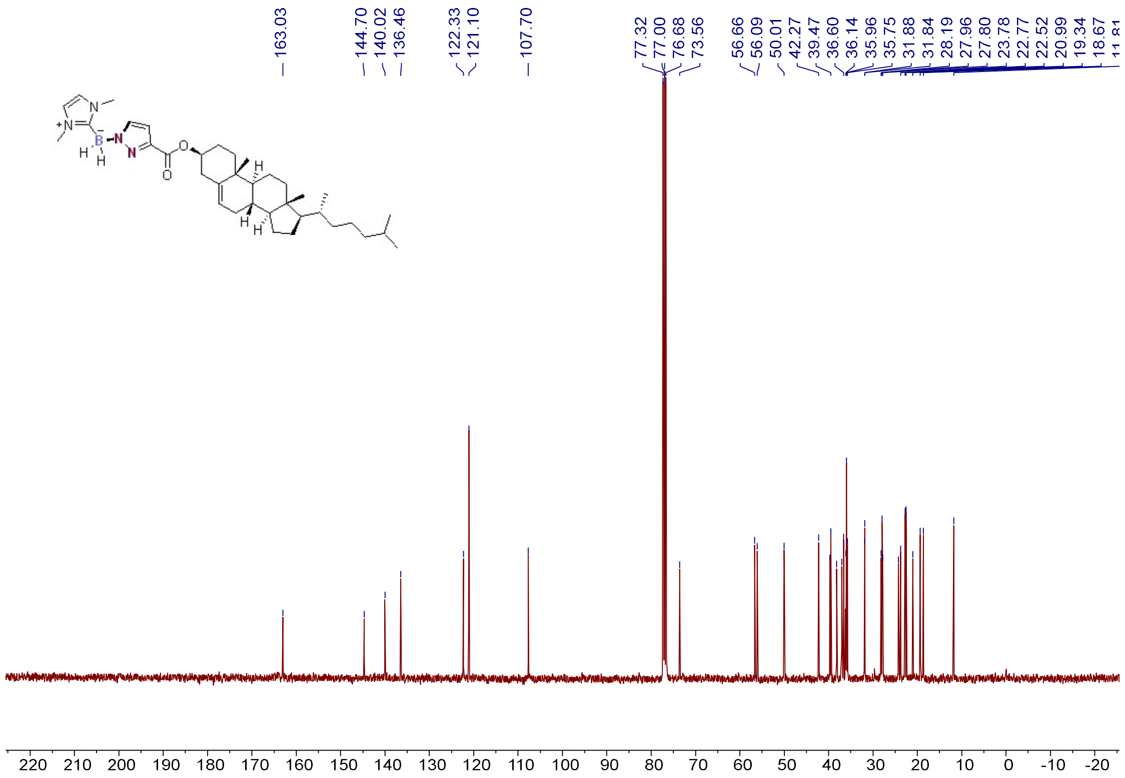

^{11}B NMR (128.4 MHz) Spectrum of 24 in $CDCl_3$

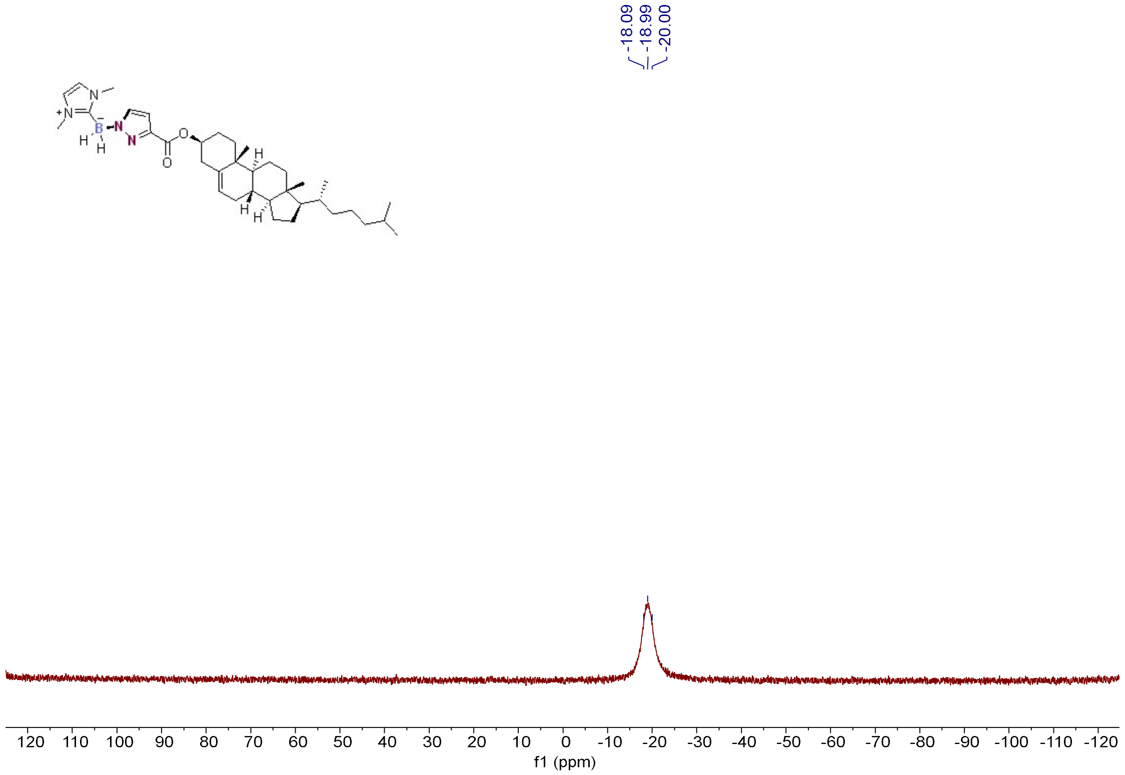

^{1}H NMR (400 MHz) Spectrum of 25 in $CDCl_3$

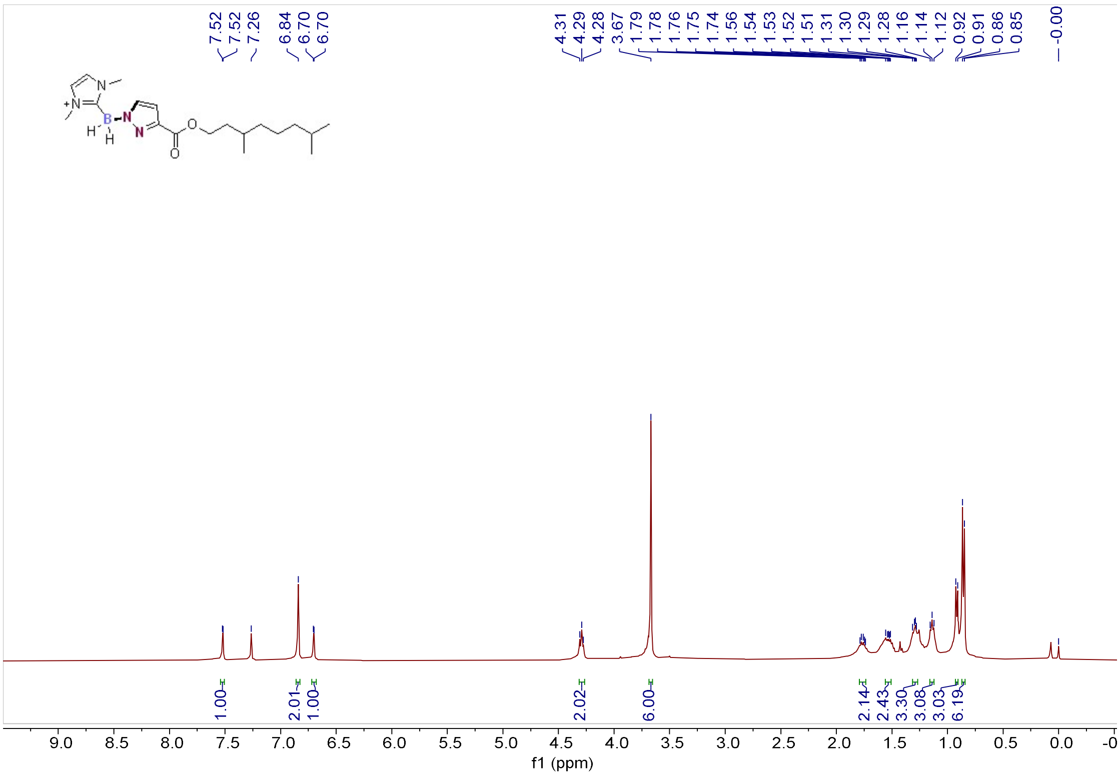

^{13}C NMR (100 MHz) Spectrum of 25 in $CDCl_3$

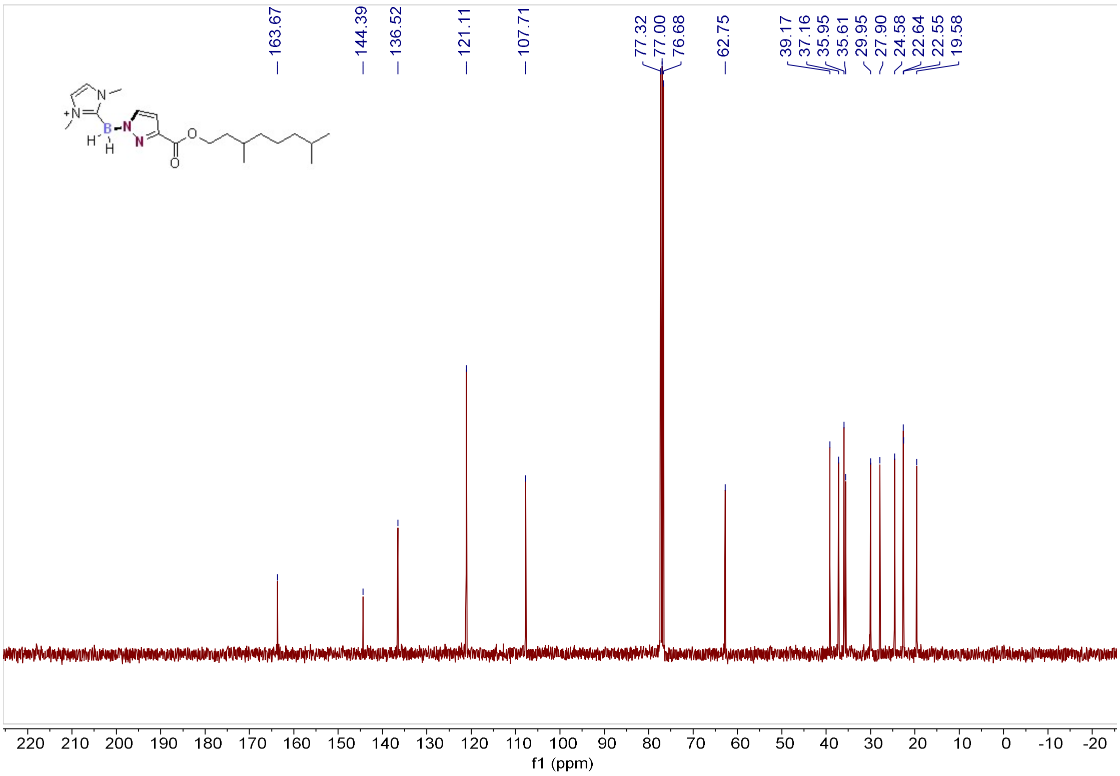

^{11}B NMR (128.4 MHz) Spectrum of 25 in $CDCl_3$

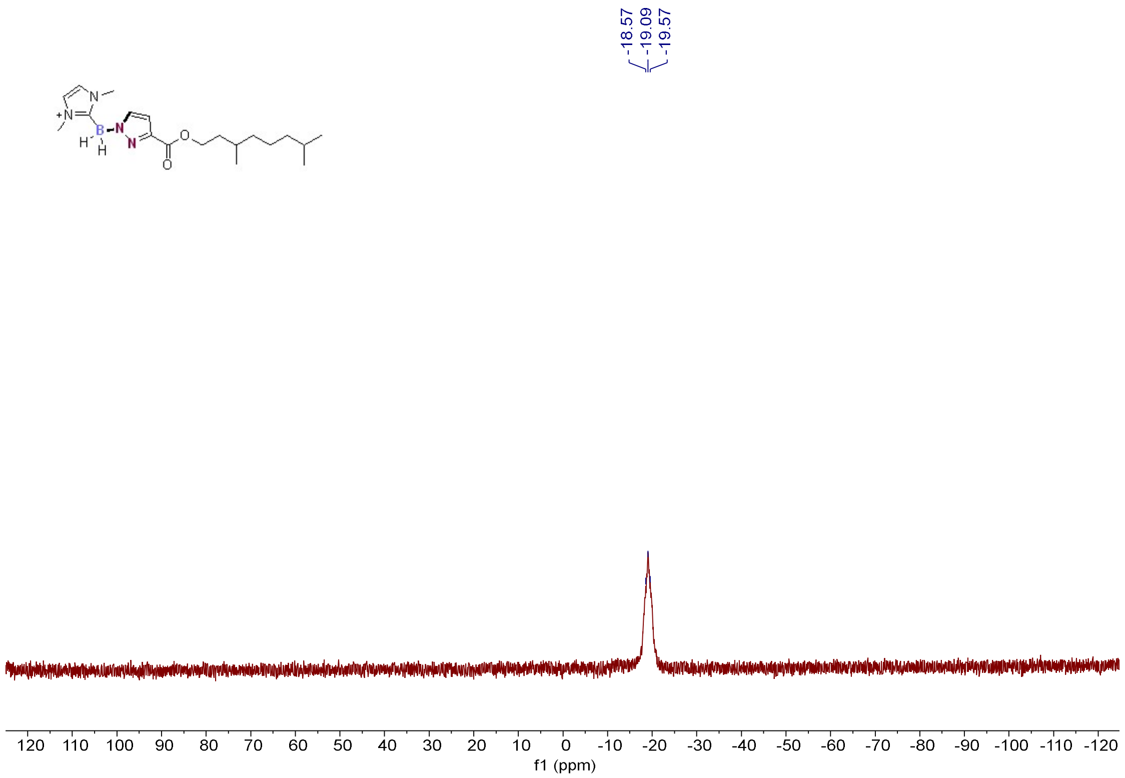

^{1}H NMR (400 MHz) Spectrum of 26 in $CDCl_3$

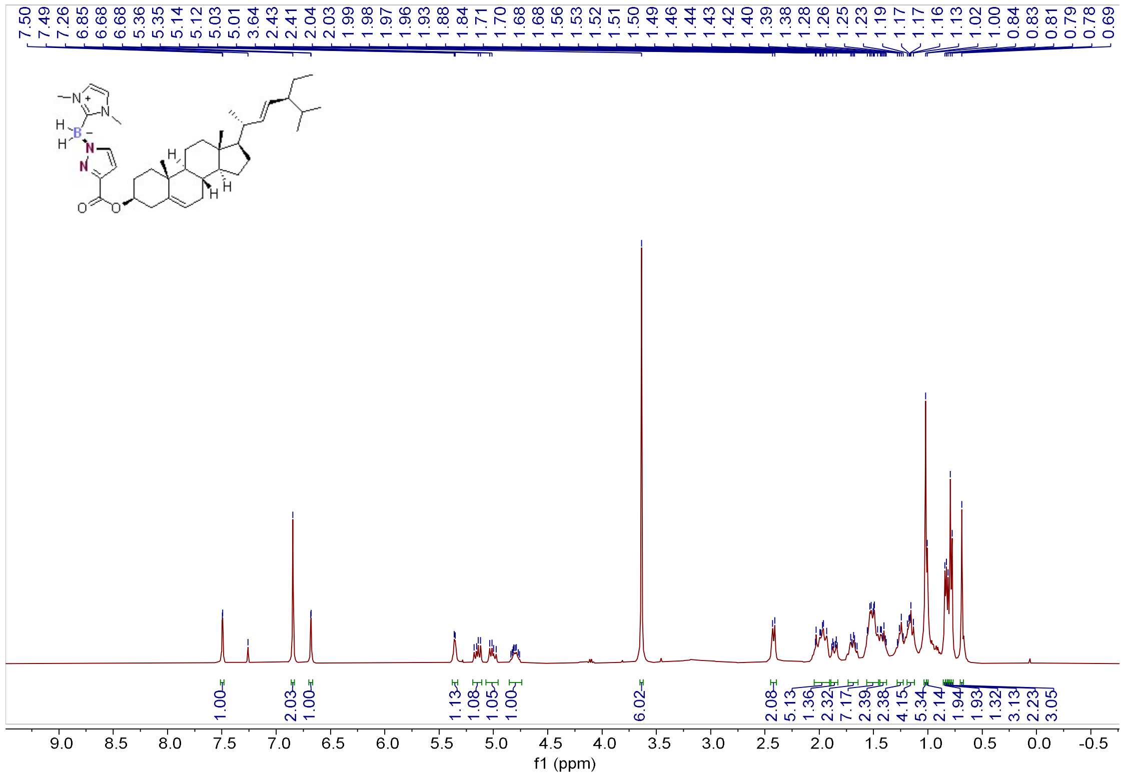

^{13}C NMR (100 MHz) Spectrum of 26 in CDCl$_3$

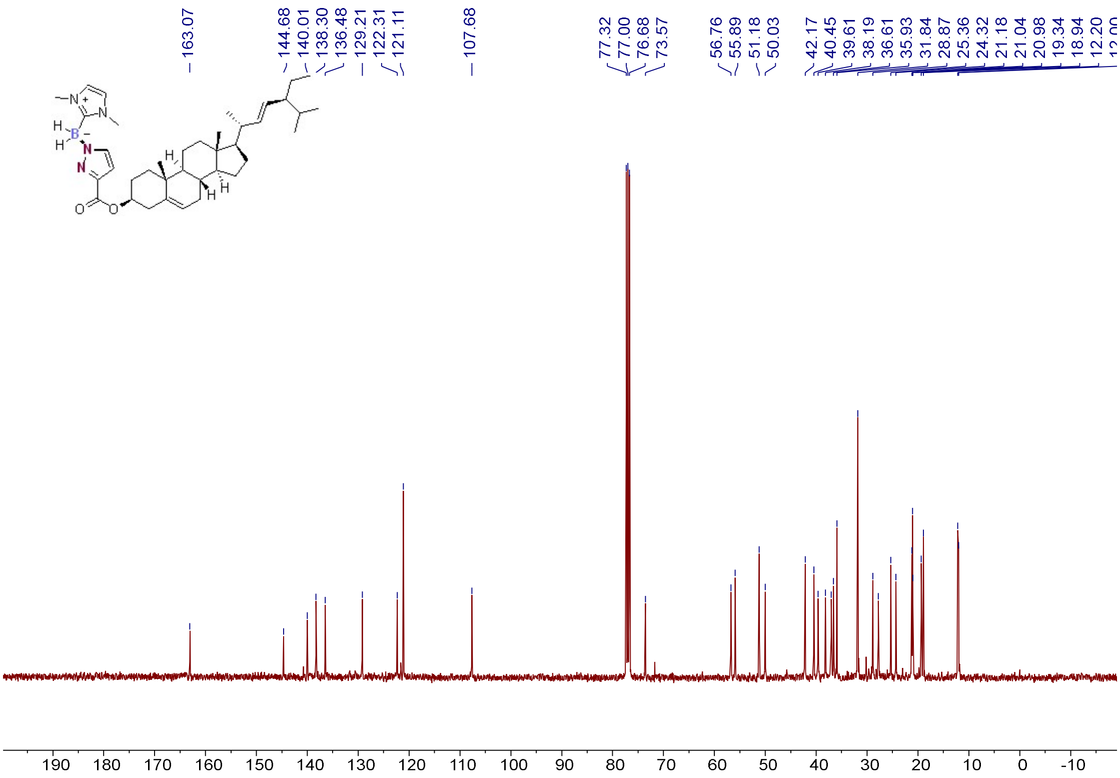

^{11}B NMR (128.4 MHz) Spectrum of 26 in CDCl$_3$

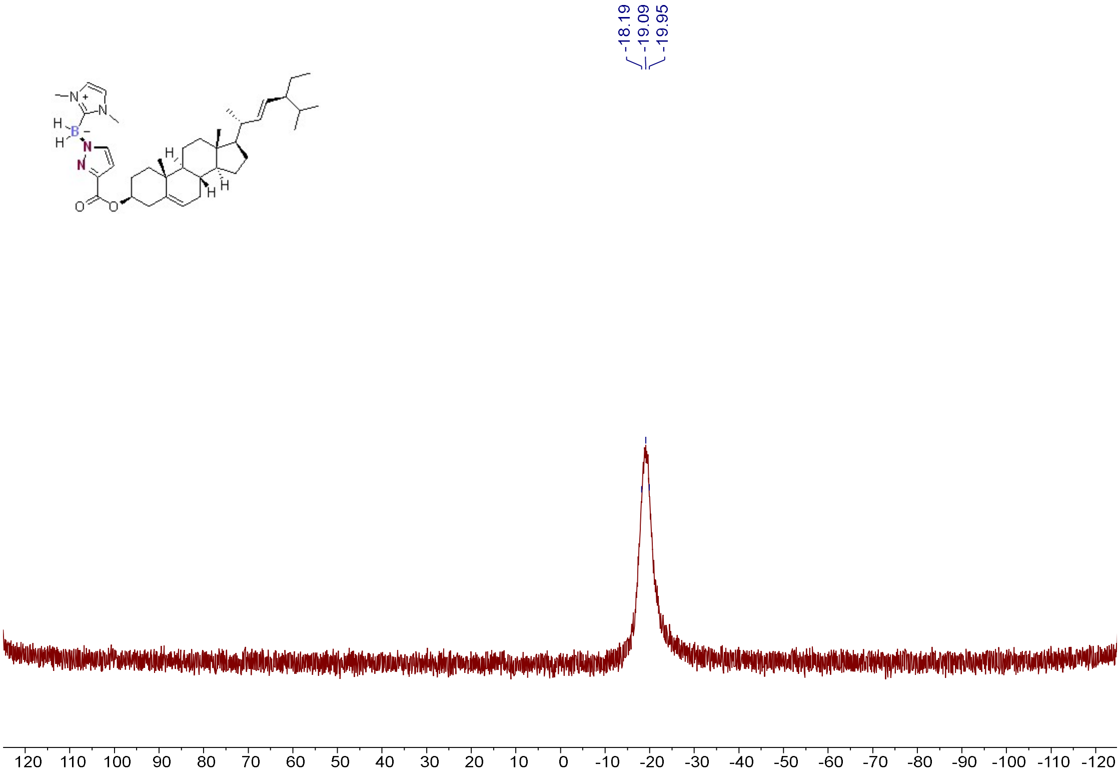

^{1}H NMR (400 MHz) Spectrum of 27 in $CDCl_3$

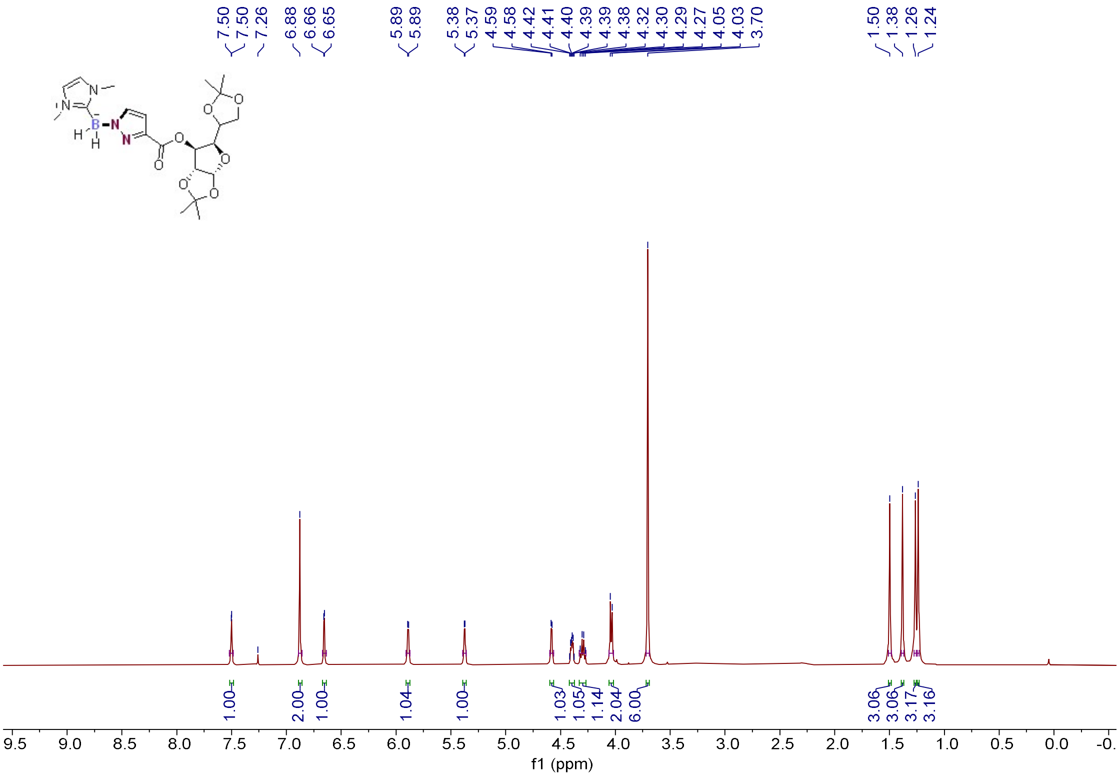

^{13}C NMR (100 MHz) Spectrum of 27 in $CDCl_3$

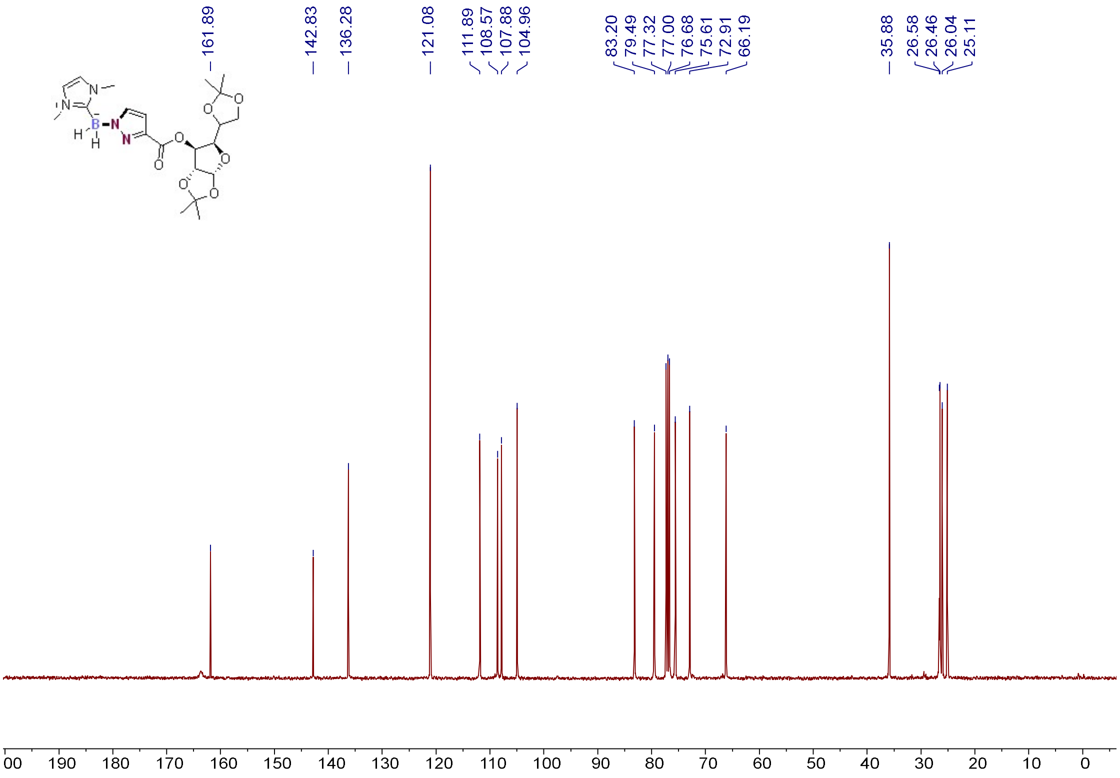

^{11}B NMR (128.4 MHz) Spectrum of 27 in $CDCl_3$

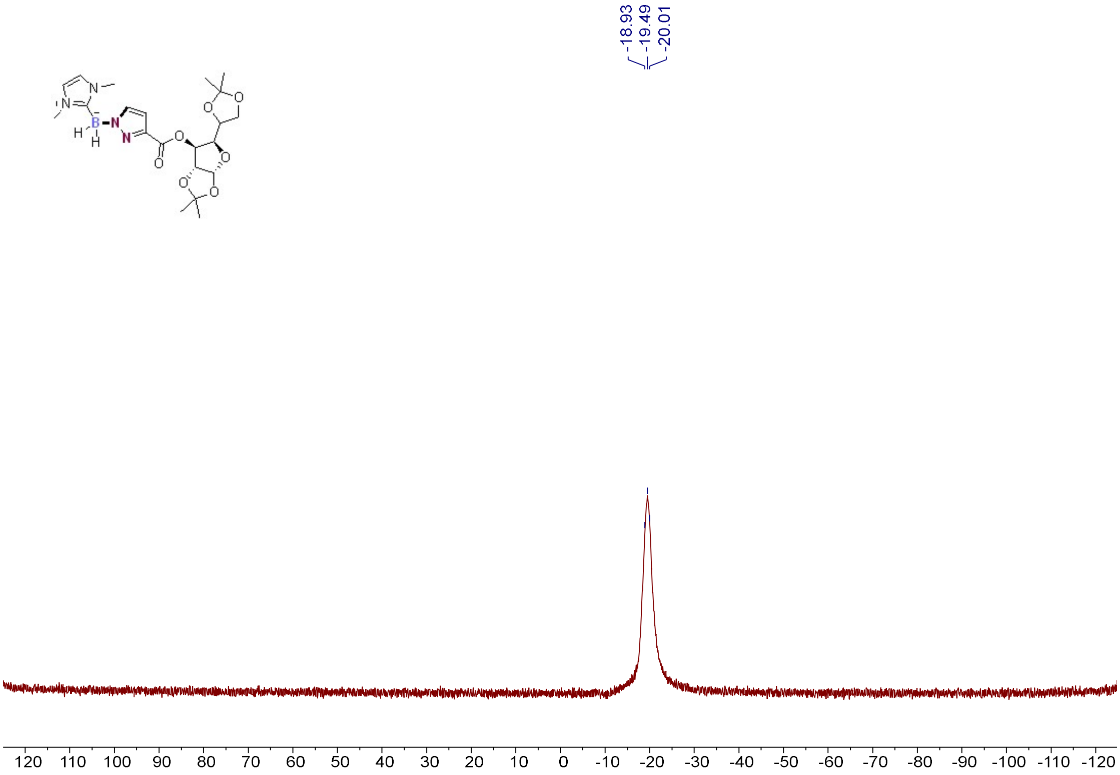

^{1}H NMR (400 MHz) Spectrum of 28 in $CDCl_3$

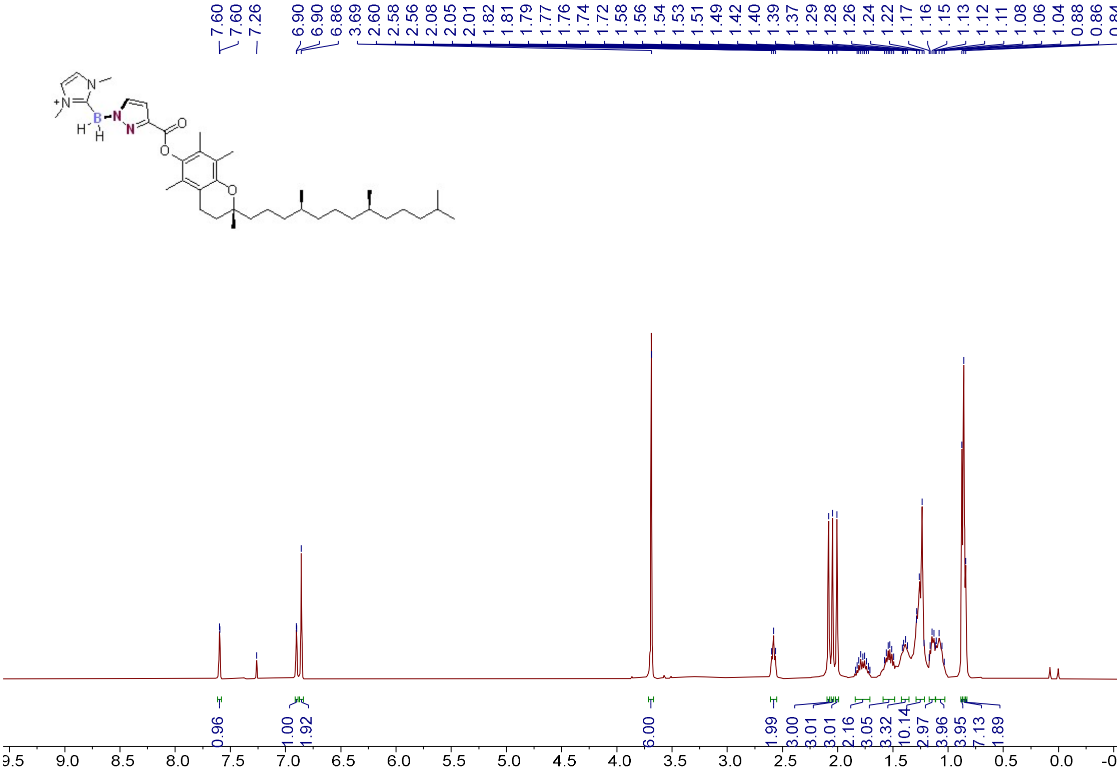

^{13}C NMR (100 MHz) Spectrum of 28 in $CDCl_3$

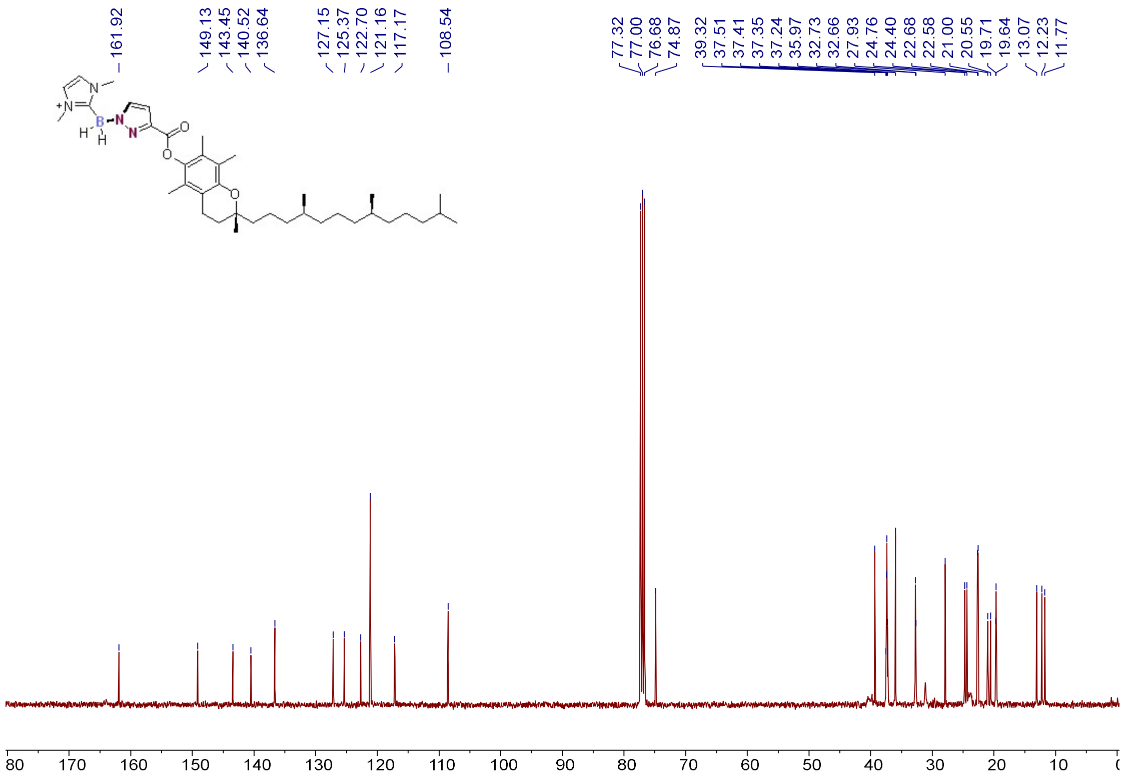

^{11}B NMR (128.4 MHz) Spectrum of 28 in $CDCl_3$

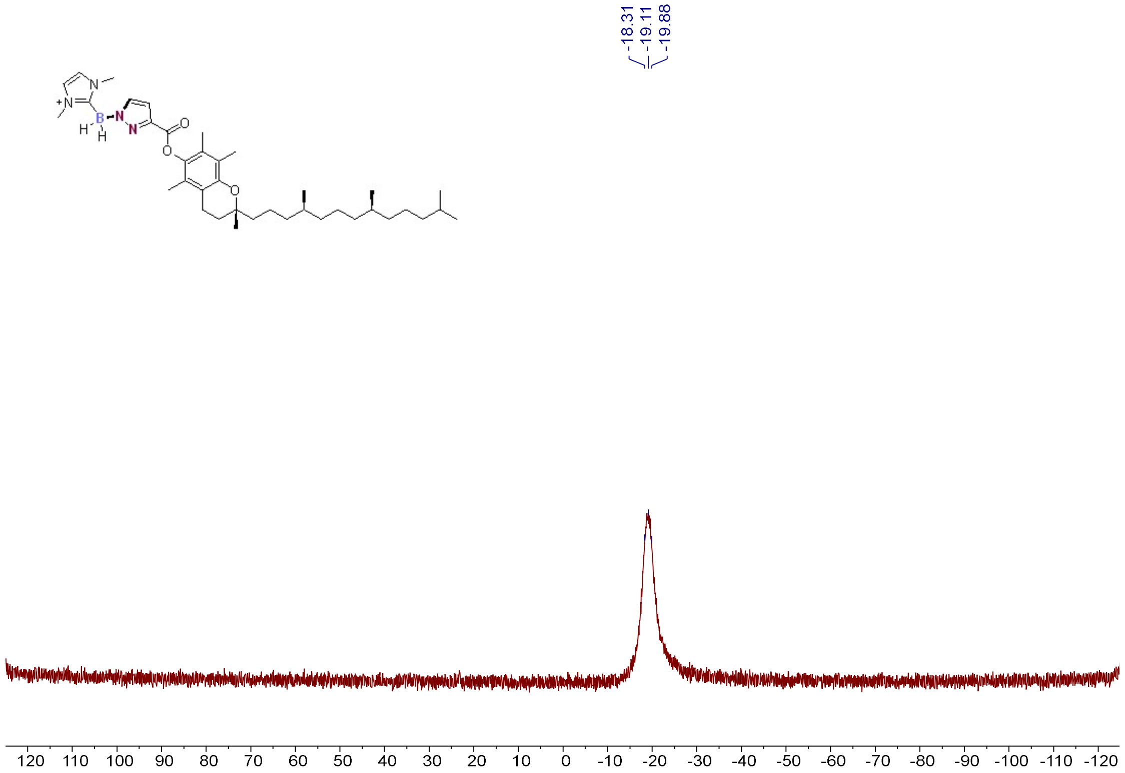

^{1}H NMR (400 MHz) Spectrum of 29 in $CDCl_3$

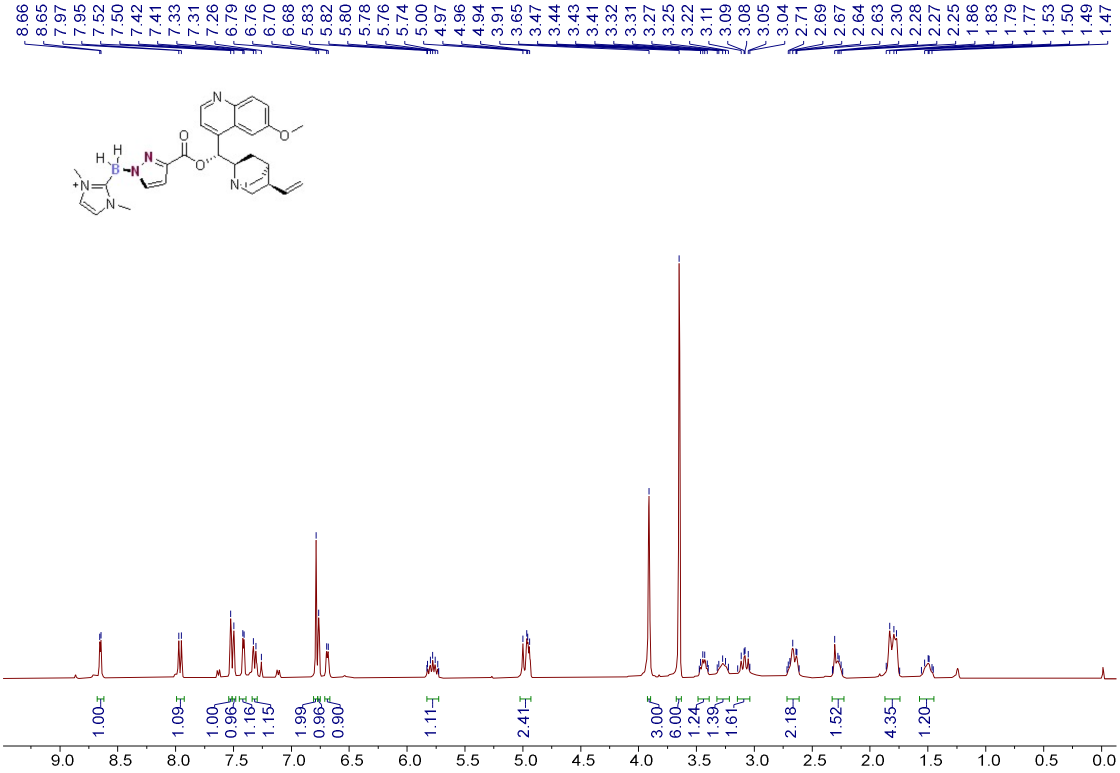

^{13}C NMR (100 MHz) Spectrum of 29 in $CDCl_3$

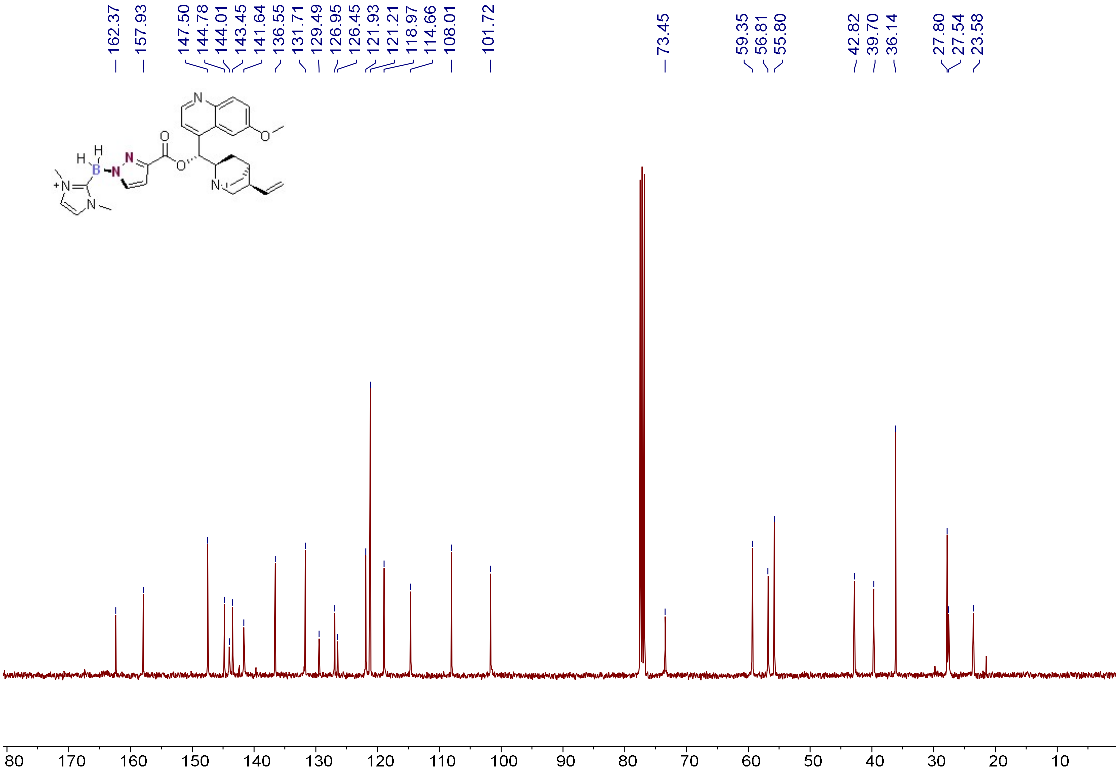

^{11}B NMR (128.4 MHz MHz) Spectrum of 29 in $CDCl_3$

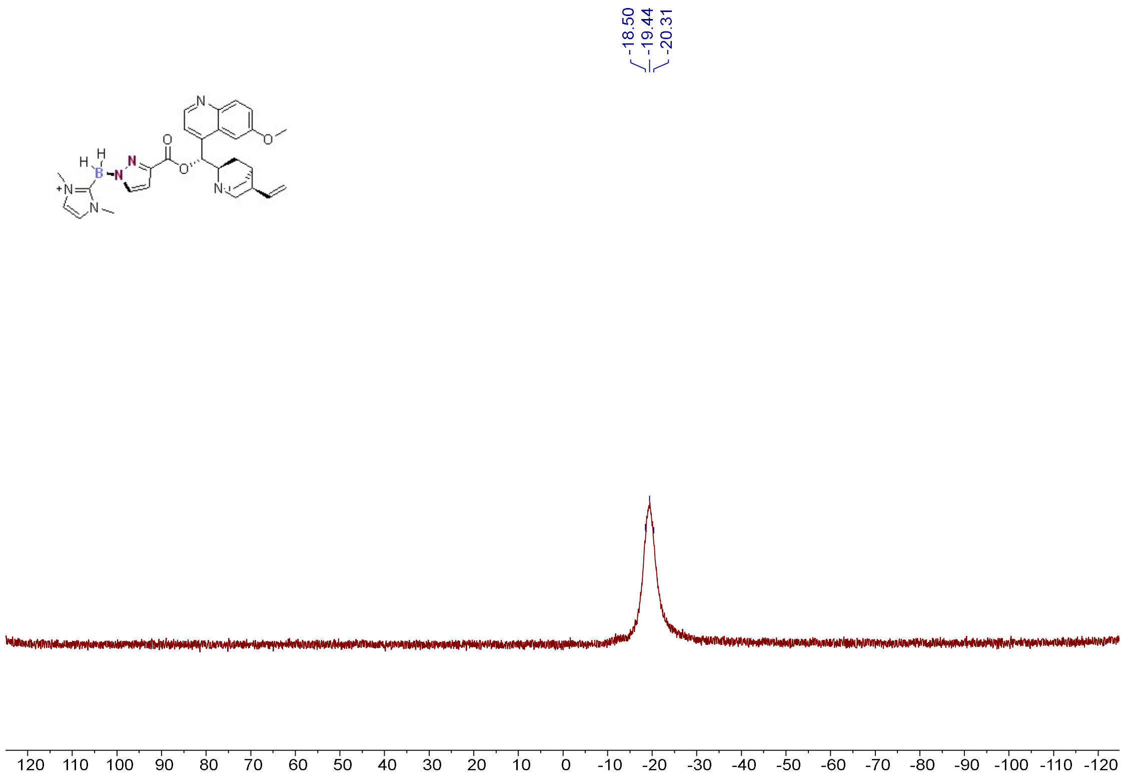

^{1}H NMR (400 MHz) Spectrum of 30 in $CDCl_3$

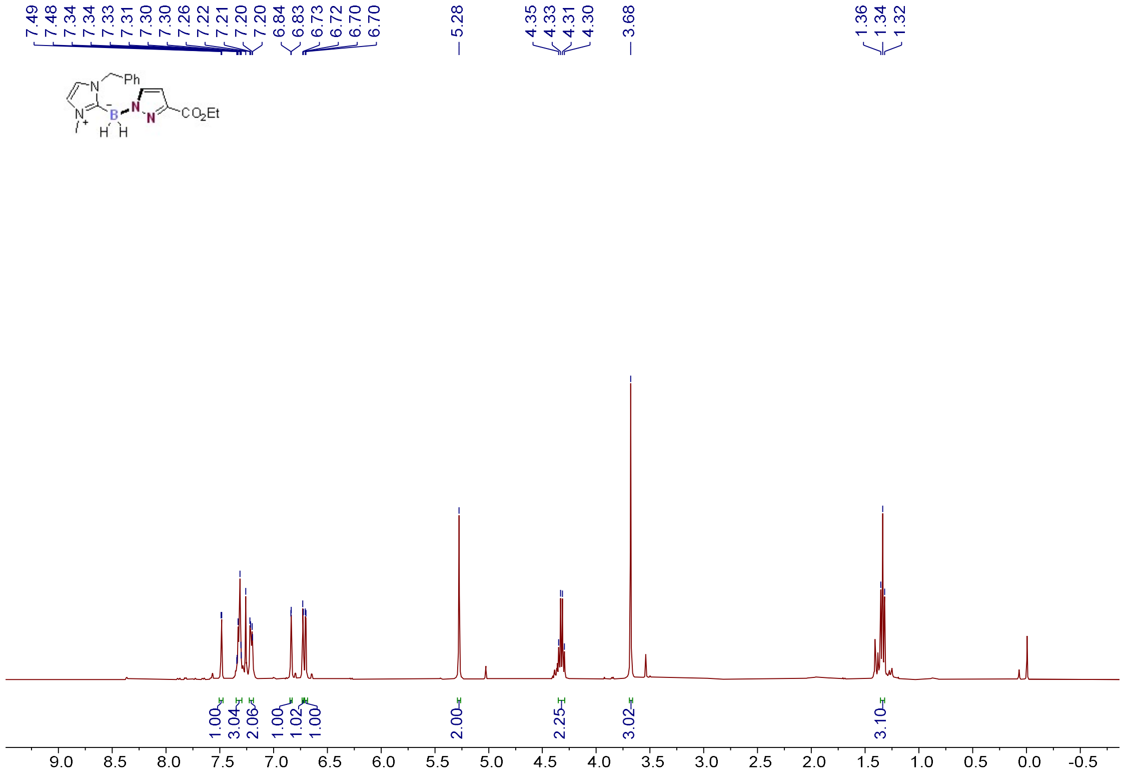

^{13}C NMR (100 MHz) Spectrum of 30 in CDCl$_3$

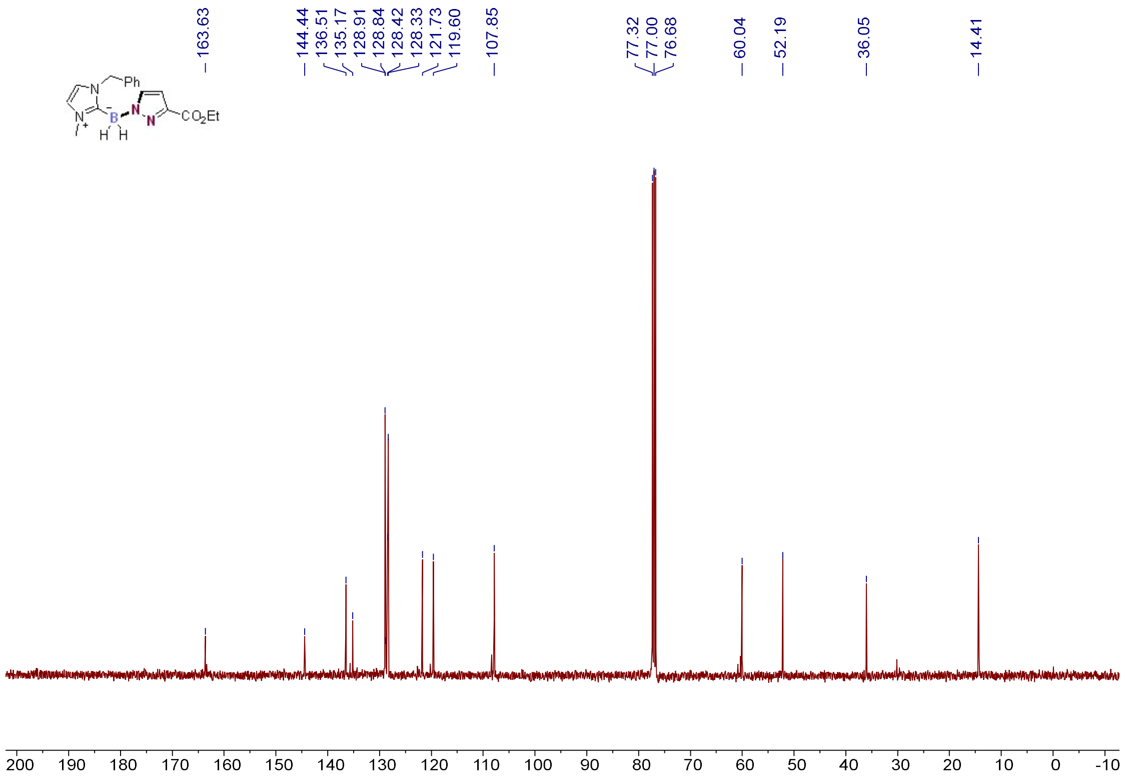

^{11}B NMR (128.4 MHz) Spectrum of 30 in CDCl$_3$

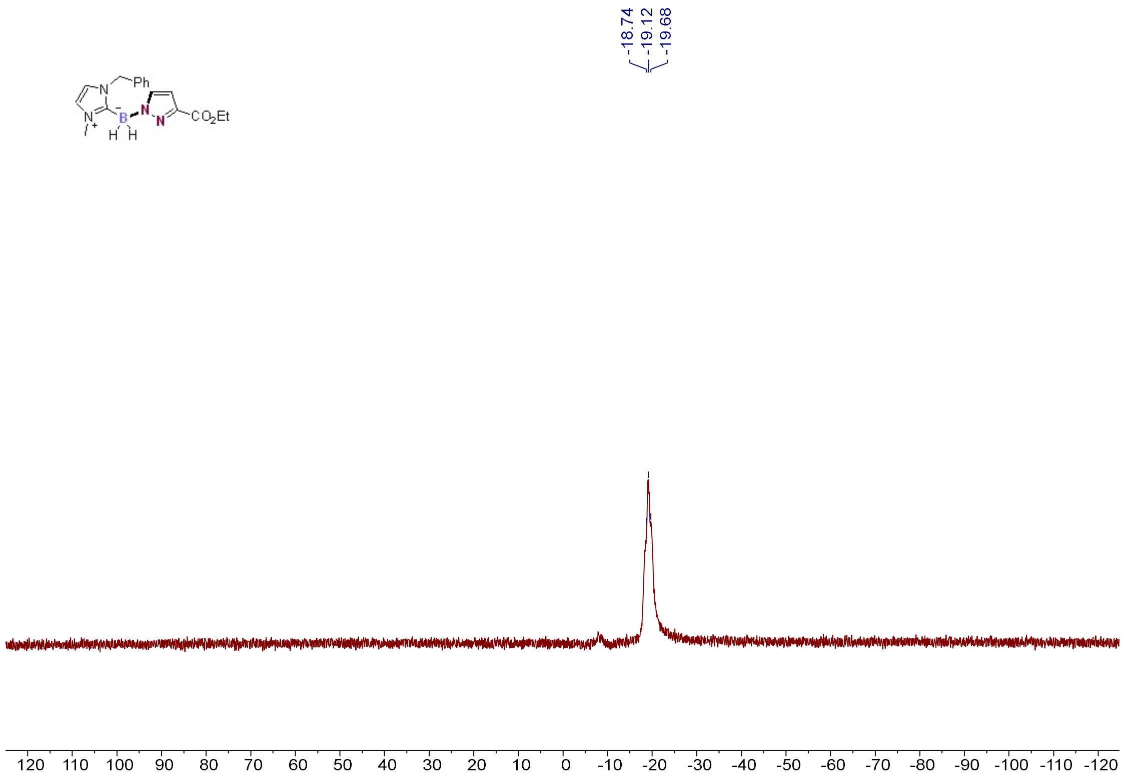

^{1}H NMR (400 MHz) Spectrum of 31 in $CDCl_3$

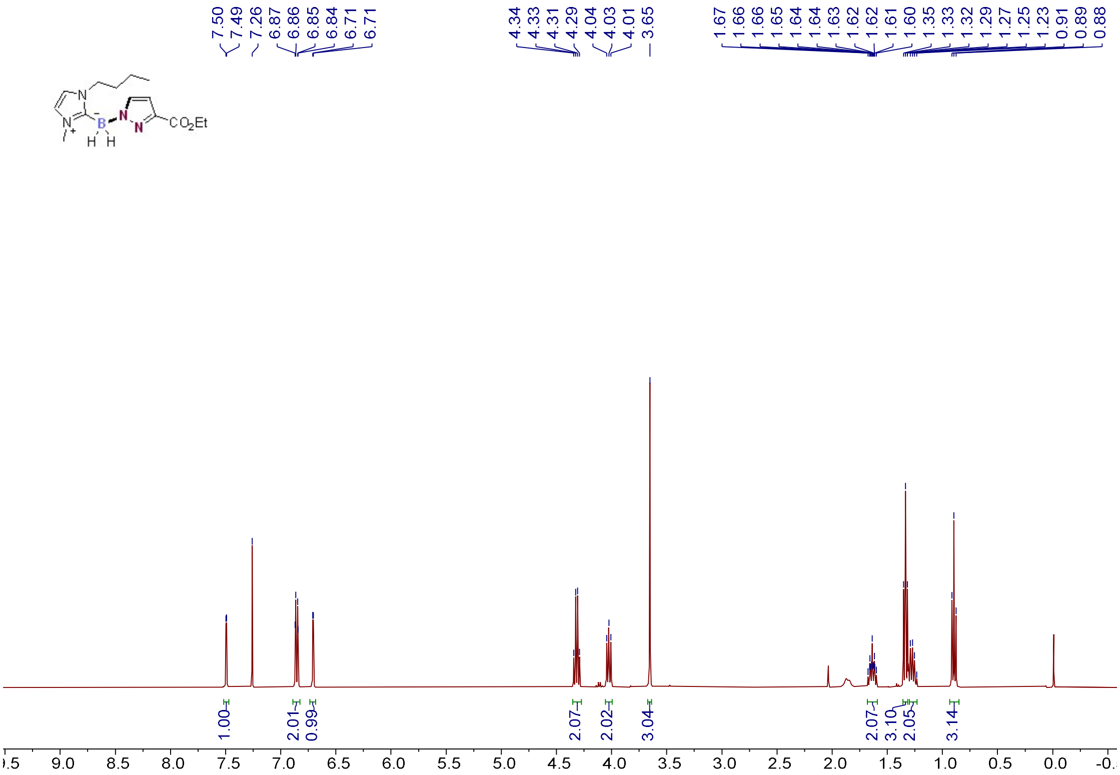

^{13}C NMR (100 MHz) Spectrum of 31 in $CDCl_3$

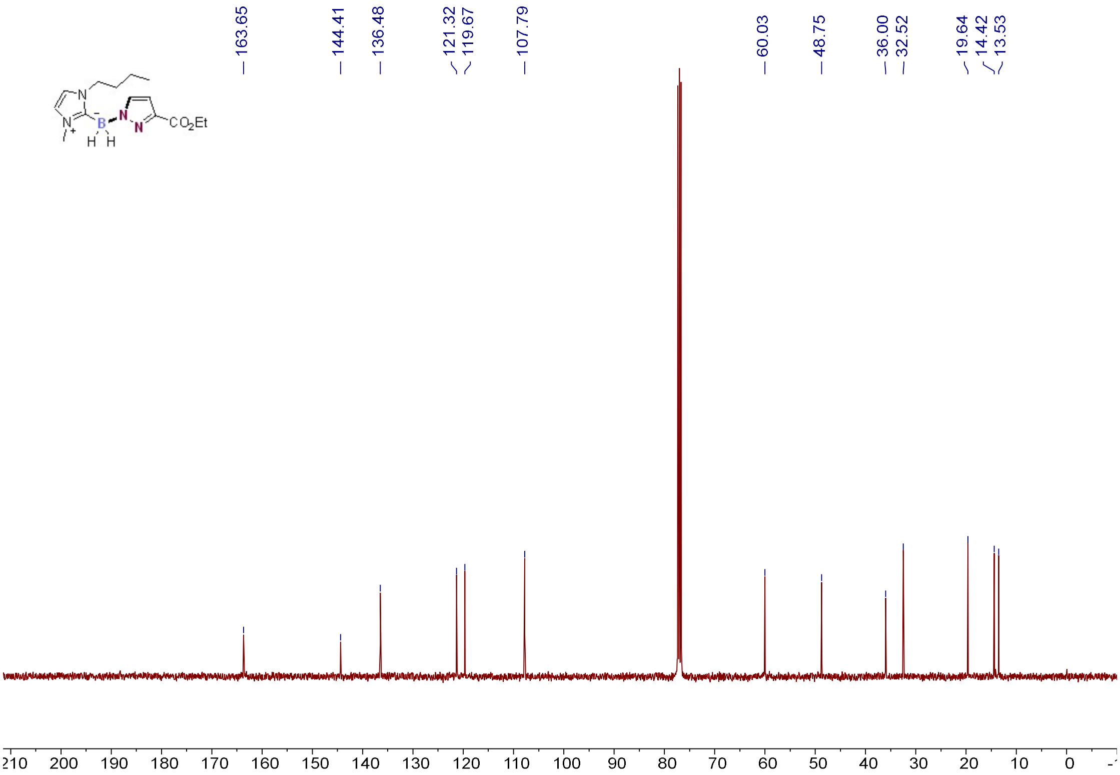

^{11}B NMR (128.4 MHz) Spectrum of 31 in $CDCl_3$

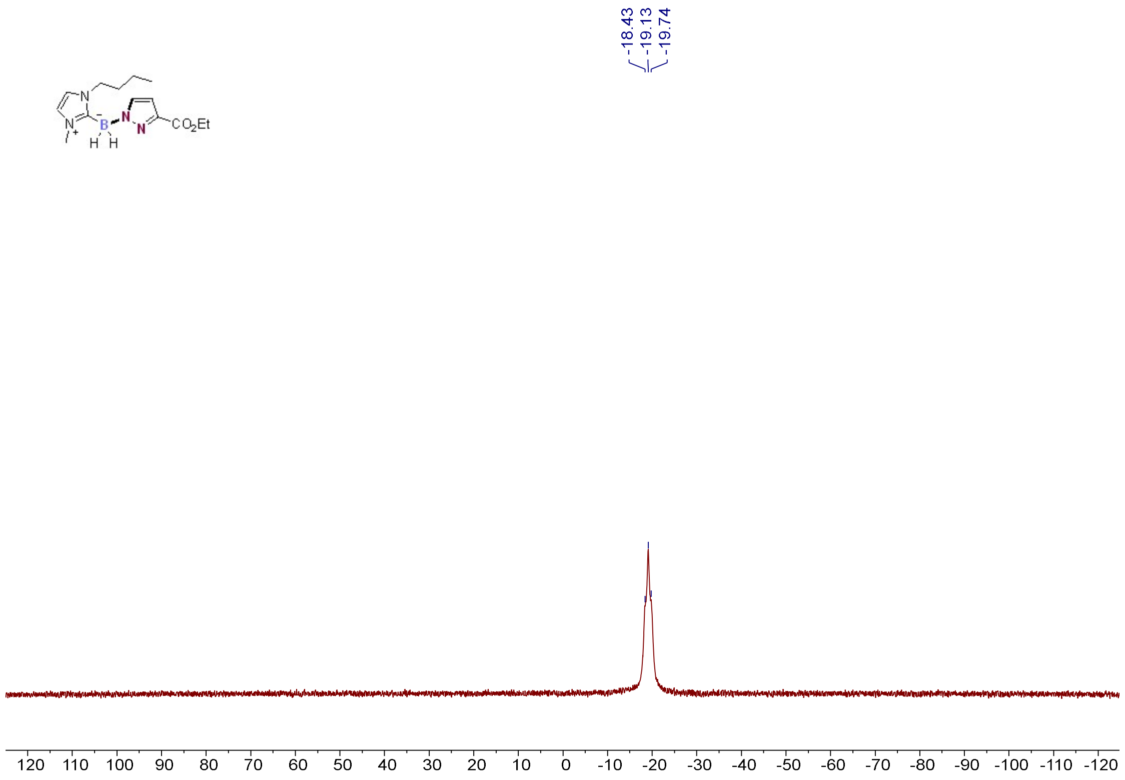

^{1}H NMR (400 MHz) Spectrum of 32 in $CDCl_3$

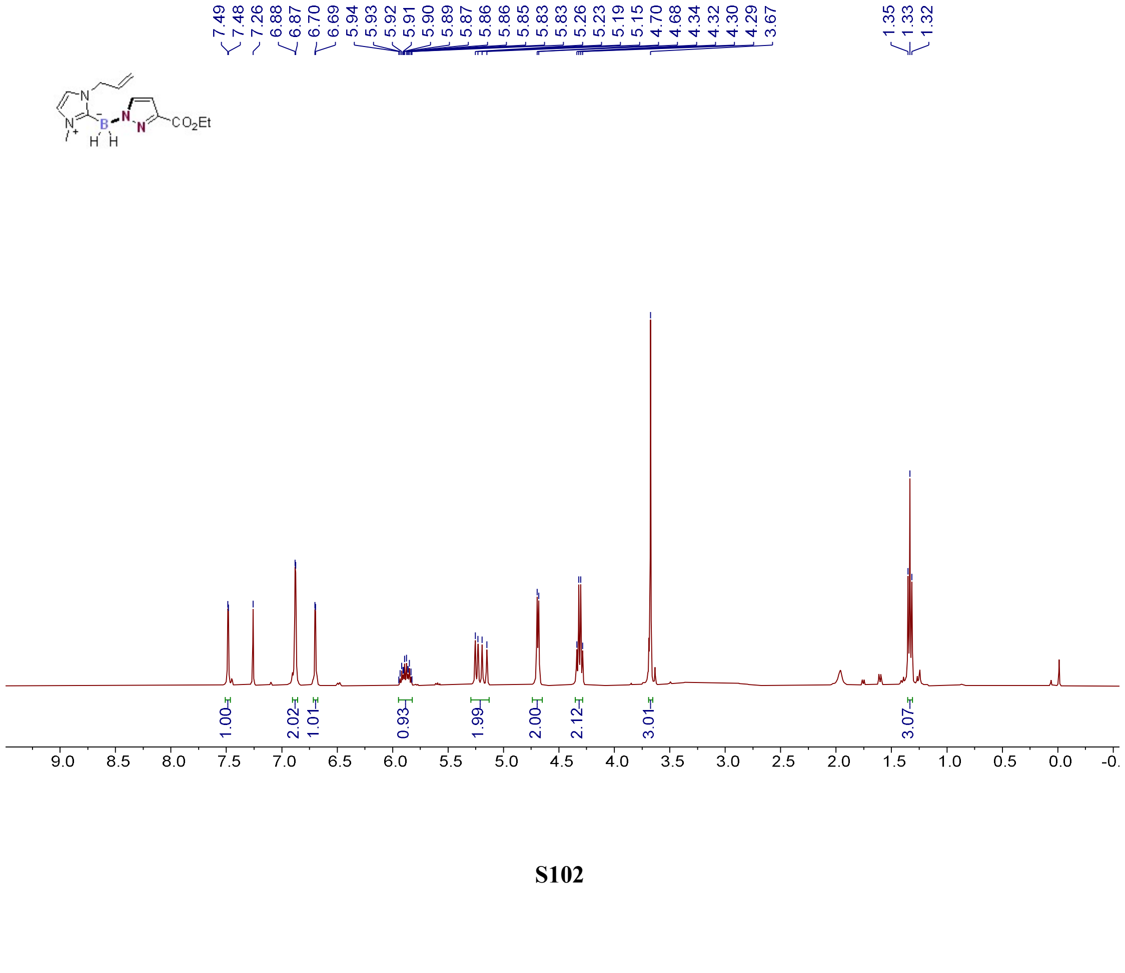

^{13}C NMR (100 MHz) Spectrum of 32 in $CDCl_3$

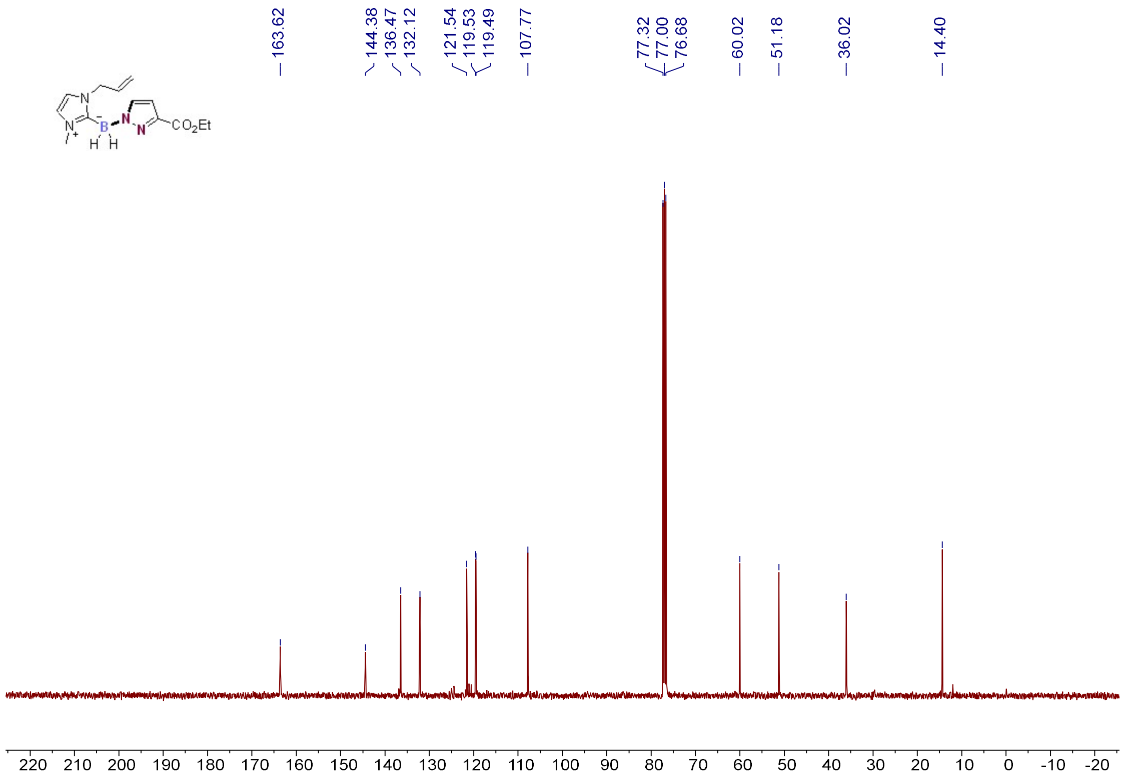

^{11}B NMR (128.4 MHz) Spectrum of 32 in $CDCl_3$

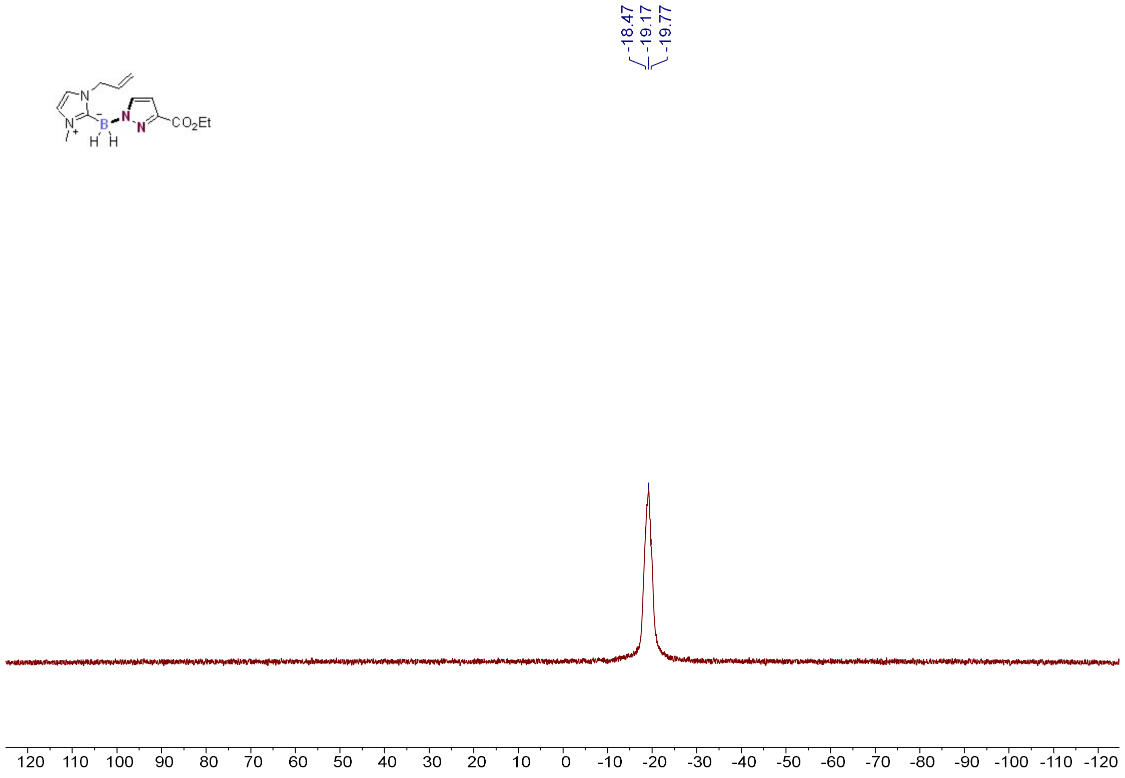

^{1}H NMR (400 MHz) Spectrum of 33 in $CDCl_3$

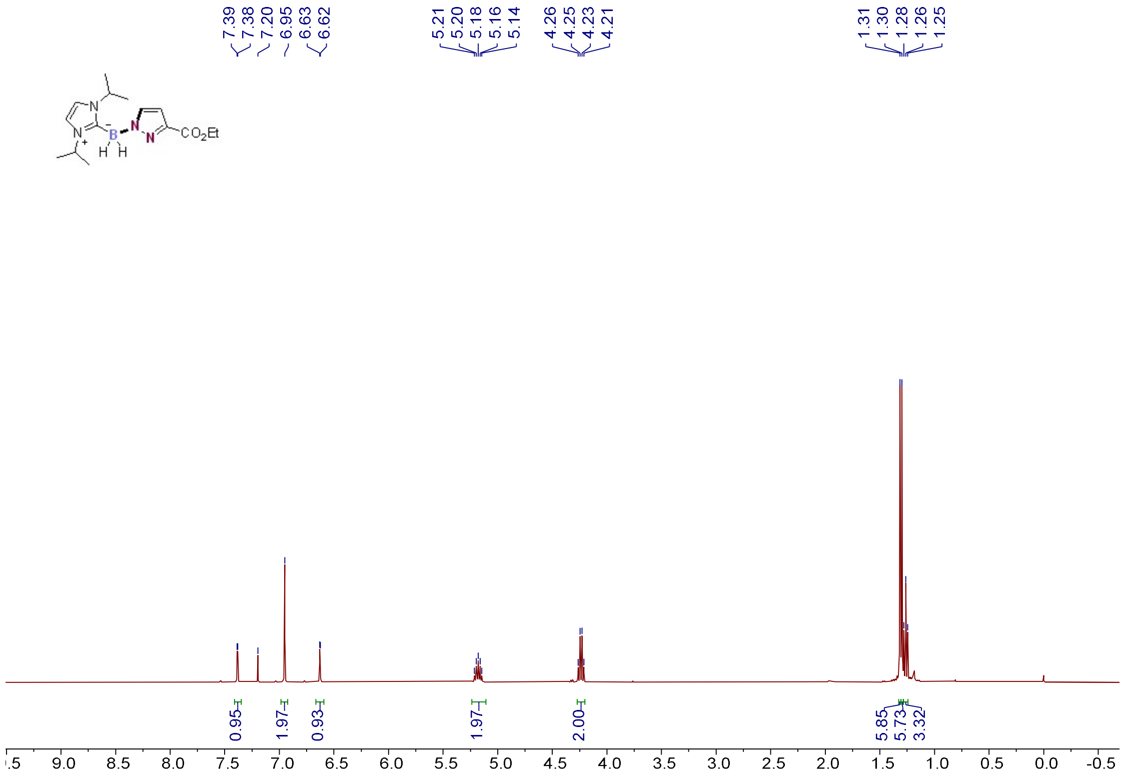

^{13}C NMR (100 MHz) Spectrum of 33 in $CDCl_3$

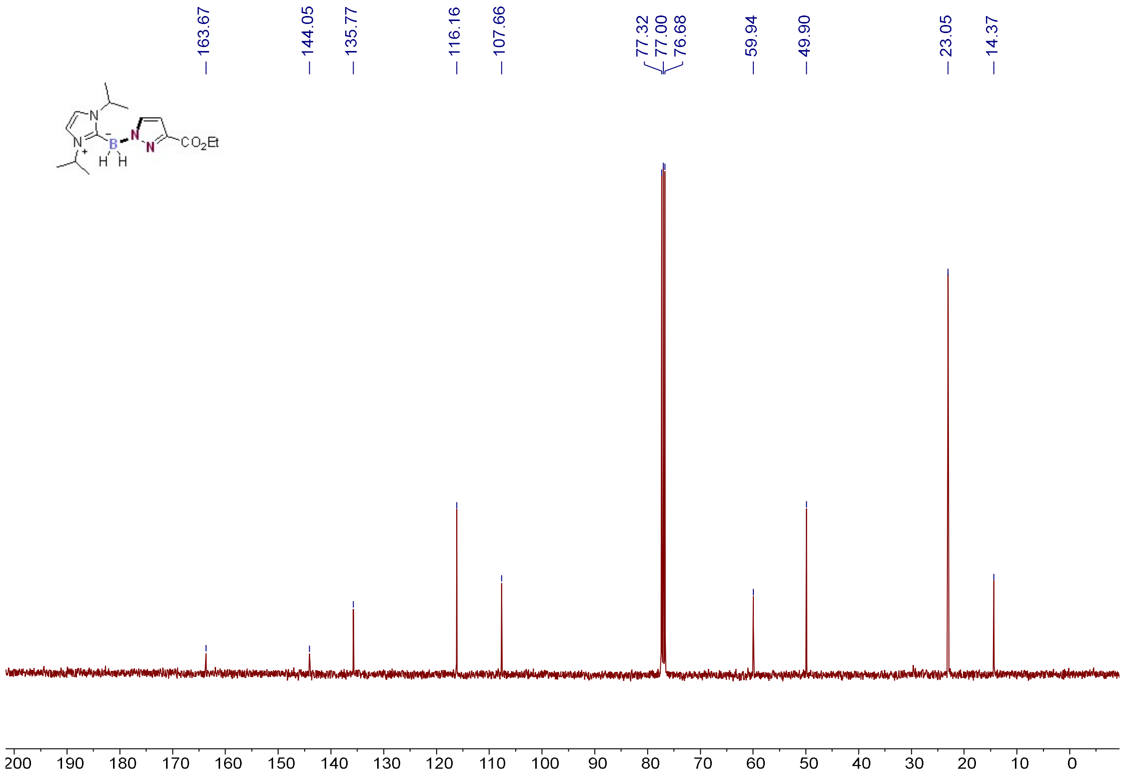

^{11}B NMR (128.4 MHz) Spectrum of 33 in $CDCl_3$

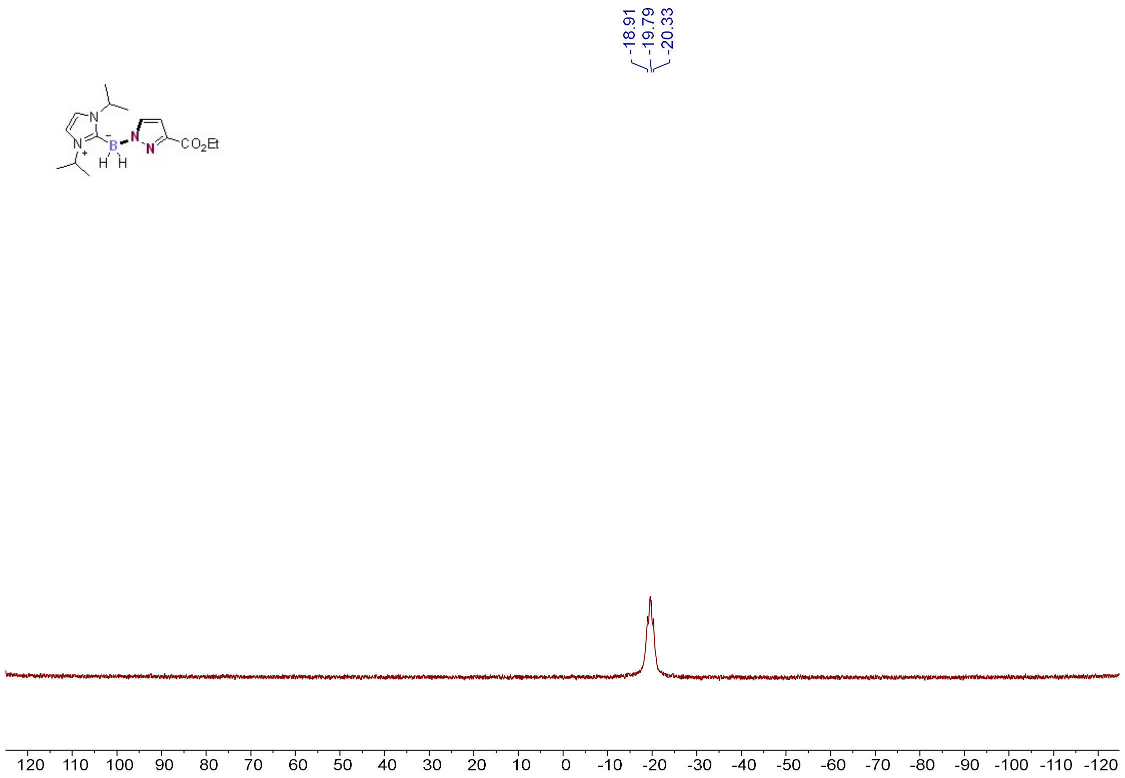

^{1}H NMR (400 MHz) Spectrum of 34 in $CDCl_3$

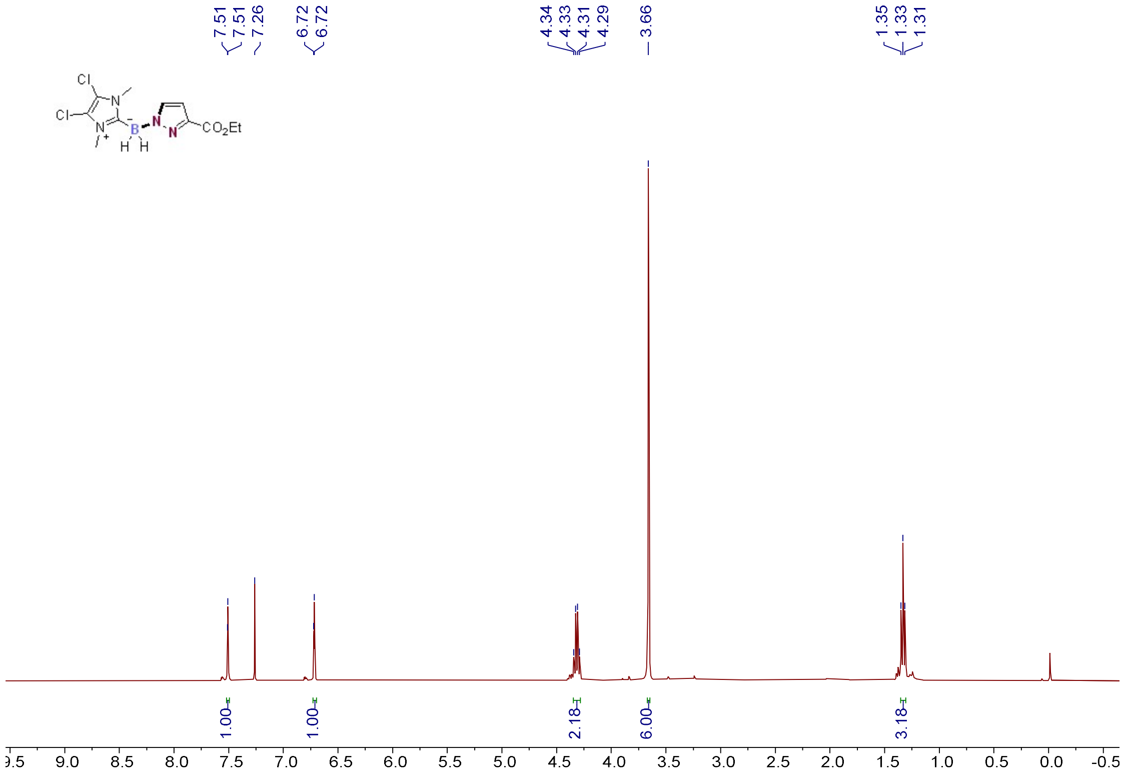

^{13}C NMR (100 MHz) Spectrum of 34 in $CDCl_3$

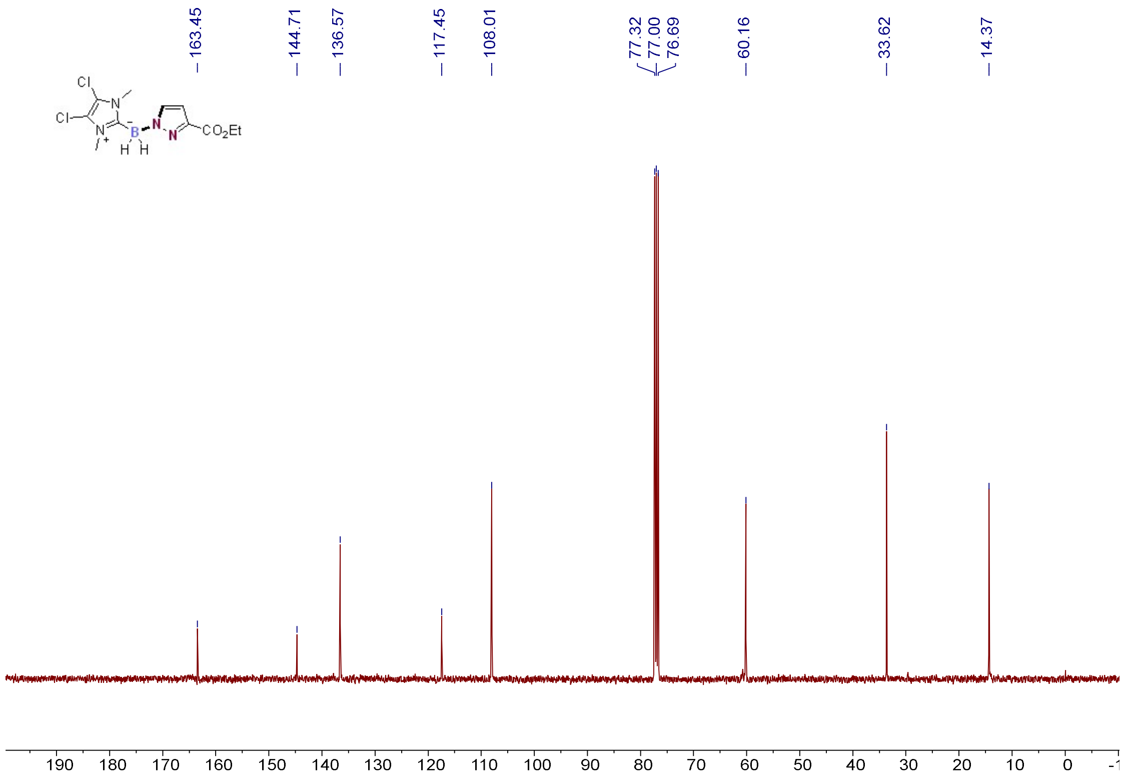

^{11}B NMR (128.4 MHz) Spectrum of 34 in $CDCl_3$

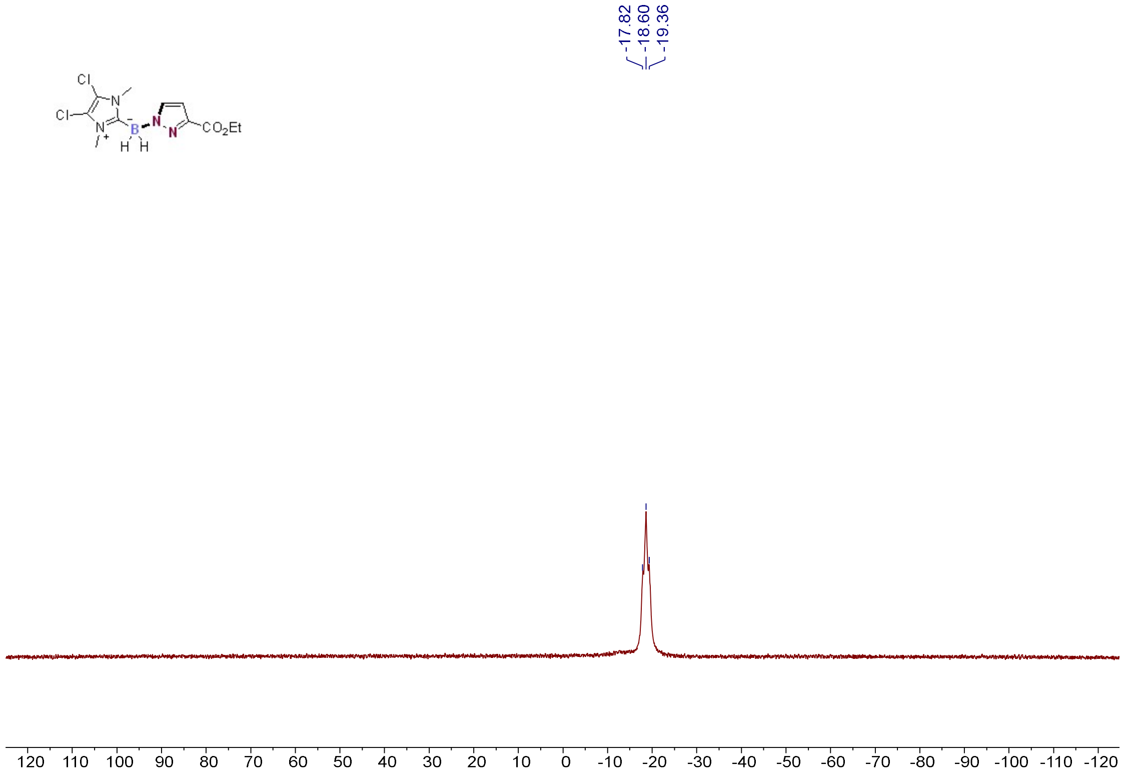

^{1}H NMR (400 MHz) Spectrum of 35 in $CDCl_3$

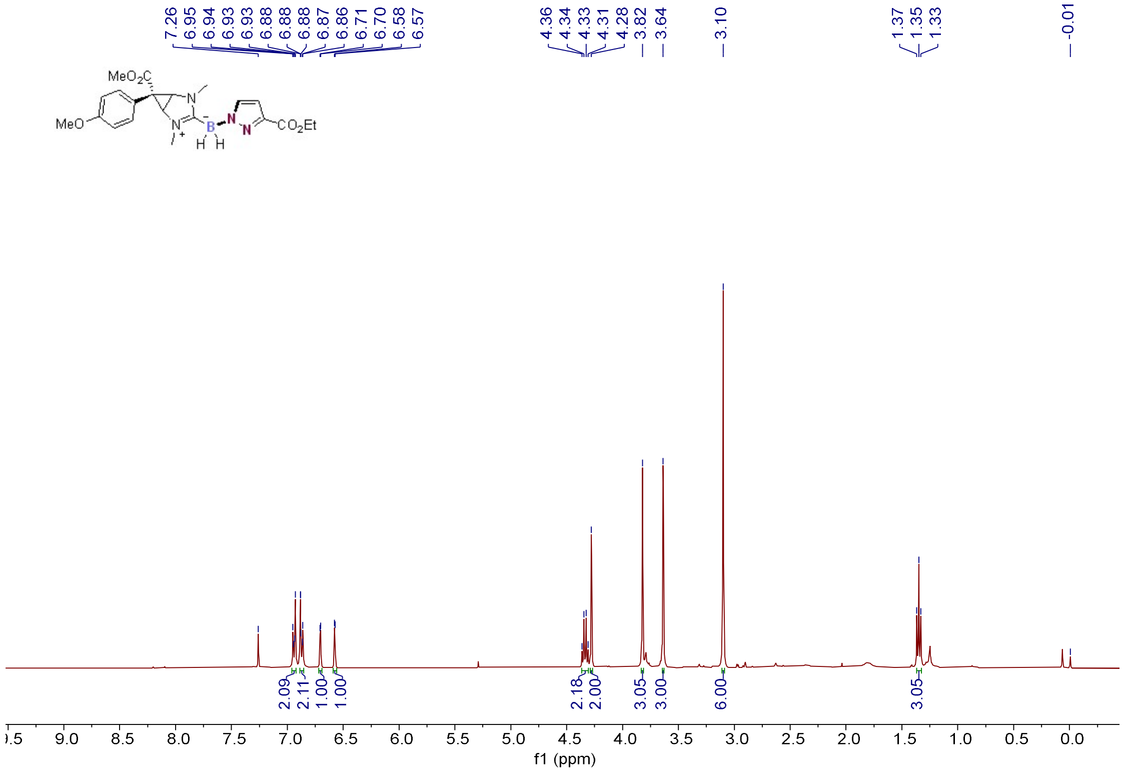

^{13}C NMR (100 MHz) Spectrum of 35 in $CDCl_3$

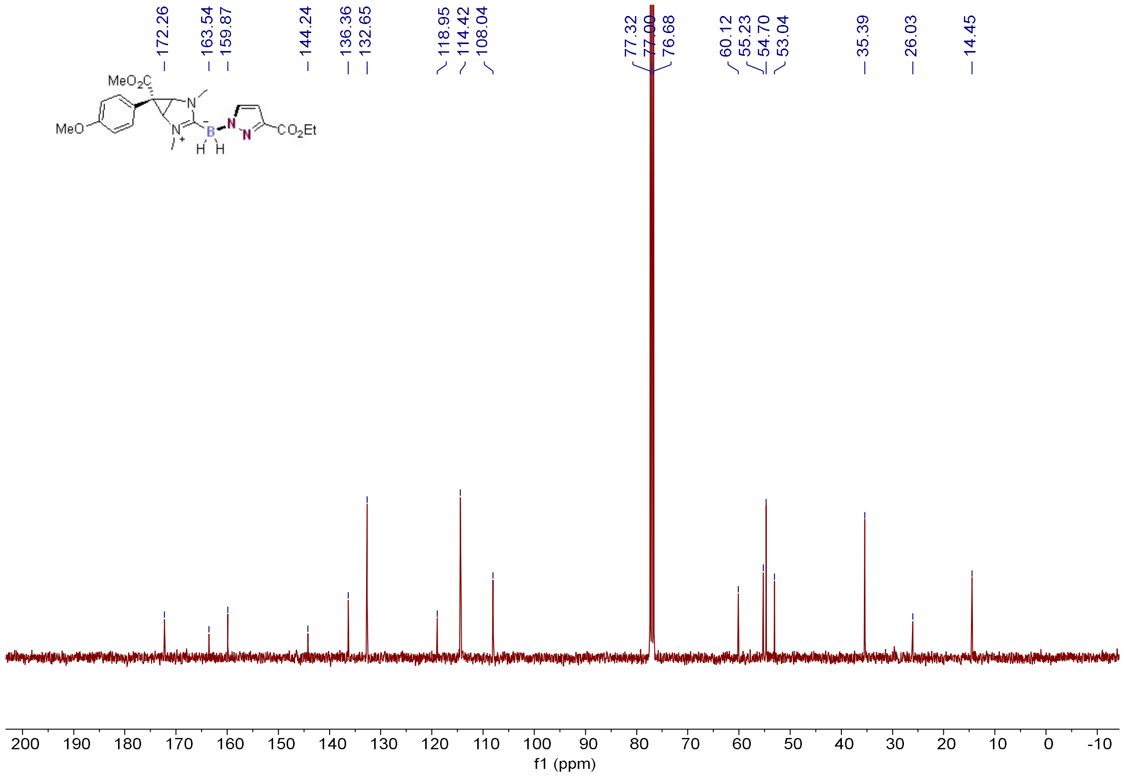

^{11}B NMR (128.4 MHz) Spectrum of 35 in $CDCl_3$

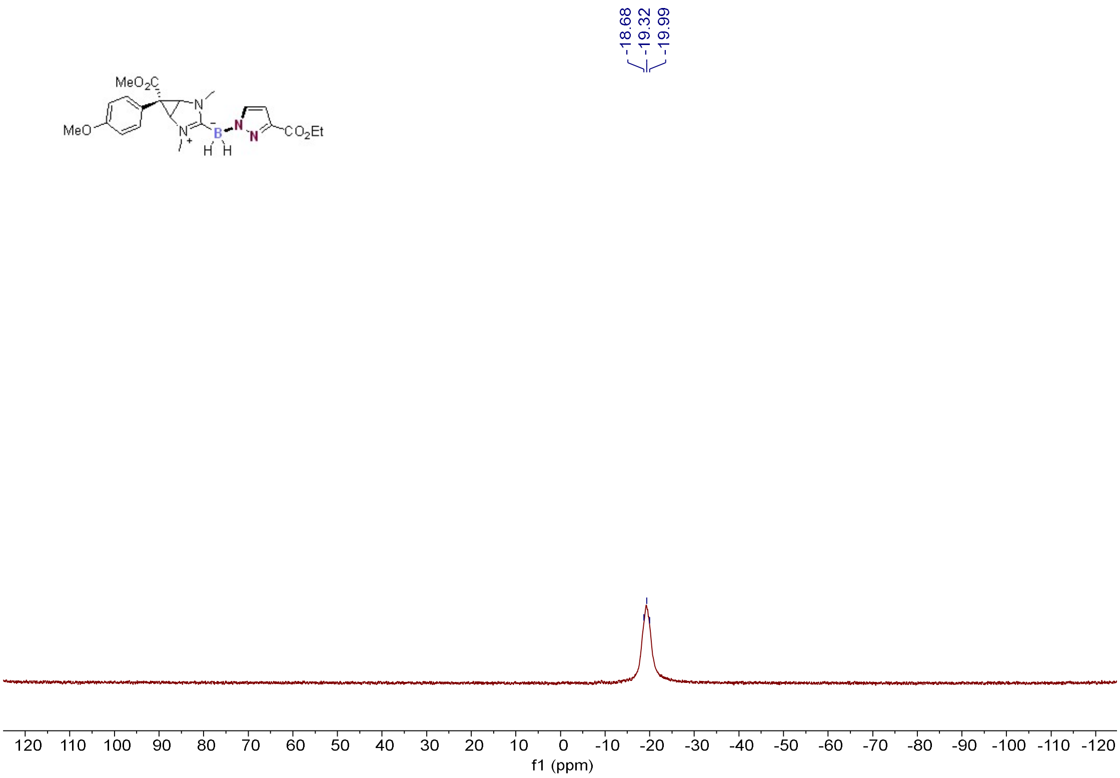

^{1}H NMR (400 MHz) Spectrum of 36 in $CDCl_3$

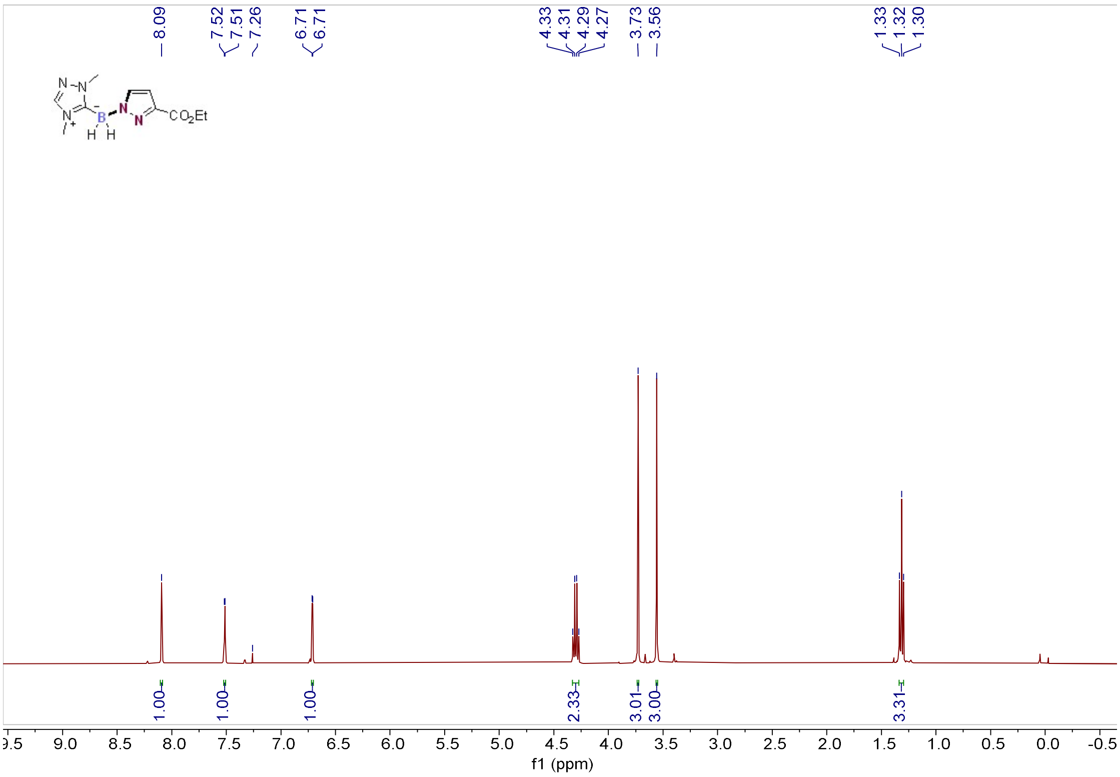

^{13}C NMR (100 MHz) Spectrum of 36 in $CDCl_3$

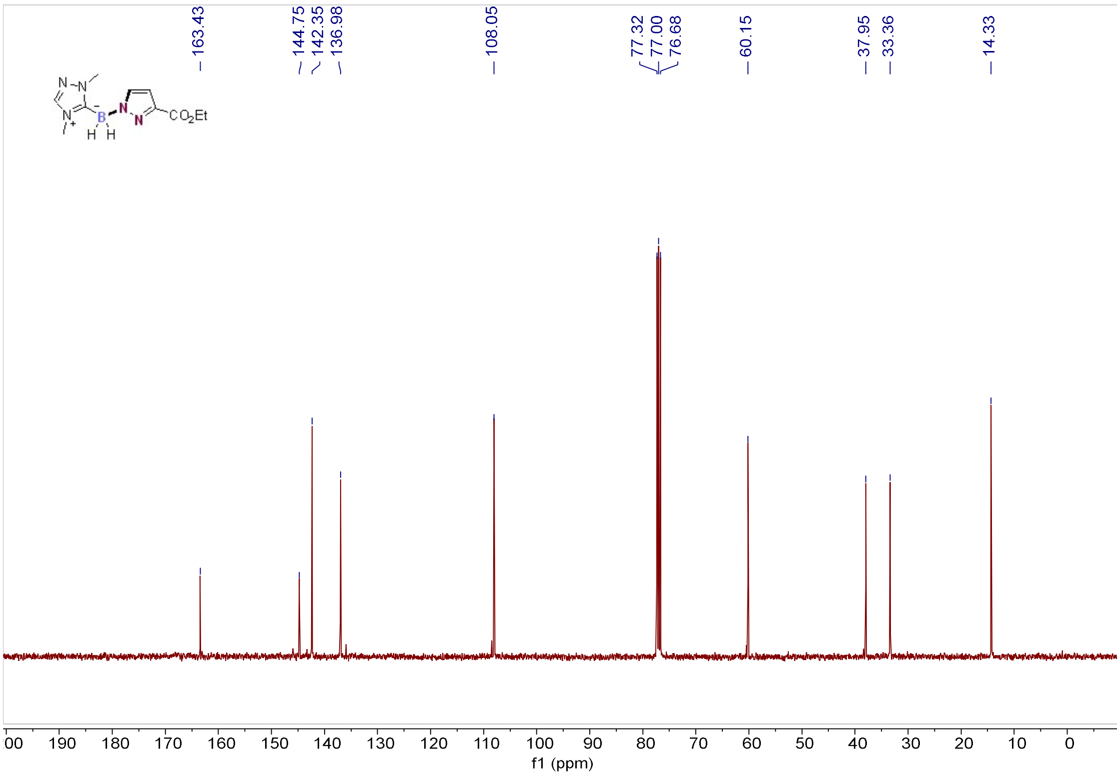

^{11}B NMR (128.4 MHz) Spectrum of 36 in $CDCl_3$

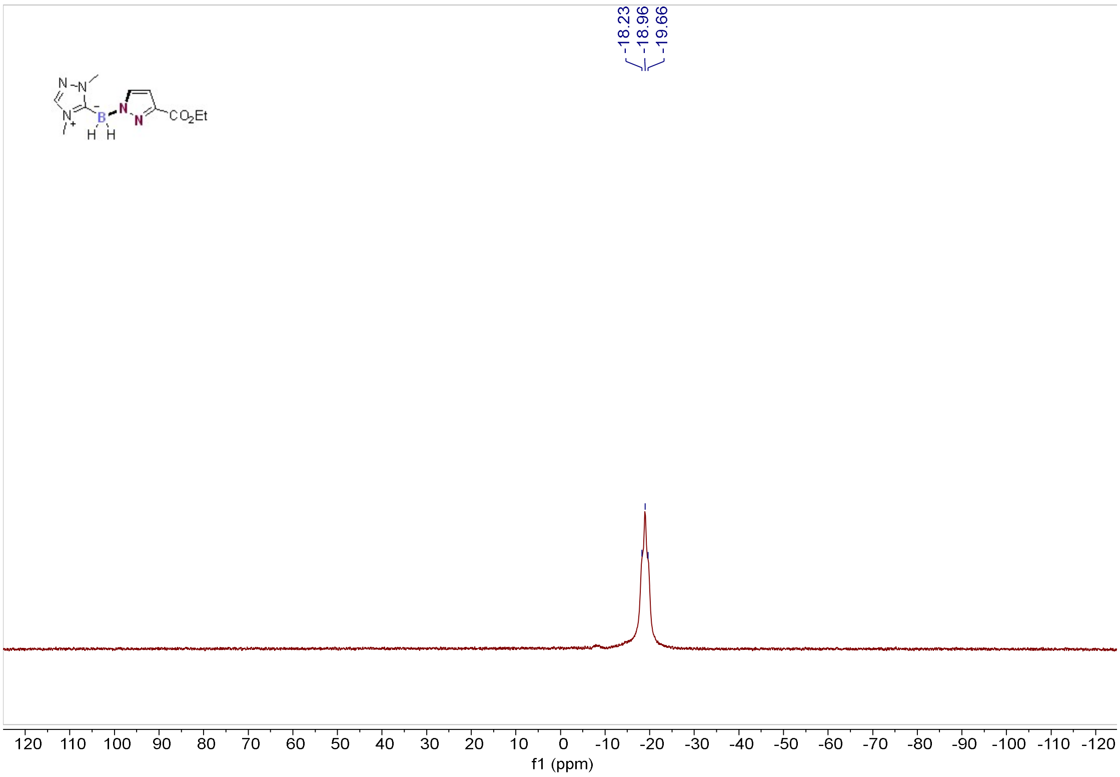

^{1}H NMR (400 MHz) Spectrum of 37 in $CDCl_3$

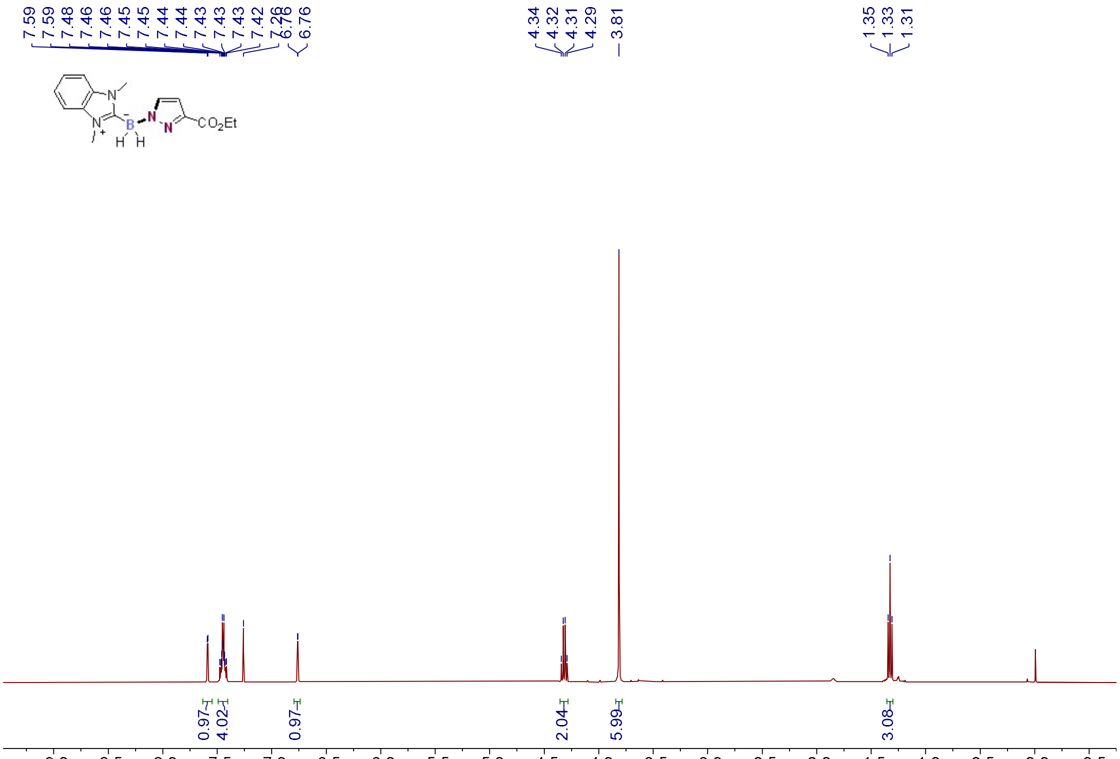

^{13}C NMR (100 MHz) Spectrum of 37 in $CDCl_3$

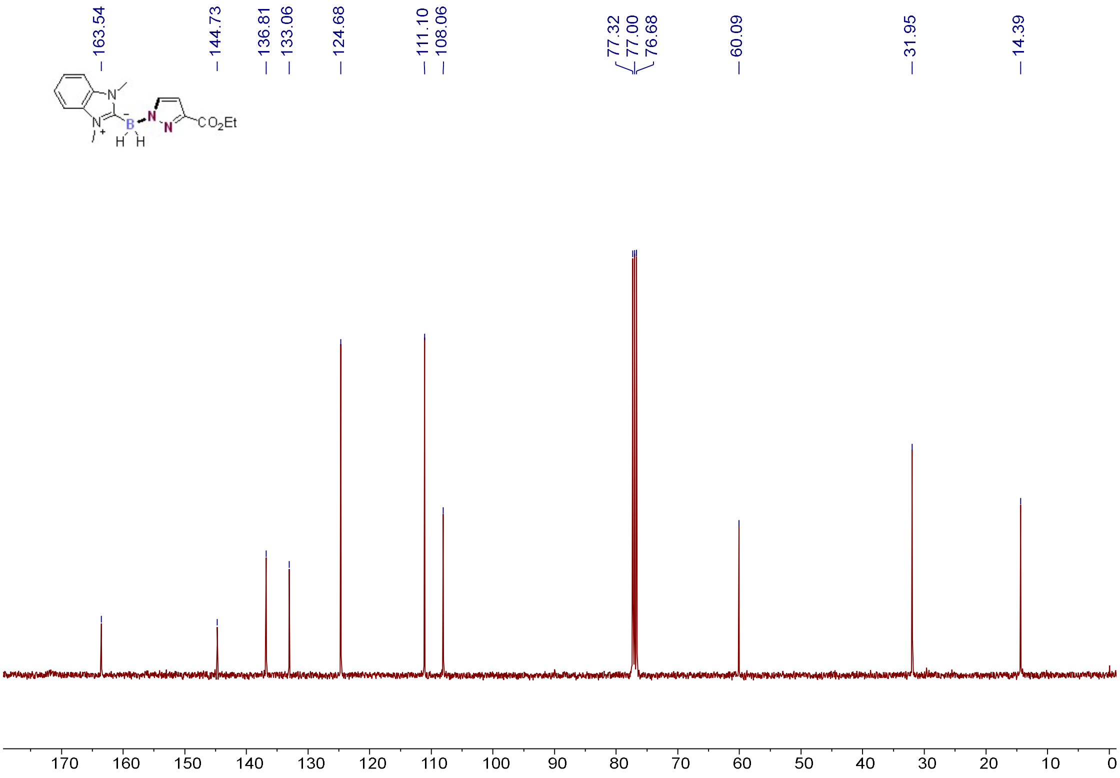

^{11}B NMR (128.4 MHz) Spectrum of 37 in CDCl$_3$

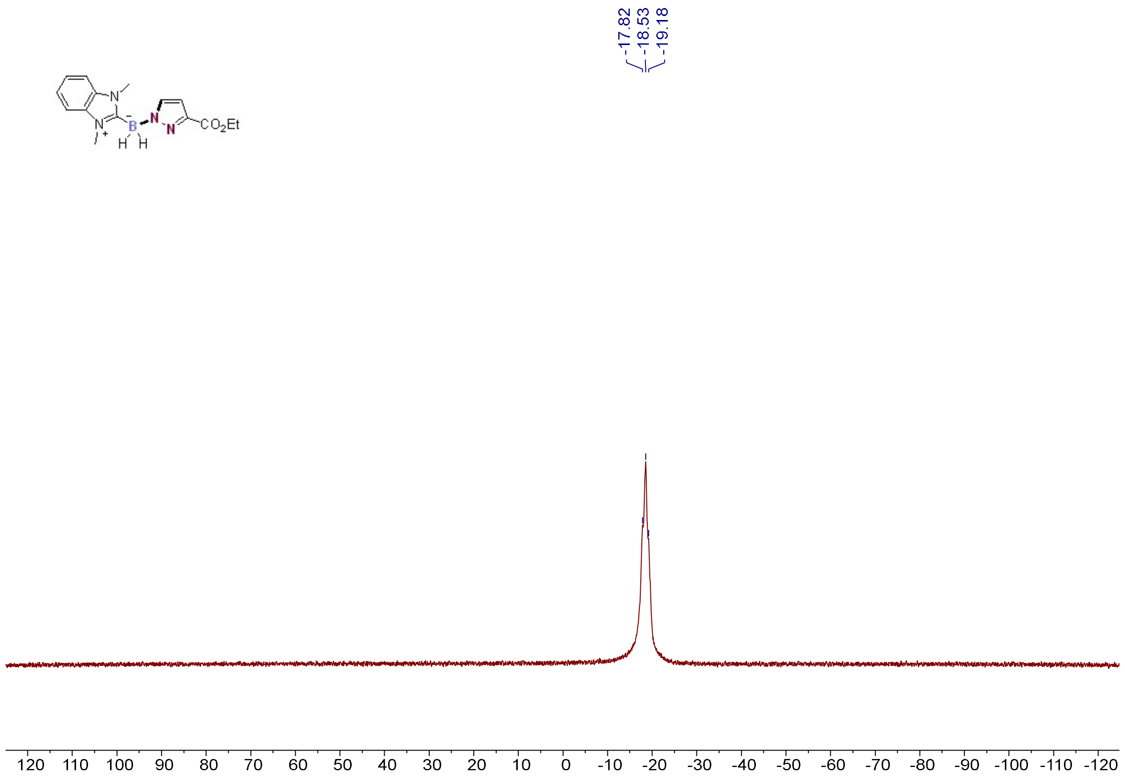

^{1}H NMR (400 MHz) Spectrum of 38 in CDCl$_3$

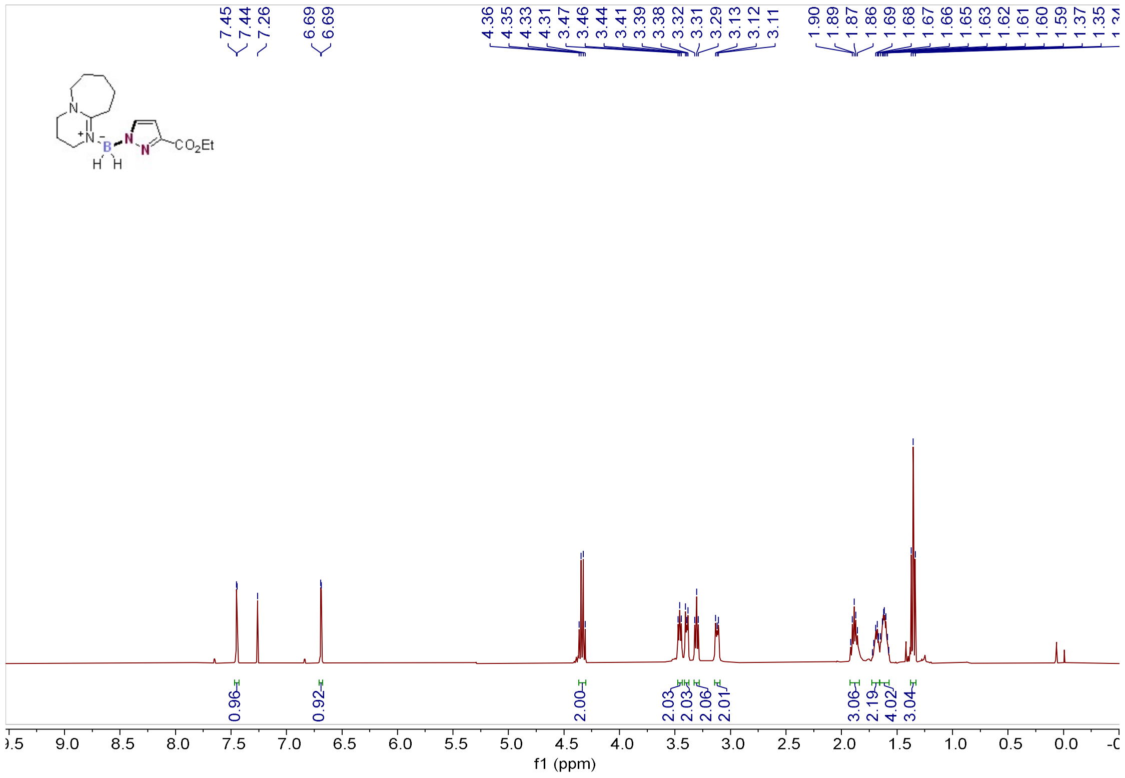

^{13}C NMR (100 MHz) Spectrum of 38 in $CDCl_3$

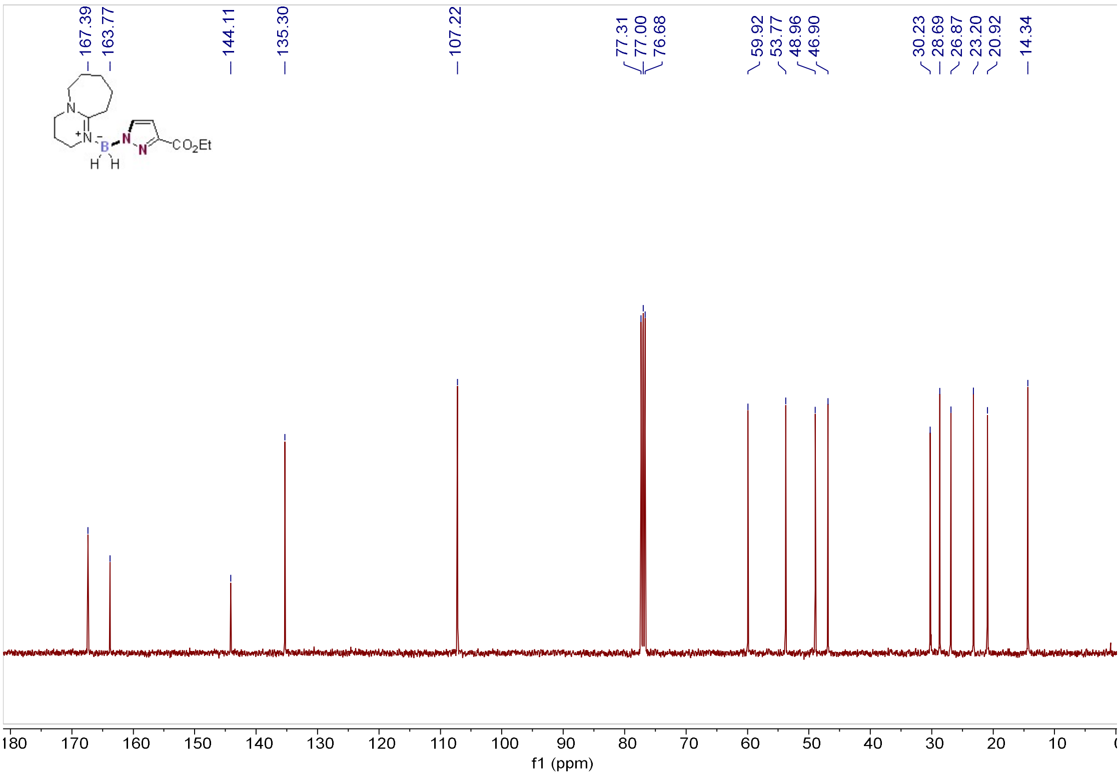

^{11}B NMR (128.4 MHz) Spectrum of 38 in $CDCl_3$

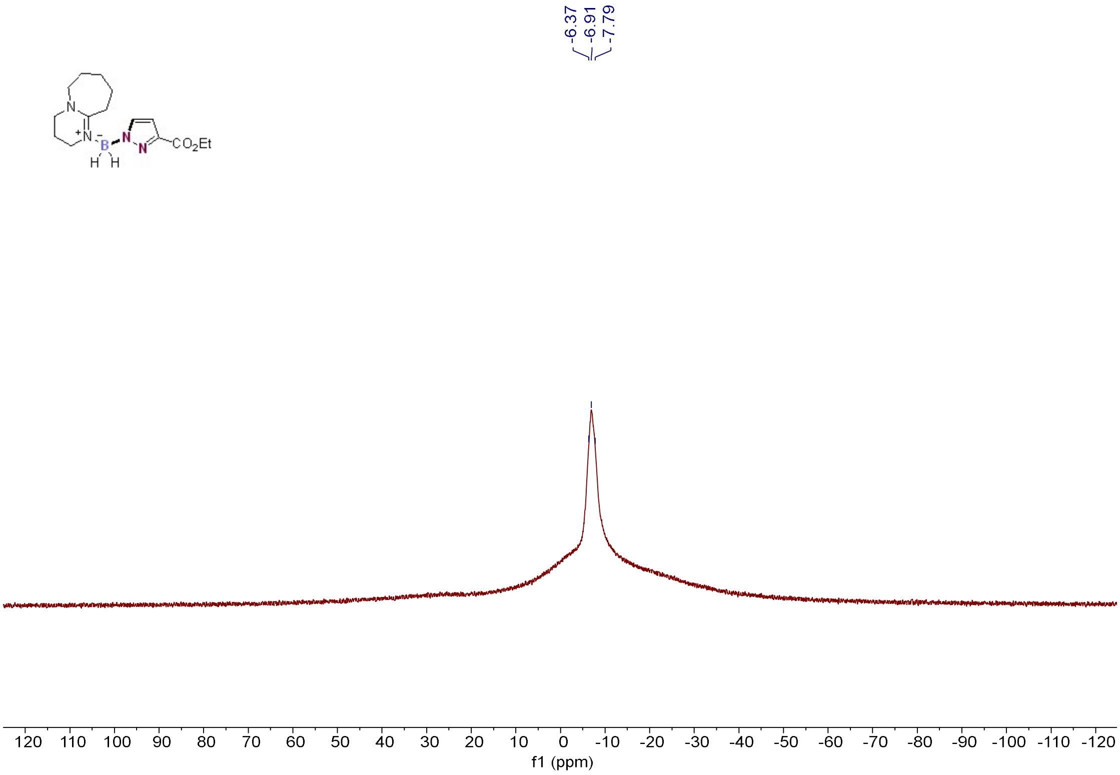

^{1}H NMR (400 MHz) Spectrum of 39 in $CDCl_3$

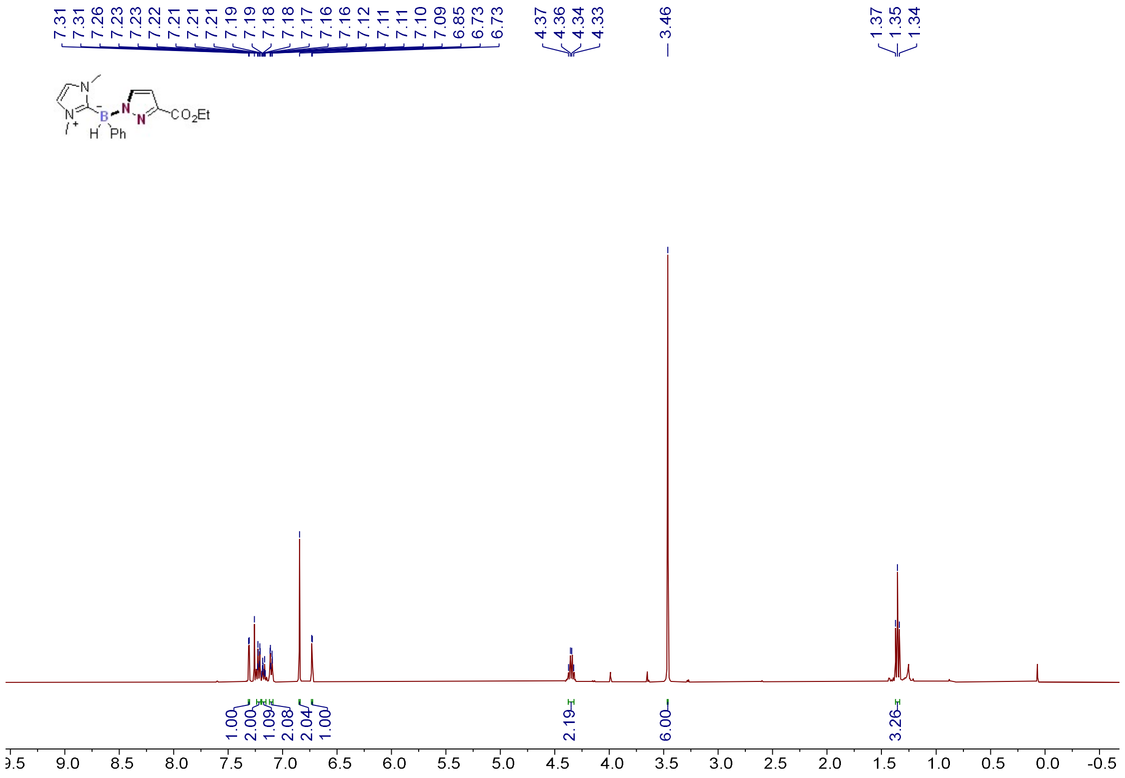

^{13}C NMR (100 MHz) Spectrum of 39 in $CDCl_3$

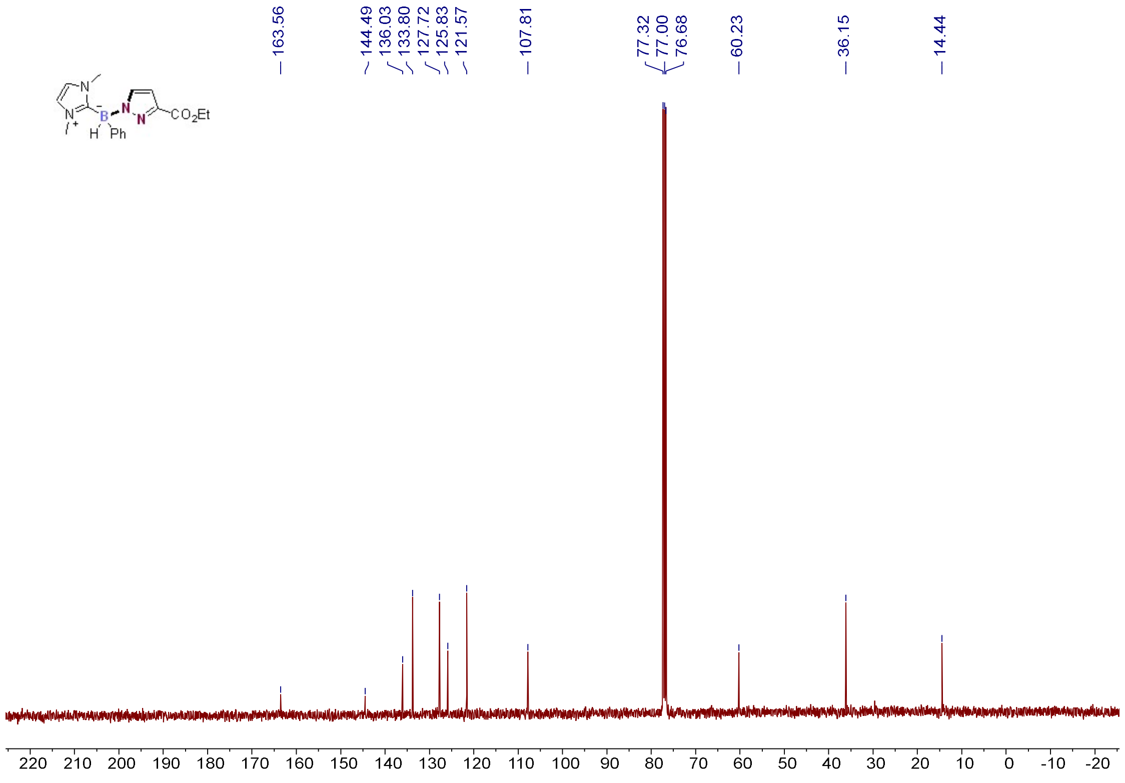

^{11}B NMR (128.4 MHz) Spectrum of 39 in $CDCl_3$

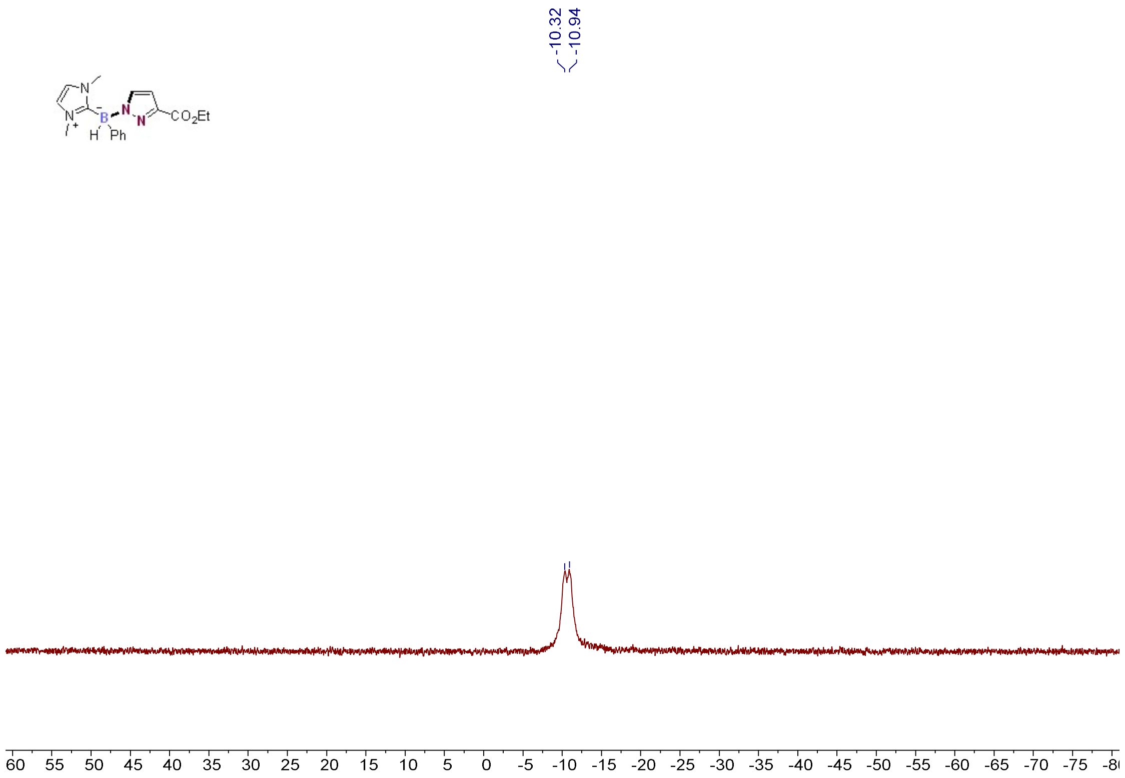

^{1}H NMR (400 MHz) Spectrum of 44 in $CDCl_3$

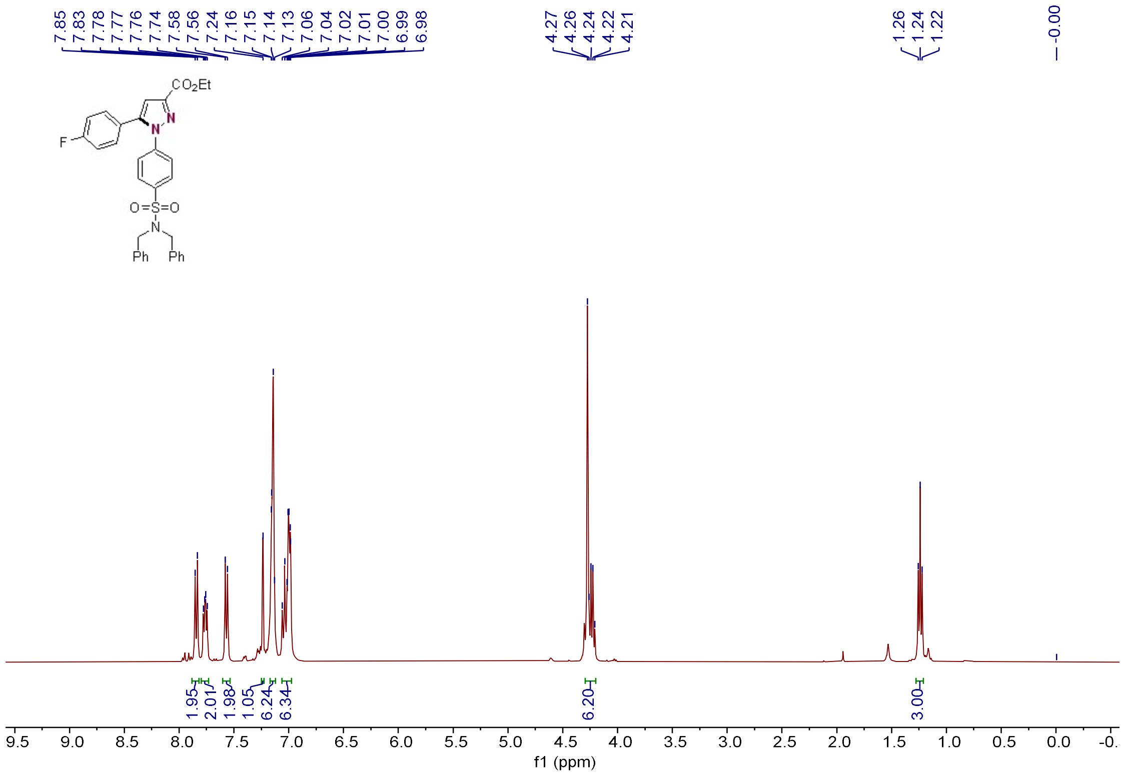

^{13}C NMR (100 MHz) Spectrum of 44 in $CDCl_3$

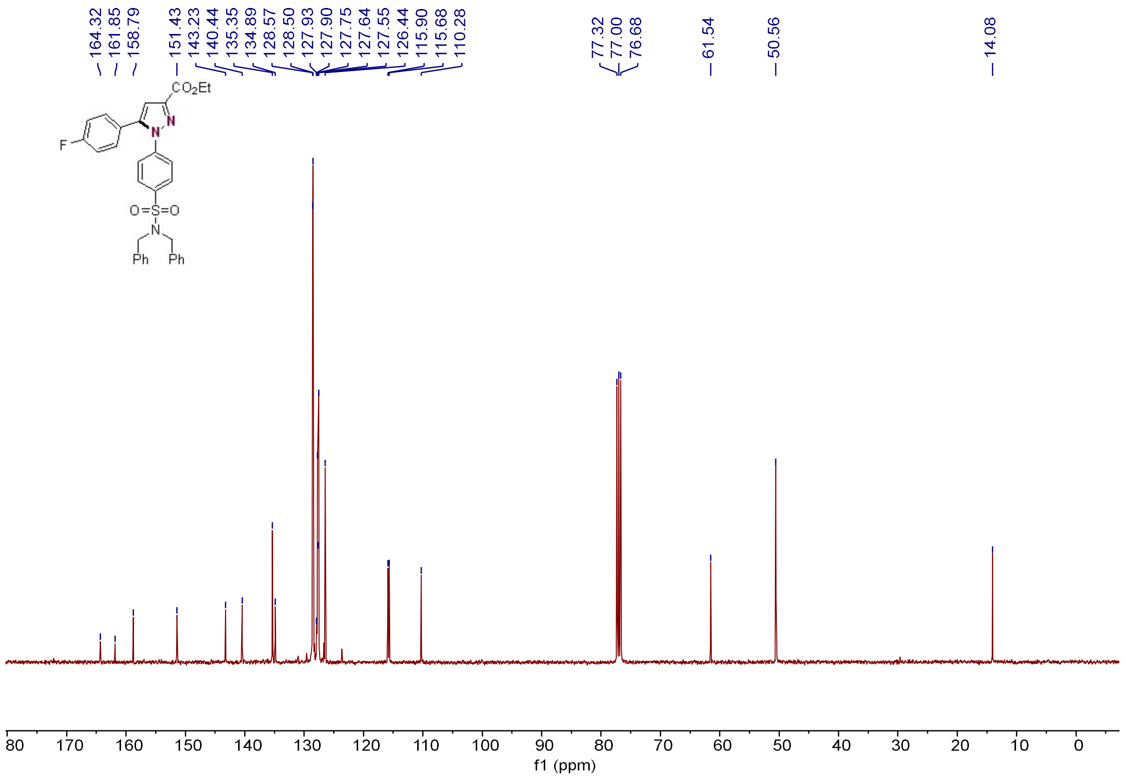

^{19}F NMR (376 MHz) Spectrum of 44 in $CDCl_3$

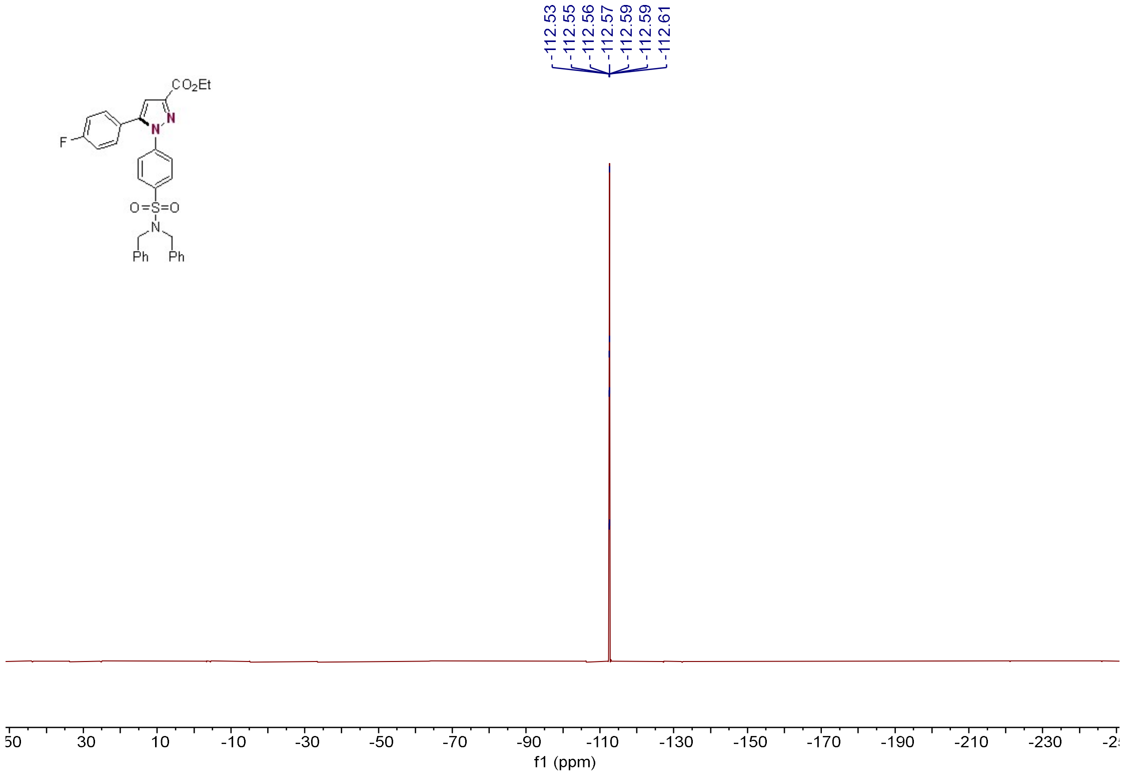

^{1}H NMR (400 MHz) Spectrum of 46 in d_6-DMSO

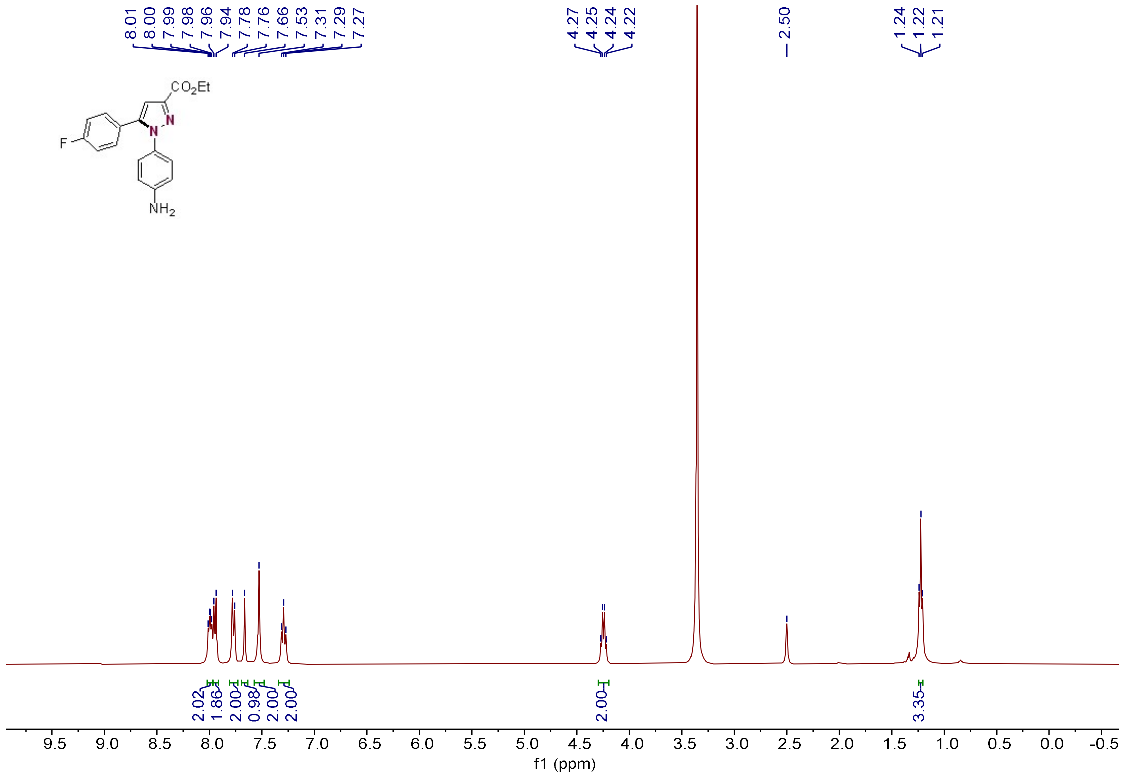

^{13}C NMR (100 MHz) Spectrum of 46 in d_6-DMSO

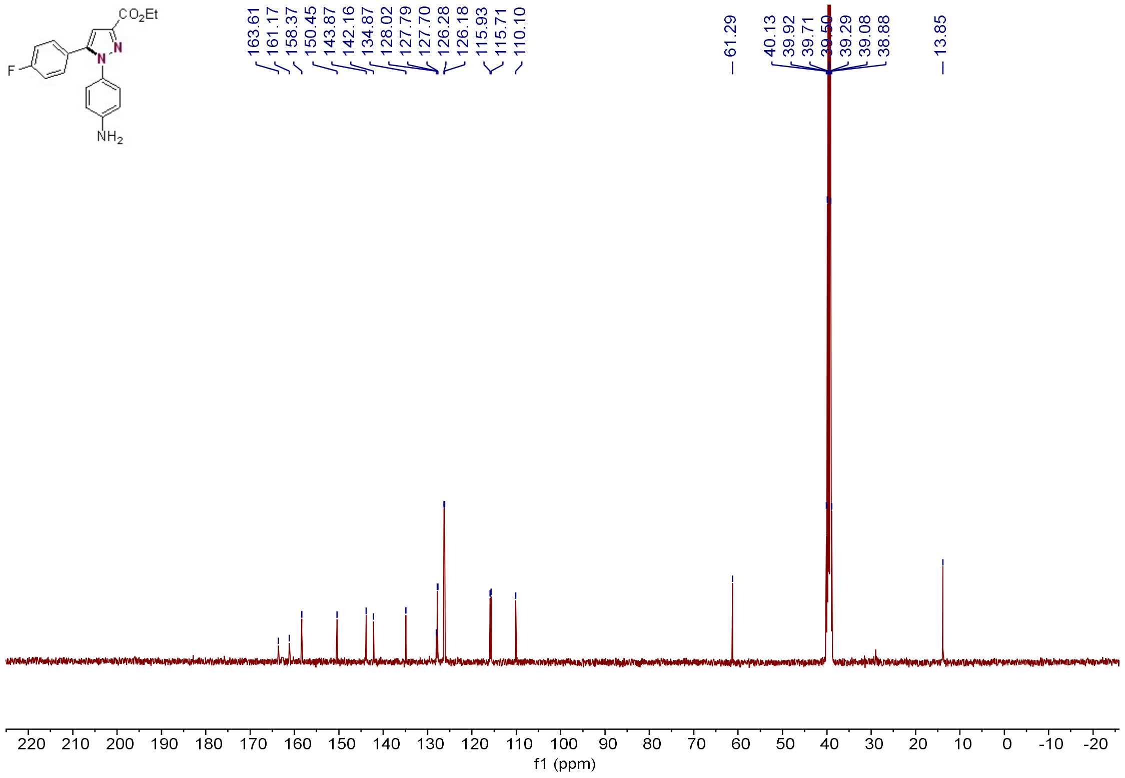

^{19}F NMR (376 MHz) Spectrum of 46 in d_6-DMSO

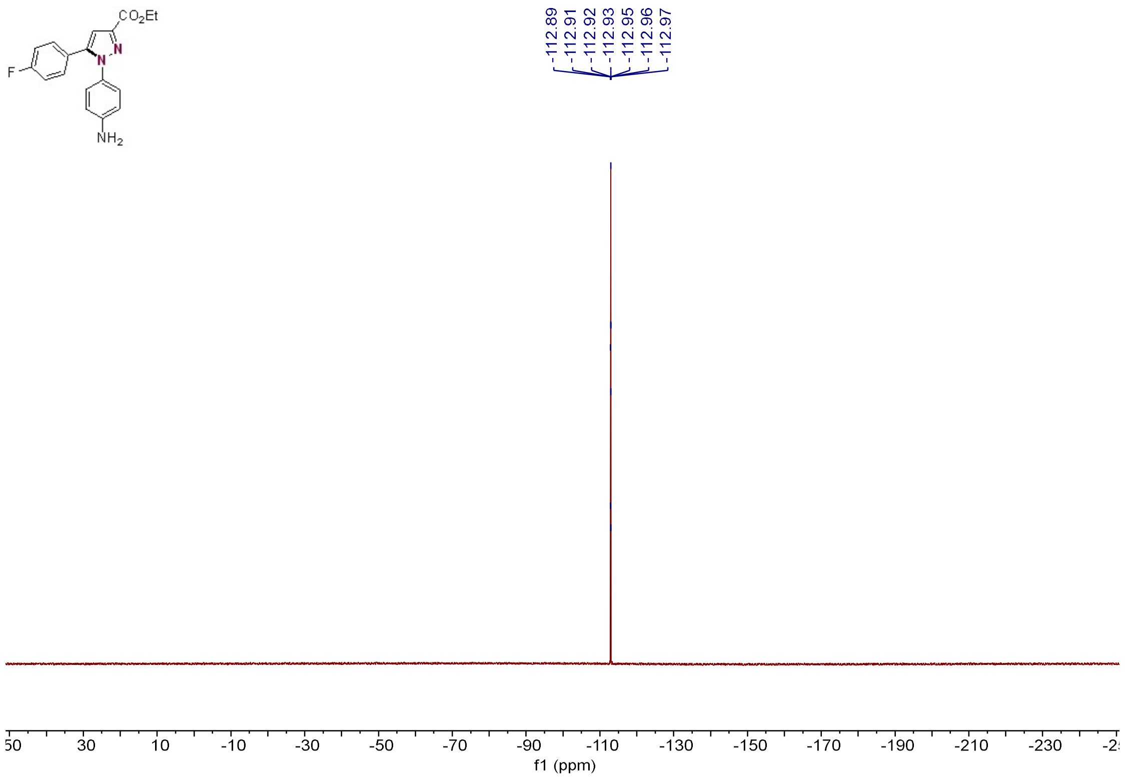

^{1}H NMR (400 MHz) Spectrum of 45 in $CDCl_3$

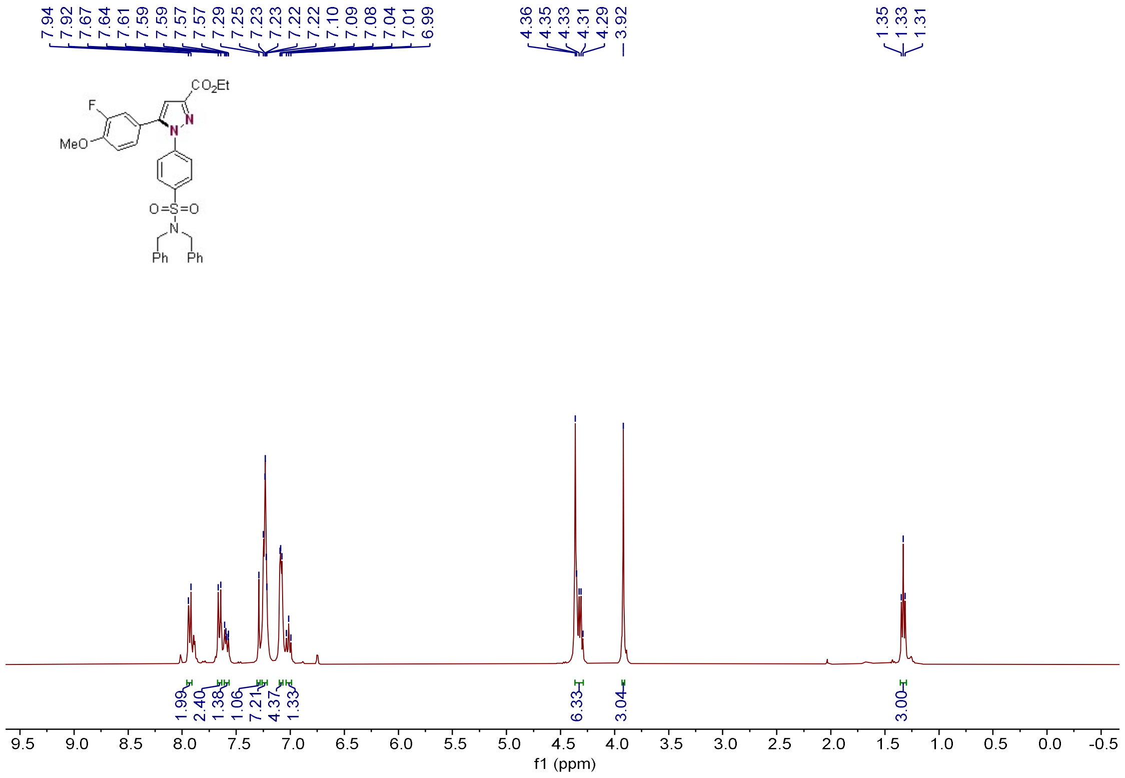

^{13}C NMR (100 MHz) Spectrum of 45 in CDCl$_3$

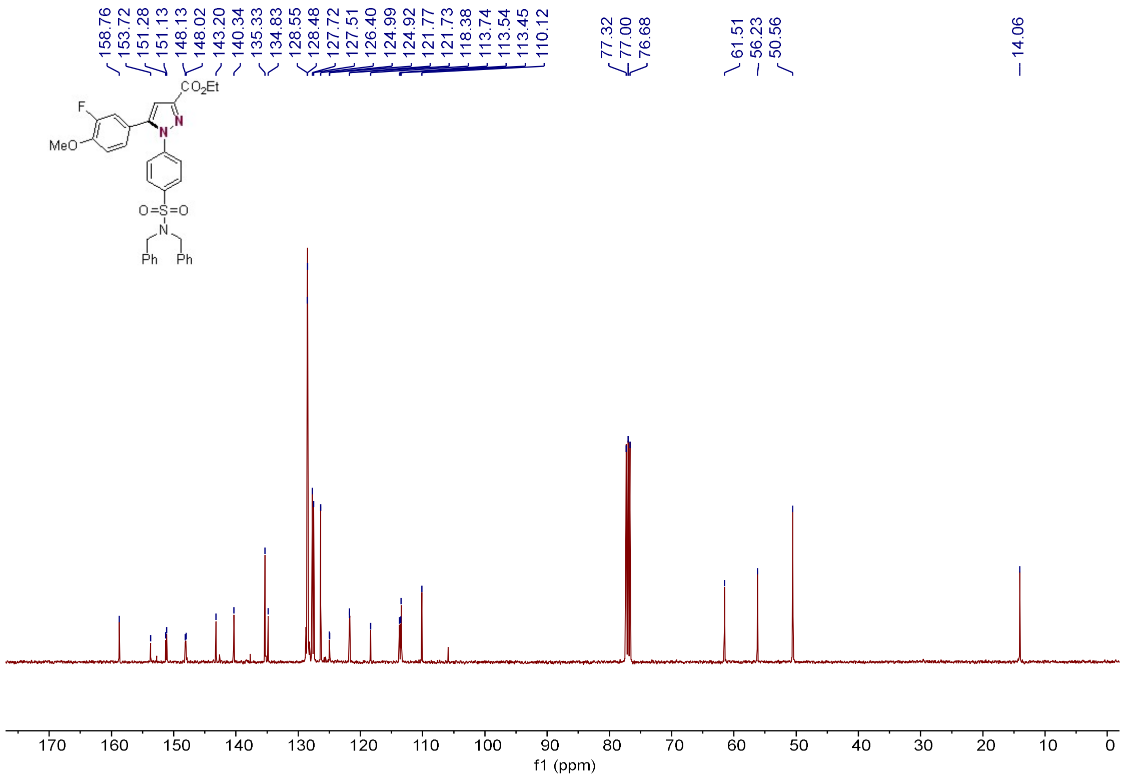

^{19}F NMR (376 MHz) Spectrum of 45 in CDCl$_3$

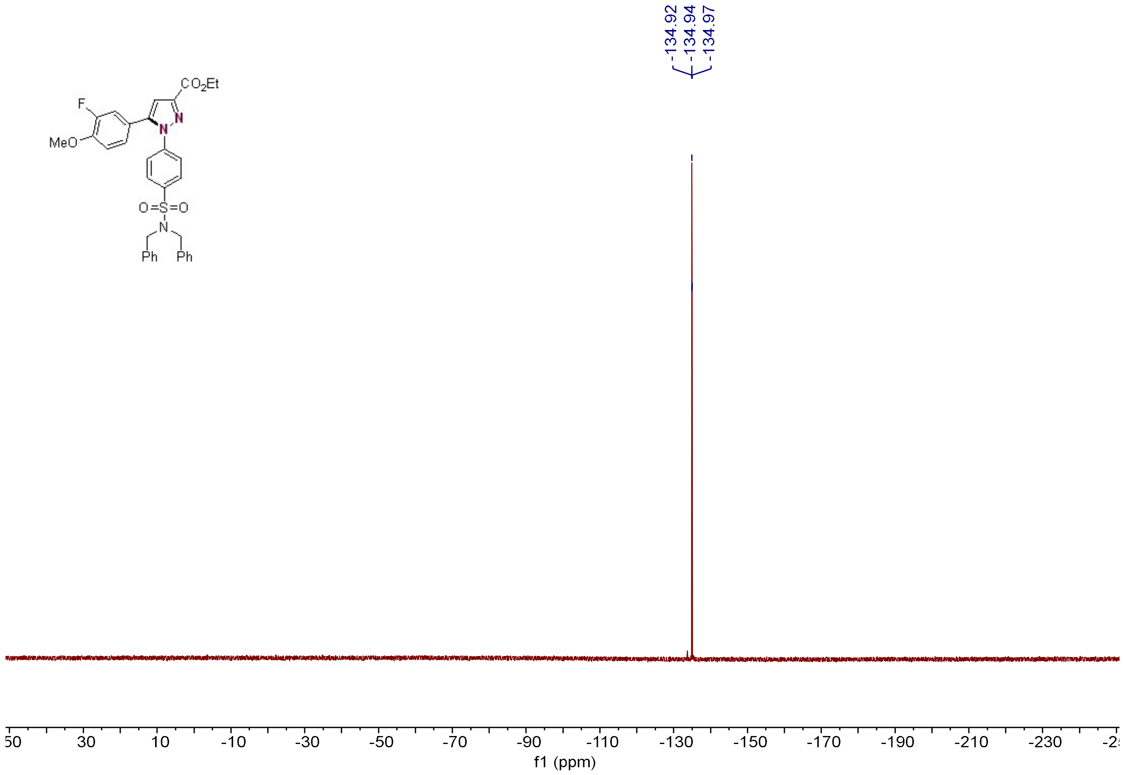

^{1}H NMR (400 MHz) Spectrum of 47 in d_6-DMSO

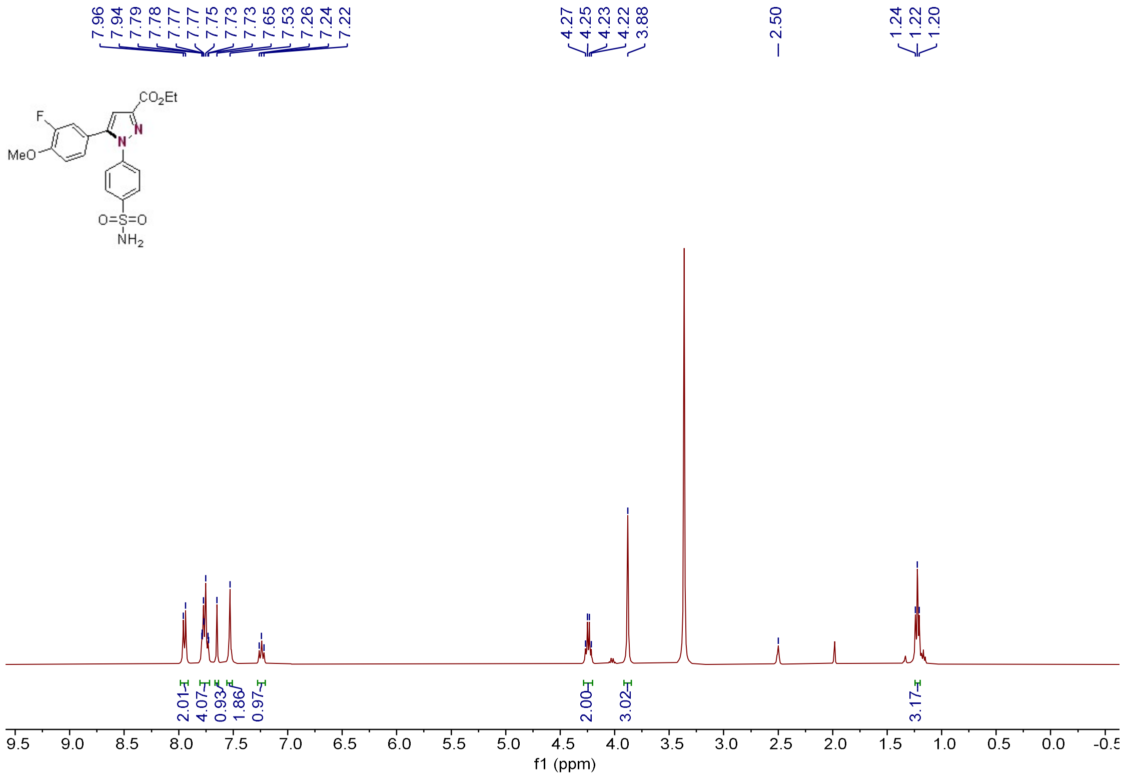

^{13}C NMR (100 MHz) Spectrum of 47 in d_6-DMSO

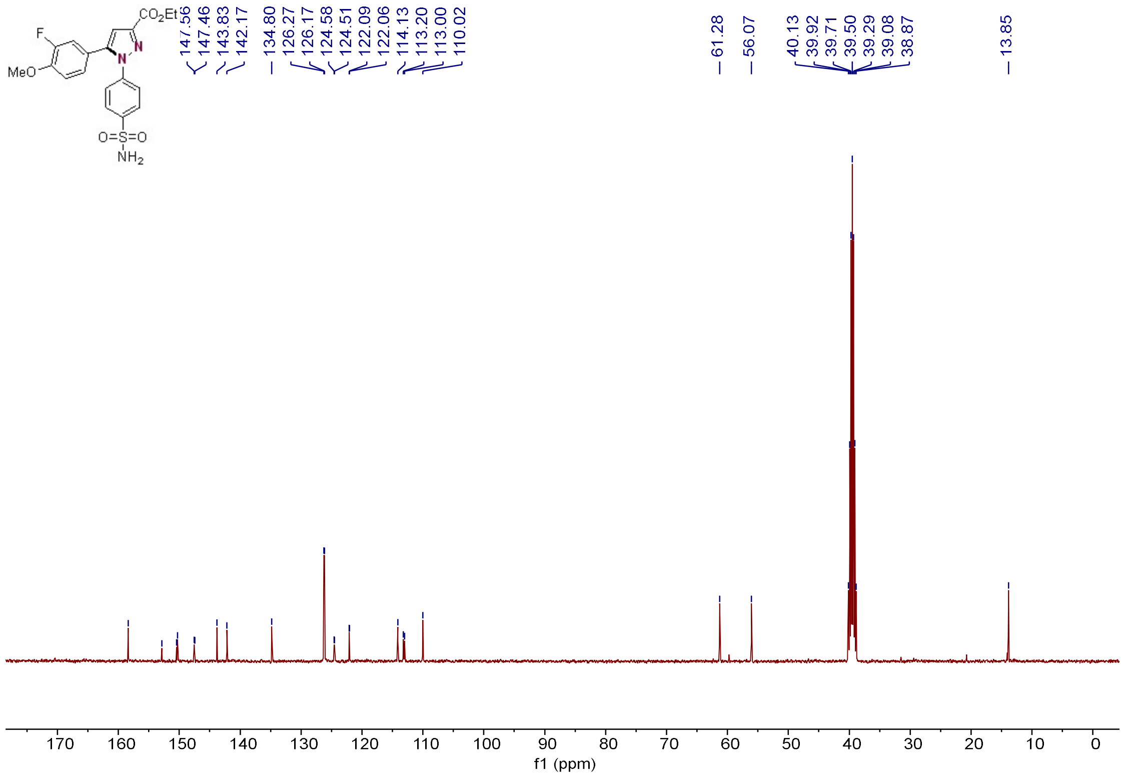

^{19}F NMR (376 MHz) Spectrum of 47 in d_6-DMSO

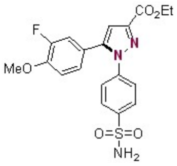

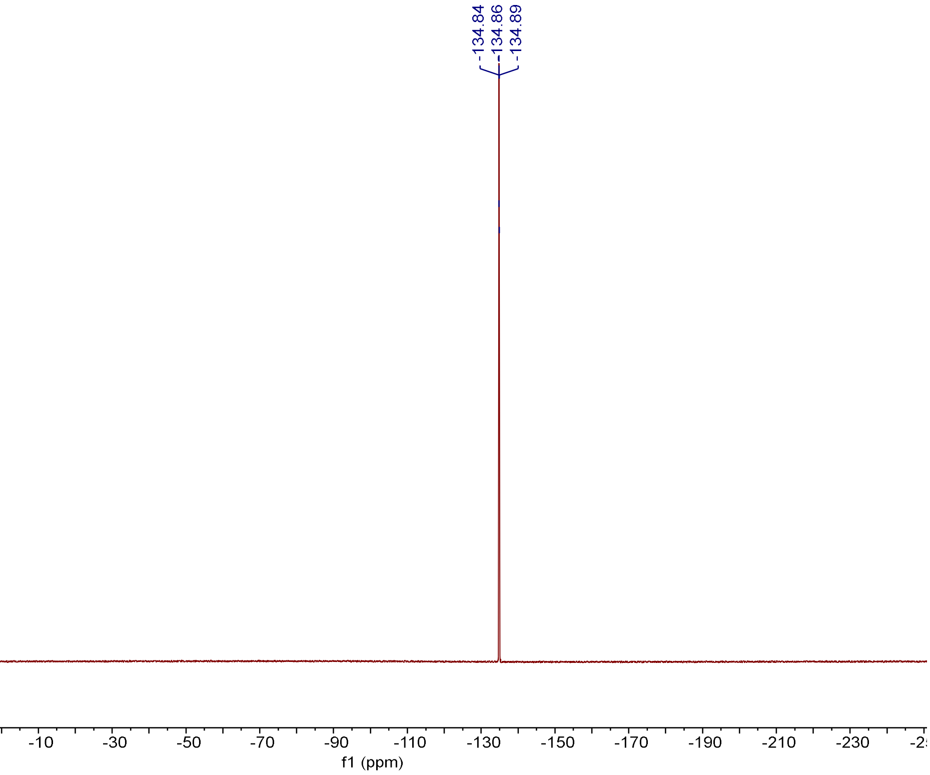

Supplement: Supplementary file 1 — Supporting Information [file ADVS-11-2306728-s001.pdf]
